# Genomic characterization of enterohaemolysin-encoding haemolytic *Escherichia coli* of animal and human origin

**DOI:** 10.1099/mgen.0.000999

**Published:** 2023-04-27

**Authors:** Katarzyna Sidorczuk, Adrianna Aleksandrowicz, Michał Burdukiewicz, Robert A. Kingsley, Rafał Kolenda

**Affiliations:** ^1^​ Department of Bioinformatics and Genomics, Faculty of Biotechnology, University of Wrocław, Wrocław, Poland; ^2^​ Department of Biochemistry and Molecular Biology, Faculty of Veterinary Medicine, Wrocław University of Environmental and Life Sciences, Wrocław, Poland; ^3^​ Clinical Research Centre, Medical University of Białystok, Białystok, Poland; ^4^​ Autonomous University of Barcelona, Institute of Biotechnology and Biomedicine, Cerdanyola del Vallès, Spain; ^5^​ Quadram Institute Biosciences, Norwich Research Park, Norwich, UK

**Keywords:** *Escherichia coli*, haemolytic, enterohaemolysin, alpha-haemolysin, adhesins, iron acquisition, toxin, EhxA

## Abstract

Enterohaemolysin (Ehx) and alpha-haemolysin are virulence-associated factors (VAFs) causing the haemolytic phenotype in *Escherichia coli *. It has been shown that chromosomally and plasmid-encoded alpha-haemolysin are characteristic of specific pathotypes, virulence-associated factors and hosts. However, the prevalence of alpha- and enterohaemolysin does not overlap in the majority of pathotypes. Therefore, this study focuses on the characterization of the haemolytic *E. coli* population associated with multiple pathotypes in human and animal infectious diseases. Using a genomics approach, we investigated characteristic features of the enterohaemolysin-encoding strains to identify factors differentiating enterohaemolysin-positive from alpha-haemolysin-positive *E. coli* populations. To shed light on the functionality of Ehx subtypes, we analysed Ehx-coding genes and inferred EhxA phylogeny. The two haemolysins are associated with a different repertoire of adhesins, iron acquisition or toxin systems. Alpha-haemolysin is predominantly found in uropathogenic *E. coli* (UPEC) and predicted to be chromosomally encoded, or nonpathogenic and undetermined *E. coli* pathotypes and typically predicted to be plasmid-encoded. Enterohaemolysin is mainly associated with Shiga toxin-producing *E. coli* (STEC) and enterohaemorrhagic *E. coli* (EHEC) and predicted to be plasmid-encoded. Both types of haemolysin are found in atypical enteropathogenic *E. coli* (aEPEC). Moreover, we identified a new EhxA subtype present exclusively in genomes with VAFs characteristic of nonpathogenic *E. coli *. This study reveals a complex relationship between haemolytic *E. coli* of diverse pathotypes, providing a framework for understanding the potential role of haemolysin in pathogenesis.

## Data Summary

The authors confirm all supporting data, code and protocols have been provided within the article or through supplementary data files.

1. Supplementary data containing all supplementary figures and tables (Supplementary Material).
2. Dataset S1: list of genomes used in this study (Supplementary Dataset S1).
3. Supplementary dataset 2: results of mapping of the contigs identified as plasmids by Platon to 306 reference plasmids from PLSDB containing enterohaemolysin (Supplementary Dataset S2).

**Impact Statement**

Enterohaemolysin and alpha-haemolysin belong to the family of RTX toxins that cause lysis of erythrocytes. Haemolysis on blood agar is used in bacteriology as part of routine diagnostics. Due to phenotypic and genotypic similarities, enterohaemolysin and alpha-haemolysin are often mistaken for each other. In this study, we characterize the haemolytic *E. coli* population of multiple pathotypes involved in human and animal infectious diseases. We show that these haemolysins are associated with a different repertoire of adhesins, iron acquisition or toxin systems. Alpha-haemolysin occurs in UPEC, nonpathogenic or undetermined *E. coli* pathotypes and may be chromosomally or plasmid-encoded. Enterohaemolysin is typically associated with STEC and EHEC and predicted to be plasmid-encoded. The only pathotype where both types of haemolysin are found is aEPEC. Moreover, we identify a new subtype of EhxA in genomes with a nonpathogenic-like virulence-associated factor profile and suggest an association between EhxA sequence variation and altered *E. coli* toxicity towards the host. These insights reveal a complex relationship between haemolytic *E. coli* of diverse pathotypes, providing a framework for understanding the potential role of haemolysin in pathogenesis.

## Introduction

*Escherichia coli* is a versatile bacterial species encompassing commensals and pathogens of animals and humans. The latter are highly adapted strains with acquired virulence attributes and infect hosts effectively, causing a wide range of intestinal and extraintestinal infections worldwide [1, 2]. Pathogenic *E. coli* are classified into intestinal (InPEC) and extraintestinal (ExPEC) based on the site of infection. Due to the characteristic virulence-associated factors/genes (VAFs/VAGs) and shared patterns of pathogenicity for particular hosts, they are further categorized into pathotypes (reviewed in detail in [2]). InPECs include enteropathogenic (EPEC), enterotoxigenic (ETEC), enteroaggregative (EAEC), enteroinvasive (EIEC), diffusely adherent (DAEC) and Shiga toxin-producing (STEC) *E. coli *, whereas ExPEC are categorized into uropathogenic (UPEC), sepsis-associated (SEPEC), neonatal meningitis (NMEC) and avian pathogenic (APEC) *E. coli* strains [1, 3].

The virulence of microorganisms, including *E. coli *, is a multistep interaction between bacterial and host cells involving numerous VAFs, comprising both extra- and intracellular structures [4]. The ability of bacteria to colonize host tissue is determined by VAFs such as adhesins, iron acquisition systems and toxins [5]. The primary role of adhesins is binding to epithelial cells, resulting in invasion and ultimately leading to host colonization. Some may also contribute to the interactions between bacteria and abiotic surfaces during biofilm formation enabling survival in diverse conditions and environments [6, 7]. The current classification system distinguishes fimbrial and nonfimbrial adhesins [8]. However, the adhesion process may also involve structures and mechanisms indirectly contributing to the binding and colonization. They are known as atypical adhesins and include flagella, lipopolysaccharide, and type III and VI secretion systems (T3SS, T6SS) [9].

The expression of genes encoding iron acquisition and storage systems allows bacteria to survive in iron-deficient environments [10]. The most relevant and effective mechanisms to acquire iron during infection include the production of siderophores, iron transporters and haem/hemin uptake systems [11]. Alternatively, bacteria secrete haemolysins to lyse red blood cells and release haemoglobin, and/or produce haemoglobin proteases that make the iron available [12].

Three types of haemolysin have been described in *E. coli *, alpha-haemolysin (α-Hly), enterohaemolysin (Ehx/EHEC-hly) and silent haemolysin (HlyE) [13]. α-Hly is encoded by an operon consisting of four genes – *hlyCABD*. The *hlyA* gene encodes a functional cytotoxic haemolysin following activation by the product of *hlyC*. In turn, products of *hlyB* and *hlyD*, along with the outer-membrane channel TolC, are involved in the secretion of haemolysin through the bacterial cell envelope [14]. α-Hly exhibits cytotoxic activity against various types of cells, including erythrocytes, monocytes, granulocytes, endothelial and epithelial cells. Ehx is encoded on a plasmid by an operon consisting of *ehxC*, *ehxA*, *ehxB* and *exhD* genes with functions analogous to *hlyC*, *hlyA*, *hlyB* and *hlyD*, respectively [15]. Ehx targets erythrocytes, which after lysis release haemoglobin and haem, positively influencing *E. coli* proliferation and growth. The role of haemolysis in *E. coli* pathogenicity and its association with other virulence factors and disease outcome remains inconclusive. We recently reported the association of VAF repertoire with the context of the *hlyCABD* cluster in the genome and demonstrated that chromosomally and plasmid-encoded α-Hly are characteristic of specific pathotypes and hosts [13]. Chromosomally encoded α-Hly was associated with UPEC strains from human infections, whereas plasmid-encoded α-Hly was found in nonpathogenic *E. coli* and less often in various intestinal pathotypes isolated from animals. Moreover, haemolytic *E. coli* with plasmid-encoded α-Hly showed a similar adhesin profile to nonpathogenic strains, whereas chromosome-encoded α-Hly had a high prevalence of Auf, P and S fimbriae characteristic for UPEC [13].

Based on our previously published analysis, we tested the hypothesis that genome context of Ehx genes is related to the virulence factor repertoire of Ehx-positive *E. coli *, as was observed for α-Hly-positive *E. coli *. Moreover, we tested whether Ehx-positive strains possess genomic signatures not found in α-Hly-positive *E. coli* to provide crucial information for the differentiation of function of Ehx and α-Hly. Using a genomics approach, we investigated characteristic features of the genome of Ehx-encoding *E. coli* isolated from animals and humans to provide a one-health perspective on these pathogens. Finally, to shed light on the prevalence of Ehx variants and subtypes, we performed an in-depth analysis of Ehx coding genes and EhxA phylogeny.

## Methods

### Genome acquisition and analysis

A RefSeq collection of *E. coli* genomes was screened for the presence of enterohaemolysin-encoding genes *ehxCABD* (from reference genome EDL 933; GenBank accession number X86087.1), yielding 2399 enterohaemolysin-positive genomes (Supplementary Dataset S1, available in the online version of this article). Additionally, the collection of 1122 *hlyCABD-*positive (alpha-haemolysin-positive) *E. coli* genomes was obtained from Kolenda *et al*. [13] to allow comparative studies (Supplementary Dataset S2). The genomic context of alpha-haemolysin-coding genes was determined as in Kolenda *et al*. [13]. Clermont typing was performed with the use of clermonTyping to determine the phylogroup of *E. coli* under analysis [16]. Multilocus sequence typing (MLST) was determined with the PubMLST database and mlst software [17]. *In silico* serotyping was performed with ABRicate using the EcOH database [18]. Bayesian analysis of population structure (BAPS) was carried out by fastBAPS with use of optimized symmetric prior to hyperparameter optimization [19]. Pathotypes were predicted for *ehxCABD*-positive *E. coli* genomes by identification of VAG profile with blast, as listed in Table S1 [13]. Genomes that did not fit the criteria for any pathotype were classified as unknown (na). The genomic context of enterohaemolysin-coding genes was analysed using blast by comparing reference sequences of 5′-upstream of *ehxC* and 3′-downstream of *ehxD* (Table S2). Reference sequences of 5′-upstream of *ehxC* and 3′-downstream of *ehxD* specific only for plasmid-encoded enterohaemolysin were selected by analysis with use of complete chromosome and plasmid sequences in the Nucleotide collection (nr/nt) database using blast. Platon software with default settings was used as additional confirmation that *ehxCABD* operon is encoded on a plasmid [20]. First, contigs that encode plasmid sequences were identified with Platon and next they were analysed with blast for the presence of *ehxCABD* (from reference genome EDL 933; GenBank accession number X86087.1). Additionally, contigs identified as plasmids/part of a plasmid with Platon were searched against 306 plasmids encoding *ehxCABD* identified in PLSDB (described in paragraph ‘Analysis of plasmids’). All blast results with identity and query coverage equal to or above 95 % were considered positive. The pangenome of *ehxCABD*-positive *E. coli* was analysed via Roary. Variable sites were extracted from core gene alignment generated in Roary with SNP-sites and used in phylogenetic analysis with FastTree 2.1 [21, 22]. The phylogenetic tree was annotated by iTOL [23].

### Comparison of gene frequency between *hlyCABD*- and *ehxCABD*-positive *E. coli*

The pangenome of *hlyCABD*- and *ehxCABD*-positive *E. coli* was determined with the use of Roary [24]. Genes in the pangenome were functionally annotated by using eggnog-mapper [25]. Gene frequencies between *hlyCABD*- and *ehxCABD*-positive *E. coli* were compared with Scoary [26]. Clusters of orthologous groups (COGs) were compared between *hlyCABD*- and *ehxCABD*-positive *E. coli* for all genes with a Bonferroni *P*-value <0.001 in Scoary analysis. Comparison between numbers of genes belonging to the same functional group between *hlyCABD*- and *ehxCABD*-positive *E. coli* was performed with the chi-squared test of independence implemented in R stats package [27].

### Adhesin, toxin and iron acquisition gene frequency determination

*E. coli* genomes were tested for adhesins, toxins and iron acquisition genes as published in Kolenda *et al*. [13] using 567 genes coding for adhesins, toxins and iron acquisition collected from GenBank database. Gene frequency in analysed genomes was tested with ABRicate. The genes from the same system or with similar functionality were presented as one VAF, as shown in Table S3. System prevalence was compared with chi-squared test of independence implemented in the R stats package.

### Analysis of plasmids

A plasmid database, PLSDB, was queried for the presence of alpha-haemolysin or enterohaemolysin genes [28]. Sequences of *hlyCABD* were acquired from *E. coli* strain UTI89 (GenBank accession number CP000243.1) and sequences of *ehxCABD* genes were obtained from *E. coli* strain EDL933 (GenBank accession number X86087.1). Pangenomes for plasmid sequences were determined with Panaroo [29]. Functional annotation of plasmid genes was performed with the eggnog-mapper [25]. Comparison between numbers of genes belonging to the same functional group in haemolysin was performed with the Kruskal–Wallis test. Gene presence and absence was utilized in hierarchical clustering of plasmids with use of hclust from R package stats.

### Analysis of EhxCABD cluster

Sequences of *ehxCABD* genes were extracted via blast and the sequence of *E. coli* strain EDL933 (GenBank accession number X86087.1) was used as reference. Next, the nucleotide sequences were translated by UGENE [30]. Variant clustering was carried out by CD-HIT [31]. Protein sequences were considered the same variant if they were 100 % identical and had the same length. Protein functionality was assessed based on protein length and all variants with a sequence at least 1 % shorter than the most frequent variant were considered non-functional. EhxA phylogeny was performed using RAxML and employing a PROTGAMMADUMMY2 substitution model and 500 bootstrap replications [32]. The EhxA tree was annotated with iTOL [23]. Subtyping of *ehxA* gene was based on TaqI restriction digestion of *ehxA* gene sequence as described by Lorenz *et al*. [33]. TaqI restriction digestion of *ehxA* gene sequences was performed *in silico* with the use of DigestDNA function from DECIPHER R package [34].

## Results

### Characterization of enterohaemolysin-positive *E. coli* genomes

**Fig. 1.**
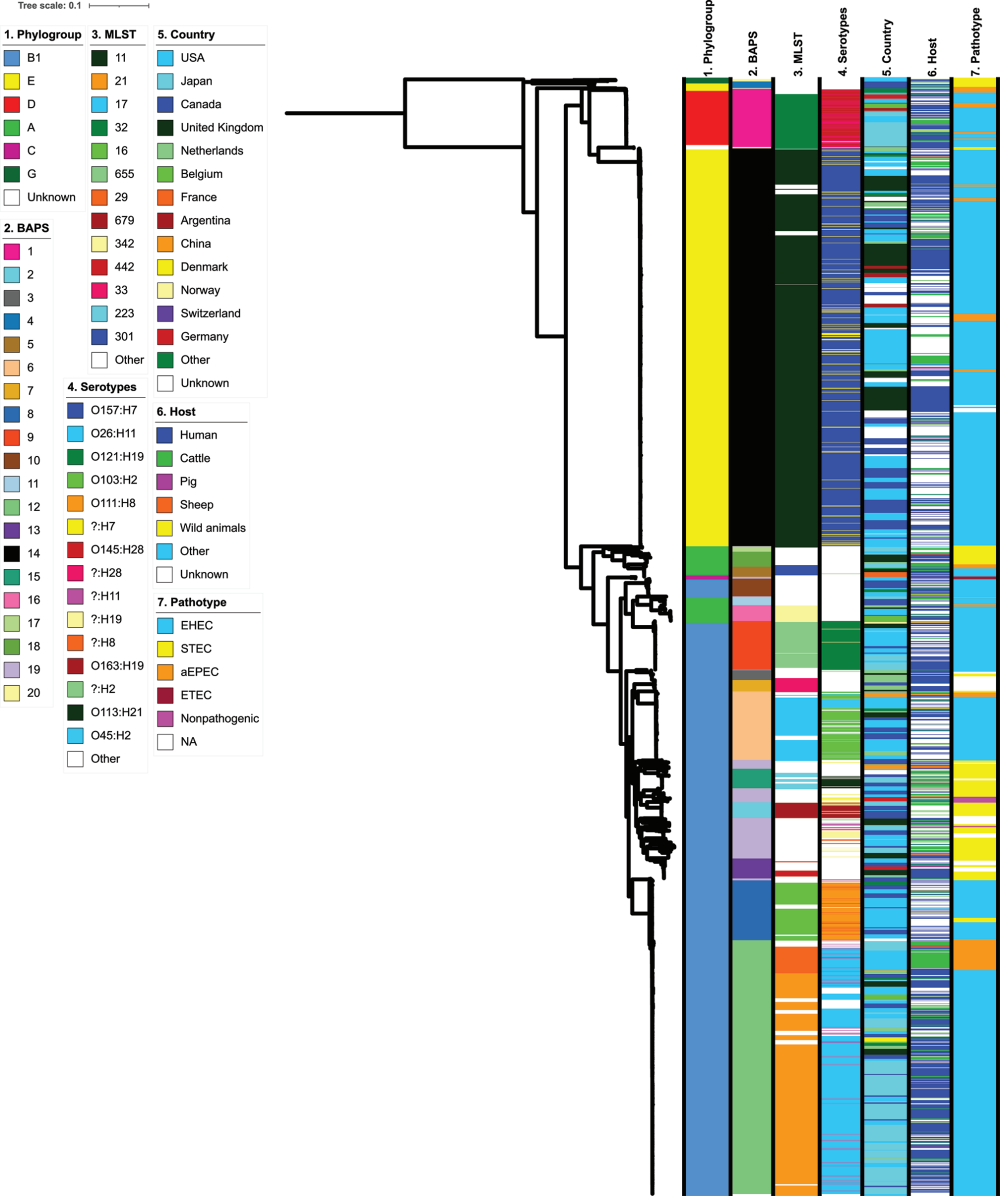
Phylogenetic relationship of 2399 genomes of enterohaemolytic *E. coli *. Phylogroup, BAPS group, MLST, serotype, origin, host and pathotype have been annotated on the midpoint-rooted tree using iTOL.

RefSeq database contained 2399 *E. coli* genomes positive for 4 enterohaemolysin genes, which were categorized into 6 phylogroups (Fig. 1). More than half (52%) of the analysed strains belonged to the B1 phylogroup, whereas phylogroup E had 36 % isolates. Phylogroups D, A, C and G were much less prevalent, consisting of 5.35, 4.96, 0.17 and 0.17 % of strains, respectively (Fig. 1). Investigation of the genetic structure with BAPS revealed 20 clusters, 11 of which belonged to phylogroup B1, whereas E1 and D phylogroups were nearly homogenous, represented mainly by clusters 14 and 1, respectively (Figs 1 and S1a). *In silico* prediction of the serotype formula identified 136 serotypes, of which only 15 were represented by 20 or more genomes (Figs 1 and S1c). The strains were isolated from diverse geographical locations, including 31 countries across 6 continents, although the majority (84 %) were isolated in the USA (34.4 %), Japan (18.1 %), Canada (17.9 %) and the UK (13.4 %) (Figs 1 and S1b). Considering the isolation source, they were obtained mainly from humans and cattle (Figs 1 and S1e). The enterohaemolysin-positive *E. coli* strains were of 149 STs, highlighting the widespread distribution of enterohaemolysin genes within *E. coli* (Figs 1 and S1f). Pathotype prediction revealed that most strains were represented by EHEC (77.9 %), followed by STEC (11.9 %) and atypical enteropathogenic *E. coli* (aEPEC) (6.3 %) (Fig. 1). The least prevalent groups were nonpathogenic *E. coli* and ETEC, with 0.7 and 0.04 % of the total, respectively. Analysis of the genomic context of enterohaemolysin clusters by comparing reference sequences of 5′-upstream of *ehxC* and 3′-downstream of *ehxD* suggests that it can only be found on plasmids, but the context of *ehxCABD* could not be determined for 17.7 % of genomes with that method (Fig. S2). Additional analysis using Platon software revealed that the majority of contigs (from 95 % of strains) that contain *ehxCABD* are identified as plasmid sequences. Mapping of the contigs identified as plasmids by Platon to 306 reference plasmids from PLSDB showed that contigs from 84.7 % of genomes can be aligned to at least 1 of the plasmids (Supplementary Dataset S2).

### Comparison of enterohaemolysin- and alpha-haemolysin-positive *E. coli*

In order to obtain a comprehensive overview of alpha-haemolysin- and enterohaemolysin-positive *E. coli* populations, their genomes were compared with respect to phylogroups, BAPS clusters, STs and serotypes. All isolates were present in one of 6 phylogroups – A, B1, B2, C, D and E – and further subdivided into 29 clusters defined by hierarchical Bayesian analysis (Fig. S3a). The majority of *hlyCABD-*positive isolates (65 %) belonged to B2, while more than half of *ehxCABD*-positive strains represented the B1 phylogroup (52 %). Both types of haemolysins displayed a comparable number of STs and serotypes (Fig. S3c, e), indicating their similar genetic and antigenic diversity. Twenty-four of 275 STs were prevalent in both groups and comprised 1102 isolates in total. Twenty-two out of 303 serotypes were commonly identified in 901 genomes from both haemolysin groups. *HlyCABD*-positive genomes deposited in the RefSeq database were more diverse in terms of country of isolation than *ehxCABD*-positive genomes (Fig. S3b). The investigations of genome context of *hlyCABD* and *ehxCABD* suggested that most alpha-haemolytic isolates from the B2 and D phylogroup had alpha-haemolysin encoded on chromosomes (Fig. S3d). Regarding the B1 and E phylogroup, more than 70 % of enterohaemolysins and alpha-haemolysins were predicted to be plasmid-encoded, similarly to alpha-haemolysins from these groups. In phylogroup A, enterohaemolysins were predicted to be plasmid-encoded, whereas alpha-haemolysins as encoded on a chromosome or plasmid. In the case of group C, the determination of *ehxCABD* context was not achieved. Comparison of genome context with the host origin of strains revealed that animal isolates mainly possessed plasmid-encoded haemolysins of both types, whereas chromosomally encoded toxins were considerably more common (76 %) for alpha-haemolytic human isolates (Fig. S3g).

**Fig. 2.**
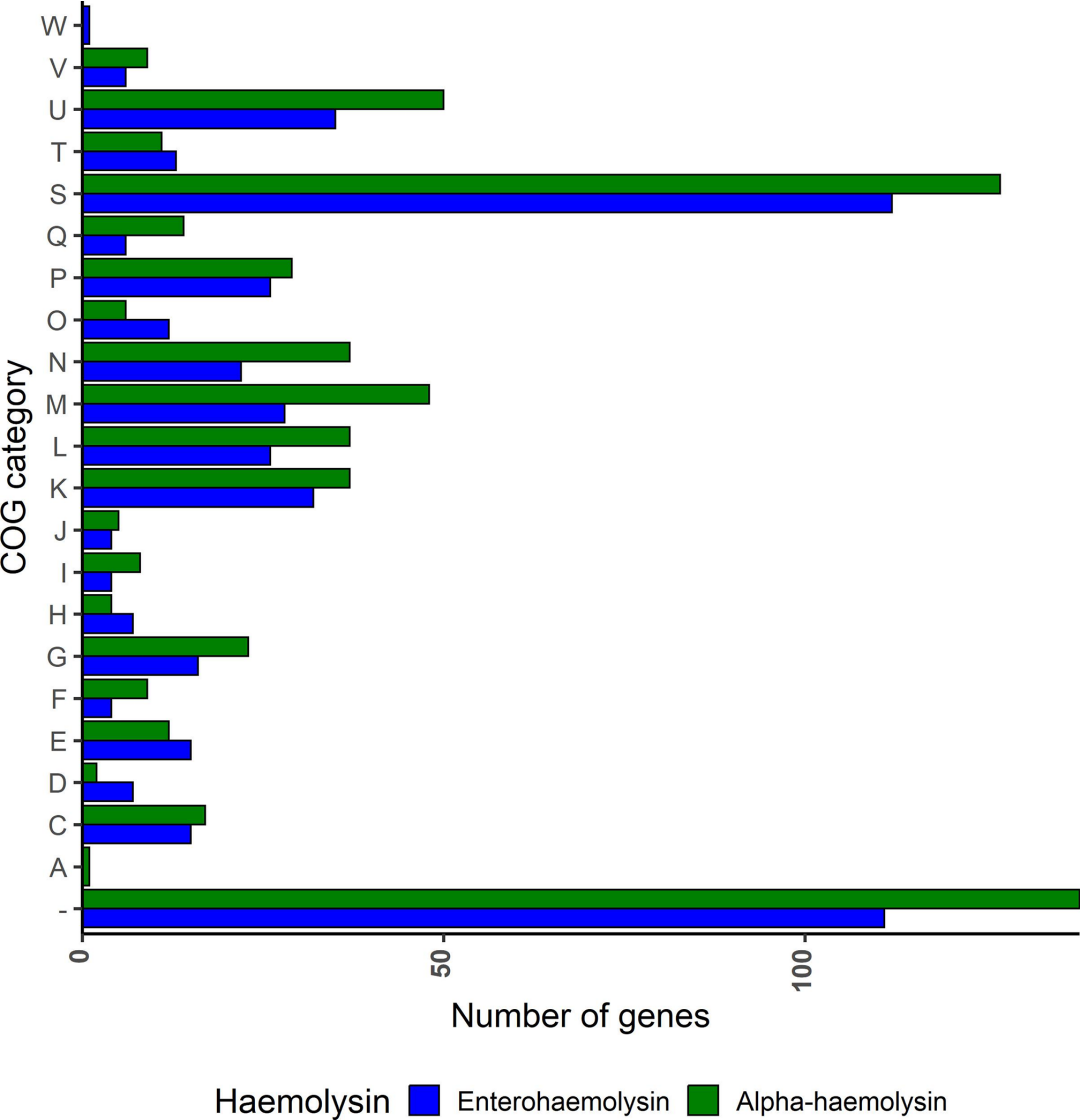
Functional annotation of genes with different prevalence in alpha-haemolysin- and enterohaemolysin-positive *E. coli *. Barplot with gene frequency in clusters of orthologous groups of alpha-haemolysin- and enterohaemolysin-positive *E. coli *. Genes with a different frequency between *E. coli* with alpha-haemolysin and enterohaemolysin were functionally annotated with eggnog-mapper. The number of genes from a particular COG is shown on the *x*-axis. COG groups are shown on the *y*-axis. Bar colours represent different haemolysins and are described in the legend below the plot.

The pangenome of *hlyCABD*- and *ehxCABD*-positive *E. coli* was determined and used to annotate gene function classification associated with strains encoding each haemolysin. Based on inferred gene function, all genes with different frequencies in *hlyCABD*- and *ehxCABD*-positive *E. coli* were divided into 22 functional groups. Differences in gene frequency between alpha-haemolysin- and enterohaemolysin-positive *E. coli* were found in five groups, unassigned (−), M, N, Q and U (Fig. 2). Of note, these COGs were identified more frequently in *hlyCABD*-positive strains. The largest group (unassigned) covered genes whose function was not found in the eggNOG database. This group had more than 10 % higher prevalence in alpha-haemolytic strains compared to enterohaemolytic strains (*P*<0.005, chi-squared test). The second group (Q) with the highest differences in frequency between both *E. coli* groups contained genes associated with secondary metabolite biosynthesis or transport and catabolism processes (*P*<0.05, chi-squared test, Fig. S4). Additional subgroups of gene function were distinguished in group Q, with most genes encoding proteins possessing oxidoreductase activity and/or belonging to the short-chain dehydrogenases/reductases (SDRs) family. Their functions also included amino acid polymerization and lipoteichoic acid biosynthesis. Moreover, alpha-haemolytic strains were characterized by the higher prevalence of genes responsible for intracellular trafficking, secretion and vesicular transport (U, *P*<0.05, chi-squared test), encoding chaperone–usher proteins, fimbrial and fimbrial-like adhesins, and proteins associated with type II and III secretion systems (Fig. S4). Genes with similar functions were found in COG N, where adhesin-encoding proteins were identified (*P*<0.01, chi-squared test, Fig. S4). Another functional class (M) included genes constituting a component of cell wall and outer membrane structures and biogenesis, i.e. various enzymes, transporters, chaperone–usher and outer-membrane proteins (*P*<0.005, chi-squared test, Fig. S4).

### Alpha-haemolysin- and enterohaemolysin-positive *E. coli* differ in profiles of adhesin, toxin and iron acquisition systems frequency

**Fig. 3.**
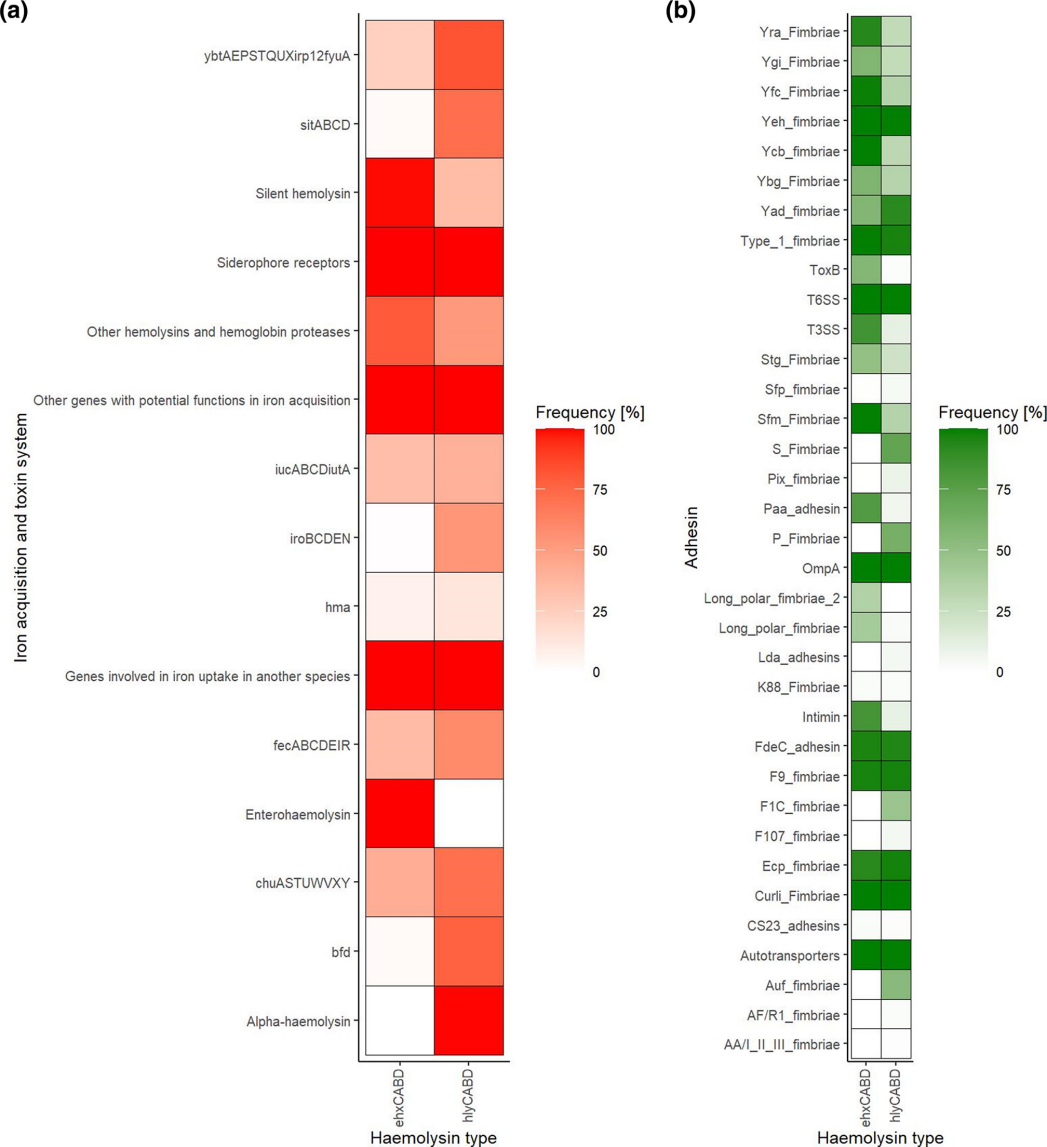
Frequency of toxin, iron acquisition and adhesin systems in alpha-haemolytic and enterohaemolytic *E. coli *. The frequency of 128 toxins and iron acquisition and 284 adhesin genes providing information about the presence of 15 toxins or iron acquisition systems and 50 adhesins or adhesion-related molecules was tested in 1122 alpha-haemolytic and 2399 enterohaemolytic *E coli *. Genome context is shown on the *x*-axis. Iron acquisition and toxin systems (**a**) or adhesins (**b**) are listed on the *y*-axis. The colour gradient is proportional to the frequency of each system, and the colour scale is shown on the legends attached to heatmaps. In the case of adhesins (**b**), each system that had a prevalence lower than or equal to 1 % in both groups was not included in the plot.

Since our comparison of predicted gene functional classes associated with haemolysins revealed differences in adhesion and toxin secretion functions (Figs 2 and S4), we next investigated the prevalence of adhesins, toxins and iron acquisition systems using a manually curated set of genes with confirmed functions. Overall, 65 VAFs encoded by 412 VAGs were identified in at least 1 isolate. The VAFs consisted of 50 adhesins and 15 toxin or iron acquisition system genes (Fig. 3). However, the prevalence between alpha-haemolytic and enterohaemolytic *E. coli* differed for 33 VAFs (*P*<0.05, chi-squared test). *E. coli* from both groups had genes encoding a silent haemolysin with different prevalence, 34.6 % among *hlyCABD*-positive strains and 99.3 % in *ehxCABD*-positive strains. Two VAGs associated with iron acquisition and storage systems, i.e. *pic* and *espC* belonging to the group ‘Other haemolysins and hemoglobin proteases’, were present more frequently in *hlyCABD*-positive isolates (*P*<0.001, chi-squared test). Regarding adhesins, Auf, S, P, F1C, Yad, Pix, AF/RI, Sfp and Ecp fimbriae had a higher prevalence in *hlyCABD-*positive strains (*P*<0.001, chi-squared test). Enterohaemolytic strains had a higher prevalence of intimin, Paa, Sfm, T3SS, Ycb, Yfc, Yra, ToxB, Lpf, Lpf2, Ygi, Stg and Ybg fimbriae (*P*<0.001, chi-squared test). The frequency of 29 out of 65 VAFs was similar in all isolates. Three of them were associated with iron acquisition systems and included agents involved in iron uptake in other bacterial species, other factors with potential functions in iron acquisition and siderophore receptors. The remaining 26 VAFs encoded fimbrial and nonfimbrial adhesins, including autotransporters group. Interestingly, the investigation of single genes encoding autotransporters revealed differences in the prevalence of *ehaA*, *ehaG*, *ehaB*, *espP*, *cah*, *ypjA*, *yeeJ*, *ycgV* and *flu* (Fig. S5, *P*<0.05, chi-squared test). Only the *flu* gene that encoded an autotransporter was found more frequently in alpha-haemolytic *E. coli *.

### High genetic diversity defines plasmids bearing alpha-haemolysin and enterohaemolysin

**Fig. 4.**
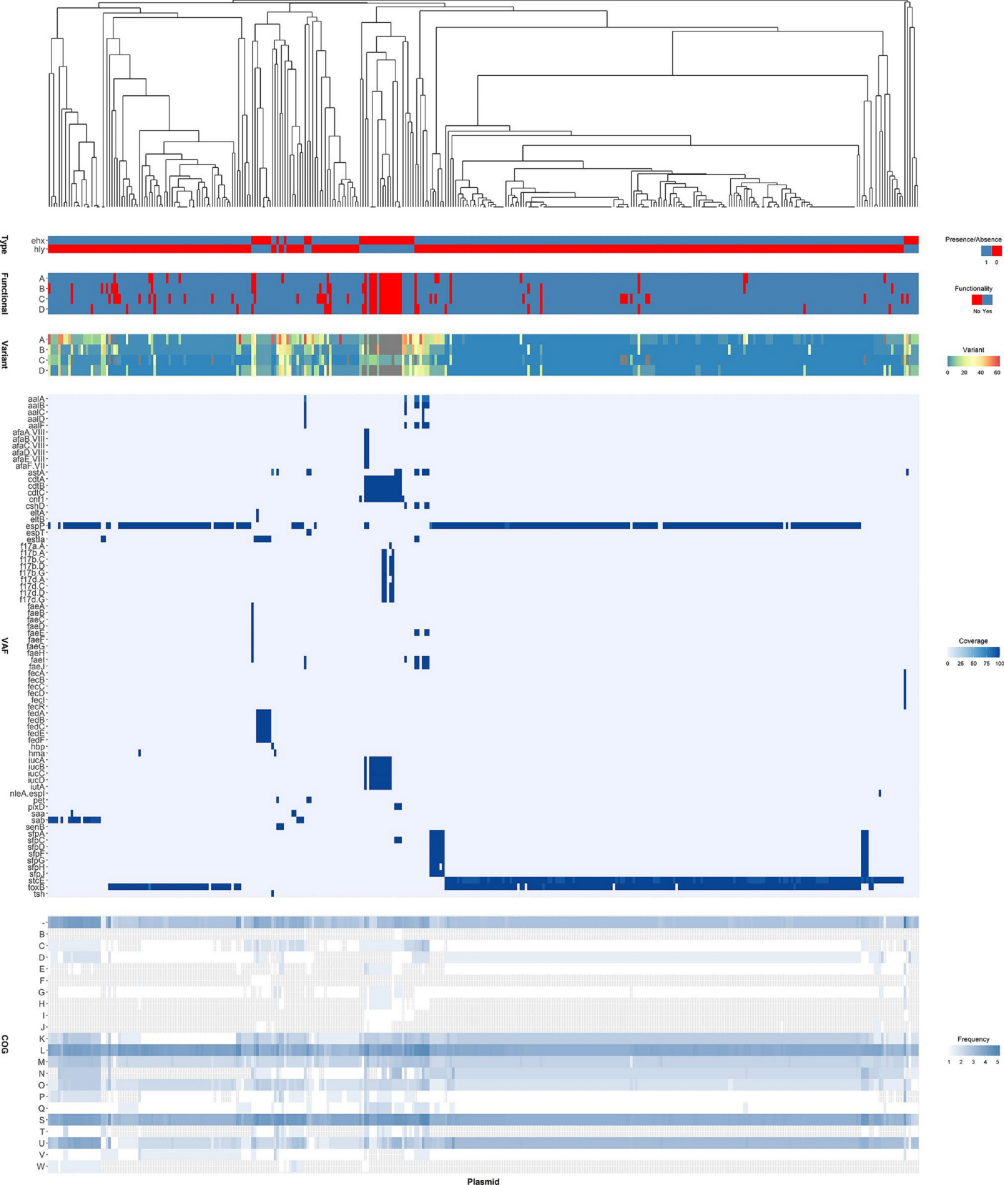
Comparison of alpha-haemolysin- and enterohaemolysin-bearing plasmids. Analysis of gene presence, haemolysin variation and functionality, VAFs and COGs in alpha-haemolysin- and enterohaemolysin-bearing plasmids. Sequences of 41 alpha-haemolysin- and 306 enterohaemolysin-bearing plasmids were used to generate the pangenome with Panaroo. Next, a gene presence/absence tree was generated and annotated with: haemolysin type (ehx, enterohaemolysin; hly, alpha-haemolysin), haemolysin protein variation and functionality (A, EhxA or HlyA; B, EhxB or HlyB; C, EhxC or HlyC; D, EhxD or EhxD), VFDB VAF coverage and COG frequency. For each heatmap, the plasmid is set on the *x*-axis and haemolysin type, haemolysin protein, VAFs or COGs are shown on the *y*-axes. Legends with colour scales are provided on the right side of each heatmap.

Considering that both haemolysins can be located on the plasmids, the diversity of these DNA molecules was assessed. All plasmids that contained genes from analysed haemolysins were downloaded from the PLSDB plasmid database. There were 41 and 306 plasmids that encoded at least 1 gene from the alpha- or enterohaemolysin operon, respectively. Most plasmids were circular (Fig. S6c). Their length and GC content varied from 44 632 to 242 187 bp and from 43.2 to 52.4 %, respectively (Fig. S6d, e). Plasmid typing with PlasmidFinder and pMLST revealed that alpha- and enterohaemolysins are mainly found on different plasmid types (Fig. S6a, b), with both haemolysins only occurring in six PlasmidFinder groups (Fig. S6a). Analysis of pangenomes for alpha- and enterohaemolysin-bearing plasmids revealed high diversity in gene content among both groups. The pangenome of alpha-haemolysin-positive plasmids contained 1234 genes, similarly to enterohaemolysin-positive plasmids, which encoded 1160 genes. For most plasmids, the only genes common in the pangenomes were the haemolysin-coding genes. Functional annotation and analysis of plasmid genes revealed differences in the frequency of genes belonging to 15 functional groups between alpha-haemolysin and enterohaemolysin-positive plasmids (Figs 4 and S7, Table S4). Moreover, clustering based on gene presence/absence showed that plasmids from these haemolysin types grouped separately, which was associated with differences in VAF prevalence (Fig. 4). Additionally, gene presence/absence was also associated with haemolysin protein variations and functionality. Up to three alpha-haemolysin genes (*hlyABD*) were lost in 13 plasmids that clustered together, whereas the *ehxC* gene from enterohaemolysin was not found in 14 plasmids. Furthermore, the investigation of gene functionality, i.e. if it encodes a full-length protein without frameshifts, identified that 17 and 57 (41.5 and 18.6 %, respectively) plasmids with alpha-haemolysin and enterohaemolysin encoded potentially non-functional haemolysins. Taken together, the data indicate that alpha-haemolysin and enterohaemolysin can be encoded on heterogeneous groups of plasmids, which share similar characteristics dependent on the haemolysin type they encode.

### Global diversity of enterohaemolysin reveals pathotype-associated variants and subtypes

**Fig. 5.**
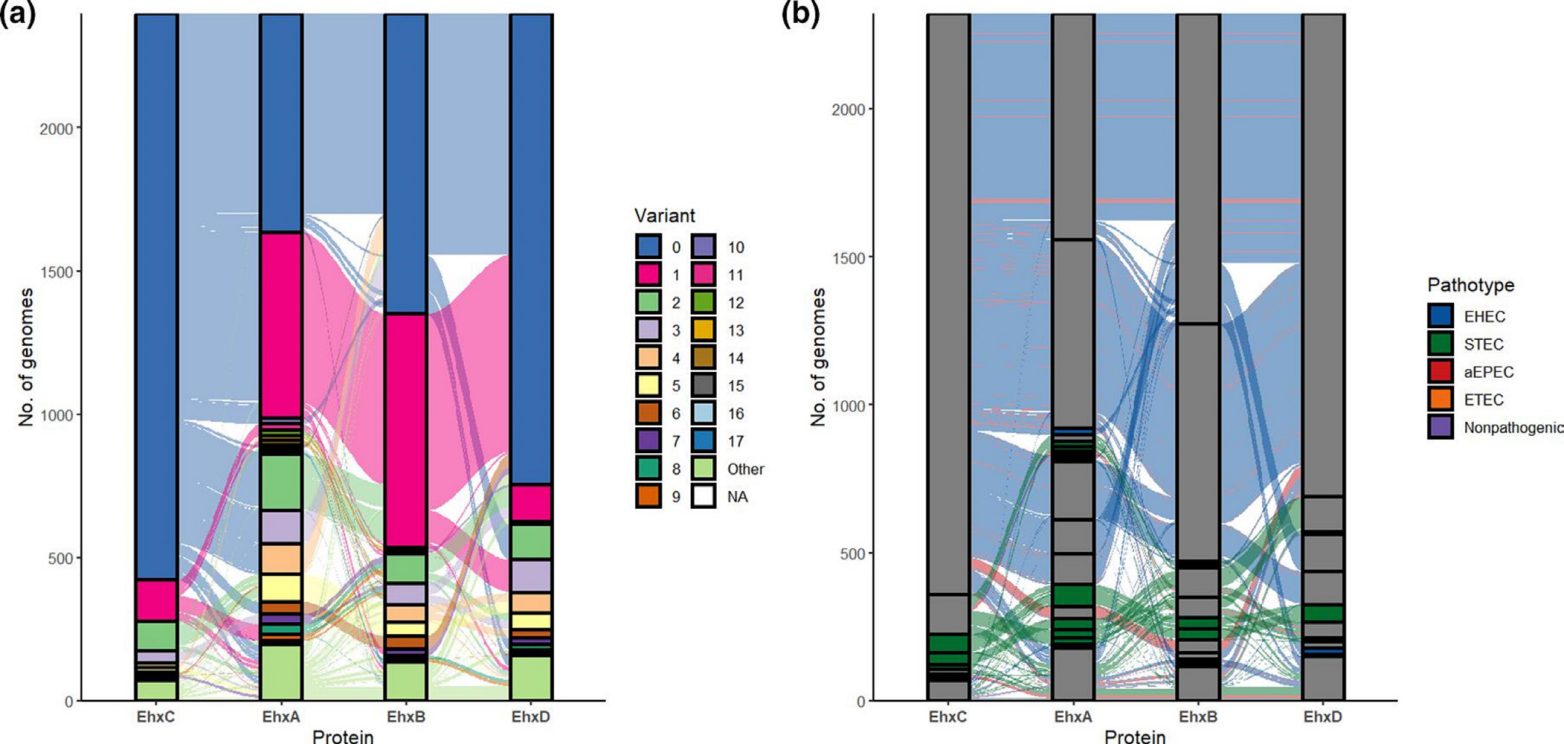
EhxCABD variants in enterohaemolysin-positive *E. coli *. Analysis of EhxCABD variant prevalence in enterohaemolysin-positive *E. coli* integrated with information about *E. coli* pathotype. The number of genomes is shown on the *y*-axis and the names of the proteins are shown on the *x*-axis. Colour streams and bars represent protein variants (**a**) or pathotypes (**b**) and are shown separately on the right side of each alluvial plot. In plot b) the bar has a grey colour if one variant is present in more than one pathotype. Variants present in fewer than 10 isolates were regrouped as ‘Other’ in plot (a) (the plot with all variants is shown in Fig. S8a). All genomes with undetermined pathotypes are not shown on plot (b) (the plot with all genomes is shown in Fig. S8c).

To assess the diversity and possible contribution of EhxCABD sequence variation to the enterohaemolysin function, the enterohaemolysin coding sequences were analysed. The highest number of variants was found for EhxA (106) followed by EhxD (84), EhxB (80) and EhxC (50) (Figs 5a and S8a). In some cases, potential loss of function mutations were detected. The highest numbers of these mutations were present in EhxD and EhxC, totalling 42 and 35, respectively, followed by only 9 strains with non-functional EhxA or EhxB. Overall, 97 of 2399 strains encoded potentially non-functional enterohaemolysin. When protein variants were contextualized with information about pathotypes from which the variants originated, it was apparent that most variants were present in more than one pathotype. It was interesting to note that EHEC and aEPEC possessed the same variants. This was also true for STEC, nonpathogenic *E. coli* and isolates with unidentified pathotypes as shown in Figs 5b, S8b and S8c. The variants found exclusively in one pathotype were characteristic of STEC.

**Fig. 6.**
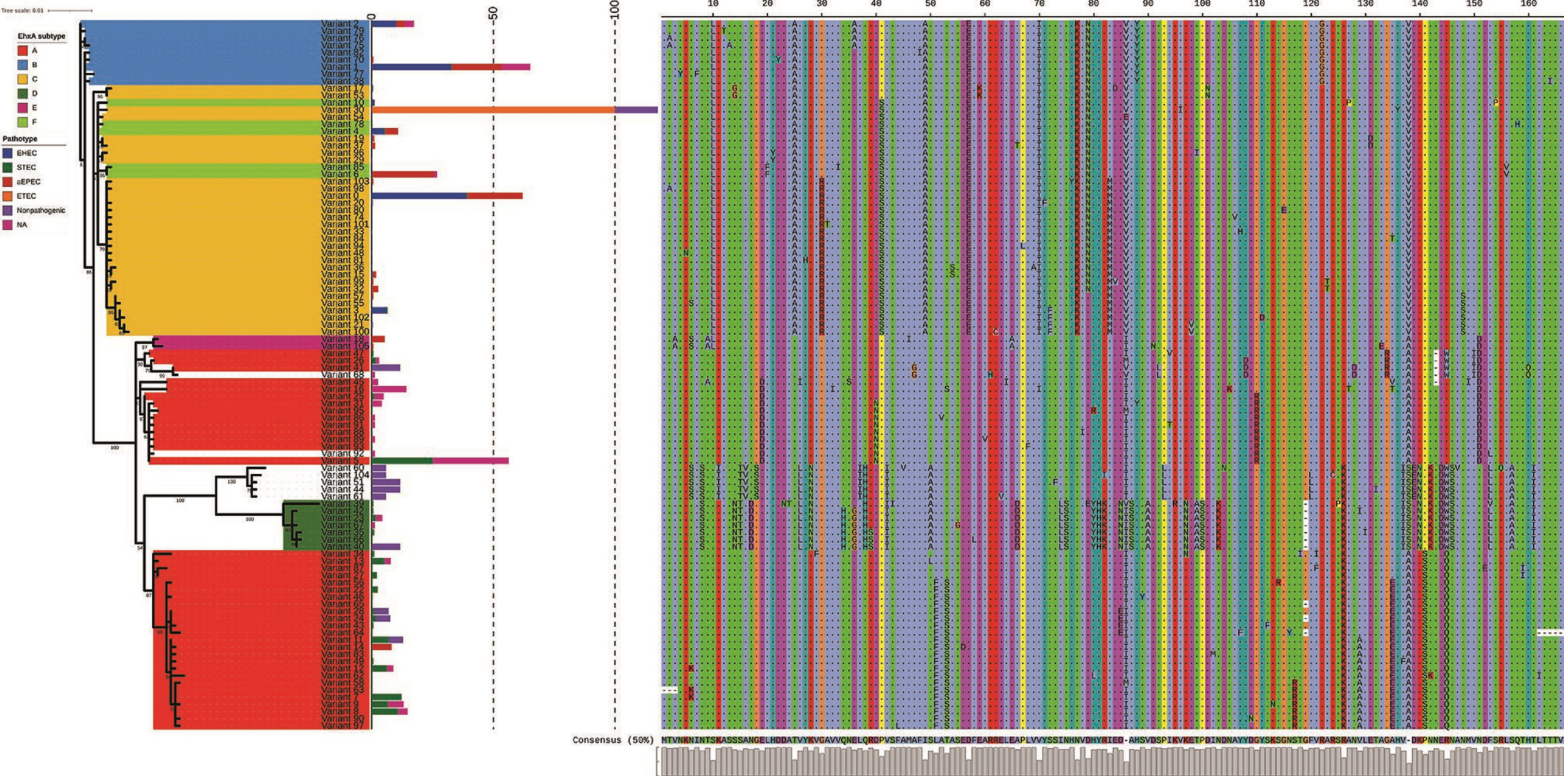
EhxA sequence variation. Phylogenetic analysis of 99 variants of EhxA found in 2399 enterohaemolysin-positive *E. coli* genomes. Relative prevalence of each variant in various pathotypes (as bars), amino acid-variable sites (166) and EhxA subtypes (colours of the clades) were annotated with use of iTOL. Amino acid-variable sites numbers on the plot do not reflect actual site numbers in the whole EhxA protein alignment. Colour keys for pathotypes shown on the barplot and EhxA subtypes marked on the phylogenetic tree are shown on the legends ‘Pathotype’ and ‘EhxA subtype’, respectively.

Since EhxA functions as a toxin and also had the highest number of variants among the proteins encoded by the *ehxCABD* operon, variants of this protein were investigated further. Of 106 EhxA variants, 99 were likely to be functional and had a total of 166 variable residues. Phylogenetic analysis revealed clustering of variants characteristic of EHEC and aEPEC together (Fig. 6). Moreover, isolates with no pathotype identified and STEC formed two clusters comprising multiple variants. Five variants from nonpathogenic *E. coli* clustered together as a separate group. EhxA subtyping analysis revealed that the subtypes align well with EhxA protein phylogeny and pathotypes (Figs 6 and S9). Additionally, two new subtypes were identified, one of which consisted of five EhxA variants found in nonpathogenic *E. coli *.

Our analyses identified a group of EhxA sequence variants associated with different pathotypes and nonpathogenic *E. coli *, indicating that the functionality of enterohaemolysin as a toxin may be altered in these bacteria.

## Discussion

Pathogenic *E. coli* are one of the main bacterial threats to human and animal health [2]. The high degree of genome plasticity leading to a large diversity of virulence factor repertoire among strains is an important factor that limits our ability to design successful preventions and treatment regimens for *E. coli* infections. The development of bacterial genomics in recent decades has allowed better characterization of pathogens, improvement of diagnostics and implementation of new preventive measures to limit disease spread [35]. In this study, we focused on the characterization of the haemolytic *E. coli* population that is associated with multiple *E. coli* pathotypes in human and animal infectious diseases.

Enterohaemolysin was mainly present in intestinal pathotypes such as EHEC, STEC and aEPEC. Similarly to previous studies, we confirmed that enterohaemolysin as a virulence factor could be an important genetic determinant used in routine diagnostics of these pathotypes [36]. STEC infections remain the third most commonly reported zoonosis in the European Union and underreporting remains one of the main issues for public health workers [37]. Therefore, the possible use of enterohaemolysin as an additional marker for diagnostic assays could offer valuable information about the investigated zoonotic agent.

Previous studies focused on enterohaemolysin typing as an additional tool to assess the pathogenic potential of STEC [33, 38]. Six EhxA subtypes based on *ehxA* gene TaqI digestion profile have been described so far, termed A to F [33]. Subtypes B, C and F have been associated with intimin-positive isolates from diseased patients [38, 39]. EhxA subgroups A and D were mainly associated with nonhuman samples and found in intimin-negative isolates. Since our dataset was generated by first searching for enterohaemolysin, we were able to identify a new subtype of EhxA, which was found exclusively in genomes with a VAF profile characteristic of nonpathogenic *E. coli *. The new EhxA subtype clustered with subtype D and was part of a larger clade together with sequences belonging to subtypes D and A (Fig. 6). Our analysis suggests that EhxA sequences from EHEC and aEPEC cluster separately from other EhxA sequences. To date, there have been no studies comparing the biological properties of distinct EhxA variants or subtypes reported. Our analysis provides the framework to address the question as to whether EhxA sequence variation contributes to the biological function of enterohaemolysin, altered *E. coli* toxicity towards the host, and outcome of infection associated with the pathotype definition. Future laboratory analyses should provide an answer to this question and might help with providing proof of the evolution of enterohaemolysin aiding the virulence of human and animal-associated pathogenic *E. coli *.

Alpha-haemolysins or enterohaemolysins are associated with the haemolytic phenotype in *E. coli* strains. We previously reported in a study focused on the characterization of alpha-haemolysin-encoding *E. coli* that the prevalence of these two VAFs does not overlap in the majority of pathotypes [13]. Alpha-haemolysin is mainly associated with UPEC, nonpathogenic *E. coli* or undetermined *E. coli* pathotype, whereas enterohaemolysin is mainly found in pathotypes such as STEC and EHEC. Interestingly, enterohaemolysin or alpha-haemolysin are found in aEPEC isolates. Our data suggest that although both haemolysins belong to the RTX toxin family, they might have a different biological impact on the host. Comparison of biological activity between alpha-haemolysin and enterohaemolysin for various host cells and in different environments could show altered functionality, thereby explaining the distribution of these toxins among *E. coli* pathotypes. Taken together, our analysis reveals a complex relationship between alpha-haemolysin- and enterohaemolysin-positive genomes and diverse *E. coli* pathotypes and provides a framework for understanding the potential role of these VAFs in pathogenesis.

## Supplementary Data

Supplementary material 1

Supplementary material 2

Supplementary material 3
