# Supplementary material for: Genomic characterization of enterohaemolysin-encoding haemolytic Escherichia coli of animal and human origin
Source: Microb Genom. 2023 Apr 27;9(4):mgen000999. doi: 10.1099/mgen.0.000999 (PMC10210957; doi:10.1099/mgen.0.000999)
Supplement: Supplementary material 1 [file mgen-9-999-s001.pdf]

Table S1. Pathotyping of *E. coli* from GenBank RefSeq database

Pathotypes were determined *in silico* using BLAST search for specific genes. Decisions for pathotyping are presented below with genes required to be present or absent to classify given genome to specific pathotype. Genomes that did not fit any of the criteria for pathotypes were termed 'NA' indicating an unknown pathotype. Accession numbers of all genes used for pathotyping are listed in the table footer.

Pathotype	Genes present	Genes absent
tEPEC	*eae*, *bfp*	*stx1a, stx1b, stx2a, stx2b*
aEPEC	*eae*	*bfp, stx1a, stx1b, stx2a, stx2b*
STEC	*stx1a, stx1b*<br>or<br>*stx2a, stx2b*	*eae, ial, ipaH, eltA, eltB, sta1, sta2, stb aggR, afaA, afaE-I, afaE-III, daaE, draE, sat, vat*
EHEC	*stx1a, stx1b, eae*<br>or<br>*stx2a, stx2b, eae*<br>or<br>*stx1a, stx1b, stx2a, stx2b,eae*	
EAEC	*aggR, aatA*	
ETEC	One of: *eltA, eltB, sta1, sta2*	*aggR*
EIEC	*ial* or *ipaH*	*stx1a, stx1b, stx2a, stx2b*
DAEC	*afaA, sat, afaE-I*<br>*or*<br>*afaA, sat, afaE-III*<br>*or*	

	afaA, sat, daaE<br>*or*<br>*afaA, sat, draE*	
UPEC	*fyuA, fimH, chuA, yfcV*<br>*or*<br>*fyuA, fimH, chuA vat*<br>*or*<br>*fyuA, fimH, yfcV, vat*	
NMEC	*sitA, vat, neuC, iucC, neuA*	
Nonpathogenic	None or only one of the following genes: *fyuA, iucC, neuC, sitA, yfcV*	*aap, aatA, aatC, aatP, afaA, afaB, afaC, afaD, afaE-I, afaE-III, aggR, bfpA, daaE, draE, eae, eltA, eltB, ial, ipaH, pet, sat, sta1, sta2, stb, stx1a, stx1b, stx2a, stx2b, vat*

Virulence gene GenBank Accession numbers: *aatA* AY351860.1:2552-3790, *afaA* FM955458.1:17-322, *afaE-I* FM955458.1:5290-5775, *afaE-III* X76688.1:8646-9128, *aggR* NC_019000.1:48472-49269, *bfpA* NC_010862.1:2646-3227, *chuA* NC_011750.1:c4160640-4158658, *daaE* M27725.1, *draE* AF329316.1:5805-6287, *eae* NC_002695.1:c4599262-4596458, *eltA* NC_013507.1:c22495-21719, *eltB* NC_013507.1:c21722-21348, *fimH* NC_011750.1:4996261-4997163, *fyuA* NC_011750.1:c1134590-1132569, *ial* AF348706.1:118694-119761, *ipaH* M32063.1, *iucC* NC_007675.1:100818-102560, *neuA* NC_011750.1:c3596071-3594815, *neuC* NC_011750.1:c3594818-3593643, *sat* NC_004431.1:c3460261-3456362, *sitA* NC_017659.1:c126422-125481, *sta1* NZ_CP024249.1:c123053-122835, *sta2* NC_017724.1:c35945-35727, *stb*

NC_018998.1:c5269-5054, *stx1a* NC_002695.1:c2925716-2924769, *stx1b* NC_002695.1:c2924759-2924490, *stx2a* NC_002695.1:1266965-1267924, *stx2b* NC_002695.1:1267936-1268205, *vat* NC_008563.1:c313072-308942, *yfcV* NC_011750.1:c2577809-2577243.

Table S2. List of overlapping regions used to determine *ehxCABD* cluster genome placement

Name	GenBank Accession Number	Genes before
Plasmid_1 pMB9_1	CP040108.1:14095-16094	hypotethical protein (pseudo), IS3 family transposase (pseudo), lipoprotein, PulS/OutS family
Plasmid_1 p493-89-1	CP038413.1:c105876-103877	hypothetical protein (pseudo), IS3 family transposase (pseudo), gamma-D-glutamyl-meso-diaminopimelate amidase (pseudo), IS30-like element IS30 family transposase
Plasmid_1 p1RM9088	CP042296.1:c9285-7286	IS3 family transposase (pseudo), plasmid pRiA4b ORF-3 family protein, hypothetical protein (pseudo), hypothetical protein
Plasmid_1 unnamed1	CP027326.1:14676-16675	
Plasmid_1 pSTEC17_183-c	CP075655.1:13347-15346	IS66 family transposase (pseudo), IS21-like element IS21 family helper ATPase IstB, IS21-like element IS21 family transposase
Plasmid_1 pO111-110512_2	AP019763.1:76471-78470	DNA-binding protein, transposase, hypothetical protein, transposase
Plasmid_1 pO145_122715_1	AP019709.1:84875-86874	/pseudogene=unknown; IS629 transposase OrfB, N-terminal part, IS629 transposase OrfA
Plasmid_1 pO121_51104	AP024472.1:79950-81949	hypothetical protein, transposase
Plasmid_1 unnamed16	CP031909.1:c15798-13799	hypothetical protein (pseudo), IS3 family transposase, conjugal transfer protein TraH (pseudo)
Plasmid_1 unnamed9	CP031920.1:75099-77098	hypothetical protein (pseudo), IS3 family transposase, hypothetical protein
Plasmid_1 pRM9975-2	CP028434.1:c65269-63270	hypothetical protein (pseudo), IS3 family transposase (pseudo), transposase
Plasmid_1 pS19_141-a	CP075641.1:c11757-9758	OmpA family protein, YadA-like family protein
Plasmid_2 pMB9_1	CP040108.1:23222-25221	transcriptional regulator, MarR family transcriptional regulator, hypothetical protein, serine/threonine protein kinase, site-specific integrase
Plasmid_2 p493-89-1	CP038413.1:c96773-94773	transcriptional regulator, MarR family transcriptional regulator, hypothetical protein, serine/threonine protein kinase, site-specific integrase

Plasmid_2 p1RM9088	CP042296.1:c162-1,c167256-165419	IS3 family transposase (pseudo), transposase (pseudo)
Plasmid_2 unnamed1	CP027326.1:c7549-5550	
Plasmid_2 pSTEC17_183-c	CP075655.1:22474-24473	site-specific integrase (pseudo), CopG family transcriptional regulator, AAA family ATPase
Plasmid_2 pO111-110512_2	AP019763.1:7128-9127	integrase, transposase
Plasmid_2 pO145_122715_1	AP019709.1:7128-9127	colicin-M, colicin M resistance protein, resolvase ResA
Plasmid_2 pO121_51104	AP024472.1:7128-9127	hypothetical protein, colicin M resistance protein, hypothetical protein
Plasmid_2 unnamed16	CP031909.1:c6695-4696	lipid II-degrading bacteriocin (pseudo), colicin M immunity protein, IS3 family transposase
Plasmid_2 unnamed9	CP031920.1:c67995-65996	transcriptional regulator (pseudo), lipid II-degrading bacteriocin (pseudo), colicin M immunity protein
Plasmid_2 pRM9975-2	CP028434.1:c56166-54167	IS3 family transposase (pseudo), IS66-like element ISEc8 family transposase
Plasmid_2 pS19_141-a	CP075641.1:c2630-631	MarR family transcriptional regulator, serine/threonine protein kinase, transposase family protein (pseudo)

Table S3. Genes used for analysis of adhesin, toxin and iron acquisition gene frequency

Single accession numbers indicate sequences of whole operons, whereas multiple accessions correspond to specific genes or structural subunits of operons, with the exception of *aggR*, where three variants specific for AA/I, AA/II and AA/III fimbriae were used.

System name	Genes	Genbank Accession numbers
Type_1_fimbriae	*fimH, fimB, fimE, fimA, fimF, fimG, fimC, fimD, fimI*	NC_000913.3:4540957-4549710
Yad_fimbriae	*yadN, yadV, yadM, yadL, yadK, yadC, htrE*	NC_000913.3:156883-149715
Yeh_fimbriae	*yehA, yehB, yehC, yehD, yehE*	NC_000913.3:c2192796-2187380
Ybg_Fimbriae	*ybgD, ybgO, ybgP, ybgQ*	NC_000913.3:c752795-747921
987P_Fimbriae	*fasA, fasB, fasC, fasD, fasE, fasF, fasG, fasH*	NZ_UART01000006.1:c10922-3662
Yfc_Fimbriae	*yfcO, yfcP, yfcQ, yfcR, yfcS, yfcT, yfcU, yfcV*	CP000948.1:c2545433-2539015
Yra_Fimbriae	*yraH, yraI, yraJ, yraK*	NC_000913.3:3287426-3292432
Ygi_Fimbriae	*ygiL, yqiG, yqiH, yqiI*	NC_000913.3:3185414-3191696
Type_3_Fimbriae	*mrkA, mrkB, mrkC, mrkD, mrkF*	NC_010378.1:c8694-3156

P_Fimbriae	*papA, papB, papC, papD, papE, papF, papG, papH, papI, papJ, papK*	NZ_CP033092.2:c853171-852950; NZ_CP033092.2:853606-862040
K99_Fimbriae	*fanA, fanB, fanC, fanD, fanE, fanF, fanG, fanH*	NZ_CP026930.1:c17264-10780
K88_Fimbriae	*faeB, faeA, faeC, faeD, faeE, faeF, faeG, faeH, faeI, faeJ*	NC_017639.1:2949-12348
Curli_Fimbriae	*csgB, csgC, csgA, csgD, csgE, csgF, csgG*	NC_000913.3:c1105293-1103951; NC_000913.3:c1103196-1100851
S_Fimbriae	*sfaC, sfaB, sfaA, sfaD, sfaE, sfaF, sfaG, sfaS, sfaH, sfaX, sfaY*	CP001671.1:c1161914-1161693; CP001671.1:1162333-1164922; NC_017631.1:1219598-1222736; NC_017631.1:1223040-1224478
Stg_Fimbriae	*stgA, stgB, stgC, stgD*	NC_022364.1:c4055730-4050748
Sfm_Fimbriae	*sfmA, sfmC, sfmD, sfmH, sfmF*	NC_000913.3:558212-563845
Hda_Fimbriae	*hdaA, hdaB, hdaC, hdaD*	NC_008460.1:15366-19732
Tsh	*tsh*	NC_007675.1:c170806-166673
Autotransporters	*aatA, aatB, ehaG, ehaA, ehaB, espP, sab, cah, upaG, sadA, ypjA, yfaL, yeeJ, ycgV, flu, aidA, tibA*	NC_008460.1:27389-28627; NC_008460.1:28524-29345; WMHS01000001.1:c110208-105358; NZ_JABADO010000042.1:c48223-44240; NZ_VYQD01000015.1:11668-14670; NC_002128.1:80757-84659; NZ_BFXE01000019.1:8148-12443; CP001368.1:1388023-1390872; CP051749.1:1870738-1876203; UWWB01000001.1:2273779-2274585; NC_000913.3:c2782726-2778146; NC_000913.3:c2344169-2340417; NC_000913.3:2044938-2052014; NC_000913.3:c1255952-1253085; NC_000913.3:2071539-2074658; NC_002695.1:1444150-1446999; NC_017633.1:2299853-2302822
Intimin	*eae, tir*	NC_002655.2:c4668245-4665441; NC_002655.2:c4670589-4668913
Long_polar_fimbriae	*lpfA, lpfB, lpfC, lpfC', lpfD, lpfE, lpfA2, lpfB2, lpfC2, lpfD2, lpfD'2*	AE005174.2:c4525276-4519782
Long_polar_fimbriae_2	*lpfA2, lpfB2, lpfC2, lpfD2, lpfD'2*	AE005174.2:c4773565-4767426
Ecp_fimbriae	*ecpA, ecpB, ecpC, ecpD, ecpE, ecpR*	NC_000913.3:c311336-304495
F9_fimbriae	*ydeT, ydeR, ydeS, ydeQ*	NC_000913.3:c1590001-1586820
Ycb_fimbriae	*ycbQ, ycbR, ycbS, ycbT, ycbU, ycbV, ycbF*	NC_000913.3:997868-1004657
Sfp_fimbriae	*sfpA, sfpH, sfpC, sfpD, sfpJ, sfpF, sfpG*	NG_036703.1:829-7666

Auf_fimbriae	*aufA, aufB, aufC, aufD, aufE, aufF, aufG*	NC_011742.1:c3773337-3766183
Bfp_fimbriae	*bfpA, bfpB, bfpC, bfpD, bfpE, bfpF, bfpG, bfpH, bfpI, bfpJ, bfpK, bfpL, bfpP, bfpU*	NC_010862.1:2646-13903
Paa_adhesin	*paa*	NC_010862.1:6523-7017
ToxB	*toxB*	NC_002128.1:55981-65490
Efa1/LifA_adhesin	*efa1/lifA*	NC_013364.1:c5097333-5087662
Saa_adhesin	*saa*	NC_007365.1:143552-145156
OmpA	*ompA*	NC_000913.3:c1020053-1019013
EtpA_adhesin	*etpA*	NC_014232.1:79223-83851
AA/II_fimbriae	*aafA, aafB, aafC, aafD*	NC_017627.1:c25581-22554; NC_017627.1:38499-40134
AA/I_fimbriae	*aggA, aggB, aggC, aggD*	NC_022743.1:c65609-61253
AA/I_II_III_fimbriae	*aggR*	NC_022743.1:c50895-50098; NC_017627.1:c41877-41080; NC_011752.1:c48186-47389
AA/III_fimbriae	*agg3A, agg3B, agg3C, agg3D*	NC_011752.1:c58326-53842
PCF071_fimbriae	*cosB, cosA, cosC, cosD*	AY513487.1:994-6061
Pix_fimbriae	*pixB, pixA, pixH, pixC, pixD, pixJ, pixF, pixG*	CP051727.1:4124656-4131784
FdeC_adhesin	*fdeC*	NZ_CP033092.2:c3690246-3685996
T3SS	*espA, espB, espD, escF, escC, escI, escJ, escD, escR, escS, escT, escV, escO, escU, sepL, escK, escQ, cesT, cesF, escN, escL, orf3, orf2, ler*	NC_011601.1:c4108763-4104932; NC_002695.1:c4590593-4590372; NZ_JAAKCS010000059.1:7325-11297; NC_002695.1:4611376-4612914; NC_002695.1:4609912-4610915; NC_002695.1:c4609045-4605314; NZ_BFBN01000057.1:c3122-2232; NC_002695.1:4594974-4596194; NC_013364.1:3734354-3734716; NC_002695.1:c4599792-4599322
T6SS	*hcp, vgrG, clpV*	NC_000913.3:c913814-912162; NZ_CP033092.2:c1049102-1046622; NC_013008.1:c253887-251110
CFA/I_fimbriae	*cfaA, cfaB, cfaC, cfaE, cfaD*	M55661.1:848-6822
Dr_fimbriae	*draA, draB, draC, draD, draP, draE*	CP034404.1:c3248821-3243044
CS2_fimbriae	*cotB, cotA, cotC, cotD*	Z47800.1:499-5545
CS1_fimbriae	*cooB, cooA, cooC, cooD*	FN822748.1:6260-11330
CS3_fimbriae	*cstA, cstB, cstC, cstD, cstE, cstH*	X16944.1:378-4659
CS4_fimbriae	*csaA, csaB, csaC, csaE*	NZ_CP025855.1:24484-29482
CS5_fimbriae	*csfA, csfB, csfC, csfE, csfF, csfD*	AX741419.1:1427-7644
CS6_adhesins	*cssA, cssB, cssC, cssD*	U04844.1:649-4801

CS12_fimbriae	*cswA, cswB, cswC, cswD, cswE, cswF, cswG*	AY009096.1:3505-10129
CS14_fimbriae	*csuB, csuA1, csuA2, csuC, csuD*	AY283611.1:542-6105
CS17_fimbriae	*csbB, csbA, csbC, csbD*	AY515609.1:509-5555
CS18_fimbriae	*fotA, fotB, fotC, fotD, fotE, fotF, fotG*	AF335469.1:2044-8670
CS19_fimbriae	*csdB, csdA, csdC, csdD*	AY288101.1:264-5304
CS13_fimbriae	*cshA, cshB, cshC, cshD, cshE, cshF, cshG*	X71971.1:191-7331
CS23_adhesins	*aalR, aalA, aalB, aalC, aalD, aalE, aalF, aalG, aalH*	JQ434477.1:555-8821
CS7_fimbriae	*csvA*	AY009095.1:144-755
CS20_fimbriae	*csnA*	AF438155.1:1-588
CS22_fimbriae	*cseA*	AF145205.1:66-566
CS8_fimbriae	*cofR, cofS, cofT, cofA, cofB, cofC, cofD, cofE, cofF, cofG, cofH, cofI, cofJ, cofP*	AB049751.1:635-13378
CS21_fimbriae	*lngX1, lngR, lngS, lngT, lngX2, lngA, lngB, lngC, lngD, lngE, lngF, lngG, lngH, lngI, lngJ, lngP*	EF595770.1:22-13886
REPEC_fimbriae	*ralC, ralD, ralE, ralF, ralG, ralH, ralI*	U84144.1:33-7005
F107_fimbriae	*fedA, fedB, fedC, fedE, fedF*	MG904998.1:60450-65874
Afa-VIII_adhesins	*afaA-VIII, afaB-VIII, afaC-VIII, afaD-VIII, afaE-VIII*	AP017620.1:4776127-4781555
Afa-III_adhesins	*afaF-III, afaA-III, afaB-III, afaC-III, afaD-III, afaE-III*	NZ_UGBE01000001.1:c91062-85592; NZ_UGBE01000001.1:c84838-84356
Afa-I_adhesins	*afaA, afaB, afaC, afaD, afaE*	QYMO01000014.1:6963-11437; QYMO01000014.1:12202-12687
F1845_fimbriae	*daaF, daaA, daaB, daaC, daaD, daaP, daaE*	NZ_SQMD01000127.1:c11208-4516
Lda_adhesins	*ldaC, ldaD, ldaE, ldaF, ldaG, ldaH, ldaI*	AY858803.1:3662-10965
F1C_fimbriae	*focA, focI, focC, focD, focF, focG, focH*	CP037449.1:3994150-4000764
AF/R1_fimbriae	*afrS, afrR, afrA, afrB, afrC, afrD, afrE*	AF050217.1:c1615-98; AF050217.1:1906-7676
F17a_fimbriae	*f17a-A, f17a-D, f17a-C, f17a-G*	CP026932.1:c970878-965955

F17b_fimbriae	*f17b-A, f17b-D, f17b-C, f17b-G*	CP001162.1:9749-14672
F17d_fimbriae	*f17d-A, f17d-C, f17d-D, f17d-G*	L77091.1:475-5397
chuASTUWVXY	*chuA, chuT, chuU, chuV, chuS, chuW, chuX, chuY*	NZ_CP033092.2:223304-232256
sitABCD	*sitA, sitB, sitC, sitD*	NZ_CP033091.2:124903-128352
Silent hemolysin	*hlyE*	NC_000913.3:c1230394-1229483
iroBCDEN	*iroB, iroC, iroD, iroE, iroN*	NZ_CP033091.2:26642-36152
fecABCDEIR	*fecA, fecB, fecC, fecD, fecE, fecI, fecR*	NC_000913.3:4510690-4518235
iucABCDiutA	*iucA, iucB, iucC, iucD, iutA, tonB, fhuB, fhuC, fhuD, fhuE, exbB, exbD, fur, tonB*	NZ_CP033091.2:113600-121571; NC_000913.3:1311089-1311808; NC_000913.3:167484-173444; NC_000913.3:3150818-3151984; NC_000913.3:c710646-710200; NC_000913.3:1311089-1311808
ybtAEPSTQUXirp12fyuA	*ybtA, ybtE, ybtP, ybtT, ybtS, ybtX, ybtQ, ybtU, irp1, irp2, fyuA*	NZ_CP033092.2:2026886-2055717
bfd	*bfd*	NC_000913.3:c3466991-3466797
Other genes with potential functions in iron acquisition	*ybiJ, ybiI, ybiX, fiu, ybiM, yncD, yncE, pqqL, yddB, yddA, ydiE, yqjH*	NC_000913.3:837665-842056; NC_000913.3:1520963-1524368; NC_000913.3:1572407-1579342; NC_000913.3:1789613-1789804; NC_000913.3:c3216491-3215727
Heme transport system (Hma)	*hma, tonB*	NC_009133.1:962-1237; NC_000913.3:1311089-1311808
Other hemolysins and hemoglobin proteases	*hbp, espC, pic, shlA, shlB*	NZ_CP022731.1:54397-58530; NC_017644.1:4454260-4458024; NC_017626.1:c4928958-4924840; NC_002655.2:1437607-1439226; NZ_CP033092.2:c4336856-4336488
Enterobactin/Enterochelin	*fepA, fepB, fepC, fepD, fepE, fepF, fepG, tolC, tonB, exbB, exbD, fes, entS, entA, entB, entC, entD, entE, entF, entH*	NC_000913.3:609459-613939; NC_000913.3:614157-629713; NC_000913.3:3178115-3179596; NC_000913.3:1311089-1311808; NC_000913.3:3150818-3151984
TonB energy transduction system	*tonB, exbB, exbD*	NC_000913.3:1311089-1311808; NC_000913.3:3150818-3151984
Efe system	*efeU, efeO, efeB*	NC_000913.3:1081356-1084647
Heme biosynthetic and assimilation pathway	*hemA, hemL, hemM, hemC, hemD, hemH, hemY, hemB*	NC_000913.3:1262877-1264970; NC_000913.3:c174882-173602; NC_000913.3:3986686-3987882; NC_000913.3:3989088-3990766; NC_000913.3:498055-499017; NC_000913.3:c389727:388753
Alpha hemolysin	*hlyA, hlyB, hlyC, hlyD, tolC*	CP000243.1:4821373-4828752; NC_000913.3:3178115-3179596
Enterohemolysin	*ehxA, ehxB, ehxC, ehxD, tolC*	X86087.1:74-7191; NC_000913.3:3178115-3179596
Feo system	*feoA, feoB, feoC, fur, fnr*	NC_000913.3:3540163-3542964; NC_000913.3:c710646-710200; NC_000913.3:c1399526-1398774
Fur - transcriptional regulator of iron transport	*fur*	NC_000913.3:c710646-710200
RyhB regulator	*ryhB*	NC_000913.3:c3581016-3580927

Siderophore receptors	*cir, fecA, fepA, fhuA, fhuE, fiu, iroN, iutA, chuA, fyuA*	NC_000913.3:c2246769-2244778; NC_000913.3:c4516677-4514353; NC_000913.3:c612494-610254; NC_000913.3:167484-169727; NC_000913.3:1159362-1161551; NC_000913.3:839249-841531; NZ_CP033091.2:c36152-33975; NZ_CP033091.2:c115801-113600; NZ_CP033092.2:229197-231179; NZ_CP033092.2:c2028907-2026886
Iron storage proteins	*bfd, bfr, ftnA, ftnB, dps*	NC_000913.3:c3466249-3466991; NC_000913.3:1986925-1989213; NC_000913.3:c848911-848408
Permeases involved in iron transport	*mntH, zupT, yiiP*	NC_000913.3:c2512706-2511468; NC_000913.3:3182550-3183323; NC_000913.3:4106469-4107371
Genes involved in iron uptake, identified in E. coli genome and play a main role in another species of bacteria or their main role is different than iron uptake	*afuC, afuB, rcnR, rcnA, yaaA*	NC_000913.3:277756-279162; NC_000913.3:2185524-2186741; NC_000913.3:c6459-5683

Table S4. Statistical analysis for comparison of inferred functional group frequency in alpha-hemolysin and enterohemolysin-bearing plasmids

Comparison between numbers of genes belonging to the same functional (according to the eggnog-mapper analysis) was performed with the Kruskal-Wallis test.

COG group name	p value (Benjamini-Hochberg corrected)
-	8.62354E-08
B	8.9588E-08
C	3.21311E-10
D	3.55883E-06
E	0.458577687
F	9.0234E-16
G	0.315204052
H	5.85935E-19
I	1.02202E-36
J	3.48152E-17
K	0.115012543
L	0.053391461
M	0.837208181
N	0.038594689
O	2.03875E-06
P	0.115012543
Q	9.99398E-11
S	2.62915E-08
T	1.60595E-24
U	1.97527E-05

V	6.29723E-06
W	0.550032254

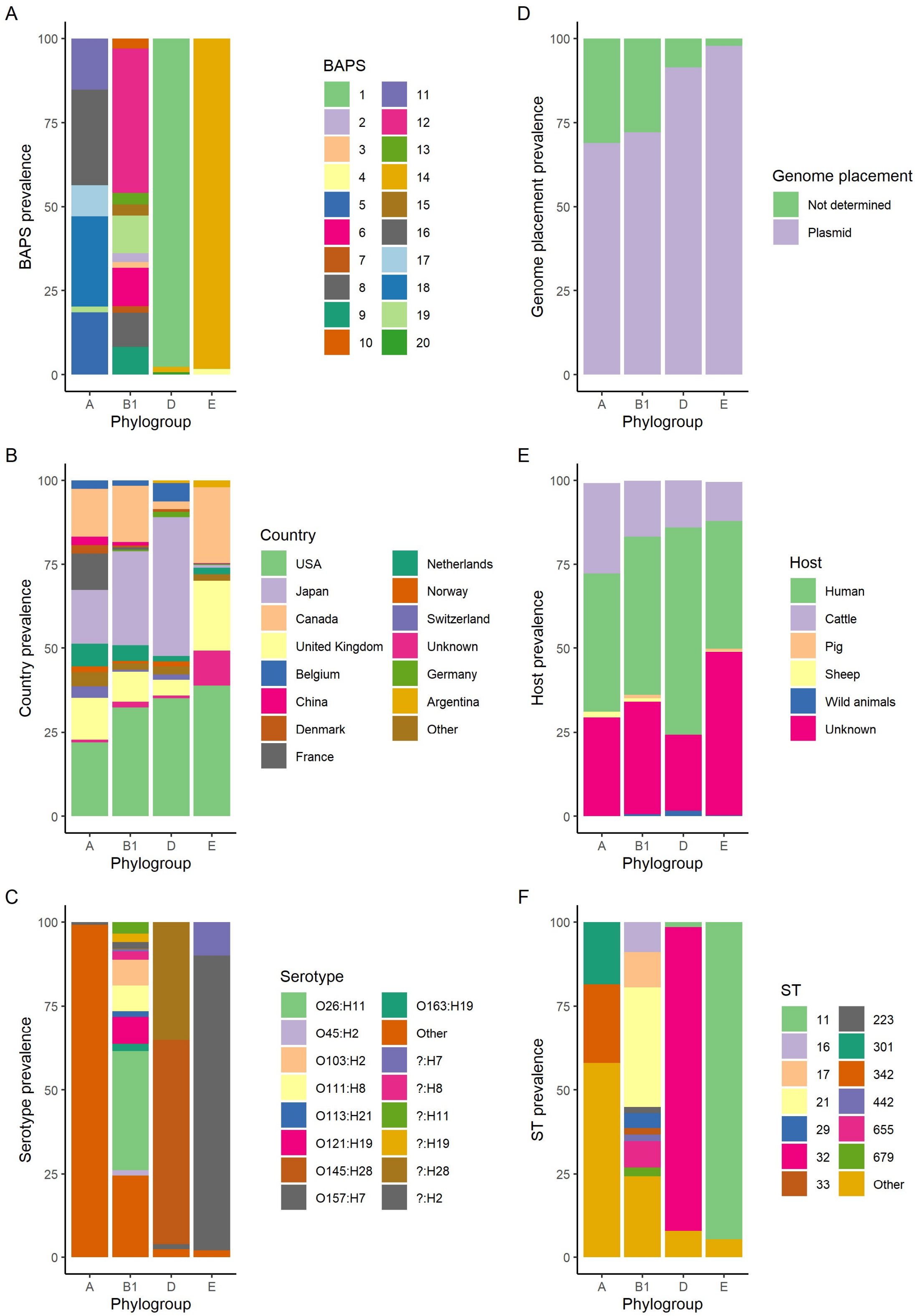

Figure S1. Analysis of enterohemolysin-positive *E. coli* genomes

Prevalence of BAPS groups, Countries, STs, Genome placement, Serotypes and Host in enterohemolysin-positive *E. coli* belonging to different phylogroups. Names of Phylogroup (A, B1, D, E) are shown on the x-axis. Prevalence of BAPS groups (A), Countries, (B) Serotypes (C), Genome placement (D), Host (E) and STs (F) are listed on the y-axis. Various colours represent different BAPS groups (A), Countries, (B) Serotypes (C), Genome placement (D), Host (E) and STs (F) and are described on the legend for each plot separately. Group "Unknown" contains prevalence of unknown variables presented and group "Other" contains prevalence of variants present in less than 20 isolates. Phylogroups C and G are not included in this analysis due to the low number of isolates belonging to these groups.

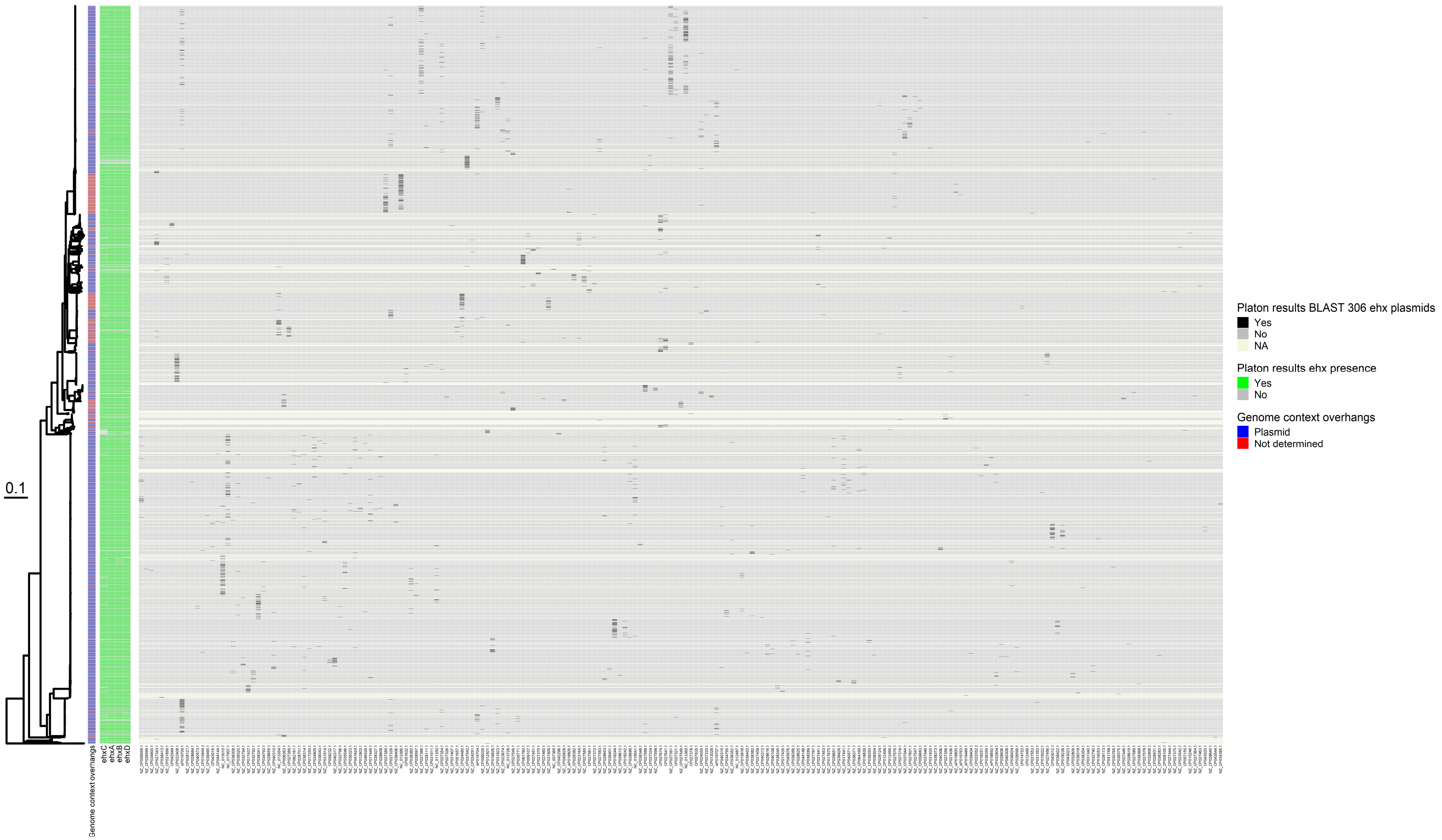

Figure S2. Determination of the genomic context of *ehxCABD*

Results of genomic context analysis for enterohemolysin-coding genes were annotated on the midpoint-rooted tree with the use of ggtree. Results obtained from comparison of 5'-upstream of *ehxC* and 3'-downstream of *ehxD* sequences to reference sequences are presented on the first heatmap from the tree (Legend title: Genome context overhangs, plasmid localization- blue colour). Results from Platon plasmid analysis are shown on the second heatmap, where presence of *ehxCABD* in plasmid contigs is marked in green (Legend title: Platon results ehx presence). Results of analysis of contigs identified as plasmids/part of a plasmid with Platon and BLAST search against 306 plasmids encoding *ehcCABD* identified in PLSDB are shown on the third heatmap from the tree (Legend title: Platon results BLAST 306 ehx plasmids), where presence of nearly identical sequence of plasmid contig in one of enterohemolysin-encoding plasmid (on the x-axis) was marked in black.

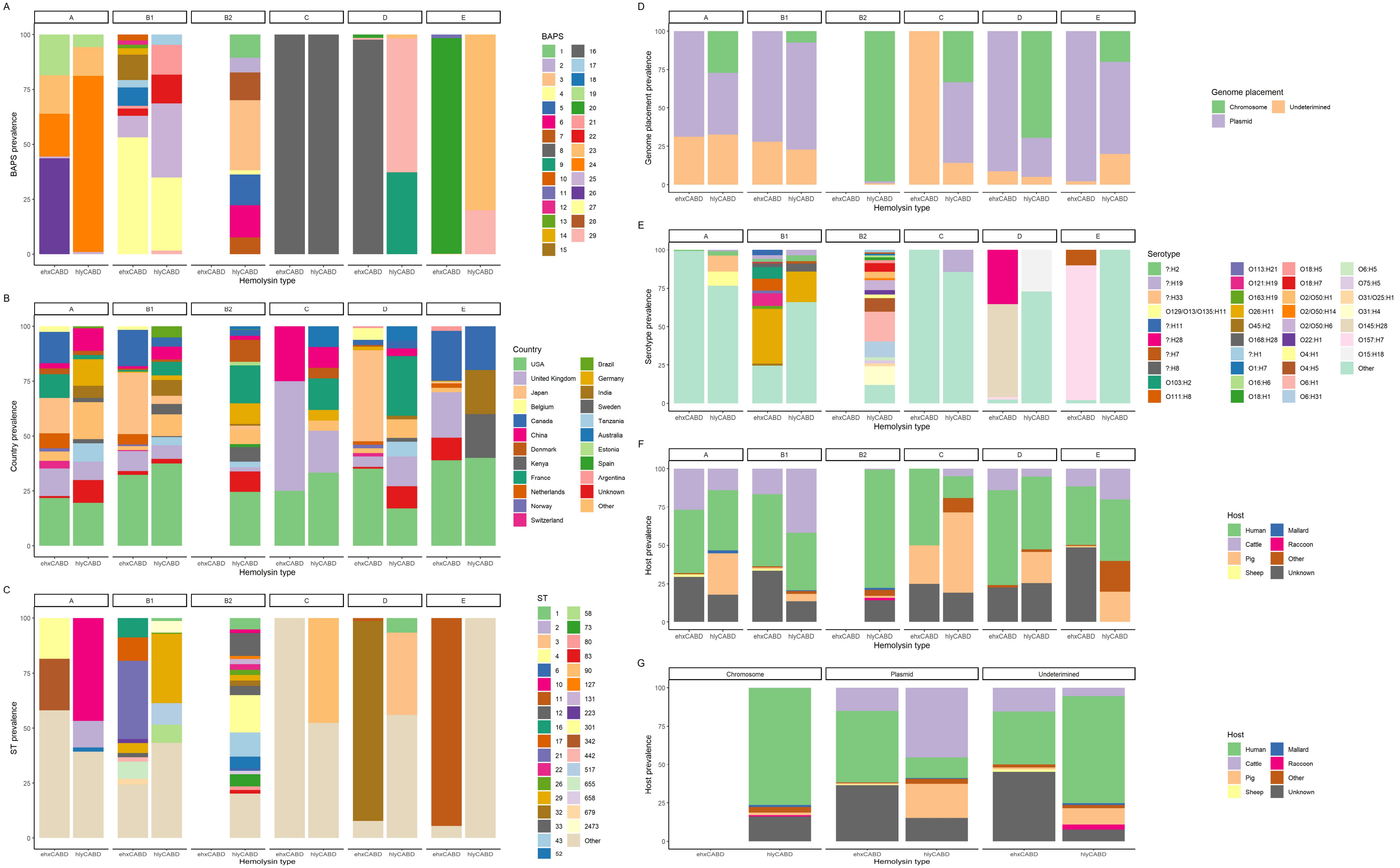

A
B
C
D
E
F
G
BAPS prevalence
Country prevalence
ST prevalence
Genome placement prevalence
Serotype prevalence
Host prevalence
Hemolysin type
ehxCABD
hlyCABD
A
B1
B2
C
D
E
Chromosome
Plasmid
Undetermined
BAPS
Country
USA
United Kingdom
Japan
Belgium
Canada
China
Denmark
Kenya
France
Netherlands
Norway
Switzerland
Brazil
Germany
India
Sweden
Tanzania
Australia
Estonia
Spain
Argentina
Unknown
Other
ST
Genome placement
Chromosome
Plasmid
Undeterimined
Serotype
?:H2
?:H19
?:H33
O129/O13/O135:H11
?:H11
?:H28
?:H7
?:H8
O103:H2
O111:H8
O113:H21
O121:H19
O163:H19
O26:H11
O45:H2
O168:H28
?:H1
O1:H7
O16:H6
O18:H1
O18:H5
O18:H7
O2/O50:H1
O2/O50:H14
O2/O50:H6
O22:H1
O4:H1
O4:H5
O6:H1
O6:H31
O6:H5
O75:H5
O31/O25:H1
O31:H4
O145:H28
O157:H7
O15:H18
Other
Host
Human
Cattle
Pig
Sheep
Mallard
Raccoon
Other
Unknown

Figure S3. Comparison of enterohemolytic and alpha-hemolytic *E. coli* genomes

Prevalence of BAPS groups, countries of isolation, STs, genome context, serotypes and host in enterohemolysin- and alpha-hemolysin-positive *E. coli* belonging to different phylogroups. Names of phylogroup (A, B1, B2, C, D, E) are shown on the top x-axis. *E. coli* hemolysin types (ehxCABD - enterohemolysin, hlyCABD - alpha-hemolysin) are shown on the bottom x-axis. Prevalence of BAPS groups (A), countries (B), STs (C), genome context (D), serotypes (E) and hosts (F, G) are listed on the y-axis. Various colours represent different BAPS groups (A), countries (B), STs (C), genome context (D), serotypes (E) and hosts (F, G) and are described on the legend for each plot separately. Group "Unknown" contains prevalence of unknown variables and group "Other" contains prevalence of variants present in less than 20 isolates. Phylogroups G and F are not included in this analysis due to the low amount of isolates belonging to these groups.

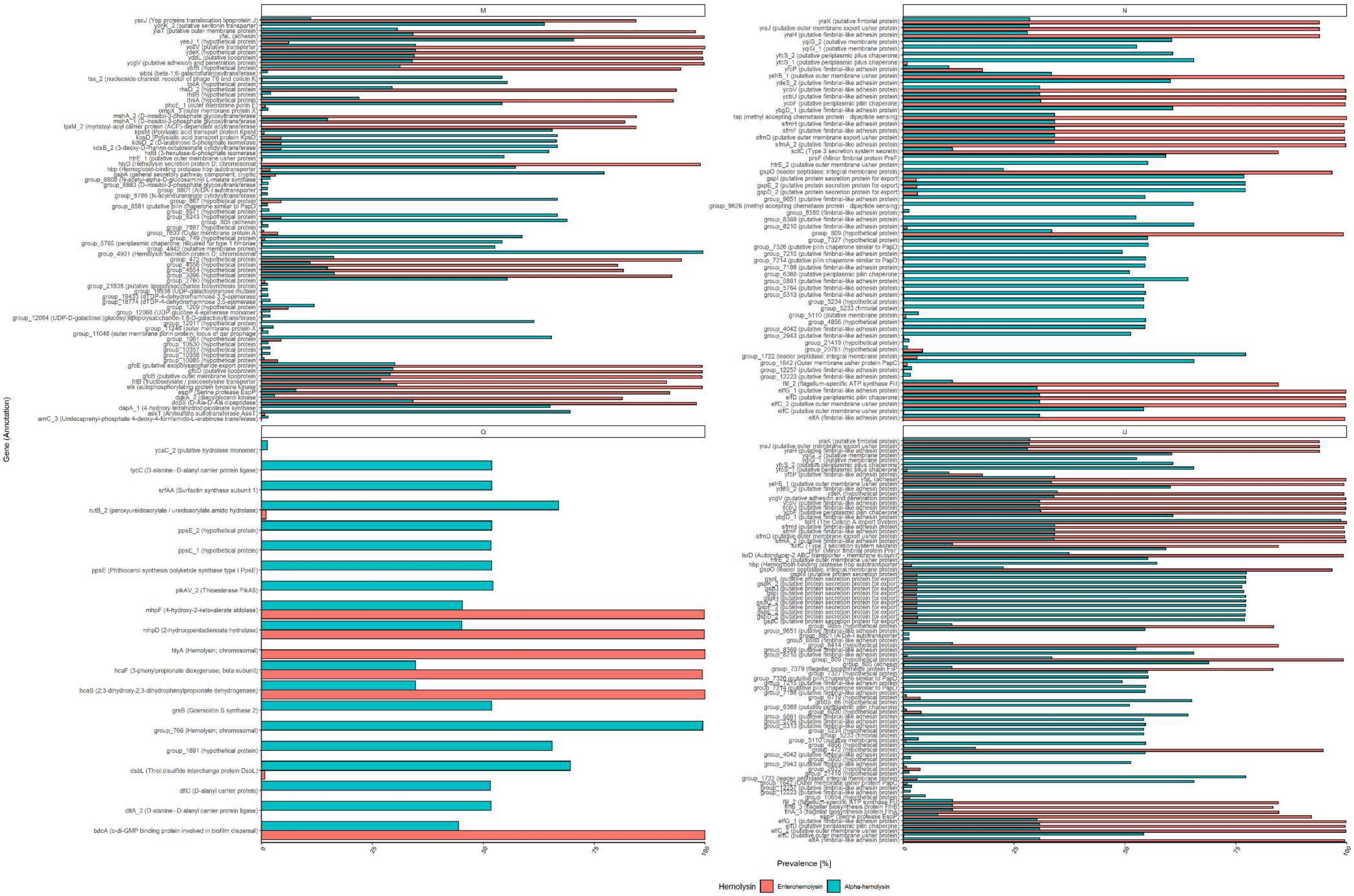
M
N
Q
U
ycaC_2 (putative hydrolase monomer)
tycC (D-alanine--D-alanyl carrier protein ligase)
srfAA (Surfactin synthase subunit 1)
rutB_2 (peroxyureidoacrylate / ureidoacrylate amido hydrolase)
ppsE_2 (hypothetical protein)
ppsE_1 (hypothetical protein)
ppsE (Phthiocerol synthesis polyketide synthase type I PpsE)
pikAV_2 (Thioesterase PikA5)
mhpF (4-hydroxy-2-ketovalerate aldolase)
mhpD (2-hydroxypentadienoate hydratase)
hlyA (Hemolysin; chromosomal)
hcaF (3-phenylpropionate dioxygenase; beta subunit)
hcaB (2;3-dihydroxy-2;3-dihydrophenylpropionate dehydrogenase)
grsB (Gramicidin S synthase 2)
group_768 (Hemolysin; chromosomal)
group_1891 (hypothetical protein)
dsbL (Thiol:disulfide interchange protein DsbL)
dltC (D-alanyl carrier protein)
dltA_3 (D-alanine--D-alanyl carrier protein ligase)
bdcA (c-di-GMP binding protein involved in biofilm dispersal)
Gene (Annotation)
0
25
50
75
100
Prevalence [%]
Hemolysin
Enterohemolysin
Alpha-hemolysin

Figure S4. Prevalence of genes belonging to M, N, Q and U cluster of orthologous groups (COG) in alpha-hemolysin- and enterohemolysin-positive *E. coli*

Barplots with gene prevalence from COG functional groups M, N, Q and U. Names of group (M, N, Q, U) are shown on the top x-axis. Prevalence of *E. coli* hemolysin types (ehxCABD- Enterohemolysin, hlyCABD- Alpha-hemolysin) is shown on the bottom x-axis. Gene names and annotations (in brackets) are listed on the y-axis. Bar colours represent different hemolysins and are described on the legend below the plots.

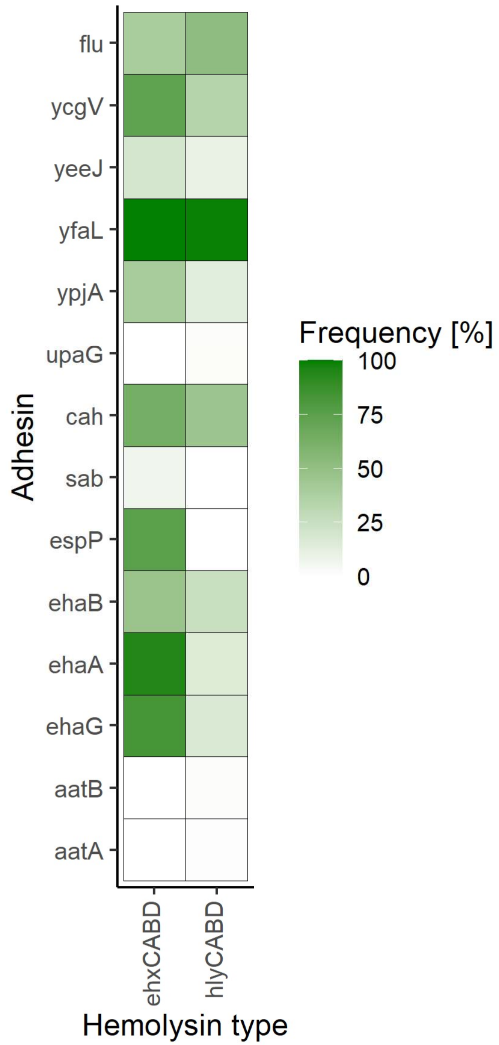

Adhesin
flu
ycgV
yeeJ
yfaL
ypjA
upaG
cah
sab
espP
ehaB
ehaA
ehaG
aatB
aatA
ehxCABD
hlyCABD
Hemolysin type
Frequency [%]
100
75
50
25
0

Figure S5. Frequency of autotransporter genes in alpha-hemolysin- and enterohemolysin-positive *E. coli.* Frequency of 14 autotransporter genes providing information about the difference of prevalence in 1122 hemolytic and 2399 enterohemolytic *E coli*. Hemolysin type is shown on the x-axis. Autotransporter genes are listed on the y-axis. The colour gradient is proportional to the frequency of each gene, and the colour scale is shown on the legends attached to heatmap.

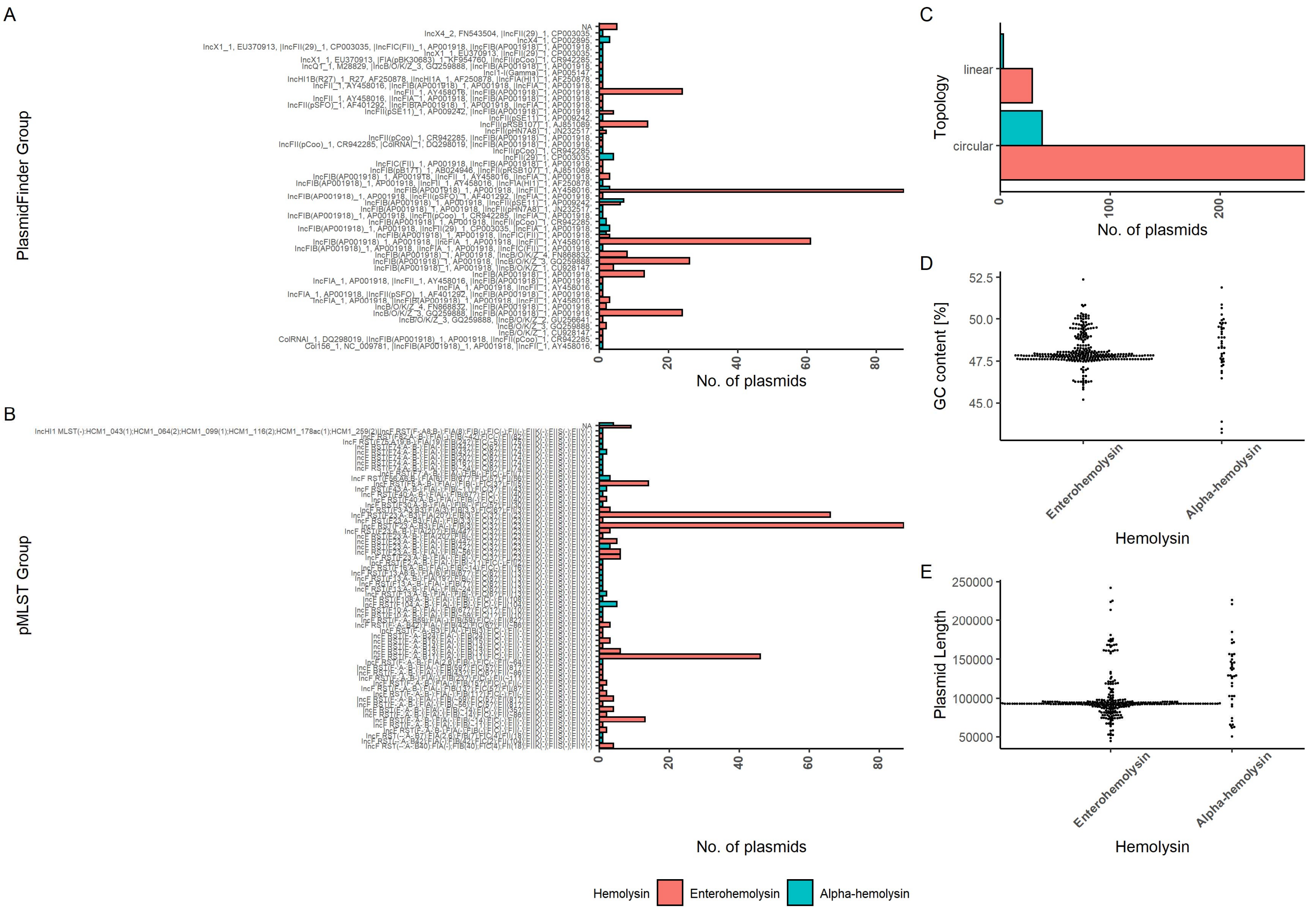
A
PlasmidFinder Group
No. of plasmids
0
20
40
60
80
B
pMLST Group
No. of plasmids
C
Topology
linear
circular
No. of plasmids
0
100
200
D
GC content [%]
52.5
50.0
47.5
45.0
Enterohemolysin
Alpha-hemolysin
Hemolysin
E
Plasmid Length
250000
200000
150000
100000
50000
Enterohemolysin
Alpha-hemolysin
Hemolysin
Hemolysin
Enterohemolysin
Alpha-hemolysin

Figure S6. Basic information about plasmids encoding alpha-hemolysin and enterohemolysin genes

Plasmids bearing alpha-hemolysin (n=41) and enterohemolysin (n=306) downloaded from PLSDB. Plots A and B contain information about PlasmidFinder and pMLST groups for analysed plasmids. Topology of collected plasmids is shown in plot C. Plot D and E present information about GC content and length. Number of plasmids is shown on the x-axis in plots A, B and C. Hemolysin type is shown on the x-axis in plots D and E. PlasmidFinder group (A), pMLST group (B), topology (C), GC content (D) and plasmids length (E) are shown on the y-axis. Bar colours on plots A, B and C represent different hemolysins and are described on the legend below the plots.

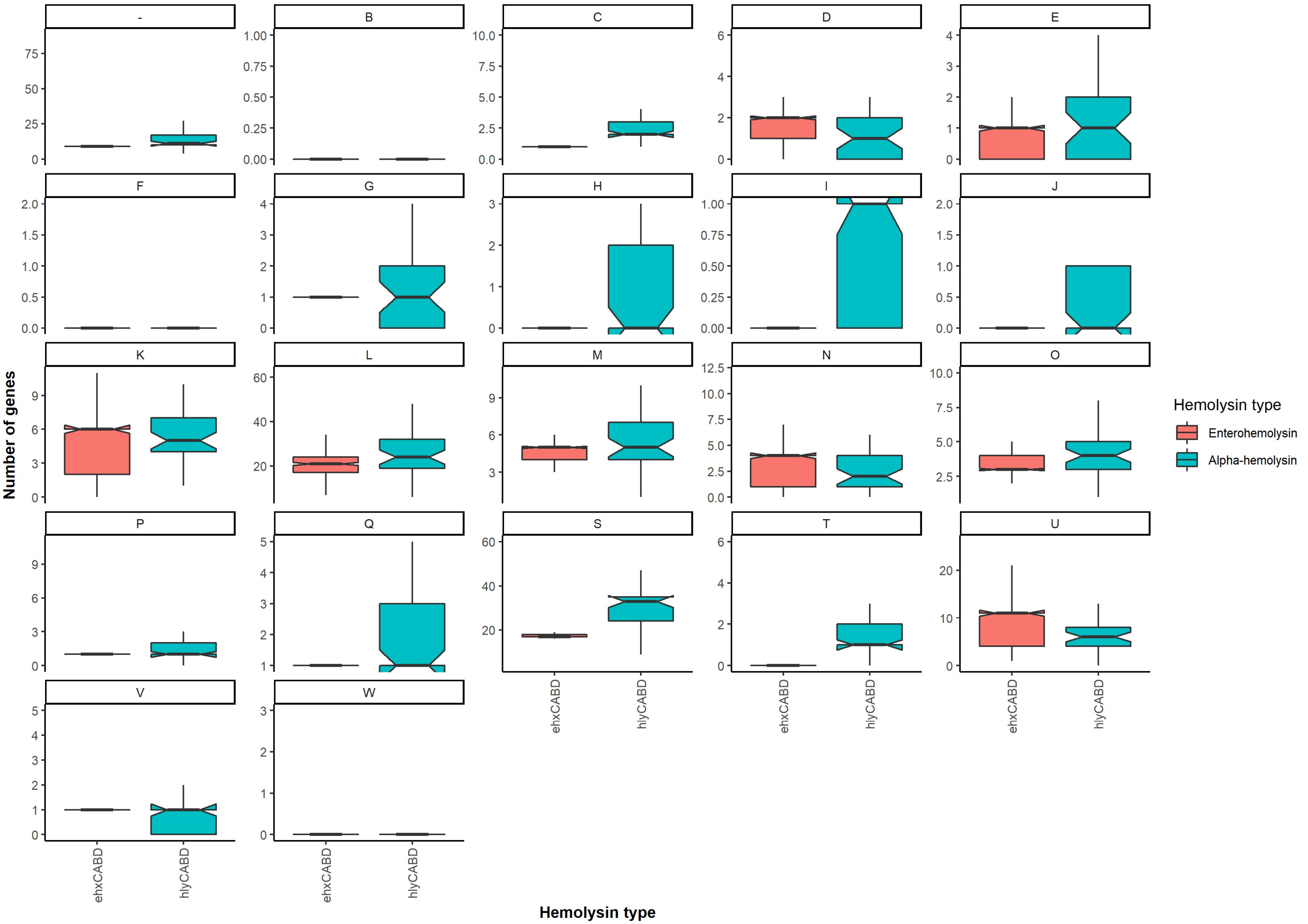

-
B
C
D
E
F
G
H
I
J
K
L
M
N
O
P
Q
S
T
U
V
W
Number of genes
Hemolysin type
ehxCABD
hlyCABD
Hemolysin type
Enterohemolysin
Alpha-hemolysin

Figure S7. Comparison of gene frequency in various functional groups found in alpha-hemolysin and enterohemolysin-bearing plasmids

Box-and-whisker plot with gene frequency belonging to various functional groups in alpha-hemolysin and enterohemolysin-bearing plasmids. Genes present in plasmids were annotated to functional groups with use of eggnog-mapper and functional group prevalence alpha-hemolysin and enterohemolysin-bearing plasmids were compared and shown. The bottom and top of each box represent the first (25%) and third (75%) quartiles, and bands inside the boxes are second quartiles (medians). Notches in the boxes show the 95% confidence intervals for medians. Whiskers (vertical lines) extend from the boxes to a maximum of 1.5x of the interquartile range. Hemolysin type is shown on the x-axes and number of genes belonging to a particular COG group is showin on the y-axis. Meaning of abbreviations: J- Translation, including ribosome structure and biogenesis; K- Transcription; L- Replication, recombination and repair; B- Chromatin structure and dynamics; D- Cell division and chromosome partitioning; V- Defence mechanisms; T- Signal transduction; M- Cell wall structure and biogenesis and outer membrane; N- Secretion, motility and chemotaxis; W- Extracellular structures; U- Intracellular trafficking, secretion, and vesicular transport; O- Molecular chaperones and related functions; C- Energy production and conversion; G- Carbohydrate metabolism and transport; E- Amino acid metabolism and transport; F- Nucleotide metabolism and transport; H- Coenzyme metabolism; I- Lipid metabolism; P- Inorganic ion transport and metabolism; Q- Secondary metabolites biosynthesis, transport and catabolism; S- Function unknown; “-”- functional group not determined by eggnog-mapper.

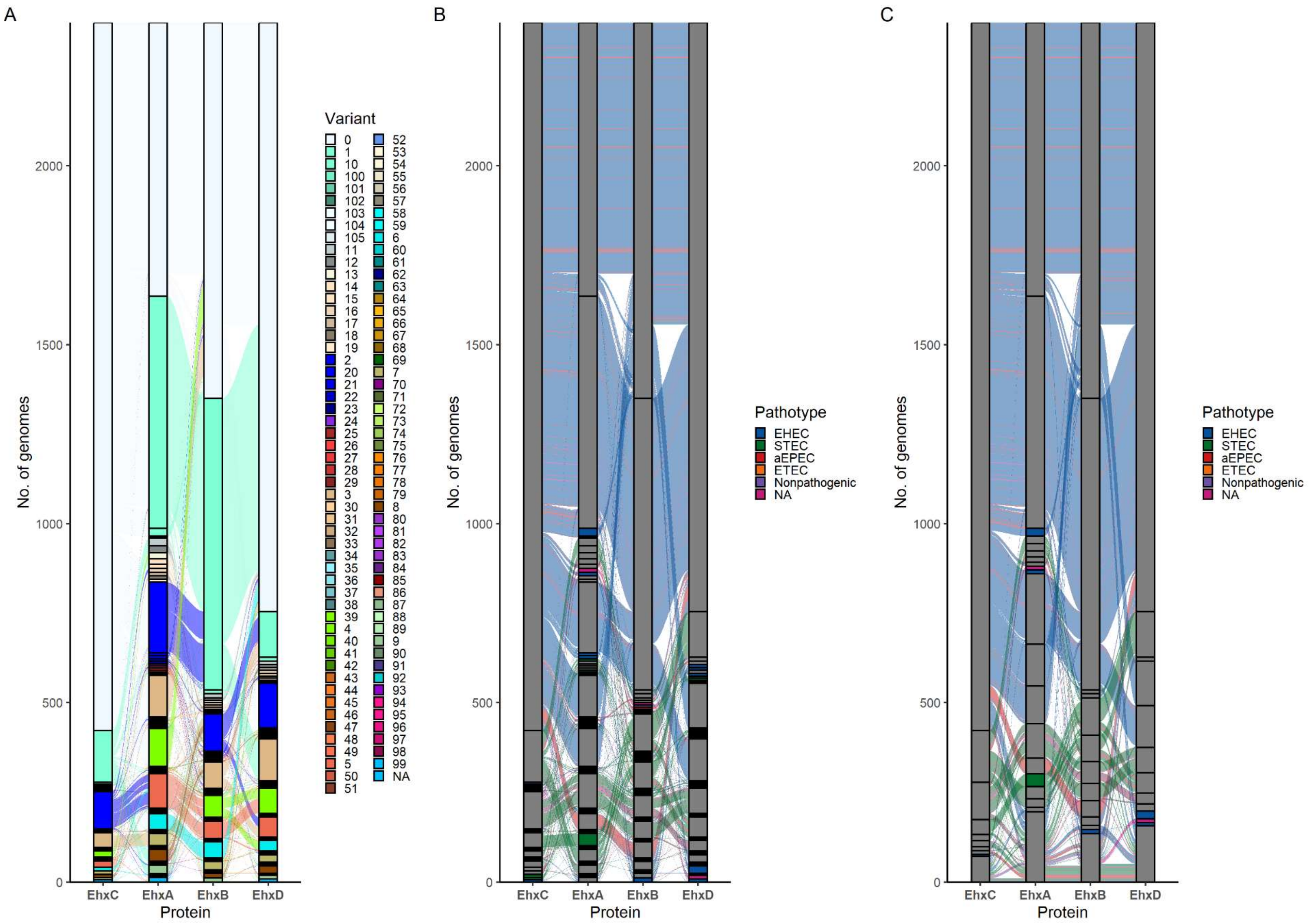
A
B
C
No. of genomes
0
500
1000
1500
2000
EhxC
EhxA
EhxB
EhxD
Protein
Variant
0 1 10 100 101 102 103 104 105 11 12 13 14 15 16 17 18 19 2 20 21 22 23 24 25 26 27 28 29 3 30 31 32 33 34 35 36 37 38 39 4 40 41 42 43 44 45 46 47 48 49 5 50 51
52 53 54 55 56 57 58 59 6 60 61 62 63 64 65 66 67 68 69 7 70 71 72 73 74 75 76 77 78 79 8 80 81 82 83 84 85 86 87 88 89 9 90 91 92 93 94 95 96 97 98 99 NA
Pathotype
EHEC
STEC
aEPEC
ETEC
Nonpathogenic
NA

Figure S8. EhxCABD variants in enterohemolysin-positive *E. coli*

Analysis of EhxCABD variant prevalence in enterohemolysin-positive *E. coli* enriched with information about *E. coli* pathotype. Number of genomes is shown on the y-axis and names of the proteins are shown on the x-axis. Colour streams and bars represent protein variants (A) or pathotype (B, C) and are shown separately on the right side of each plot. In plot B and C, the bar has grey colour if one variant is present in more than one pathotype. All genomes with no coding sequence for one of the analysed proteins are shown as NA. All genomes with undetermined pathotype are shown as NA on the plot B and C. Variants present in less than 10 isolates were regrouped as "Other" in the plot C.

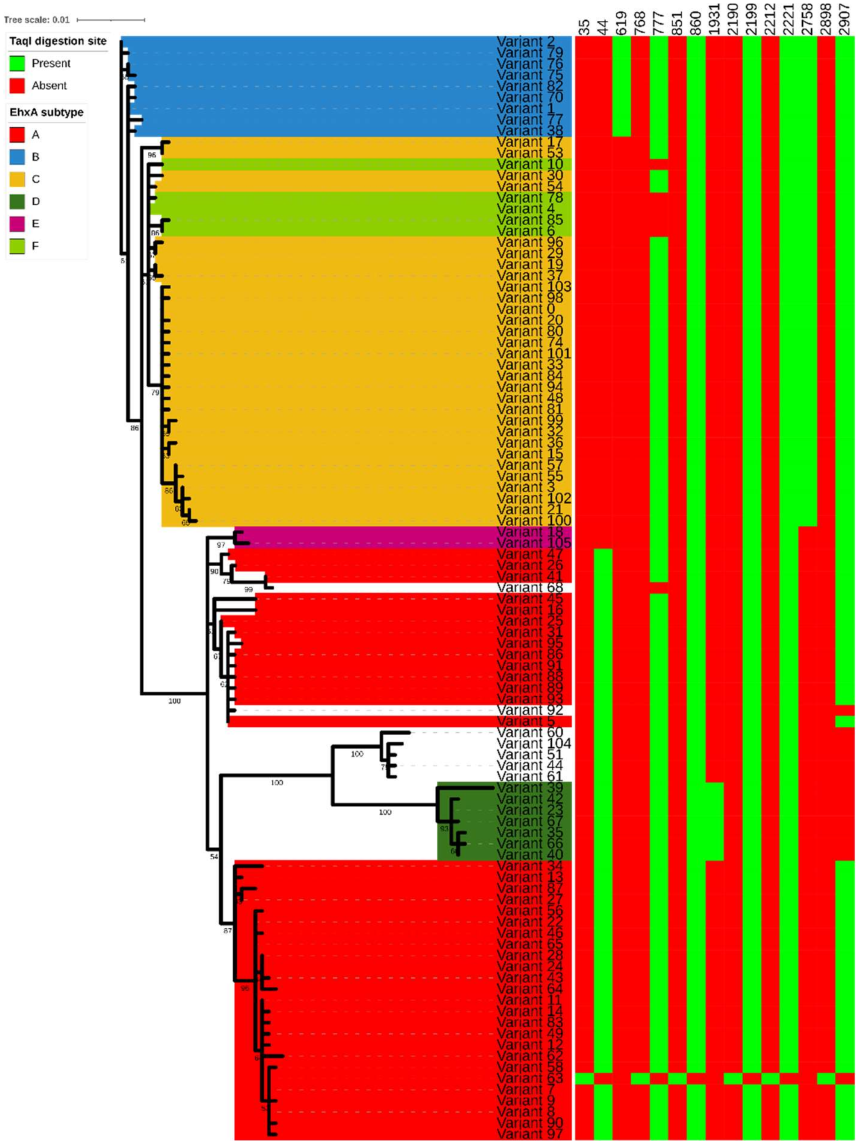
Tree scale: 0.01
TaqI digestion site
Present
Absent
EhxA subtype
A
B
C
D
E
F
35
44
619
768
777
851
860
1931
2190
2199
2212
2221
2758
2898
2907
Variant 2
Variant 79
Variant 76
Variant 75
Variant 82
Variant 70
Variant 1
Variant 77
Variant 38
Variant 17
Variant 53
Variant 10
Variant 30
Variant 54
Variant 78
Variant 4
Variant 85
Variant 6
Variant 96
Variant 29
Variant 19
Variant 37
Variant 103
Variant 98
Variant 0
Variant 20
Variant 80
Variant 74
Variant 101
Variant 33
Variant 84
Variant 94
Variant 48
Variant 81
Variant 99
Variant 32
Variant 36
Variant 15
Variant 57
Variant 55
Variant 3
Variant 102
Variant 21
Variant 100
Variant 18
Variant 105
Variant 47
Variant 26
Variant 41
Variant 68
Variant 45
Variant 16
Variant 25
Variant 31
Variant 95
Variant 86
Variant 91
Variant 88
Variant 89
Variant 93
Variant 92
Variant 5
Variant 60
Variant 104
Variant 51
Variant 44
Variant 61
Variant 39
Variant 42
Variant 23
Variant 67
Variant 35
Variant 66
Variant 40
Variant 34
Variant 13
Variant 87
Variant 27
Variant 56
Variant 22
Variant 46
Variant 65
Variant 28
Variant 24
Variant 43
Variant 64
Variant 11
Variant 14
Variant 83
Variant 49
Variant 12
Variant 62
Variant 58
Variant 63
Variant 7
Variant 9
Variant 8
Variant 90
Variant 97

Figure S9. EhxA subtypes and phylogenetic analysis

Subtyping and phylogenetic analysis of 99 functional variants of EhxA found in 2399 enterohemolysin-positive *E. coli* genomes. EhxA subtypes and TaqI digestion sites were annotated with the use of iTOL. Colour keys for EhxA subtypes marked on the phylogenetic tree and heatmap with TaqI digestion sites are shown on the legend named "EhxA subtype" and "TaqI digestion site", respectively. Variants without indicated EhxA subtype represent a novel subtype identified in this study.
