# Supplementary material for: Genomic characterization of enterohaemolysin-encoding haemolytic Escherichia coli of animal and human origin
Source: Microb Genom. 2023 Apr 27;9(4):mgen000999. doi: 10.1099/mgen.0.000999 (PMC10210957; doi:10.1099/mgen.0.000999)
Supplement: Supplementary material 2 [file mgen-9-999-s002.pdf]

Genome	Phylogroup	Country	ST	Serotype	Genome_placement	Host	Hemolytic	BAPS
GCA_018431605	B2	Germany	33	O6:H31	Chromosome	Mallard	hlyCABD	1
GCA_018431645	B2	Germany	27	O2.Gp7/O2.O50.Gp7:H6	Chromosome	Mallard	hlyCABD	2
GCA_018431625	B2	Germany	4	O22:H1	Chromosome	Mallard	hlyCABD	3
GCA_018431585	B2	Germany	123	O35:H31	Undeterimined	Raccoon	hlyCABD	28
GCA_018431565	B2	Germany	304	?:H10	Chromosome	Deer	hlyCABD	28
GCA_018431515	B2	Germany	26	O2.Gp7/O2.O50.Gp7:H6	Chromosome	Deer	hlyCABD	2
GCA_018431505	B2	Germany	83	O6:H5	Chromosome	Mallard	hlyCABD	4
GCA_018431545	B2	Germany	12	O4:H5	Chromosome	Mallard	hlyCABD	5
GCA_018431485	B2	Germany	127	O6:H31	Chromosome	Marten	hlyCABD	1
GCA_018431455	B2	Germany	83	O6:H5	Chromosome	Mallard	hlyCABD	4
GCA_018431425	B2	Germany	12	O4:H5	Chromosome	Mallard	hlyCABD	5
GCA_018431435	B2	Germany	304	?:H10	Chromosome	Deer	hlyCABD	28
GCA_018431405	B2	Germany	304	?:H10	Chromosome	Deer	hlyCABD	28
GCA_018431385	B1	Germany	86	O23:H16	Chromosome	Mallard	hlyCABD	25
GCA_018431305	B2	Germany	22	O6:H31	Chromosome	Mallard	hlyCABD	1
GCA_018431325	B2	Germany	32	O6:H31	Chromosome	Mallard	hlyCABD	1
GCA_018431335	B2	Germany	882	O4:H5	Chromosome	Fox	hlyCABD	5
GCA_018431315	B2	Germany	83	O6:H5	Chromosome	Fox	hlyCABD	4
GCA_018431285	B2	Germany	12	O4:H5	Chromosome	Marten	hlyCABD	5
GCA_018431265	B2	Germany	882	O4:H5	Chromosome	Fox	hlyCABD	5
GCA_018431225	B2	Germany	2558	O2.Gp7/O2.O50.Gp7:H14	Chromosome	Fox	hlyCABD	28
GCA_018431235	B2	Germany	7092	O6:H5	Chromosome	Raccoon	hlyCABD	4
GCA_018431205	B2	Germany	26	O2.Gp7/O2.O50.Gp7:H6	Chromosome	Deer	hlyCABD	2
GCA_018431185	B2	Germany	32	O6:H31	Chromosome	Pig	hlyCABD	1
GCA_018431165	B2	Germany	32	O6:H31	Chromosome	Pig	hlyCABD	1
GCA_018431125	A	Germany	10	O36:H19	Plasmid	Pig	hlyCABD	24
GCA_018431105	A	Germany	10	O36:H19	Plasmid	Pig	hlyCABD	24
GCA_018431145	A	Germany	10	O2.Gp7/O2.O50.Gp7:H32	Plasmid	Pig	hlyCABD	24
GCA_018431085	A	Germany	10	O36:H19	Plasmid	Pig	hlyCABD	24
GCA_018431065	A	Germany	1112	O142:H27	Plasmid	Pig	hlyCABD	23
GCA_018430985	A	Germany	10	O36:H19	Plasmid	Pig	hlyCABD	24
GCA_018431035	B2	Germany	26	O2.Gp7/O2.O50.Gp7:H6	Chromosome	Deer	hlyCABD	2
GCA_018431005	B2	Germany	26	O2.Gp7/O2.O50.Gp7:H6	Chromosome	Deer	hlyCABD	2
GCA_018431015	B1	Germany	392	?:H2	Plasmid	Fox	hlyCABD	25
GCA_018430965	B2	Germany	22	O6:H31	Chromosome	Human	hlyCABD	1
GCA_018430935	B2	Germany	83	O6:H5	Chromosome	Pig	hlyCABD	4
GCA_018430925	B2	Germany	27	O2.Gp7/O2.O50.Gp7:H6	Chromosome	Human	hlyCABD	2
GCA_018430885	B2	Germany	43	Onovel31:H4	Chromosome	Pig	hlyCABD	6
GCA_018430895	B2	Germany	4	O2.Gp7/O2.O50.Gp7:H1	Chromosome	Human	hlyCABD	3
GCA_018430865	B2	Germany	27	O2.Gp7/O2.O50.Gp7:H6	Chromosome	Human	hlyCABD	2
GCA_018430845	B2	Germany	1	O18.Gp12:H7	Chromosome	Human	hlyCABD	7
GCA_018430825	B2	Germany	4	O6:H1	Chromosome	Human	hlyCABD	3
GCA_018430805	B2	Germany	4	O6:H1	Chromosome	Human	hlyCABD	3
GCA_018430785	B2	Germany	27	O2.Gp7/O2.O50.Gp7:H6	Chromosome	Human	hlyCABD	2
GCA_018430745	B2	Germany	29	O6:H1	Chromosome	Human	hlyCABD	3
GCA_018430755	B2	Germany	27	O2.Gp7/O2.O50.Gp7:H6	Chromosome	Human	hlyCABD	2
GCA_018430725	B2	Germany	51	O75:H5	Chromosome	Human	hlyCABD	28
GCA_018430705	B2	Germany	9	Onovel31:H4	Chromosome	Human	hlyCABD	6
GCA_018430685	B2	Germany	9	Onovel31:H4	Chromosome	Human	hlyCABD	6
GCA_018430645	B2	Germany	10	O2.Gp7/O2.O50.Gp7:H6	Chromosome	Human	hlyCABD	2
GCA_018430615	A	Germany	2	O141:H4	Plasmid	Mallard	hlyCABD	24
GCA_018430585	A	Germany	1112	O142:H27	Plasmid	Pig	hlyCABD	23
GCA_018430605	B2	Germany	73	O22:H1	Chromosome	Mallard	hlyCABD	3
GCA_018430525	B2	Germany	26	O2.Gp7/O2.O50.Gp7:H6	Chromosome	Pig	hlyCABD	2
GCA_018430545	B2	Germany	83	O6:H5	Chromosome	Marten	hlyCABD	4
GCA_018430565.	A	Germany	10	O2.Gp7/O2.O50.Gp7:H32	Plasmid	Pig	hlyCABD	24
GCA_018430495	B2	Germany	83	O6:H5	Chromosome	Raccoon	hlyCABD	4
GCA_018430485	B2	Germany	83	O6:H5	Chromosome	Raccoon	hlyCABD	4
GCA_018430465	B2	Germany		O143:H27	Undeterimined	Raccoon	hlyCABD	28
GCA_018430395	B2	Germany	32	O6:H31	Chromosome	Human	hlyCABD	1
GCA_018430385	B2	Germany	1	O18.Gp12:H7	Chromosome	Human	hlyCABD	7
GCA_018430415	B2	Germany	6	O75:H5	Chromosome	Human	hlyCABD	28
GCA_018430405	B2	Germany	83	O6:H5	Chromosome	Raccoon	hlyCABD	4
GCA_018430365	B2	Germany	620	O4:H5	Chromosome	Marten	hlyCABD	5
GCA_018430295	B2	Germany	83	O6:H5	Chromosome	Raccoon	hlyCABD	4
GCA_018430345	B2	Germany	83	O6:H5	Chromosome	Raccoon	hlyCABD	4
GCA_018430285	B2	Germany	26	O2.Gp7/O2.O50.Gp7:H6	Chromosome	Marten	hlyCABD	2
GCA_018430325	B2	Germany	4	Onovel31/O25:H1	Chromosome	Marten	hlyCABD	3
GCA_018430265	B2	Germany	83	O6:H5	Chromosome	Raccoon	hlyCABD	4
GCA_018430245	B2	Germany	646	O4:H7	Undeterimined	Raccoon	hlyCABD	28
GCA_018430225	B2	Germany	83	O6:H5	Chromosome	Raccoon	hlyCABD	4
GCA_018430185	B2	Germany	26	O2.Gp7/O2.O50.Gp7:H6	Chromosome	Deer	hlyCABD	2
GCA_018430665	A	Germany	2	O2.Gp7/O2.O50.Gp7:H25	Undeterimined	Mallard	hlyCABD	24
GCA_018430195	B2	Germany	27	O2.Gp7/O2.O50.Gp7:H6	Chromosome	Human	hlyCABD	2
GCA_018430165	B2	Germany	83	O6:H5	Chromosome	Human	hlyCABD	4
GCA_018430115	B2	Germany	33	O6:H31	Chromosome	Human	hlyCABD	1
GCA_018430145	B1	Germany	360	O6:H49	Undeterimined	Human	hlyCABD	25

GCA_018430085	B2	Germany	29	O2.Gp7/O2.O50.Gp7:H1	Chromosome	Human	hlyCABD	3
GCA_018430105	B2	Germany	4260	O54:H14	Chromosome	Human	hlyCABD	28
GCA_018430065	B2	Germany	509	O6:H7	Chromosome	Human	hlyCABD	28
GCA_018430045	B2	Germany	509	O6:H7	Chromosome	Human	hlyCABD	28
GCA_000164295	B2	Unknown	4	O18.Gp12:H1	Chromosome	Human	hlyCABD	3
GCA_000164575	B2	Unknown	4	O22:H1	Chromosome	Human	hlyCABD	3
GCA_000259695	B2	Unknown	52	O6:H1	Chromosome	Unknown	hlyCABD	3
GCA_000317395	B2	China	52	O6:H1	Chromosome	Unknown	hlyCABD	3
GCA_000326165	B2	Unknown	36	O4:H5	Chromosome	Unknown	hlyCABD	5
GCA_000326225	B2	Unknown	4	O18.Gp12:H1	Chromosome	Unknown	hlyCABD	3
GCA_000326285	B2	Unknown	22	O6:H31	Chromosome	Unknown	hlyCABD	1
GCA_000326305	B2	Unknown	129	O2.Gp7/O2.O50.Gp7:H6	Chromosome	Unknown	hlyCABD	2
GCA_000326385	B2	Unknown	4	O6:H1	Chromosome	Unknown	hlyCABD	3
GCA_000326405	B2	Unknown	52	O6:H1	Chromosome	Unknown	hlyCABD	3
GCA_000326445	B2	Unknown	26	O2.Gp7/O2.O50.Gp7:H6	Chromosome	Unknown	hlyCABD	2
GCA_000326465	B2	Unknown	70	O1:H7	Chromosome	Unknown	hlyCABD	7
GCA_000326525	B2	Unknown	929	O21:H14	Chromosome	Unknown	hlyCABD	28
GCA_000326605	B2	Unknown	52	O6:H1	Chromosome	Unknown	hlyCABD	3
GCA_000326705	B2	Unknown	26	O2.Gp7/O2.O50.Gp7:H6	Chromosome	Unknown	hlyCABD	2
GCA_000326905	B2	Unknown	52	?:H1	Chromosome	Unknown	hlyCABD	3
GCA_000331615	B2	China	52	O6:H1	Chromosome	Unknown	hlyCABD	3
GCA_000350865	B2	Unknown	4	Onovel31/O25:H1	Chromosome	Unknown	hlyCABD	3
GCA_000350965	B2	Unknown	4	Onovel31/O25:H1	Chromosome	Human	hlyCABD	3
GCA_000350985	B2	Unknown	12	O4:H5	Chromosome	Human	hlyCABD	5
GCA_000351005	B2	Unknown	36	O4:H5	Chromosome	Human	hlyCABD	5
GCA_000351045	B2	Unknown	4	O18.Gp12:H1	Chromosome	Human	hlyCABD	3
GCA_000351105	B2	Unknown	22	O6:H31	Chromosome	Human	hlyCABD	1
GCA_000351205	B2	Unknown	4	Onovel31/O25:H1	Chromosome	Human	hlyCABD	3
GCA_000351245	B2	Unknown	52	O6:H1	Chromosome	Human	hlyCABD	3
GCA_000351265	B2	Unknown	26	O2.Gp7/O2.O50.Gp7:H6	Chromosome	Human	hlyCABD	2
GCA_000351305	B2	Unknown	4	Onovel31/O25:H1	Chromosome	Human	hlyCABD	3
GCA_000351485	B2	Denmark	4	O6:H1	Chromosome	Human	hlyCABD	3
GCA_000351505	B2	Denmark	1	O18.Gp12:H7	Chromosome	Human	hlyCABD	7
GCA_000351645	B2	Denmark	29	O2.Gp7/O2.O50.Gp7:H1	Chromosome	Human	hlyCABD	3
GCA_000351825	B2	Denmark	73	O6:H1	Chromosome	Human	hlyCABD	3
GCA_000351985	A	Denmark	10	O86:H2	Chromosome	Human	hlyCABD	24
GCA_000352025	B2	Denmark	26	O2.Gp7/O2.O50.Gp7:H6	Chromosome	Human	hlyCABD	2
GCA_000352085	B2	Unknown	127	O6:H31	Chromosome	Unknown	hlyCABD	1
GCA_000352225	B2	Unknown	26	O2.Gp7/O2.O50.Gp7:H6	Chromosome	Unknown	hlyCABD	2
GCA_000353005	B2	Unknown	129	O2.Gp7/O2.O50.Gp7:H6	Chromosome	Human	hlyCABD	2
GCA_000353065	B2	Unknown	26	O2.Gp7/O2.O50.Gp7:H6	Chromosome	Human	hlyCABD	2
GCA_000353105	B2	Unknown	4	O6:H1	Chromosome	Human	hlyCABD	3
GCA_000353125	B2	Unknown	52	O6:H1	Chromosome	Human	hlyCABD	3
GCA_000353145	B2	Unknown	70	O1:H7	Chromosome	Human	hlyCABD	7
GCA_000353185	B2	Unknown	12	O4:H5	Chromosome	Unknown	hlyCABD	5
GCA_000387785	B2	USA	10	O2.Gp7/O2.O50.Gp7:H6	Chromosome	Human	hlyCABD	2
GCA_000387825	B2	USA	12	?:H5	Chromosome	Human	hlyCABD	5
GCA_000397225	B2	USA	36	O4:H5	Chromosome	Human	hlyCABD	5
GCA_000397245	B2	USA		O4:H5	Chromosome	Human	hlyCABD	5
GCA_000397265	B2	USA	36	O4:H5	Chromosome	Human	hlyCABD	5
GCA_000397285	B2	USA	36	O4:H5	Chromosome	Human	hlyCABD	5
GCA_000397445	B2	USA	544	O4:H5	Chromosome	Human	hlyCABD	5
GCA_000397465	B2	USA	36	O4:H5	Chromosome	Human	hlyCABD	5
GCA_000397485	B2	USA	36	O4:H5	Chromosome	Human	hlyCABD	5
GCA_000397625	B2	USA	73	O22:H1	Chromosome	Human	hlyCABD	3
GCA_000397645	B2	USA	73	O22:H1	Chromosome	Human	hlyCABD	3
GCA_000397665	B2	USA	73	O22:H1	Chromosome	Human	hlyCABD	3
GCA_000397685	B2	USA	197	O22:H1	Chromosome	Human	hlyCABD	3
GCA_000397705	B2	USA	73	O22:H1	Chromosome	Human	hlyCABD	3
GCA_000401755	B2	Unknown	52	O6:H1	Chromosome	Unknown	hlyCABD	3
GCA_000407865	F	Unknown	728	O1:H7	Chromosome	Unknown	hlyCABD	29
GCA_000408085	B2	Unknown	929	O21:H14	Chromosome	Human	hlyCABD	28
GCA_000408125	B2	Unknown	12	O4:H1	Chromosome	Human	hlyCABD	5
GCA_000408285	B2	Unknown	52	O6:H1	Chromosome	Human	hlyCABD	3
GCA_000446805	B1	Austria	517	O111:H19	Plasmid	Human	hlyCABD	21
GCA_000456025	B2	Denmark	4	O22:H1	Chromosome	Human	hlyCABD	3
GCA_000456065	B2	Denmark	12	O4:H1	Chromosome	Human	hlyCABD	5
GCA_000456125	B2	Denmark	4	O6:H1	Chromosome	Human	hlyCABD	3
GCA_000456185	B2	Denmark	4	O22:H1	Chromosome	Human	hlyCABD	3
GCA_000456205	B2	Denmark	4	O6:H1	Chromosome	Human	hlyCABD	3
GCA_000456285	B2	Denmark	52	O6:H1	Chromosome	Human	hlyCABD	3
GCA_000456305	B2	Denmark	35	O4:H1	Chromosome	Human	hlyCABD	5
GCA_000456425	B2	Denmark	22	O6:H31	Chromosome	Human	hlyCABD	1
GCA_000456585	B2	Denmark	26	O2.Gp7/O2.O50.Gp7:H6	Chromosome	Human	hlyCABD	2
GCA_000456605	B2	Denmark	30	O2.Gp7/O2.O50.Gp7:H1	Chromosome	Human	hlyCABD	3
GCA_000456745	B2	Denmark	40	O83:H31	Chromosome	Human	hlyCABD	28
GCA_000456825	B2	Denmark	537	O75:H5	Chromosome	Human	hlyCABD	28
GCA_000456845	B2	Denmark	33	O6:H31	Chromosome	Human	hlyCABD	1

GCA_000456865	B2	Denmark	4	O6:H1	Chromosome	Human	hlyCABD	3
GCA_000456905	B2	Denmark	4	O6:H1	Chromosome	Human	hlyCABD	3
GCA_000457005	B2	Denmark	55	O16:H6	Chromosome	Human	hlyCABD	28
GCA_000457045	B2	Denmark	4	O6:H1	Chromosome	Human	hlyCABD	3
GCA_000457085	B2	Denmark	73	O18.Gp12:H1	Chromosome	Human	hlyCABD	3
GCA_000457105	B2	Denmark	12	O4:H1	Chromosome	Human	hlyCABD	5
GCA_000457265	B2	Denmark	4	O6:H1	Chromosome	Human	hlyCABD	3
GCA_000457325	B2	Denmark	4	O6:H1	Chromosome	Human	hlyCABD	3
GCA_000457385	B2	Denmark	4	O6:H1	Chromosome	Human	hlyCABD	3
GCA_000457405	B2	Denmark	33	O6:H31	Chromosome	Human	hlyCABD	1
GCA_000457495	B2	Denmark	6	O75:H5	Chromosome	Human	hlyCABD	28
GCA_000457515	B2	Denmark	4	O2.Gp7/O2.O50.Gp7:H1	Chromosome	Human	hlyCABD	3
GCA_000457555	B2	Denmark	12	O4:H5	Chromosome	Human	hlyCABD	5
GCA_000457575	B2	Denmark	32	?:H31	Chromosome	Human	hlyCABD	1
GCA_000457615	B2	Denmark	30	O2.Gp7/O2.O50.Gp7:H1	Chromosome	Human	hlyCABD	3
GCA_000457695	B2	Denmark	4	O2.Gp7/O2.O50.Gp7:H1	Chromosome	Human	hlyCABD	3
GCA_000457775	C	Denmark	88	O11:H4	Chromosome	Human	hlyCABD	16
GCA_000457815	B2	Denmark	80	?:H7	Chromosome	Human	hlyCABD	28
GCA_000457875	B2	Denmark	6	O75:H5	Chromosome	Human	hlyCABD	28
GCA_000457915	B2	Denmark	4	O6:H1	Chromosome	Human	hlyCABD	3
GCA_000458015	B2	Denmark	12	O4:H1	Chromosome	Human	hlyCABD	5
GCA_000458095	B2	Denmark	52	O6:H1	Chromosome	Human	hlyCABD	3
GCA_000458135	B2	Denmark	4	O6:H1	Chromosome	Human	hlyCABD	3
GCA_000458275	B2	Denmark	40	O83:H31	Chromosome	Human	hlyCABD	28
GCA_000458335	B2	Denmark	4	Onovel31/O25:H1	Chromosome	Human	hlyCABD	3
GCA_000458355	B2	Denmark	4	O6:H1	Chromosome	Human	hlyCABD	3
GCA_000458375	B2	Denmark	55	O16:H6	Chromosome	Human	hlyCABD	28
GCA_000458395	B2	Denmark	4	O6:H1	Chromosome	Human	hlyCABD	3
GCA_000458415	B2	Denmark	4	O6:H1	Chromosome	Human	hlyCABD	3
GCA_000458435	B2	Denmark	4	O18.Gp12:H1	Chromosome	Human	hlyCABD	3
GCA_000458535	B2	Denmark	4	O6:H1	Chromosome	Human	hlyCABD	3
GCA_000458575	B2	Denmark	12	O4:H5	Chromosome	Human	hlyCABD	5
GCA_000458605	B2	Denmark	4	O6:H1	Chromosome	Human	hlyCABD	3
GCA_000458765	B2	Denmark	32	O6:H31	Chromosome	Human	hlyCABD	1
GCA_000458935	B2	Denmark	144	O16:H6	Chromosome	Human	hlyCABD	28
GCA_000458975	B2	Denmark	70	O1:H7	Chromosome	Human	hlyCABD	7
GCA_000458995	B2	Denmark	12	O4:H5	Chromosome	Human	hlyCABD	5
GCA_000459135	B2	Denmark	73	?:H1	Chromosome	Human	hlyCABD	3
GCA_000459195	B2	Denmark	29	O120:H31	Chromosome	Human	hlyCABD	3
GCA_000459295	B2	Denmark	4	O6:H1	Chromosome	Human	hlyCABD	3
GCA_000459355	B2	Denmark	30	O2.Gp7/O2.O50.Gp7:H1	Chromosome	Human	hlyCABD	3
GCA_000459395	B2	Denmark	4	O6:H1	Chromosome	Human	hlyCABD	3
GCA_000459415	B2	Denmark	4	O6:H1	Chromosome	Human	hlyCABD	3
GCA_000459515	B2	Denmark	4	O6:H1	Chromosome	Human	hlyCABD	3
GCA_000459535	B2	Denmark	12	O4:H5	Chromosome	Human	hlyCABD	5
GCA_000459595	B2	Denmark	55	O16:H6	Chromosome	Human	hlyCABD	28
GCA_000459615	B2	Denmark	32	O6:H31	Chromosome	Human	hlyCABD	1
GCA_000459695	B2	Denmark	29	O6:H1	Chromosome	Human	hlyCABD	3
GCA_000459715	B2	Denmark	29	O6:H1	Chromosome	Human	hlyCABD	3
GCA_000459735	B2	Denmark	80	O75:H7	Chromosome	Human	hlyCABD	28
GCA_000459755	B2	Denmark	32	O6:H31	Chromosome	Human	hlyCABD	1
GCA_000459835	B2	Denmark	33	O6:H31	Chromosome	Human	hlyCABD	1
GCA_000459975	B2	Sweden	22	O6:H31	Chromosome	Human	hlyCABD	1
GCA_000460055	B2	Sweden	537	O75:H5	Chromosome	Human	hlyCABD	28
GCA_000460095	B2	Sweden	29	O6:H1	Chromosome	Human	hlyCABD	3
GCA_000460115	B2	Sweden	6	O75:H5	Chromosome	Human	hlyCABD	28
GCA_000460135	B2	Sweden	127	O6:H31	Chromosome	Human	hlyCABD	1
GCA_000460175	B2	Sweden	32	O6:H31	Chromosome	Human	hlyCABD	1
GCA_000460215	B2	Sweden	73	O6:H1	Chromosome	Human	hlyCABD	3
GCA_000460415	B2	Sweden	4	O6:H1	Chromosome	Human	hlyCABD	3
GCA_000460435	B2	Sweden	372	O83:H31	Chromosome	Human	hlyCABD	28
GCA_000460455	B2	Sweden	29	O2.Gp7/O2.O50.Gp7:H1	Chromosome	Human	hlyCABD	3
GCA_000460535	B2	Sweden	4	Onovel31/O25:H1	Chromosome	Human	hlyCABD	3
GCA_000460575	B2	Sweden	4	O6:H1	Chromosome	Human	hlyCABD	3
GCA_000460615	B2	Sweden	4	O22:H1	Chromosome	Human	hlyCABD	3
GCA_000460635	A	Sweden	10	O3:H2	Chromosome	Human	hlyCABD	24
GCA_000460655	B2	Sweden	12	O4:H5	Chromosome	Human	hlyCABD	5
GCA_000460695	B2	Sweden	12	O4:H5	Chromosome	Human	hlyCABD	5
GCA_000460815	B2	Sweden	4	Onovel31/O25:H1	Chromosome	Human	hlyCABD	3
GCA_000460895	B2	Sweden	22	O6:H31	Chromosome	Human	hlyCABD	1
GCA_000460915	B2	Sweden	4	O18.Gp12:H1	Chromosome	Human	hlyCABD	3
GCA_000460935	B2	Sweden	80	O75:H7	Chromosome	Human	hlyCABD	28
GCA_000460975	B2	Sweden	80	O75:H7	Chromosome	Human	hlyCABD	28
GCA_000460995	B2	Sweden	4	Onovel31/O25:H1	Chromosome	Human	hlyCABD	3
GCA_000461035	B2	Sweden	12	O4:H5	Chromosome	Human	hlyCABD	5
GCA_000461055	B2	Sweden	4	O2.Gp7/O2.O50.Gp7:H1	Chromosome	Human	hlyCABD	3
GCA_000461075	B2	Sweden	73	O6:H1	Chromosome	Human	hlyCABD	3
GCA_000461095	B2	Sweden	73	O6:H1	Chromosome	Human	hlyCABD	3

GCA_000461135	B1	Sweden	29	O119:H8	Undeterimined	Human	hlyCABD	27
GCA_000461155	B2	Sweden	1	O18.Gp12:H7	Chromosome	Human	hlyCABD	7
GCA_000461275	B2	Sweden	4	Onovel31/O25:H1	Chromosome	Human	hlyCABD	3
GCA_000461295	B2	Sweden	22	O6:H31	Chromosome	Human	hlyCABD	1
GCA_000461355	B2	Sweden	4	Onovel31/O25:H1	Chromosome	Human	hlyCABD	3
GCA_000461375	B2	Sweden	127	O6:H31	Chromosome	Human	hlyCABD	1
GCA_000461435	B2	Sweden	22	O6:H31	Chromosome	Human	hlyCABD	1
GCA_000461555	B2	Sweden	29	O6:H1	Chromosome	Human	hlyCABD	3
GCA_000461575	B2	Sweden	29	O6:H1	Chromosome	Human	hlyCABD	3
GCA_000461635	B2	Sweden	29	O22:H1	Chromosome	Human	hlyCABD	3
GCA_000461655	B2	Sweden	4	O6:H1	Chromosome	Human	hlyCABD	3
GCA_000461715	B2	Sweden	29	O6:H1	Chromosome	Human	hlyCABD	3
GCA_000461735	B2	Sweden	1	O18.Gp12:H7	Chromosome	Human	hlyCABD	7
GCA_000461815	B2	Sweden	12	O4:H5	Chromosome	Human	hlyCABD	5
GCA_000461835	B2	Sweden	12	O4:H5	Chromosome	Human	hlyCABD	5
GCA_000461855	B2	Sweden	217	O85:H1	Chromosome	Human	hlyCABD	28
GCA_000461875	B2	Sweden	29	O6:H1	Chromosome	Human	hlyCABD	3
GCA_000463605	B2	Sweden	4	O6:H1	Chromosome	Human	hlyCABD	3
GCA_000488035	B1	Denmark	88	O11:H12	Chromosome	Human	hlyCABD	25
GCA_000488095	B2	Sweden	22	O6:H31	Chromosome	Human	hlyCABD	1
GCA_000488115	B2	Sweden	12	O4:H5	Chromosome	Human	hlyCABD	5
GCA_000488155	B2	Sweden	29	O6:H1	Chromosome	Human	hlyCABD	3
GCA_000488315	B2	Unknown	52	O6:H1	Chromosome	Human	hlyCABD	3
GCA_000488455	D	Unknown	44	O2.Gp7/O2.O50.Gp7:NA	Chromosome	Human	hlyCABD	29
GCA_000488475	B2	Unknown	4	O6:H1	Chromosome	Human	hlyCABD	3
GCA_000488635	D	Unknown	14	O153var1:H6	Chromosome	Human	hlyCABD	29
GCA_000488815	B2	Unknown	52	O6:H1	Chromosome	Human	hlyCABD	3
GCA_000488835	B2	Unknown	33	O6:H31	Chromosome	Human	hlyCABD	1
GCA_000494975	B2	Denmark	4	O6:H1	Chromosome	Human	hlyCABD	3
GCA_000498815	B2	Lebanon	43	Onovel31:H4	Chromosome	Unknown	hlyCABD	6
GCA_000506445	B2	Lebanon	52	O6:H1	Chromosome	Human	hlyCABD	3
GCA_000507605	B2	Denmark	543	O4:H1	Chromosome	Human	hlyCABD	3
GCA_000507625	B2	Denmark	4	O22:H1	Chromosome	Human	hlyCABD	3
GCA_000599745	B2	USA	73	O22:H1	Chromosome	Human	hlyCABD	3
GCA_000599825	D	USA	3	O15:H6	Chromosome	Human	hlyCABD	9
GCA_000601255	A	Tanzania	10	O117.Gp8:H10	Chromosome	Human	hlyCABD	24
GCA_000618905	B1	USA	381	O39:H9	Undeterimined	Unknown	hlyCABD	25
GCA_000619245	B1	USA	5082	O121:H7	Plasmid	Unknown	hlyCABD	25
GCA_000627905	A	Tanzania	10	O117.Gp8:H10	Chromosome	Human	hlyCABD	24
GCA_000627945	A	Tanzania	10	O117.Gp8:H10	Chromosome	Human	hlyCABD	24
GCA_000633655	B2	Unknown	12	O4:H5	Chromosome	Human	hlyCABD	5
GCA_000692715	B2	USA	43	Onovel31:H4	Chromosome	Human	hlyCABD	6
GCA_000695505	B2	USA	43	Onovel31:H4	Chromosome	Human	hlyCABD	6
GCA_000696545	B2	Australia	33	O6:H31	Chromosome	Human	hlyCABD	1
GCA_000696835	A	China	100	O149:H10	Plasmid	Pig	hlyCABD	19
GCA_000699365	B2	USA	127	O6:H31	Chromosome	Unknown	hlyCABD	1
GCA_000700085	B1	Tanzania	5251	O128:H8	Undeterimined	Human	hlyCABD	27
GCA_000700145	B2	Tanzania	12	O18.Gp12:H5	Chromosome	Human	hlyCABD	5
GCA_000700405	B1	Tanzania		O128:H8	Undeterimined	Human	hlyCABD	27
GCA_000700705	A	Tanzania	10	O117.Gp8:H10	Chromosome	Human	hlyCABD	24
GCA_000703585	A	Tanzania	378	?:H33	Undeterimined	Human	hlyCABD	24
GCA_000703865	D	Tanzania	38	O86:H5	Chromosome	Human	hlyCABD	29
GCA_000703885	D	Tanzania	8	O86:H5	Chromosome	Human	hlyCABD	29
GCA_000704345	B2	Tanzania	43	O18.Gp12:H4	Chromosome	Human	hlyCABD	6
GCA_000704445	B2	Tanzania	43	Onovel31:H4	Chromosome	Human	hlyCABD	6
GCA_000711355	B1	Tanzania	223	O132:H16	Chromosome	Human	hlyCABD	22
GCA_000711365	B2	Tanzania	543	O6:H1	Chromosome	Human	hlyCABD	3
GCA_000711375	D	Tanzania	38	O153var1:H2	Chromosome	Human	hlyCABD	29
GCA_000711435	B2	Tanzania	73	O6:H1	Chromosome	Human	hlyCABD	3
GCA_000711455	B2	Tanzania	73	O6:H1	Chromosome	Human	hlyCABD	3
GCA_000711485	B2	Tanzania	131	Onovel31:H4	Chromosome	Human	hlyCABD	6
GCA_000711525	B2	Tanzania	827	O4:H40	Chromosome	Human	hlyCABD	5
GCA_000711555	B2	Tanzania		O18.Gp12:H4	Chromosome	Human	hlyCABD	6
GCA_000711595	B1	Tanzania	223	O132:H16	Chromosome	Human	hlyCABD	22
GCA_000713035	B2	Tanzania	827	O4:H40	Chromosome	Human	hlyCABD	5
GCA_000713045	B2	Tanzania	827	O4:H40	Chromosome	Human	hlyCABD	5
GCA_000713135	D	Tanzania	8	O153var1:H2	Chromosome	Human	hlyCABD	29
GCA_000713185	B2	Tanzania	36	O4:H40	Chromosome	Human	hlyCABD	5
GCA_000713455	B2	Tanzania	73	O6:H1	Chromosome	Human	hlyCABD	3
GCA_000713495	B2	Tanzania	73	O6:H1	Chromosome	Human	hlyCABD	3
GCA_000713585	B2	Tanzania	73	O6:H1	Chromosome	Human	hlyCABD	3
GCA_000713615	B1	Tanzania	223	O132:H16	Chromosome	Human	hlyCABD	22
GCA_000713825	B1	Tanzania	223	O132:H16	Chromosome	Human	hlyCABD	22
GCA_000713855	A	Tanzania	5793	O176:H33	Chromosome	Human	hlyCABD	24
GCA_000713865	B2	Tanzania		Onovel31:H4	Chromosome	Human	hlyCABD	6
GCA_000713935	B1	Tanzania	29	O71:H8	Undeterimined	Human	hlyCABD	27
GCA_000713945	A	Tanzania	10	O117.Gp8:H10	Undeterimined	Human	hlyCABD	24
GCA_000713975	A	Tanzania	10	O117.Gp8:H10	Chromosome	Human	hlyCABD	24

GCA_000714345	B2	Tanzania	827	O4:H40	Chromosome	Human	hlyCABD	5
GCA_000714375	B2	Tanzania	827	O4:H40	Chromosome	Human	hlyCABD	5
GCA_000753035	B2	Norway	80	O75:H7	Chromosome	Sheep	hlyCABD	28
GCA_000753195	B2	Norway	27	O2.Gp7/O2.O50.Gp7:H6	Chromosome	Human	hlyCABD	2
GCA_000768405	A	Germany	10	?:NA	Undeterimined	Human	hlyCABD	24
GCA_000768465	A	Germany	10	O169.Gp16:NA	Undeterimined	Human	hlyCABD	24
GCA_000768505	A	Germany	10	O169.Gp16:NA	Undeterimined	Human	hlyCABD	24
GCA_000776615	B2	USA	80	O75:H7	Chromosome	Human	hlyCABD	28
GCA_000778335	B2	USA	4	O6:H1	Chromosome	Human	hlyCABD	3
GCA_000778375	B2	USA	70	O1:H7	Chromosome	Human	hlyCABD	7
GCA_000778415	B2	USA	73	Onovel31/O25:H1	Chromosome	Human	hlyCABD	3
GCA_000778685	B2	USA	33	O6:H31	Chromosome	Human	hlyCABD	1
GCA_000778955	B2	USA	127	O6:H31	Chromosome	Human	hlyCABD	1
GCA_000779255	B2	USA	12	O4:H5	Chromosome	Human	hlyCABD	5
GCA_000779715	B2	USA	372	O83:H31	Chromosome	Human	hlyCABD	28
GCA_000779815	B2	USA	929	O21:H14	Chromosome	Human	hlyCABD	28
GCA_000780095	B2	USA	1	O18.Gp12:H7	Chromosome	Human	hlyCABD	7
GCA_000780155	B2	USA	127	O6:H31	Chromosome	Human	hlyCABD	1
GCA_000780195	B2	USA	12	O4:H5	Chromosome	Human	hlyCABD	5
GCA_000780335	B2	USA	80	O2.Gp7/O2.O50.Gp7:H7	Chromosome	Human	hlyCABD	28
GCA_000780735	B2	USA	127	O6:H31	Chromosome	Human	hlyCABD	1
GCA_000780925	B2	USA	95	O18.Gp12:H7	Chromosome	Human	hlyCABD	7
GCA_000781355	B2	USA	4	O6:H1	Chromosome	Human	hlyCABD	3
GCA_000781575	B2	USA	95	O18.Gp12:H7	Chromosome	Human	hlyCABD	7
GCA_000782695	B2	USA	1	O18.Gp12:H7	Chromosome	Human	hlyCABD	7
GCA_000782715	B2	USA	43	Onovel31:H4	Chromosome	Human	hlyCABD	6
GCA_000782775	B2	USA	29	O2.Gp7/O2.O50.Gp7:H1	Chromosome	Human	hlyCABD	3
GCA_000800675	B2	Spain	4	O6:H1	Chromosome	Human	hlyCABD	3
GCA_000807555	B2	Spain	4	O6:H1	Chromosome	Human	hlyCABD	3
GCA_000807565	B2	Spain	4	O6:H1	Chromosome	Human	hlyCABD	3
GCA_000807575	B2	Spain	4	O6:H1	Chromosome	Human	hlyCABD	3
GCA_000807635	B2	Spain	4	O6:H1	Chromosome	Human	hlyCABD	3
GCA_000807655	B2	Spain	4	O6:H1	Chromosome	Human	hlyCABD	3
GCA_000817345	B2	USA	1	O18.Gp12:H7	Chromosome	Human	hlyCABD	7
GCA_000817355	B2	USA	1	O18.Gp12:H7	Chromosome	Human	hlyCABD	7
GCA_000817375	B2	USA	26	O2.Gp7/O2.O50.Gp7:H6	Chromosome	Human	hlyCABD	2
GCA_000819105	A	USA	10	O84:H11	Plasmid	Unknown	hlyCABD	24
GCA_000835045	C	Australia	885	O157:H19	Plasmid	Unknown	hlyCABD	16
GCA_000835055	C	Australia	885	?:H19	Plasmid	Unknown	hlyCABD	16
GCA_000954035	B1	Israel	302	O139:H19	Plasmid	Cattle	hlyCABD	25
GCA_000954045	A	Israel	10	O105:H32	Plasmid	Cattle	hlyCABD	24
GCA_001005685	A	China	5786	O141:H4	Plasmid	Pig	hlyCABD	24
GCA_001012535	B1	USA		O121:H7	Plasmid	Cattle	hlyCABD	25
GCA_001012575	B1	USA	154	O142:H38	Plasmid	Cattle	hlyCABD	25
GCA_001030435	B2	Unknown	4	O2.Gp7/O2.O50.Gp7:H1	Chromosome	Human	hlyCABD	3
GCA_001030445	B2	Unknown	4	O2.Gp7/O2.O50.Gp7:H1	Chromosome	Human	hlyCABD	3
GCA_001030665	B2	Unknown	1	O18.Gp12:H7	Chromosome	Human	hlyCABD	7
GCA_001032735	D	USA	44	O2.Gp7/O2.O50.Gp7:H4	Chromosome	Human	hlyCABD	29
GCA_001039215	B1	France	29	?:H11	Plasmid	Human	hlyCABD	27
GCA_001191215	B1	USA	2217	O45:H16	Plasmid	Cattle	hlyCABD	25
GCA_001262455	B2	Canada	95	O18.Gp12:H7	Chromosome	Human	hlyCABD	7
GCA_001263065	B1	USA	154	O142:H38	Plasmid	Cattle	hlyCABD	25
GCA_001265625	A	Gambia	367	O70:H40	Undeterimined	Human	hlyCABD	24
GCA_001265685	B1	Gambia	381	O177:H9	Undeterimined	Human	hlyCABD	25
GCA_001277395	B1	Kenya	517	O165:H9	Undeterimined	Human	hlyCABD	21
GCA_001277415	A	Bangladesh	10	O70:H40	Undeterimined	Human	hlyCABD	24
GCA_001277475	B1	Kenya	517	O165:H9	Undeterimined	Human	hlyCABD	21
GCA_001277615	B1	India	546	?:H19	Plasmid	Human	hlyCABD	21
GCA_001277695	E	India	4424	O84:H9	Plasmid	Human	hlyCABD	29
GCA_001277735	A	Mali	10	O70:H40	Undeterimined	Human	hlyCABD	24
GCA_001283305	A	India	378	?:H33	Undeterimined	Human	hlyCABD	24
GCA_001283325	A	India	378	?:H33	Undeterimined	Human	hlyCABD	24
GCA_001283385	B1	India	517	O71:H19	Plasmid	Human	hlyCABD	21
GCA_001283425	B1	India	517	O71:H19	Plasmid	Human	hlyCABD	21
GCA_001283565	A	Pakistan	378	?:H33	Undeterimined	Human	hlyCABD	24
GCA_001283665	A	Gambia	10	O70:H40	Undeterimined	Human	hlyCABD	24
GCA_001283765	B1	Mali	29	O82:NA	Undeterimined	Human	hlyCABD	27
GCA_001283785	A	Mozambique	367	O70:H40	Undeterimined	Human	hlyCABD	24
GCA_001283805	B1	India	517	O71:H19	Plasmid	Human	hlyCABD	21
GCA_001283945	A	Mozambique	367	O70:H40	Undeterimined	Human	hlyCABD	24
GCA_001284205	B1	Mali	517	O116var1:H9	Undeterimined	Human	hlyCABD	21
GCA_001284345	B1	India	517	O71:H19	Plasmid	Human	hlyCABD	21
GCA_001284365	A	Kenya	378	?:H33	Undeterimined	Human	hlyCABD	24
GCA_001284465	A	Mali	378	?:H33	Undeterimined	Human	hlyCABD	24
GCA_001284505	B1	Kenya	29	O26:H8	Undeterimined	Human	hlyCABD	27
GCA_001284525	B1	Pakistan	381	O177:H9	Undeterimined	Human	hlyCABD	25
GCA_001284565	A	Mozambique	367	O70:H40	Undeterimined	Human	hlyCABD	24
GCA_001284585	A	Mali	378	?:H33	Undeterimined	Human	hlyCABD	24

GCA_001284885	B1	India	29	O34:H9	Undeterimined	Human	hlyCABD	27
GCA_001284925	B1	Kenya	29	O2var1:H8	Undeterimined	Human	hlyCABD	27
GCA_001284945	B1	India	546	?:H19	Plasmid	Human	hlyCABD	21
GCA_001285225	B1	Mali	517	O116var1:H9	Undeterimined	Human	hlyCABD	21
GCA_001285305	B1	India	546	?:H19	Plasmid	Human	hlyCABD	21
GCA_001285325	B1	Kenya	517	O116var1:H9	Undeterimined	Human	hlyCABD	21
GCA_001285345	A	Mali	10	O70:H40	Undeterimined	Human	hlyCABD	24
GCA_001285405	B1	Gambia	546	O171:H19	Plasmid	Human	hlyCABD	21
GCA_001285425	B1	Mali	29	O82:NA	Undeterimined	Human	hlyCABD	27
GCA_001285465	A	Kenya	5330	?:H33	Undeterimined	Human	hlyCABD	24
GCA_001285485	B1	Mozambique	381	O103:H9	Undeterimined	Human	hlyCABD	25
GCA_001285625	A	Mali	367	O70:H40	Undeterimined	Human	hlyCABD	24
GCA_001285685	B1	India	546	?:H19	Plasmid	Human	hlyCABD	21
GCA_001285785	A	India	378	?:H33	Undeterimined	Human	hlyCABD	24
GCA_001285865	B1	India	29	O34:H9	Undeterimined	Human	hlyCABD	27
GCA_001285885	B1	Kenya	29	O2var1:H8	Undeterimined	Human	hlyCABD	27
GCA_001285965	B1	Kenya	29	O71:H8	Undeterimined	Human	hlyCABD	27
GCA_001285985	B1	Gambia	517	O171:H19	Plasmid	Human	hlyCABD	21
GCA_001286045	B1	India	517	O71:H19	Plasmid	Human	hlyCABD	21
GCA_001286145	B1	India	29	O34:H9	Undeterimined	Human	hlyCABD	27
GCA_001286205	B1	Pakistan	29	O34:H9	Undeterimined	Human	hlyCABD	27
GCA_001286285	A	India	378	?:H33	Undeterimined	Human	hlyCABD	24
GCA_001286385	B1	Kenya	481	O128:H8	Undeterimined	Human	hlyCABD	27
GCA_001286405	B1	Mozambique	29	O38:H9	Undeterimined	Human	hlyCABD	27
GCA_001286445	B1	India	29	O34:H9	Undeterimined	Human	hlyCABD	27
GCA_001286545	B1	Kenya	481	O128:H8	Undeterimined	Human	hlyCABD	27
GCA_001286645	B1	India	546	?:H19	Undeterimined	Human	hlyCABD	21
GCA_001306575	B2	USA	4	O2.Gp7/O2.O50.Gp7:H1	Chromosome	Human	hlyCABD	3
GCA_001306585	B2	USA	129	O2.Gp7/O2.O50.Gp7:H6	Chromosome	Human	hlyCABD	2
GCA_001306605	B2	USA	43	?:H4	Chromosome	Human	hlyCABD	6
GCA_001309615	D	Canada	30	O17.O44.O77.Gp9/O44.Gp9:H18	Chromosome	Unknown	hlyCABD	29
GCA_001419965	A	Tanzania	2	O28ab:H9	Chromosome	Human	hlyCABD	24
GCA_001448025	B2	USA	12	O4:H1	Chromosome	Human	hlyCABD	5
GCA_001462715	C	Norway	410	O8:H9	Chromosome	Human	hlyCABD	16
GCA_001463205	B2	Norway	6355	O18.Gp12:H5	Chromosome	Human	hlyCABD	5
GCA_001518355	B2	USA	52	O6:H1	Chromosome	Human	hlyCABD	3
GCA_001519125	B2	USA	1278	O6:H1	Chromosome	Human	hlyCABD	3
GCA_001519135	B2	USA	1	O18.Gp12:H7	Chromosome	Human	hlyCABD	7
GCA_001519215	B2	USA	52	O6:H1	Chromosome	Human	hlyCABD	3
GCA_001519235	B2	USA	1	O18.Gp12:H7	Chromosome	Human	hlyCABD	7
GCA_001519285	B2	USA	1	O18.Gp12:H7	Chromosome	Human	hlyCABD	7
GCA_001519315	B2	USA	33	O15:H31	Chromosome	Human	hlyCABD	1
GCA_001519475	B2	USA	29	O6:H1	Chromosome	Human	hlyCABD	3
GCA_001519485	B2	USA	127	O6:H31	Chromosome	Human	hlyCABD	1
GCA_001519555	B2	USA	1	O18.Gp12:H7	Chromosome	Human	hlyCABD	7
GCA_001519595	B2	USA	4	O6:H1	Chromosome	Human	hlyCABD	3
GCA_001519645	B2	USA	12	O4:H5	Chromosome	Human	hlyCABD	5
GCA_001519675	B2	USA	12	O18.Gp12:H5	Chromosome	Human	hlyCABD	5
GCA_001519715	B2	USA	1	O18.Gp12:H7	Chromosome	Human	hlyCABD	7
GCA_001519735	B2	USA	1	O18.Gp12:H7	Chromosome	Human	hlyCABD	7
GCA_001519755	B2	USA	1	O18.Gp12:H7	Chromosome	Human	hlyCABD	7
GCA_001520555	B2	USA	73	O2.Gp7/O2.O50.Gp7:H1	Chromosome	Human	hlyCABD	3
GCA_001520595	B2	USA	43	Onovel31:H4	Chromosome	Human	hlyCABD	6
GCA_001520715	B2	USA	33	O6:H31	Chromosome	Human	hlyCABD	1
GCA_001520775	B2	USA	1	O18.Gp12:H7	Chromosome	Human	hlyCABD	7
GCA_001520815	B2	USA	543	O6:H1	Chromosome	Human	hlyCABD	3
GCA_001521015	B2	USA	12	O4:H5	Chromosome	Human	hlyCABD	5
GCA_001521155	B2	USA	52	O6:H1	Chromosome	Human	hlyCABD	3
GCA_001521315	B2	USA	12	O4:H5	Chromosome	Human	hlyCABD	5
GCA_001521355	B2	USA	4	O2.Gp7/O2.O50.Gp7:H1	Chromosome	Human	hlyCABD	3
GCA_001521635	B2	USA	12	O4:H5	Chromosome	Human	hlyCABD	5
GCA_001571505	B1	USA	29	O26:H11	Plasmid	Cattle	hlyCABD	27
GCA_001571565	B2	Canada	1	O18.Gp12:H7	Chromosome	Human	hlyCABD	7
GCA_001571575	B2	Canada	4	O2.Gp7/O2.O50.Gp7:H1	Chromosome	Human	hlyCABD	3
GCA_001571645	B2	Canada	55	O16:H6	Chromosome	Human	hlyCABD	28
GCA_001571755	B2	Canada	647	O109:H5	Plasmid	Human	hlyCABD	7
GCA_001571825	B1	USA	29	O26:H11	Plasmid	Cattle	hlyCABD	27
GCA_001571835	B1	USA	29	O26:H11	Plasmid	Cattle	hlyCABD	27
GCA_001571845	B1	USA	29	O26:H11	Plasmid	Cattle	hlyCABD	27
GCA_001571885	B1	USA	29	O26:H11	Plasmid	Cattle	hlyCABD	27
GCA_001571905	B1	USA	29	O26:H11	Plasmid	Cattle	hlyCABD	27
GCA_001571965	B1	USA	29	?:H11	Plasmid	Cattle	hlyCABD	27
GCA_001571985	B1	USA	29	O26:H11	Plasmid	Cattle	hlyCABD	27
GCA_001572005	B1	USA	29	O26:H11	Plasmid	Cattle	hlyCABD	27
GCA_001572045	B1	USA	29	O26:H11	Plasmid	Cattle	hlyCABD	27
GCA_001572135	B1	USA	29	O26:H11	Plasmid	Cattle	hlyCABD	27
GCA_001572145	B1	USA	29	O26:H11	Plasmid	Cattle	hlyCABD	27
GCA_001572185	B1	USA	29	O26:H11	Plasmid	Cattle	hlyCABD	27

GCA_001572205	B1	USA	29	O26:H11	Plasmid	Cattle	hlyCABD	27
GCA_001572215	B1	USA	29	O26:H11	Plasmid	Cattle	hlyCABD	27
GCA_001572245	B1	USA	29	O26:NA	Plasmid	Cattle	hlyCABD	27
GCA_001572265	B1	USA	29	O26:H11	Plasmid	Cattle	hlyCABD	27
GCA_001572285	B1	USA	29	O26:H11	Plasmid	Cattle	hlyCABD	27
GCA_001572345	B1	USA	29	O26:H11	Plasmid	Cattle	hlyCABD	27
GCA_001572365	B1	USA	29	O26:H11	Plasmid	Cattle	hlyCABD	27
GCA_001572375	B1	USA	29	O26:H11	Plasmid	Cattle	hlyCABD	27
GCA_001572405	B1	USA	29	O26:H11	Plasmid	Cattle	hlyCABD	27
GCA_001572425	B1	USA	29	O26:NA	Plasmid	Cattle	hlyCABD	27
GCA_001572445	B1	USA	29	O26:H11	Plasmid	Cattle	hlyCABD	27
GCA_001572485	B1	USA	29	O26:H11	Plasmid	Cattle	hlyCABD	27
GCA_001572505	B1	USA	29	O26:H11	Plasmid	Cattle	hlyCABD	27
GCA_001572525	B1	USA	29	O26:H11	Plasmid	Cattle	hlyCABD	27
GCA_001572535	B1	USA	29	O26:H11	Plasmid	Cattle	hlyCABD	27
GCA_001572565	B1	USA	29	O26:NA	Plasmid	Cattle	hlyCABD	27
GCA_001572635	B1	USA	29	O26:H11	Plasmid	Cattle	hlyCABD	27
GCA_001605825	B2	India	43	Onovel31:H4	Chromosome	Human	hlyCABD	6
GCA_001608125	B2	Netherlands	80	O75:H7	Chromosome	Human	hlyCABD	28
GCA_001614495	B1	China	392	?:H2	Plasmid	Sheep	hlyCABD	25
GCA_001614855	A	China		O149:H10	Plasmid	Pig	hlyCABD	19
GCA_001615125	C	China	90	?:H19	Undeterimined	Pig	hlyCABD	16
GCA_001615225	B1	China	392	?:H2	Plasmid	Sheep	hlyCABD	25
GCA_001615625	A	China	100	O149:H10	Plasmid	Pig	hlyCABD	19
GCA_001615785	B1	China	1642	?:H7	Undeterimined	Pig	hlyCABD	25
GCA_001615895	B1	China	529	O29:H12	Plasmid	Pig	hlyCABD	25
GCA_001616045	D	China	114	O139:H1	Plasmid	Pig	hlyCABD	29
GCA_001616095	A	China	4214	O3:H45	Undeterimined	Pig	hlyCABD	23
GCA_001616175	A	China	2	O141:H4	Undeterimined	Pig	hlyCABD	24
GCA_001616215	A	China	100	O149:H10	Plasmid	Pig	hlyCABD	19
GCA_001616345	A	China	4214	O3:H45	Plasmid	Pig	hlyCABD	23
GCA_001616375	A	China	2	O141:H4	Undeterimined	Pig	hlyCABD	24
GCA_001616415	A	China	5995	O3:H27	Plasmid	Pig	hlyCABD	19
GCA_001621225	B2	USA	33	O6:H31	Chromosome	Human	hlyCABD	1
GCA_001621675	B2	USA	73	O2.Gp7/O2.O50.Gp7:H1	Chromosome	Human	hlyCABD	3
GCA_001621745	B2	USA	43	Onovel31:H4	Chromosome	Human	hlyCABD	6
GCA_001621885	B2	USA	52	O6:H1	Chromosome	Human	hlyCABD	3
GCA_001621915	B2	USA	12	O4:H5	Chromosome	Human	hlyCABD	5
GCA_001621945	B2	USA	43	Onovel31:H4	Chromosome	Human	hlyCABD	6
GCA_001621995	B2	USA	4	O6:H1	Chromosome	Human	hlyCABD	3
GCA_001637785	C	Germany	88	O21:NA	Chromosome	Cattle	hlyCABD	16
GCA_001650605	D	United Kingdom	42	O138:H14	Plasmid	Pig	hlyCABD	29
GCA_001651625	B2	Unknown	12	O4:H5	Chromosome	Human	hlyCABD	5
GCA_001651685	B1	Unknown	442	O146:H21	Undeterimined	Human	hlyCABD	17
GCA_001651725	B2	Unknown	1858	O6:H5	Chromosome	Human	hlyCABD	28
GCA_001865185	B2	Germany	998	O2.Gp7/O2.O50.Gp7:H6	Chromosome	Human	hlyCABD	2
GCA_001865915	B2	Slovakia	12	O4:H5	Chromosome	Human	hlyCABD	5
GCA_001865925	B2	Estonia	12	O4:H1	Chromosome	Human	hlyCABD	5
GCA_001865985	B2	Sweden	12	O4:H1	Chromosome	Human	hlyCABD	5
GCA_001997075	B2	Unknown	52	O6:H1	Chromosome	Unknown	hlyCABD	3
GCA_002002125	B2	Australia	52	O6:H1	Chromosome	Human	hlyCABD	3
GCA_002002165	B2	Australia	52	O6:H1	Chromosome	Human	hlyCABD	3
GCA_002002465	B2	Australia	32	O6:H31	Chromosome	Human	hlyCABD	1
GCA_002087575	B2	USA	73	O6:H1	Chromosome	Human	hlyCABD	3
GCA_002087615	B2	USA	4	O6:H1	Chromosome	Human	hlyCABD	3
GCA_002087735	B2	USA	1	O18.Gp12:H7	Chromosome	Human	hlyCABD	7
GCA_002087775	B2	USA	4	O6:H1	Chromosome	Human	hlyCABD	3
GCA_002133385	B2	Brazil	882	?:H5	Chromosome	Human	hlyCABD	5
GCA_002133905	B1	Canada	1	O139:H1	Plasmid	Unknown	hlyCABD	29
GCA_002134235	B1	Canada	350	O146:H21	Undeterimined	Unknown	hlyCABD	17
GCA_002134325	B1	Canada	1	O139:H1	Plasmid	Unknown	hlyCABD	29
GCA_002152145	B2	USA	51	O75:H5	Chromosome	Human	hlyCABD	28
GCA_002163275	B2	USA	12	?:H5	Chromosome	Human	hlyCABD	5
GCA_002189715	B2	USA	73	Onovel31/O25:H1	Chromosome	Human	hlyCABD	3
GCA_002189745	B2	USA	12	O4:H5	Chromosome	Human	hlyCABD	5
GCA_002189905	B2	Sweden	80	?:H7	Chromosome	Human	hlyCABD	28
GCA_002223845	B1	China	88	O51:H4	Chromosome	Human	hlyCABD	25
GCA_002225505	C	USA	90	O149:H19	Plasmid	Pig	hlyCABD	16
GCA_002225565	B1	USA	58	O121:H21	Plasmid	Cattle	hlyCABD	22
GCA_002225655	A	USA	2	O141:H4	Plasmid	Cattle	hlyCABD	24
GCA_002225665	C	USA	90	O149:H43	Undeterimined	Pig	hlyCABD	16
GCA_002227295	B1	USA	641	O121:H10	Plasmid	Pig	hlyCABD	25
GCA_002227625	B1	USA	641	O121:H10	Plasmid	Pig	hlyCABD	25
GCA_002227665	B1	USA	641	O121:H10	Plasmid	Pig	hlyCABD	25
GCA_002228785	B1	USA	162	?:H19	Plasmid	Cattle	hlyCABD	21
GCA_002229495	A	USA		?:H25	Plasmid	Cattle	hlyCABD	25
GCA_002229695	E	USA	302	O45:H9	Plasmid	Dog	hlyCABD	23
GCA_002230735	B2	USA		O88:H4	Chromosome	Unknown	hlyCABD	25

GCA_002230765	B2	USA	509	O6:NA	Chromosome	Dog	hlyCABD	28
GCA_002230855	B2	USA	33	O6:H31	Chromosome	Dog	hlyCABD	1
GCA_002231575	B1	USA	2217	O76:H21	Plasmid	Cattle	hlyCABD	25
GCA_002231695	B2	USA	509	O6:H7	Chromosome	Cat	hlyCABD	28
GCA_002231995	C	USA	90	O149:H19	Plasmid	Pig	hlyCABD	16
GCA_002232915	A	USA	100	O149:H10	Plasmid	Pig	hlyCABD	19
GCA_002232975	B2	USA	129	O2.Gp7/O2.O50.Gp7:H6	Chromosome	Horse	hlyCABD	2
GCA_002233015	A	USA	10	O21:H2	Chromosome	Unknown	hlyCABD	24
GCA_002239785	B2	Thailand	131	Onovel31:H4	Chromosome	Human	hlyCABD	6
GCA_002239795	B2	Thailand	131	Onovel31:H4	Chromosome	Human	hlyCABD	6
GCA_002244015	B2	USA	33	O6:H31	Chromosome	Unknown	hlyCABD	1
GCA_002244065	B2	USA	33	O6:H31	Chromosome	Unknown	hlyCABD	1
GCA_002244125	B2	USA	1	O18.Gp12:H7	Chromosome	Unknown	hlyCABD	7
GCA_002244155	B2	USA	12	O4:H5	Chromosome	Unknown	hlyCABD	5
GCA_002244165	B2	USA	1	O18.Gp12:H7	Chromosome	Unknown	hlyCABD	7
GCA_002244195	B2	USA	12	O4:H5	Chromosome	Unknown	hlyCABD	5
GCA_002244205	B2	USA	12	O4:H5	Chromosome	Unknown	hlyCABD	5
GCA_002244235	B2	USA	12	O4:H5	Chromosome	Unknown	hlyCABD	5
GCA_002244245	B2	USA	33	O6:H31	Chromosome	Unknown	hlyCABD	1
GCA_002244295	B2	USA	33	O6:H31	Chromosome	Unknown	hlyCABD	1
GCA_002244465	B2	USA	30	O6:H1	Chromosome	Unknown	hlyCABD	3
GCA_002244735	B2	USA	29	O6:H1	Chromosome	Unknown	hlyCABD	3
GCA_002244775	B2	USA	29	O6:H1	Chromosome	Unknown	hlyCABD	3
GCA_002244925	B2	USA	12	O4:H5	Chromosome	Unknown	hlyCABD	5
GCA_002245015	B2	USA	33	O6:H31	Chromosome	Unknown	hlyCABD	1
GCA_002245175	B2	USA	33	O6:H31	Chromosome	Unknown	hlyCABD	1
GCA_002247695	A	Thailand	10	O2.Gp7/O2.O50.Gp7:H32	Undeterimined	Pig	hlyCABD	24
GCA_002247975	B1	Thailand	5218	?:H7	Plasmid	Pig	hlyCABD	25
GCA_002248255	B2	Thailand	131	Onovel31:H4	Chromosome	Human	hlyCABD	6
GCA_002248265	B2	Thailand	131	O16:H5	Chromosome	Human	hlyCABD	6
GCA_002248905	B2	Thailand	43	Onovel31:H4	Chromosome	Human	hlyCABD	6
GCA_002319315	B1	USA	56	O71:H21	Undeterimined	Pig	hlyCABD	22
GCA_002319355	D	Ireland	1	O139:H1	Plasmid	Pig	hlyCABD	29
GCA_002319385	A	Hungary	10	O141:H4	Undeterimined	Pig	hlyCABD	24
GCA_002319595	B1	USA	641	O121:H10	Plasmid	Pig	hlyCABD	25
GCA_002324535	E	USA	6353	?:H18	Undeterimined	Cattle	hlyCABD	23
GCA_002407315	B2	India	43	O2.Gp7/O2.O50.Gp7:H4	Chromosome	Human	hlyCABD	6
GCA_002407375	B2	India	43	Onovel31:H4	Chromosome	Human	hlyCABD	6
GCA_002416865	B2	South Africa	4	O6:H1	Chromosome	Human	hlyCABD	3
GCA_002416955	B2	South Africa	129	?:H6	Chromosome	Human	hlyCABD	2
GCA_002456615	B2	USA	52	?:H1	Chromosome	Human	hlyCABD	3
GCA_002459045	B1	Germany	442	?:H21	Plasmid	Cattle	hlyCABD	17
GCA_002459205	E	Canada	302	O45:H9	Plasmid	Pig	hlyCABD	23
GCA_002459675	D	Argentina	44	O2.Gp7/O2.O50.Gp7:H4	Chromosome	Human	hlyCABD	29
GCA_002461695	B2	Unknown	1	O18.Gp12:H7	Chromosome	Human	hlyCABD	7
GCA_002463885	C	USA	7	?:H4	Plasmid	Pig	hlyCABD	16
GCA_002464315	B1	USA	21	O88:H25	Plasmid	Cattle	hlyCABD	22
GCA_002464345	C	USA	90	?:H43	Undeterimined	Pig	hlyCABD	16
GCA_002465245	B2	USA	12	O4:H1	Chromosome	Human	hlyCABD	5
GCA_002465645	B2	Mexico	136	?:H5	Undeterimined	Bat	hlyCABD	28
GCA_002465845	B2	USA	52	O6:H1	Chromosome	Human	hlyCABD	3
GCA_002465855	B2	USA	52	O6:H1	Chromosome	Human	hlyCABD	3
GCA_002468325	B2	USA	509	O6:H7	Chromosome	Mink	hlyCABD	28
GCA_002473535	B2	USA	4260	O54:H14	Chromosome	Pig	hlyCABD	28
GCA_002473565	B2	USA	6537	O54:H14	Chromosome	Human	hlyCABD	28
GCA_002484425	B1	USA	1992	O146:H21	Plasmid	Cattle	hlyCABD	17
GCA_002485995	D	USA	955	O139:H1	Undeterimined	Cattle	hlyCABD	29
GCA_002486305	B1	USA	6234	O163:H7	Plasmid	Pig	hlyCABD	25
GCA_002507495	B2	China	43	Onovel31:H4	Chromosome	Human	hlyCABD	6
GCA_002510125	B2	South Africa	129	O2.Gp7/O2.O50.Gp7:H6	Chromosome	Human	hlyCABD	2
GCA_002510165	B2	South Africa	43	Onovel31:H4	Chromosome	Human	hlyCABD	6
GCA_002513315	C	USA	90	?:H19	Plasmid	Horse	hlyCABD	16
GCA_002514895	B1	USA	86	O70:H10	Plasmid	Cattle	hlyCABD	25
GCA_002516935	B2	USA	537	O75:H5	Chromosome	Pig	hlyCABD	28
GCA_002517015	C	USA	90	O149:H19	Plasmid	Pig	hlyCABD	16
GCA_002517375	A	USA	10	O3:H2	Chromosome	Unknown	hlyCABD	24
GCA_002518135	A	USA	6609	O129.O13.O135.Gp10/O129.O13.Gp10:H30	Plasmid	Cattle	hlyCABD	23
GCA_002518465	A	USA	761	O176:H4	Undeterimined	Cattle	hlyCABD	23
GCA_002520015	B2	USA	43	Onovel31:H4	Chromosome	Pig	hlyCABD	6
GCA_002520265	B2	USA	509	O6:H7	Chromosome	Tortoise	hlyCABD	28
GCA_002520495	B1	USA	829	?:H21	Undeterimined	Sheep	hlyCABD	17
GCA_002521595	B2	USA	33	O6:H31	Chromosome	Human	hlyCABD	1
GCA_002522085	B2	Zambia	12	O18.Gp12:H5	Chromosome	Human	hlyCABD	5
GCA_002522095	A	Zambia	10	O107.Gp8/O107..Gp8:H27	Chromosome	Human	hlyCABD	24
GCA_002522355	B2	Zambia	12	O18.Gp12:H5	Chromosome	Human	hlyCABD	5
GCA_002522395	B2	Canada	55	O16:H6	Undeterimined	Human	hlyCABD	28
GCA_002522715	B2	USA	33	O6:H31	Chromosome	Human	hlyCABD	1
GCA_002549105	B2	USA	304	?:H5	Chromosome	Chicken	hlyCABD	28

GCA_002735045	B1	Switzerland	29	O26:H11	Plasmid	Human	hlyCABD	27
GCA_002735125	B1	Netherlands	29	O26:H11	Plasmid	Cattle	hlyCABD	27
GCA_002761595	B2	Hungary	1	O18.Gp12:H7	Chromosome	Human	hlyCABD	7
GCA_002761605	B2	Hungary	1	O18.Gp12:H7	Plasmid	Human	hlyCABD	7
GCA_002761685	B2	Hungary	4	?:H1	Chromosome	Human	hlyCABD	3
GCA_002761765	B2	Hungary	73	O6:H1	Chromosome	Human	hlyCABD	3
GCA_002764195	B1	Italy	29	O26:H11	Plasmid	Cattle	hlyCABD	27
GCA_002769495	B1	Japan	29	O26:H11	Plasmid	Human	hlyCABD	27
GCA_002803475	B2	Greece	4	O6:H1	Chromosome	Human	hlyCABD	3
GCA_002810025	B2	Portugal	22	O6:H31	Chromosome	Human	hlyCABD	1
GCA_002810085	B2	Sweden	51	O75:H5	Chromosome	Human	hlyCABD	28
GCA_002810105	B2	Greece	12	O4:H5	Chromosome	Human	hlyCABD	5
GCA_002855615	B2	Spain	43	Onovel31:H4	Chromosome	Human	hlyCABD	6
GCA_002855635	B2	Spain	43	Onovel31:H4	Chromosome	Human	hlyCABD	6
GCA_002855695	B2	Spain	43	Onovel31:H4	Chromosome	Human	hlyCABD	6
GCA_002855715	B2	Spain	9	Onovel31:H4	Chromosome	Human	hlyCABD	6
GCA_002861205	B2	USA	22	O6:H31	Chromosome	Human	hlyCABD	1
GCA_002861225	B2	USA	33	O6:H31	Chromosome	Human	hlyCABD	1
GCA_002923495	B1	Belgium	29	O26:H11	Plasmid	Cattle	hlyCABD	27
GCA_002943345	B1	Canada	58	O88:H25	Plasmid	Unknown	hlyCABD	22
GCA_002991785	B1	China	8539	Onovel20:H14	Chromosome	Unknown	hlyCABD	25
GCA_003008245	B2	India	131	Onovel31:H4	Chromosome	Human	hlyCABD	6
GCA_003203375	B2	Unknown	73	?:H1	Chromosome	Human	hlyCABD	3
GCA_003288975	B2	USA	43	Onovel31:H4	Chromosome	Rhesus macaque	hlyCABD	6
GCA_003290605	B1	China	517	O71:H19	Plasmid	Human	hlyCABD	21
GCA_003303235	B1	China	87	O28ab:H25	Chromosome	Human	hlyCABD	22
GCA_003303655	B1	China	13	O152:H8	Chromosome	Human	hlyCABD	25
GCA_003317905	B2	USA	1	O18.Gp12:H7	Chromosome	Human	hlyCABD	7
GCA_003321855	B2	Kosovo	32	O6:H31	Chromosome	Dog	hlyCABD	1
GCA_003333505	B2	Sweden	80	?:H7	Chromosome	Human	hlyCABD	28
GCA_003333615	B2	USA	73	Onovel31/O25:H1	Chromosome	Human	hlyCABD	3
GCA_003333775	E	Sweden	350	O112:H18	Chromosome	Human	hlyCABD	23
GCA_003334085	A	Sweden	52	O15:H14	Chromosome	Human	hlyCABD	23
GCA_003334535	D	Sweden	70	O23:H15	Chromosome	Human	hlyCABD	29
GCA_003357545	B1	USA	8555	O182:H8	Undeterimined	Unknown	hlyCABD	25
GCA_003358995	B2	USA	12	O4:H5	Chromosome	Unknown	hlyCABD	5
GCA_003388355	B2	France	43	Onovel31:H4	Chromosome	Human	hlyCABD	6
GCA_003388925	B2	France	73	O6:H1	Chromosome	Human	hlyCABD	3
GCA_003470505	B2	China	12	O4:H1	Chromosome	Human	hlyCABD	5
GCA_003628175	D	India	44	O102:H6	Chromosome	Human	hlyCABD	29
GCA_003666405	B1	New Zealand	88	O82:H8	Chromosome	Human	hlyCABD	25
GCA_003755965	B1	USA	641	O70:H10	Plasmid	Unknown	hlyCABD	25
GCA_003760965	B2	USA		O109:H5	Plasmid	Unknown	hlyCABD	7
GCA_003763045	B1	USA	829	O146:H21	Undeterimined	Unknown	hlyCABD	17
GCA_003770225	A	USA	10	?:H2	Chromosome	Unknown	hlyCABD	24
GCA_003774125	B1	USA	442	O91:H21	Plasmid	Unknown	hlyCABD	17
GCA_003774905	B1	USA	2521	O147:H7	Plasmid	Unknown	hlyCABD	25
GCA_003783005	B1	USA	1304	?:H7	Plasmid	Unknown	hlyCABD	25
GCA_003787665	D	USA	31	O15:H18	Chromosome	Unknown	hlyCABD	29
GCA_003791105	B1	USA	392	O182:H2	Plasmid	Unknown	hlyCABD	25
GCA_003791205	B1	USA	302	O139:H19	Plasmid	Unknown	hlyCABD	25
GCA_003791365	A	USA	8618	O123.O186.Gp5/O123.Gp5:H16	Plasmid	Unknown	hlyCABD	24
GCA_003793235	B2	USA	95	O2.Gp7/O2.O50.Gp7:H5	Plasmid	Unknown	hlyCABD	7
GCA_003793555	B1	USA	1992	?:H21	Plasmid	Unknown	hlyCABD	17
GCA_003794755	D	USA	1406	O51:H14	Plasmid	Unknown	hlyCABD	29
GCA_003795465	A	Bolivia	2	?:H9	Undeterimined	Human	hlyCABD	24
GCA_003880495	A	United Kingdom	478	?:H2	Chromosome	Human	hlyCABD	24
GCA_003883635	B1	United Kingdom	517	O185:H19	Undeterimined	Human	hlyCABD	21
GCA_003884295	B1	United Kingdom	381	?:H9	Undeterimined	Human	hlyCABD	25
GCA_003885035	B2	USA	33	?:H31	Chromosome	Human	hlyCABD	1
GCA_003885095	B2	USA	22	O6:H31	Chromosome	Human	hlyCABD	1
GCA_003885255	B2	USA	33	?:H31	Chromosome	Human	hlyCABD	1
GCA_003885295	B2	USA	30	O6:H1	Chromosome	Human	hlyCABD	3
GCA_003885615	B1	United Kingdom	26	O142:H8	Chromosome	Human	hlyCABD	25
GCA_003885875	B2	USA	1	O18.Gp12:H7	Chromosome	Human	hlyCABD	7
GCA_003885995	B2	USA	4	?:H1	Chromosome	Human	hlyCABD	3
GCA_003886015	B2	USA	33	O6:H31	Chromosome	Human	hlyCABD	1
GCA_003886045	B2	USA	4	?:H1	Chromosome	Human	hlyCABD	3
GCA_003886105	B2	USA	127	O6:H31	Chromosome	Human	hlyCABD	1
GCA_003886325	D	USA	963	?:H18	Chromosome	Human	hlyCABD	29
GCA_003886375	D	USA	3	O17.O44.O77.Gp9/O17.O77.Gp9:H18	Chromosome	Human	hlyCABD	9
GCA_003886385	B2	USA	32	O6:H31	Chromosome	Human	hlyCABD	1
GCA_003886565	B2	USA	52	O6:H1	Chromosome	Human	hlyCABD	3
GCA_003886615	D	USA	963	O2.Gp7/O2.O50.Gp7:H18	Chromosome	Human	hlyCABD	29
GCA_003887075	F	United Kingdom	59	O1:H7	Chromosome	Human	hlyCABD	29
GCA_003891455	B2	United Kingdom	4	O18.Gp12:H1	Chromosome	Human	hlyCABD	3
GCA_003892485	B2	USA	22	O6:H31	Chromosome	Human	hlyCABD	1
GCA_003892545	B2	USA	4	O6:H1	Chromosome	Human	hlyCABD	3

GCA_003892595	B2	USA	12	O4:H5	Chromosome	Human	hlyCABD	5
GCA_003896655	B2	United Kingdom	73	O6:H1	Chromosome	Unknown	hlyCABD	3
GCA_003897465	B2	United Kingdom	5261	O18.Gp12:H7	Chromosome	Unknown	hlyCABD	28
GCA_003977565	B2	Egypt	6355	O18.Gp12:H5	Chromosome	Unknown	hlyCABD	5
GCA_003977575	B2	Egypt	6355	O18.Gp12:H5	Chromosome	Unknown	hlyCABD	5
GCA_004000325	B1	China	517	O160:H19	Plasmid	Human	hlyCABD	21
GCA_004000355	B1	China	517	O160:H19	Plasmid	Human	hlyCABD	21
GCA_004025255	A	India	2	?:H9	Chromosome	Human	hlyCABD	24
GCA_004026025	A	India	2	?:H9	Chromosome	Human	hlyCABD	24
GCA_004026035	B2	India	43	Onovel31:H4	Chromosome	Human	hlyCABD	6
GCA_004100285	D	Unknown	8	?:H30	Chromosome	Unknown	hlyCABD	29
GCA_004100735	F	USA	2618	O1:H7	Chromosome	Human	hlyCABD	29
GCA_004100835	B2	USA	55	O16:H6	Chromosome	Human	hlyCABD	28
GCA_004100845	B2	USA	12	O4:H5	Chromosome	Human	hlyCABD	5
GCA_004100925	B2	USA	12	O4:H5	Chromosome	Human	hlyCABD	5
GCA_004101085	B2	USA	4	O6:H1	Chromosome	Human	hlyCABD	3
GCA_004151095	B2	Unknown	52	O6:H1	Chromosome	Human	hlyCABD	3
GCA_004153035	B2	Russia	10	O2.Gp7/O2.O50.Gp7:H6	Chromosome	Human	hlyCABD	2
GCA_004160725	B2	United Kingdom	8290	O2.Gp7/O2.O50.Gp7:H6	Chromosome	Human	hlyCABD	2
GCA_004162565	B2	United Kingdom	372	O4:H31	Chromosome	Human	hlyCABD	28
GCA_004162695	B2	United Kingdom	131	Onovel31:H4	Chromosome	Human	hlyCABD	6
GCA_004170775	B2	Australia	372	O6:H31	Chromosome	Pig	hlyCABD	28
GCA_004172245	D	Australia	3	O17.O44.O77.Gp9/O17.O77.Gp9:H18	Chromosome	Pig	hlyCABD	9
GCA_004172675	D	Australia	3	O17.O44.O77.Gp9/O17.O77.Gp9:H18	Chromosome	Pig	hlyCABD	9
GCA_004172805	D	Australia	3	O17.O44.O77.Gp9/O17.O77.Gp9:H18	Chromosome	Pig	hlyCABD	9
GCA_004232845	A	USA	10	?:H2	Undeterimined	Unknown	hlyCABD	24
GCA_004232885	D	USA	3	O15:H6	Chromosome	Unknown	hlyCABD	9
GCA_004262625	B1	United Kingdom	971	?:H25	Chromosome	Human	hlyCABD	22
GCA_004263685	B2	United Kingdom	5261	O18.Gp12:H7	Chromosome	Unknown	hlyCABD	28
GCA_004265585	D	United Kingdom	42	?:H14	Undeterimined	Unknown	hlyCABD	29
GCA_004270825	A	United Kingdom	746	?:H19	Chromosome	Unknown	hlyCABD	23
GCA_004277155	B1	USA	641	O45:H11	Plasmid	Unknown	hlyCABD	25
GCA_004344805	B2	USA	33	O6:H31	Chromosome	Hedgehog	hlyCABD	1
GCA_004404145	B2	India	33	O6:H31	Chromosome	Human	hlyCABD	1
GCA_004566415	B2	USA	43	Onovel31:H4	Chromosome	Human	hlyCABD	6
GCA_004566645	B2	USA	43	?:H4	Chromosome	Human	hlyCABD	6
GCA_004566665	B2	USA	43	Onovel31:H4	Undeterimined	Human	hlyCABD	6
GCA_004566765	B2	USA	43	Onovel31:H4	Chromosome	Human	hlyCABD	6
GCA_004566915	B2	USA	43	Onovel31:H4	Chromosome	Human	hlyCABD	6
GCA_004566965	B2	USA	43	Onovel31:H4	Chromosome	Human	hlyCABD	6
GCA_004567225	B2	USA	43	?:H4	Chromosome	Human	hlyCABD	6
GCA_004567255	B2	USA	43	Onovel31:H4	Chromosome	Human	hlyCABD	6
GCA_004567365	B2	USA	43	?:H4	Chromosome	Human	hlyCABD	6
GCA_004567405	B2	USA	43	Onovel31:H4	Chromosome	Human	hlyCABD	6
GCA_004567415	B2	USA	43	Onovel31:H4	Chromosome	Human	hlyCABD	6
GCA_004567445	B2	USA	1	?:H7	Undeterimined	Human	hlyCABD	7
GCA_004567535	B2	USA	43	Onovel31:H4	Chromosome	Human	hlyCABD	6
GCA_004568225	B2	USA	43	Onovel31:H4	Chromosome	Human	hlyCABD	6
GCA_004568235	B2	USA	43	?:H4	Chromosome	Human	hlyCABD	6
GCA_004568295	B2	USA	131	O16:H5	Chromosome	Human	hlyCABD	6
GCA_004568365	B2	USA	43	Onovel31:H4	Chromosome	Human	hlyCABD	6
GCA_004568555	B2	USA	26	O2.Gp7/O2.O50.Gp7:H6	Chromosome	Human	hlyCABD	2
GCA_004568575	B2	USA	43	Onovel31:NA	Chromosome	Human	hlyCABD	6
GCA_004568605	B2	USA	131	Onovel31:H4	Chromosome	Human	hlyCABD	6
GCA_004568675	B2	USA	43	Onovel31:H4	Chromosome	Human	hlyCABD	6
GCA_004568765	B2	USA	33	O6:H31	Chromosome	Human	hlyCABD	1
GCA_004568795	B2	USA	43	Onovel31:H4	Chromosome	Human	hlyCABD	6
GCA_004568895	B2	USA	43	Onovel31:H4	Chromosome	Human	hlyCABD	6
GCA_004569015	B2	USA	43	Onovel31:H4	Chromosome	Human	hlyCABD	6
GCA_004569075	B2	USA	33	O6:H31	Chromosome	Human	hlyCABD	1
GCA_004569825	B2	USA	43	O2.Gp7/O2.O50.Gp7:H4	Chromosome	Human	hlyCABD	6
GCA_004569905	B2	USA	43	Onovel31:H4	Chromosome	Human	hlyCABD	6
GCA_004569915	B2	USA	43	?:H4	Chromosome	Human	hlyCABD	6
GCA_004766675	A	USA	3546	Onovel2:H38	Plasmid	Pig	hlyCABD	23
GCA_004767855	B1	USA	58	O88:H25	Plasmid	Unknown	hlyCABD	22
GCA_005038395	B1	Canada	2217	O117.Gp8:H16	Plasmid	Cattle	hlyCABD	25
GCA_005041365	B2	Canada	647	O109:H5	Plasmid	Unknown	hlyCABD	7
GCA_005042235	B1	Canada	58	?:H21	Plasmid	Unknown	hlyCABD	22
GCA_005042675	B1	Canada	58	?:H21	Plasmid	Unknown	hlyCABD	22
GCA_005042685	B1	Canada	58	?:H21	Plasmid	Unknown	hlyCABD	22
GCA_005045565	B1	Denmark	1	O139:H1	Plasmid	Unknown	hlyCABD	29
GCA_005045735	A	Denmark	2	O28ab:H9	Chromosome	Unknown	hlyCABD	24
GCA_005146085	B2	Thailand	9	Onovel31:H4	Chromosome	Human	hlyCABD	6
GCA_005382085	B2	Japan	95	O18.Gp12:H7	Chromosome	Human	hlyCABD	7
GCA_005382265	B2	Japan	6	O18.Gp12:H5	Chromosome	Human	hlyCABD	28
GCA_005383165	B2	Japan	1	?:H7	Chromosome	Human	hlyCABD	7
GCA_005383205	B2	Japan	73	?:H1	Chromosome	Human	hlyCABD	3
GCA_005383465	B2	Japan	4	?:H1	Chromosome	Human	hlyCABD	3

GCA_005386965	B2	Japan	73	?:H1	Chromosome	Human	hlyCABD	3
GCA_005387045	B2	Japan	12	?:H5	Chromosome	Human	hlyCABD	5
GCA_005387585	B2	Japan	1	?:H7	Chromosome	Human	hlyCABD	7
GCA_005388925	B2	Japan	12	O4:H5	Chromosome	Human	hlyCABD	5
GCA_005389805	B2	Japan	1	?:H7	Chromosome	Human	hlyCABD	7
GCA_005390025	B1	Japan		?:H2	Plasmid	Cattle	hlyCABD	25
GCA_005391285	B1	Japan	392	?:H2	Plasmid	Cattle	hlyCABD	25
GCA_005391925	B2	Japan	1	O2.Gp7/O2.O50.Gp7:H5	Plasmid	Cattle	hlyCABD	7
GCA_005392305	B2	Japan	1	O2.Gp7/O2.O50.Gp7:H5	Plasmid	Cattle	hlyCABD	7
GCA_005392935	B2	Japan		O109:H5	Plasmid	Cattle	hlyCABD	7
GCA_005393585	B1	Japan	13	?:H8	Plasmid	Cattle	hlyCABD	25
GCA_005393605	B1	Japan	392	?:H2	Plasmid	Cattle	hlyCABD	25
GCA_005393845	B1	Japan	58	?:H21	Plasmid	Cattle	hlyCABD	22
GCA_005394025	B1	Japan	29	O26:H11	Plasmid	Cattle	hlyCABD	27
GCA_005397765	A	USA	10	O129.O13.O135.Gp10/O129.O13.Gp10:H11	Plasmid	Cattle	hlyCABD	24
GCA_005397785	B1	USA	2473	O168:H28	Plasmid	Cattle	hlyCABD	25
GCA_005397865	A	USA	10	O129.O13.O135.Gp10/O129.O13.Gp10:H11	Plasmid	Cattle	hlyCABD	24
GCA_005397885	A	USA	10	O129.O13.O135.Gp10/O129.O13.Gp10:H11	Plasmid	Cattle	hlyCABD	24
GCA_005397905	A	USA	10	O129.O13.O135.Gp10/O129.O13.Gp10:H11	Plasmid	Cattle	hlyCABD	24
GCA_005397925	B1	USA	2473	O168:H28	Plasmid	Cattle	hlyCABD	25
GCA_005397945	A	USA	10	O129.O13.O135.Gp10/O129.O13.Gp10:H11	Plasmid	Cattle	hlyCABD	24
GCA_005397985	B1	USA	2473	O168:H28	Plasmid	Cattle	hlyCABD	25
GCA_005398065	B1	USA	2473	O168:H28	Plasmid	Cattle	hlyCABD	25
GCA_005398125	B1	USA	2473	O168:H28	Plasmid	Cattle	hlyCABD	25
GCA_005398205	B1	USA	2473	O168:H28	Plasmid	Cattle	hlyCABD	25
GCA_005398285	A	USA	10	O129.O13.O135.Gp10/O129.O13.Gp10:H11	Plasmid	Cattle	hlyCABD	24
GCA_005398425	B1	USA	2473	O168:H28	Plasmid	Cattle	hlyCABD	25
GCA_005398625	B1	USA	2473	O168:H28	Plasmid	Cattle	hlyCABD	25
GCA_005398705	B1	USA	2473	O168:H28	Plasmid	Cattle	hlyCABD	25
GCA_005398745	A	USA	10	O129.O13.O135.Gp10/O129.O13.Gp10:H11	Plasmid	Cattle	hlyCABD	24
GCA_005398805	B1	USA	58	?:H21	Plasmid	Cattle	hlyCABD	22
GCA_005398845	B1	USA	58	O109:H21	Undeterimined	Cattle	hlyCABD	22
GCA_005398865	B1	USA	2473	O168:H28	Plasmid	Cattle	hlyCABD	25
GCA_005398885	A	USA	10	O129.O13.O135.Gp10/O129.O13.Gp10:H11	Plasmid	Cattle	hlyCABD	24
GCA_005398905	A	USA	10	O129.O13.O135.Gp10/O129.O13.Gp10:H11	Plasmid	Cattle	hlyCABD	24
GCA_005398965	B1	France	29	O26:NA	Plasmid	Cattle	hlyCABD	27
GCA_005399085	B1	France	1172	O88:H16	Plasmid	Cattle	hlyCABD	25
GCA_005399425	D	France	132	O149:H1	Undeterimined	Cattle	hlyCABD	29
GCA_005399485	B1	France	29	O26:NA	Plasmid	Cattle	hlyCABD	27
GCA_005399525	B1	France	392	?:H2	Plasmid	Cattle	hlyCABD	25
GCA_005399585	B1	France	58	O58:H40	Plasmid	Cattle	hlyCABD	22
GCA_005399605	B1	France	58	O58:H40	Plasmid	Cattle	hlyCABD	22
GCA_005399865	B1	France	442	O146:H21	Plasmid	Cattle	hlyCABD	17
GCA_005399965	B1	France	29	O26:H11	Plasmid	Cattle	hlyCABD	27
GCA_005400165	B1	France	58	O88:H25	Plasmid	Cattle	hlyCABD	22
GCA_005502265	A	China	9992	O3:H45	Plasmid	Pig	hlyCABD	23
GCA_006229885	D	Russia	8	O86:H18	Chromosome	Human	hlyCABD	29
GCA_006230505	B2	Russia	43	?:NA	Chromosome	Human	hlyCABD	6
GCA_006230715	B2	Russia	43	?:H4	Chromosome	Human	hlyCABD	6
GCA_006231415	B2	Estonia	658	O2.Gp7/O2.O50.Gp7:H14	Chromosome	Human	hlyCABD	28
GCA_006231545	B2	Estonia	658	O2.Gp7/O2.O50.Gp7:H14	Chromosome	Human	hlyCABD	28
GCA_006231595	B2	Estonia	658	O2.Gp7/O2.O50.Gp7:H14	Chromosome	Human	hlyCABD	28
GCA_006231615	B2	Estonia	658	O2.Gp7/O2.O50.Gp7:H14	Chromosome	Human	hlyCABD	28
GCA_006231955	B2	Estonia	658	O2.Gp7/O2.O50.Gp7:H14	Chromosome	Human	hlyCABD	28
GCA_006232935	B2	Russia	43	Onovel31:H4	Chromosome	Human	hlyCABD	6
GCA_006232955	D	Norway	8	O153var1:H30	Chromosome	Human	hlyCABD	29
GCA_006233135	B2	Norway	12	O4:H1	Chromosome	Human	hlyCABD	5
GCA_006233495	B2	Norway	43	Onovel31:H4	Chromosome	Human	hlyCABD	6
GCA_006233905	B2	Norway	43	Onovel31:H4	Chromosome	Human	hlyCABD	6
GCA_006233955	B2	Norway	43	Onovel31:H4	Chromosome	Human	hlyCABD	6
GCA_006235335	B2	Latvia	10	O2.Gp7/O2.O50.Gp7:H6	Chromosome	Human	hlyCABD	2
GCA_006235395	B2	Latvia	10	O2.Gp7/O2.O50.Gp7:H6	Chromosome	Human	hlyCABD	2
GCA_006235975	B2	Lithuania	12	O4:H5	Chromosome	Human	hlyCABD	5
GCA_006236105	B2	Lithuania	9	Onovel31:H4	Chromosome	Human	hlyCABD	6
GCA_006236265	B2	Lithuania	1	O18.Gp12:H7	Chromosome	Human	hlyCABD	7
GCA_006236705	B2	Latvia	10	O2.Gp7/O2.O50.Gp7:H6	Chromosome	Human	hlyCABD	2
GCA_006237085	B2	Lithuania	9	Onovel31:H4	Chromosome	Human	hlyCABD	6
GCA_006237565	B2	Estonia	658	O2.Gp7/O2.O50.Gp7:H14	Chromosome	Human	hlyCABD	28
GCA_006237735	B2	Estonia	658	O2.Gp7/O2.O50.Gp7:H14	Chromosome	Human	hlyCABD	28
GCA_006237995	B2	Estonia	80	O75:H7	Chromosome	Human	hlyCABD	28
GCA_006238045	B2	Estonia	658	O2.Gp7/O2.O50.Gp7:H14	Chromosome	Human	hlyCABD	28
GCA_006238155	B2	Estonia	658	?:H14	Chromosome	Human	hlyCABD	28
GCA_006238495	B2	Estonia	2015	O2.Gp7/O2.O50.Gp7:H14	Chromosome	Human	hlyCABD	28
GCA_006239035	B2	Estonia	658	O2.Gp7/O2.O50.Gp7:H14	Chromosome	Human	hlyCABD	28
GCA_007644235	B1	Brazil	29	O2var1:H9	Undeterimined	Human	hlyCABD	27
GCA_007646545	A	Brazil	10	O129.O13.O135.Gp10/O129.O13.Gp10:H11	Undeterimined	Human	hlyCABD	24
GCA_007646625	B1	Brazil	517	?:H19	Plasmid	Human	hlyCABD	21
GCA_007646825	B1	Brazil	381	O177:H9	Undeterimined	Human	hlyCABD	25

GCA_007647335	B1	Brazil	517	O126:H19	Plasmid	Human	hlyCABD	21
GCA_007647475	B1	Brazil	517	O126:H19	Plasmid	Human	hlyCABD	21
GCA_007648065	B1	Brazil	483	?:H12	Undeterimined	Human	hlyCABD	25
GCA_007648705	B1	Brazil	381	O39:H9	Undeterimined	Human	hlyCABD	25
GCA_007648715	B1	Brazil	381	O39:H9	Undeterimined	Human	hlyCABD	25
GCA_007648925	B1	Brazil	29	O26:H11	Plasmid	Human	hlyCABD	27
GCA_007648955	B1	Brazil	29	O26:H11	Plasmid	Human	hlyCABD	27
GCA_007844295	B2	China	52	O6:H1	Chromosome	Human	hlyCABD	3
GCA_007844345	B2	China	52	O6:H1	Chromosome	Human	hlyCABD	3
GCA_007844355	B2	China	52	O6:H1	Chromosome	Human	hlyCABD	3
GCA_007844635	B2	China	4702	O4:H5	Chromosome	Human	hlyCABD	5
GCA_007844645	B2	China	4702	O4:H5	Chromosome	Human	hlyCABD	5
GCA_007844685	B2	China	4702	O4:H5	Chromosome	Human	hlyCABD	5
GCA_007844755	B2	China		O6:H31	Chromosome	Human	hlyCABD	1
GCA_007844775	B2	China	32	O6:H31	Chromosome	Human	hlyCABD	1
GCA_007844805	B2	China	32	O6:H31	Chromosome	Human	hlyCABD	1
GCA_008040725	D	Russia	3	O15:H18	Chromosome	Human	hlyCABD	9
GCA_008040815	B2	Russia	70	O1:H7	Chromosome	Human	hlyCABD	7
GCA_008041115	A	Russia	2	O28ab:H9	Chromosome	Human	hlyCABD	24
GCA_008041535	B2	Russia	52	O6:H1	Chromosome	Human	hlyCABD	3
GCA_008081785	B2	USA	73	O22:H1	Chromosome	Human	hlyCABD	3
GCA_008082155	B2	USA	12	O4:H5	Chromosome	Human	hlyCABD	5
GCA_008272075	B2	Canada	43	Onovel31:H4	Chromosome	Unknown	hlyCABD	6
GCA_008272515	B2	Canada	43	Onovel31:H4	Chromosome	Unknown	hlyCABD	6
GCA_008272795	B2	Canada	43	Onovel31:H4	Chromosome	Unknown	hlyCABD	6
GCA_008273915	D	Canada		?:H14	Plasmid	Cattle	hlyCABD	29
GCA_008274005	B2	Canada	95	O2.Gp7/O2.O50.Gp7:H5	Plasmid	Cattle	hlyCABD	7
GCA_008385225	B2	Australia	4	O18.Gp12:H1	Chromosome	Human	hlyCABD	3
GCA_008385545	B2	Australia	4	O2.Gp7/O2.O50.Gp7:H1	Chromosome	Human	hlyCABD	3
GCA_008388695	B2	Australia	4	O6:H1	Chromosome	Human	hlyCABD	3
GCA_008553695	B2	Unknown	1	O18.Gp12:H7	Chromosome	Human	hlyCABD	7
GCA_008553755	B2	Unknown	73	?:H1	Chromosome	Human	hlyCABD	3
GCA_008930675	B2	USA	12	O4:H5	Chromosome	Human	hlyCABD	5
GCA_009361745	B2	Canada	29	O6:H1	Chromosome	Unknown	hlyCABD	3
GCA_009361875	B2	Canada	1	O18.Gp12:H7	Chromosome	Unknown	hlyCABD	7
GCA_009361915	B2	Canada	43	Onovel31:H4	Chromosome	Unknown	hlyCABD	6
GCA_009361925	B2	Canada	43	Onovel31:H4	Chromosome	Unknown	hlyCABD	6
GCA_009361935	B2	Canada	80	O2.Gp7/O2.O50.Gp7:H7	Chromosome	Unknown	hlyCABD	28
GCA_009361975	B2	Canada	43	Onovel31:H4	Chromosome	Unknown	hlyCABD	6
GCA_009362015	B2	Canada	1	O18.Gp12:H7	Chromosome	Unknown	hlyCABD	7
GCA_009362035	B2	Canada	509	O6:H7	Chromosome	Unknown	hlyCABD	28
GCA_009362065	B2	Canada	43	Onovel31:H4	Chromosome	Unknown	hlyCABD	6
GCA_009362115	B2	Canada	43	Onovel31:H4	Chromosome	Unknown	hlyCABD	6
GCA_009362145	B2	Canada	22	O6:H31	Chromosome	Unknown	hlyCABD	1
GCA_009890645	B2	USA	12	O4:H5	Chromosome	Human	hlyCABD	5
GCA_009903585	C	China	66	O180:H11	Plasmid	Bird	hlyCABD	16
GCA_009903595	D	China	1	O139:H1	Plasmid	Bird	hlyCABD	29
GCA_900000205	B1	Unknown	29	O26:H11	Plasmid	Unknown	hlyCABD	27
GCA_900196475	D	Australia	1	O139:H1	Plasmid	Pig	hlyCABD	29
GCA_900448115	F	United Kingdom	62	O7:H45	Chromosome	Human	hlyCABD	29
GCA_900448225	B2	Unknown	12	?:H5	Chromosome	Unknown	hlyCABD	5
GCA_900448325	B2	Unknown	40	O83:H31	Chromosome	Unknown	hlyCABD	28
GCA_900448375	F	Unknown	59	O1:H7	Chromosome	Unknown	hlyCABD	29
GCA_900448395	D	USA	42	O138:H14	Plasmid	Unknown	hlyCABD	29
GCA_900448425	B2	Unknown	52	O6:H1	Chromosome	Unknown	hlyCABD	3
GCA_900448455	A	Unknown	52	Onovel31:H14	Undeterimined	Unknown	hlyCABD	23
GCA_900448555	B2	Unknown	73	O2.Gp7/O2.O50.Gp7:H1	Chromosome	Unknown	hlyCABD	3
GCA_900448815	A	Unknown	10	O166:H4	Chromosome	Unknown	hlyCABD	24
GCA_900448985	D	Unknown	72	O50.Gp7/O2.O50.Gp7:H4	Chromosome	Unknown	hlyCABD	29
GCA_900448995	B2	Unknown	29	O6:H1	Chromosome	Unknown	hlyCABD	3
GCA_900449035	A	Unknown	34	O62.Gp14:H30	Chromosome	Unknown	hlyCABD	24
GCA_900449135	A	Unknown	10	O3:H2	Chromosome	Unknown	hlyCABD	24
GCA_900449195	A	Unknown	10	O30:H4	Chromosome	Unknown	hlyCABD	24
GCA_900449245	A	Unknown	10	O30:H4	Chromosome	Unknown	hlyCABD	24
GCA_900449335	B2	Unknown	12	O4:H5	Chromosome	Unknown	hlyCABD	5
GCA_900449475	D	Unknown	44	O2.Gp7/O2.O50.Gp7:H4	Chromosome	Human	hlyCABD	29
GCA_900449485	B1	United Kingdom	29	O26:H11	Plasmid	Unknown	hlyCABD	27
GCA_900449525	A	Unknown	34	?:H33	Plasmid	Unknown	hlyCABD	24
GCA_900449575	B1	Unknown	278	O91:H10	Chromosome	Unknown	hlyCABD	25
GCA_900449615	A	Unknown		O45:H10	Chromosome	Unknown	hlyCABD	24
GCA_900449925	B2	Unknown		O75:H5	Chromosome	Unknown	hlyCABD	28
GCA_900450025	B2	Unknown	5261	O18.Gp12:H7	Chromosome	Human	hlyCABD	28
GCA_900450095	B1	Unknown	29	O26:H11	Plasmid	Unknown	hlyCABD	27
GCA_900450115	A	Unknown	10	O123.O186.Gp5/O123.Gp5:H16	Plasmid	Unknown	hlyCABD	24
GCA_900450125	A	Unknown	2	O71:H12	Chromosome	Unknown	hlyCABD	24
GCA_900450305	A	Unknown	2	O28ab:H9	Chromosome	Unknown	hlyCABD	24
GCA_900450405	D	Unknown	1	O139:H1	Plasmid	Unknown	hlyCABD	29
GCA_900478465	A	United Kingdom	5749	O98:H7	Plasmid	Pig	hlyCABD	23

GCA_900478525	A	United Kingdom	5755	O36:H19	Plasmid	Pig	hlyCABD	24
GCA_900478845	A	United Kingdom	10	O2.Gp7/O2.O50.Gp7:H32	Plasmid	Pig	hlyCABD	24
GCA_900478925	A	United Kingdom	10	O2.Gp7/O2.O50.Gp7:H32	Plasmid	Pig	hlyCABD	24
GCA_900478965	B1	United Kingdom	303	O150:H8	Plasmid	Cattle	hlyCABD	25
GCA_900478975	B1	United Kingdom	303	O150:H8	Plasmid	Cattle	hlyCABD	25
GCA_900479115	B1	United Kingdom	58	O49:H30	Plasmid	Cattle	hlyCABD	22
GCA_900479155	D	United Kingdom	42	O138:H14	Plasmid	Pig	hlyCABD	29
GCA_900479205	D	United Kingdom	42	O138:H14	Plasmid	Pig	hlyCABD	29
GCA_900479235	C	United Kingdom	90	O157:H19	Plasmid	Pig	hlyCABD	16
GCA_900479345	D	United Kingdom	42	O138:H14	Plasmid	Pig	hlyCABD	29
GCA_900479375	D	United Kingdom	42	O138:H14	Plasmid	Pig	hlyCABD	29
GCA_900479385	A	United Kingdom	10	O71:H25	Plasmid	Cattle	hlyCABD	24
GCA_900479465	B1	United Kingdom	186	O88:H21	Plasmid	Cattle	hlyCABD	22
GCA_900479545	D	United Kingdom	42	?:H14	Plasmid	Pig	hlyCABD	29
GCA_900479875	C	United Kingdom	90	O157:H19	Plasmid	Pig	hlyCABD	16
GCA_900479925	C	United Kingdom	90	O157:H19	Plasmid	Pig	hlyCABD	16
GCA_900480195	A	United Kingdom	5749	O98:H7	Plasmid	Pig	hlyCABD	23
GCA_900480365	B1	United Kingdom	58	O88:H25	Plasmid	Cattle	hlyCABD	22
GCA_900480565	B1	United Kingdom	58	O88:H25	Plasmid	Cattle	hlyCABD	22
GCA_900480595	B1	United Kingdom	58	O88:H25	Plasmid	Cattle	hlyCABD	22
GCA_900482495	C	United Kingdom	66	?:H12	Chromosome	Pig	hlyCABD	16
GCA_900490205	A	United Kingdom	1112	O142:H27	Plasmid	Pig	hlyCABD	23
GCA_900499215	B2	France	4	O6:H1	Chromosome	Unknown	hlyCABD	3
GCA_900499415	B2	France	73	O22:H1	Chromosome	Unknown	hlyCABD	3
GCA_900499475	B2	France	4	O6:H1	Chromosome	Unknown	hlyCABD	3
GCA_900499485	B2	France	43	Onovel31:H4	Chromosome	Unknown	hlyCABD	6
GCA_900499545	B2	France	131	O16:H5	Chromosome	Unknown	hlyCABD	6
GCA_900499555	D	France	3	O45:H16	Chromosome	Unknown	hlyCABD	9
GCA_900499605	B2	France	882	O4:H5	Chromosome	Unknown	hlyCABD	5
GCA_900499615	B2	France	1	O18.Gp12:H7	Chromosome	Unknown	hlyCABD	7
GCA_900499635	B2	France	6	O18.Gp12:H5	Chromosome	Unknown	hlyCABD	28
GCA_900499645	B2	France	32	O6:H31	Chromosome	Unknown	hlyCABD	1
GCA_900499735	B2	France	70	O1:H7	Chromosome	Unknown	hlyCABD	7
GCA_900499765	B2	France	4	O6:H1	Chromosome	Unknown	hlyCABD	3
GCA_900499815	B2	France	4	O6:H1	Chromosome	Unknown	hlyCABD	3
GCA_900499845	C	France	410	?:H27	Chromosome	Unknown	hlyCABD	16
GCA_900499965	B2	France	4	O6:H1	Chromosome	Unknown	hlyCABD	3
GCA_900499985	B2	France	217	O85:H1	Chromosome	Unknown	hlyCABD	28
GCA_900499995	B2	France	55	O16:H6	Chromosome	Unknown	hlyCABD	28
GCA_900500005	B2	France	884	O75:H5	Chromosome	Unknown	hlyCABD	28
GCA_900500075	D	France	3	O15:H18	Chromosome	Unknown	hlyCABD	9
GCA_900500095	C	France	88	O179:H19	Chromosome	Unknown	hlyCABD	16
GCA_900500115	D	France	3	O15:H18	Chromosome	Unknown	hlyCABD	9
GCA_900500135	B2	France	12	O4:H5	Chromosome	Unknown	hlyCABD	5
GCA_900500155	B2	France	10	O2.Gp7/O2.O50.Gp7:H6	Chromosome	Unknown	hlyCABD	2
GCA_900500195	B2	France	131	O16:H5	Chromosome	Unknown	hlyCABD	6
GCA_900500205	D	France	3	O15:H18	Chromosome	Unknown	hlyCABD	9
GCA_900500335	B2	France	6	O18.Gp12:H5	Chromosome	Unknown	hlyCABD	28
GCA_900500345	B2	France	4	O6:H1	Chromosome	Unknown	hlyCABD	3
GCA_900500355	B2	France	4	O18.Gp12:H1	Chromosome	Unknown	hlyCABD	3
GCA_900500375	B2	France	12	O4:H5	Chromosome	Unknown	hlyCABD	5
GCA_900500415	B2	France	4	O18.Gp12:H1	Chromosome	Unknown	hlyCABD	3
GCA_900500455	B2	France	6	O18.Gp12:H5	Chromosome	Unknown	hlyCABD	28
GCA_900500475	B2	France	658	O2.Gp7/O2.O50.Gp7:H14	Chromosome	Unknown	hlyCABD	28
GCA_900500515	B2	France	4	O18.Gp12:H1	Chromosome	Unknown	hlyCABD	3
GCA_900500555	D	France	3	O15:H18	Chromosome	Unknown	hlyCABD	9
GCA_900500595	D	France	3	O15:H18	Chromosome	Unknown	hlyCABD	9
GCA_900536915	B2	Australia	52	O6:H1	Chromosome	Human	hlyCABD	3
GCA_900536975	B2	Australia	4	Onovel31/O25:H1	Chromosome	Human	hlyCABD	3
GCA_901482675	D	United Kingdom	969	O160:H26	Plasmid	Human	hlyCABD	23
GCA_901485085	B2	Unknown	131	Onovel31:H4	Chromosome	Unknown	hlyCABD	6
GCA_901485095	B2	Unknown	43	Onovel31:H4	Chromosome	Unknown	hlyCABD	6
GCA_901485145	B2	Unknown	131	Onovel31:H4	Chromosome	Unknown	hlyCABD	6
GCA_901485185	B2	Unknown	131	Onovel31:H4	Chromosome	Unknown	hlyCABD	6
GCA_901485195	B2	Unknown		Onovel31:H4	Chromosome	Unknown	hlyCABD	6
GCA_901669775	B2	Unknown		Onovel31:H4	Chromosome	Unknown	hlyCABD	6
GCA_901669885	B2	Unknown	43	Onovel31:H4	Chromosome	Unknown	hlyCABD	6
GCA_902160895	B2	United Kingdom	1	O18.Gp12:H7	Chromosome	Human	hlyCABD	7
GCA_902161765	B2	United Kingdom	22	O6:H31	Chromosome	Human	hlyCABD	1
GCA_902161775	B2	United Kingdom	22	O6:H31	Chromosome	Human	hlyCABD	1
GCA_902387885	F	Unknown	967	O36:H42	Plasmid	Unknown	hlyCABD	29
GCA_902668605	B2	United Kingdom	43	Onovel31:H4	Chromosome	Human	hlyCABD	6
GCA_902668615	B2	United Kingdom	43	Onovel31:H4	Chromosome	Human	hlyCABD	6
GCA_902668665	B2	United Kingdom	43	Onovel31:H4	Chromosome	Human	hlyCABD	6
GCA_902668675	B2	United Kingdom	43	Onovel31:H4	Chromosome	Human	hlyCABD	6
GCA_902706715	D	France	3	O15:H18	Chromosome	Human	hlyCABD	9
GCA_902706765	B2	France	537	?:H5	Chromosome	Human	hlyCABD	28
GCA_902706895	B2	France	32	O6:H31	Chromosome	Human	hlyCABD	1

GCA_902707075	B2	France	12	O21:H5	Chromosome	Human	hlyCABD	5
GCA_902707145	B2	France	52	O6:H1	Chromosome	Human	hlyCABD	3
GCA_902707205	A	France	10	O148:H32	Undeterimined	Human	hlyCABD	24
GCA_902707235	B2	France	33	O6:H31	Chromosome	Human	hlyCABD	1
GCA_902707275	B2	France	32	O6:H31	Chromosome	Human	hlyCABD	1
GCA_902707365	D	France	3	O15:H18	Chromosome	Human	hlyCABD	9
GCA_902707385	B2	France	12	O4:H5	Chromosome	Human	hlyCABD	5
GCA_902707425	B2	France	4	O6:H1	Chromosome	Human	hlyCABD	3
GCA_902707445	B2	France	26	O2.Gp7/O2.O50.Gp7:H6	Chromosome	Human	hlyCABD	2
GCA_902707455	B2	France	4	O2.Gp7/O2.O50.Gp7:H1	Chromosome	Human	hlyCABD	3
GCA_902707515	D	France	3	O15:H18	Chromosome	Human	hlyCABD	9
GCA_902707525	B1	France	388	Onovel31/O25:H2	Plasmid	Human	hlyCABD	25
GCA_902707675	B2	France	4	O6:H1	Chromosome	Human	hlyCABD	3
GCA_902707875	B2	France	4	O6:H1	Chromosome	Human	hlyCABD	3
GCA_902707905	B2	France	4	O6:H1	Chromosome	Human	hlyCABD	3
GCA_902707925	B2	France	10	O2.Gp7/O2.O50.Gp7:H6	Chromosome	Human	hlyCABD	2
GCA_902707945	B2	France	4	O6:H1	Chromosome	Human	hlyCABD	3
GCA_902707995	C	France	410	O8:H9	Chromosome	Human	hlyCABD	16
GCA_902708025	B2	France	43	Onovel31:H4	Chromosome	Human	hlyCABD	6
GCA_902708065	B2	France	43	Onovel31:H4	Chromosome	Human	hlyCABD	6
GCA_902708095	B2	France	12	O4:H1	Chromosome	Human	hlyCABD	5
GCA_902708105	B2	France	12	O4:H1	Chromosome	Human	hlyCABD	5
GCA_902708125	B2	France	4	O18.Gp12:H1	Chromosome	Human	hlyCABD	3
GCA_902708165	F	France	59	O1:H7	Chromosome	Human	hlyCABD	29
GCA_902708285	B2	France	73	O22:H1	Chromosome	Human	hlyCABD	3
GCA_902708295	D	France	3	O15:H18	Chromosome	Human	hlyCABD	9
GCA_902708365	B2	France	55	O16:H6	Chromosome	Human	hlyCABD	28
GCA_902708385	B2	France	28	O6:H1	Chromosome	Human	hlyCABD	3
GCA_902708405	B2	France	27	O2.Gp7/O2.O50.Gp7:H6	Chromosome	Human	hlyCABD	2
GCA_902708425	B2	France	33	O6:H31	Chromosome	Human	hlyCABD	1
GCA_902708475	B2	France	73	O22:H1	Chromosome	Human	hlyCABD	3
GCA_902708535	B2	France	43	Onovel31:H4	Chromosome	Human	hlyCABD	6
GCA_902708675	B2	France	4	O2.Gp7/O2.O50.Gp7:H1	Chromosome	Human	hlyCABD	3
GCA_902708685	B2	France	4	O2.Gp7/O2.O50.Gp7:H1	Chromosome	Human	hlyCABD	3
GCA_902708695	B2	France	4	O6:H1	Chromosome	Human	hlyCABD	3
GCA_902708715	B2	France	22	O6:H31	Chromosome	Human	hlyCABD	1
GCA_902708735	B2	France	12	O4:H5	Chromosome	Human	hlyCABD	5
GCA_902708745	B2	France	4	O2.Gp7/O2.O50.Gp7:H1	Chromosome	Human	hlyCABD	3
GCA_902708815	B2	France	43	Onovel31:H4	Chromosome	Human	hlyCABD	6
GCA_902708825	B2	France	12	O4:H1	Chromosome	Human	hlyCABD	5
GCA_902708835	B2	France	372	O153var1:H31	Chromosome	Human	hlyCABD	28
GCA_902708855	B2	France	12	O18.Gp12:H5	Chromosome	Human	hlyCABD	5
GCA_902708915	B2	France	4	O6:H1	Chromosome	Human	hlyCABD	3
GCA_902709055	B2	France	4	O22:H1	Chromosome	Human	hlyCABD	3
GCA_902709085	B2	France	43	Onovel31:H4	Chromosome	Human	hlyCABD	6
GCA_902709115	B2	France	10	O2.Gp7/O2.O50.Gp7:H6	Chromosome	Human	hlyCABD	2
GCA_902709125	D	France	3	O15:H18	Chromosome	Human	hlyCABD	9
GCA_902709165	B2	France	52	O6:H1	Chromosome	Human	hlyCABD	3
GCA_902709205	B2	France	43	Onovel31:H4	Chromosome	Human	hlyCABD	6
GCA_902709235	B2	France	537	O75:H5	Chromosome	Human	hlyCABD	28
GCA_902709305	B2	France	131	O16:H5	Chromosome	Human	hlyCABD	6
GCA_902709355	B2	France	131	O16:H5	Chromosome	Human	hlyCABD	6
GCA_902709495	D	France	3	O15:H18	Chromosome	Human	hlyCABD	9
GCA_902709525	B2	France	6	O18.Gp12:H5	Chromosome	Human	hlyCABD	28
GCA_902709605	B2	France	43	Onovel31:H4	Chromosome	Human	hlyCABD	6
GCA_902709635	B2	France	40	O83:H31	Chromosome	Human	hlyCABD	28
GCA_902709705	B2	France	4	O6:H1	Chromosome	Human	hlyCABD	3
GCA_902709715	A	France	10	O3:H2	Chromosome	Human	hlyCABD	24
GCA_902709905	B2	France	43	Onovel31:H4	Chromosome	Human	hlyCABD	6
GCA_902710015	B2	France	43	Onovel31:H4	Chromosome	Human	hlyCABD	6
GCA_902710045	B2	France	12	O4:H5	Chromosome	Human	hlyCABD	5
GCA_902710085	B2	France	10	O2.Gp7/O2.O50.Gp7:H6	Chromosome	Human	hlyCABD	2
GCA_902710105	B2	France	4	O6:H1	Chromosome	Human	hlyCABD	3
GCA_902710135	B2	France	12	O4:H5	Chromosome	Human	hlyCABD	5
GCA_902710255	B2	France	537	O75:H5	Chromosome	Human	hlyCABD	28
GCA_902710285	B2	France	4	O2.Gp7/O2.O50.Gp7:H1	Chromosome	Human	hlyCABD	3
GCA_902710355	B2	France	6	O18.Gp12:H5	Chromosome	Human	hlyCABD	28
GCA_902710405	B2	France	43	Onovel31:H4	Chromosome	Human	hlyCABD	6
GCA_902710415	B2	France	4	O6:H1	Chromosome	Human	hlyCABD	3
GCA_902710455	B2	France	28	O6:H1	Chromosome	Human	hlyCABD	3
GCA_902710565	B2	France	52	O6:H1	Chromosome	Human	hlyCABD	3
GCA_902710575	B2	France	131	Onovel31:H4	Chromosome	Human	hlyCABD	6
GCA_902710625	B2	France	4	O2.Gp7/O2.O50.Gp7:H1	Chromosome	Human	hlyCABD	3
GCA_902710635	B2	France	4	O2.Gp7/O2.O50.Gp7:H1	Chromosome	Human	hlyCABD	3
GCA_902710695	B2	France	129	O2.Gp7/O2.O50.Gp7:H6	Chromosome	Human	hlyCABD	2
GCA_902710705	B2	France	129	O2.Gp7/O2.O50.Gp7:H6	Chromosome	Human	hlyCABD	2
GCA_902710725	B2	France	12	O4:H1	Chromosome	Human	hlyCABD	5
GCA_902710795	B2	France	4	O2.Gp7/O2.O50.Gp7:H1	Chromosome	Human	hlyCABD	3

GCA_902710805	B2	France	55	O16:H6	Chromosome	Human	hlyCABD	28
GCA_902710935	B2	France	70	O1:H7	Chromosome	Human	hlyCABD	7
GCA_902710965	B2	France	10	O2.Gp7/O2.O50.Gp7:H6	Chromosome	Human	hlyCABD	2
GCA_902711005	B2	France	12	O4:H5	Chromosome	Human	hlyCABD	5
GCA_902711015	B2	France	4	O6:H1	Chromosome	Human	hlyCABD	3
GCA_902711035	B2	France	43	Onovel31:H4	Chromosome	Human	hlyCABD	6
GCA_902711125	D	France	3	O15:H18	Chromosome	Human	hlyCABD	9
GCA_902711145	B2	France	73	O6:H1	Chromosome	Human	hlyCABD	3
GCA_902711155	B2	France	43	Onovel31:H4	Chromosome	Human	hlyCABD	6
GCA_902711185	B2	France	73	O6:H1	Chromosome	Human	hlyCABD	3
GCA_902711205	B2	France	4	O6:H1	Chromosome	Human	hlyCABD	3
GCA_902711225	B2	France	141	O2.Gp7/O2.O50.Gp7:H6	Chromosome	Human	hlyCABD	2
GCA_902711235	B2	France	52	O6:H1	Chromosome	Human	hlyCABD	3
GCA_902711295	B2	France	12	O4:H5	Chromosome	Human	hlyCABD	5
GCA_902711365	B2	France	12	O4:H1	Chromosome	Human	hlyCABD	5
GCA_902711425	B2	France	4	O18.Gp12:H1	Chromosome	Human	hlyCABD	3
GCA_902711485	B2	France	4	O22:H1	Chromosome	Human	hlyCABD	3
GCA_902711605	B2	France	4	?:H1	Chromosome	Human	hlyCABD	3
GCA_902711615	D	France	3	O15:H18	Chromosome	Human	hlyCABD	9
GCA_902711635	B2	France	4	O6:H1	Chromosome	Human	hlyCABD	3
GCA_902711745	B2	France	52	O6:H1	Chromosome	Human	hlyCABD	3
GCA_902711755	B2	France	73	O6:H1	Chromosome	Human	hlyCABD	3
GCA_902711785	B2	France	32	O6:H31	Chromosome	Human	hlyCABD	1
GCA_902711815	B2	France	4	O6:H1	Chromosome	Human	hlyCABD	3
GCA_902711825	D	France	3	O15:H18	Chromosome	Human	hlyCABD	9
GCA_902711875	B2	France	10	O2.Gp7/O2.O50.Gp7:H6	Chromosome	Human	hlyCABD	2
GCA_902711885	B2	France	73	O6:H1	Chromosome	Human	hlyCABD	3
GCA_902712015	B2	France	4	O2.Gp7/O2.O50.Gp7:H1	Chromosome	Human	hlyCABD	3
GCA_902712025	B2	France	4	O2.Gp7/O2.O50.Gp7:H1	Chromosome	Human	hlyCABD	3
GCA_902712035	B1	France	302	O139:H19	Plasmid	Human	hlyCABD	25
GCA_902712075	B2	France	73	O6:H1	Chromosome	Human	hlyCABD	3
GCA_001609855	B1	Netherlands	21	O26:H11	Plasmid	Human	ehxCABD	27
GCA_005392065	E	Japan	11	O157:H7	Plasmid	Cattle	ehxCABD	20
GCA_005398785	A	USA	325	O15:H16	Plasmid	Cattle	ehxCABD	23
GCA_008756745	B1	USA	17	O103:H2	Not determined	Human	ehxCABD	15
GCA_001616615	B1	China	278	Onovel30:H7	Not determined	Sheep	ehxCABD	25
GCA_003919135	B1	USA	21	?:H16	Plasmid	Unknown	ehxCABD	27
GCA_002475965	E	USA	11	O157:H7	Plasmid	Unknown	ehxCABD	20
GCA_004797265	D	USA	32	?:H28	Plasmid	Cattle	ehxCABD	8
GCA_000462665	E	Unknown	11	O157:H7	Plasmid	Unknown	ehxCABD	20
GCA_000622755	B1	USA	29	O26:H11	Plasmid	Unknown	ehxCABD	27
GCA_005383945	B1	Japan	205	?:H19	Plasmid	Cattle	ehxCABD	25
GCA_003362015	E	Canada	11	O157:H7	Plasmid	Human	ehxCABD	20
GCA_001012095	B1	USA	679	O163:H19	Plasmid	Unknown	ehxCABD	10
GCA_002515075	D	USA	32	?:H28	Plasmid	Human	ehxCABD	8
GCA_004230545	B1	USA	21	?:H16	Not determined	Unknown	ehxCABD	27
GCA_000462325	E	Unknown	11	O157:H7	Plasmid	Unknown	ehxCABD	20
GCA_003787825	E	USA	11	O157:H7	Plasmid	Unknown	ehxCABD	20
GCA_004231925	D	USA	32	?:H28	Plasmid	Unknown	ehxCABD	8
GCA_002245055	C	USA	74	O78:H4	Not determined	Unknown	ehxCABD	16
GCA_000965555	A	Norway	342	O177:H25	Plasmid	Human	ehxCABD	26
GCA_001563715	B1	USA	16	O111:H8	Plasmid	Environment	ehxCABD	27
GCA_000617545	B1	USA	16	O111:H8	Not determined	Unknown	ehxCABD	27
GCA_003359635	E	Canada	11	O157:H7	Plasmid	Unknown	ehxCABD	20
GCA_001191025	B1	USA	16	O111:H8	Not determined	Human	ehxCABD	27
GCA_002531185	B1	Canada	21	O111:H11	Plasmid	Unknown	ehxCABD	27
GCA_008633805	D	Belgium	32	O145:H28	Plasmid	Human	ehxCABD	8
GCA_002164125	E	USA	11	O157:H7	Plasmid	Cattle	ehxCABD	20
GCA_001607255	B1	Netherlands	21	O26:H11	Plasmid	Human	ehxCABD	27
GCA_003922775	E	USA	11	O157:H7	Plasmid	Unknown	ehxCABD	20
GCA_001607795	E	Netherlands	724	Onovel3:H20	Plasmid	Human	ehxCABD	11
GCA_004231905	E	USA	11	O157:H7	Plasmid	Unknown	ehxCABD	20
GCA_003761485	B1	USA	5487	O28ac.O42.Gp2/O42.Gp2:H25	Plasmid	Unknown	ehxCABD	22
GCA_003756685	B1	USA	21	O71:H11	Not determined	Unknown	ehxCABD	27
GCA_003360155	E	Canada	11	O157:H7	Plasmid	Human	ehxCABD	20
GCA_001281725	E	Netherlands	11	O157:H7	Plasmid	Human	ehxCABD	20
GCA_008757335	B1	USA	17	O103:H2	Not determined	Human	ehxCABD	15
GCA_000619565	B1	USA	16	O111:H8	Not determined	Unknown	ehxCABD	27
GCA_001309735	A	Canada	10	O113:H4	Plasmid	Unknown	ehxCABD	24
GCA_005042365	B1	Canada	679	O163:H19	Plasmid	Unknown	ehxCABD	10
GCA_003361585	E	Canada	11	O157:H7	Plasmid	Cattle	ehxCABD	20
GCA_001617215	B1	China	17	O45:H2	Not determined	Sheep	ehxCABD	15
GCA_002769315	B1	Japan	21	O26:H11	Plasmid	Human	ehxCABD	27
GCA_003787205	E	USA	11	O157:H7	Plasmid	Unknown	ehxCABD	20
GCA_008755655	D	USA	32	?:H28	Plasmid	Human	ehxCABD	8
GCA_001609215	B1	Netherlands	33	O91:H14	Not determined	Human	ehxCABD	12
GCA_003919415	E	USA	11	O157:H7	Plasmid	Unknown	ehxCABD	20
GCA_003113795	B1	Japan	21	O26:H11	Plasmid	Human	ehxCABD	27

GCA_003361555	E	Canada	11	O157:H7	Plasmid	Unknown	ehxCABD	20
GCA_008635645	D	Japan	32	?:H28	Plasmid	Human	ehxCABD	8
GCA_003864835	B1	Japan	655	O121:H19	Plasmid	Human	ehxCABD	18
GCA_004769025	E	USA	11	O157:H7	Plasmid	Pig	ehxCABD	20
GCA_003746545	E	USA	11	?:H7	Plasmid	Unknown	ehxCABD	20
GCA_001309715	E	Canada	11	O157:H7	Plasmid	Unknown	ehxCABD	20
GCA_003787245	E	USA	11	O157:H7	Plasmid	Unknown	ehxCABD	20
GCA_003884155	A	United Kingdom	10	?:H4	Plasmid	Human	ehxCABD	24
GCA_004278555	B1	USA	718	?:H8	Not determined	Unknown	ehxCABD	25
GCA_002923515	B1	Japan	21	O26:H11	Plasmid	Cattle	ehxCABD	27
GCA_003362505	E	Canada	11	O157:H7	Plasmid	Human	ehxCABD	20
GCA_005380705	B1	Japan	655	O121:H19	Plasmid	Human	ehxCABD	18
GCA_005046605	B1	USA	17	O45:H2	Not determined	Human	ehxCABD	15
GCA_005043305	E	Canada	691	?:H20	Plasmid	Unknown	ehxCABD	11
GCA_005046885	D	Switzerland	32	O145:H28	Plasmid	Human	ehxCABD	8
GCA_008634095	D	Japan	32	O145:H28	Plasmid	Human	ehxCABD	8
GCA_004234265	B1	USA	1792	?:H8	Not determined	Unknown	ehxCABD	27
GCA_005044095	B1	Canada	16	O111:H8	Not determined	Human	ehxCABD	27
GCA_005043565	B1	Canada	481	?:H11	Plasmid	Unknown	ehxCABD	27
GCA_002768935	B1	Belgium	21	O26:H11	Plasmid	Human	ehxCABD	27
GCA_001609835	B1	Netherlands	33	O91:H14	Plasmid	Human	ehxCABD	12
GCA_003882975	E	United Kingdom	11	O157:H7	Plasmid	Human	ehxCABD	20
GCA_002764955	B1	Japan	21	O26:H11	Plasmid	Human	ehxCABD	27
GCA_005041705	B1	Canada	2385	?:H19	Plasmid	Unknown	ehxCABD	21
GCA_002769615	B1	Japan	21	O26:H11	Plasmid	Human	ehxCABD	27
GCA_000948825	A	Unknown	342	O177:H25	Plasmid	Unknown	ehxCABD	26
GCA_002924185	B1	Japan	21	O26:H11	Plasmid	Human	ehxCABD	27
GCA_004231865	B1	USA	21	O26:H11	Plasmid	Unknown	ehxCABD	27
GCA_000965655	D	Norway	137	O145:H28	Not determined	Human	ehxCABD	8
GCA_001309775	B1	Canada	16	O111:H8	Not determined	Unknown	ehxCABD	27
GCA_004233765	B1	USA	17	O103:H2	Not determined	Unknown	ehxCABD	15
GCA_003906355	B1	United Kingdom	25	O128:H2	Plasmid	Human	ehxCABD	13
GCA_004234085	B1	USA	655	O121:H19	Plasmid	Unknown	ehxCABD	18
GCA_005381265	B1	Japan	655	O121:H19	Plasmid	Human	ehxCABD	18
GCA_000966935	B1	Norway	655	O121:H19	Plasmid	Human	ehxCABD	18
GCA_000619465	B1	USA	16	O111:H8	Not determined	Unknown	ehxCABD	27
GCA_002768165	B1	Japan	21	O26:H11	Plasmid	Human	ehxCABD	27
GCA_000618925	E	USA	11	O157:H7	Plasmid	Unknown	ehxCABD	20
GCA_000259385	B1	South Korea	4017	O108var1:H2	Plasmid	Human	ehxCABD	25
GCA_002765755	B1	Japan	21	O26:H11	Plasmid	Human	ehxCABD	27
GCA_002195405	E	USA	11	O157:H7	Plasmid	Cattle	ehxCABD	20
GCA_005395845	B1	Japan	101	?:H8	Plasmid	Cattle	ehxCABD	25
GCA_001609815	A	Netherlands	342	O5:NA	Plasmid	Human	ehxCABD	26
GCA_004161835	E	United Kingdom	11	O157:H7	Plasmid	Human	ehxCABD	20
GCA_003908475	B1	United Kingdom	442	O146:H21	Plasmid	Human	ehxCABD	17
GCA_008633685	D	Belgium	32	O145:H28	Plasmid	Unknown	ehxCABD	8
GCA_000948765	E	Unknown	11	O157:H7	Plasmid	Unknown	ehxCABD	20
GCA_004231245	B1	USA	17	?:H2	Not determined	Unknown	ehxCABD	15
GCA_002766655	B1	Japan	21	O26:H11	Plasmid	Human	ehxCABD	27
GCA_005380525	B1	Japan	655	O121:H19	Plasmid	Human	ehxCABD	18
GCA_004256705	E	United Kingdom	628	O157:H7	Plasmid	Human	ehxCABD	20
GCA_002767935	B1	Japan	21	O26:H11	Plasmid	Human	ehxCABD	27
GCA_008753565	E	USA	11	O157:H7	Plasmid	Human	ehxCABD	20
GCA_005041775	B1	Canada	679	O163:H19	Plasmid	Unknown	ehxCABD	10
GCA_005045045	B1	USA	2520	OgN31:H49	Not determined	Unknown	ehxCABD	25
GCA_001191105	B1	USA	17	O103:H2	Plasmid	Human	ehxCABD	15
GCA_003361965	E	Canada	11	O157:H7	Plasmid	Human	ehxCABD	20
GCA_002835085	B1	USA	29	O26:H11	Plasmid	Cattle	ehxCABD	27
GCA_005394825	B1	Japan	297	?:H11	Plasmid	Cattle	ehxCABD	25
GCA_000006665	E	Unknown	-	O157:H7	Plasmid	Unknown	ehxCABD	20
GCA_002469465	E	Mexico	11	O157:H7	Plasmid	Human	ehxCABD	20
GCA_009896505	E	USA	11	O157:H7	Plasmid	Cattle	ehxCABD	20
GCA_005043675	E	Canada	691	Onovel3:H20	Plasmid	Unknown	ehxCABD	11
GCA_002765415	B1	Japan	21	O26:H11	Plasmid	Human	ehxCABD	27
GCA_004253805	E	United Kingdom	11	O157:H7	Plasmid	Human	ehxCABD	20
GCA_004230445	B1	USA	17	O103:H2	Not determined	Unknown	ehxCABD	15
GCA_005040645	B1	Canada	679	O163:H19	Plasmid	Unknown	ehxCABD	10
GCA_002766475	B1	Japan	21	O26:H11	Plasmid	Human	ehxCABD	27
GCA_004264705	E	United Kingdom	11	O157:H7	Plasmid	Human	ehxCABD	20
GCA_001616785	A	China	2	?:H9	Not determined	Sheep	ehxCABD	24
GCA_002531095	B1	Canada	16	O111:H8	Not determined	Unknown	ehxCABD	27
GCA_003884015	E	United Kingdom	11	O157:H7	Plasmid	Human	ehxCABD	20
GCA_003360965	E	Canada	11	O157:H7	Plasmid	Unknown	ehxCABD	20
GCA_001609335	B1	Netherlands	17	O103:H2	Not determined	Human	ehxCABD	15
GCA_005381345	B1	Japan	655	O121:H19	Plasmid	Human	ehxCABD	18
GCA_003915795	E	USA	11	O157:H7	Plasmid	Unknown	ehxCABD	20
GCA_005045365	A	Canada	659	O177:H25	Plasmid	Human	ehxCABD	26
GCA_000618885	E	USA	11	O157:H7	Plasmid	Unknown	ehxCABD	20

GCA_004766685	B1	USA	33	O91:H14	Plasmid	Pig	ehxCABD	12
GCA_001039075	B1	France	21	O26:H11	Plasmid	Human	ehxCABD	27
GCA_001309985	E	Canada	11	O157:H7	Plasmid	Human	ehxCABD	20
GCA_003906255	B1	United Kingdom	33	?:H14	Not determined	Human	ehxCABD	12
GCA_000302715	E	Unknown	11	O157:H7	Plasmid	Unknown	ehxCABD	20
GCA_005045215	B1	USA	58	O116:H21	Plasmid	Unknown	ehxCABD	22
GCA_003113875	B1	Japan	21	O26:H11	Plasmid	Human	ehxCABD	27
GCA_008753515	E	USA	11	O157:H7	Plasmid	Human	ehxCABD	20
GCA_005046765	B1	Canada	223	O113:H21	Plasmid	Cattle	ehxCABD	22
GCA_005040615	B1	Canada	343	O103:H25	Not determined	Unknown	ehxCABD	14
GCA_003903715	B1	United Kingdom	442	O146:H21	Plasmid	Human	ehxCABD	17
GCA_005042555	B1	Canada	16	O111:H8	Not determined	Unknown	ehxCABD	27
GCA_002768555	B1	Japan	21	O26:H11	Plasmid	Human	ehxCABD	27
GCA_003916535	B1	USA	16	?:H8	Not determined	Unknown	ehxCABD	27
GCA_005043265	B1	Canada	480	?:H8	Not determined	Unknown	ehxCABD	27
GCA_001440725	A	Belgium	342	O5:NA	Plasmid	Human	ehxCABD	26
GCA_003787645	E	USA	11	O157:H7	Plasmid	Unknown	ehxCABD	20
GCA_002769295	B1	Japan	21	O26:H11	Plasmid	Human	ehxCABD	27
GCA_002379315	B1	USA	223	O113:H21	Plasmid	Cattle	ehxCABD	22
GCA_002810755	B1	USA	29	O26:H11	Plasmid	Cattle	ehxCABD	27
GCA_002765315	B1	Japan	21	O26:H11	Plasmid	Human	ehxCABD	27
GCA_003766025	B1	USA	17	O103:H2	Not determined	Unknown	ehxCABD	15
GCA_004231305	B1	USA	21	O26:H11	Plasmid	Unknown	ehxCABD	27
GCA_005045445	B1	Canada	21	O26:H11	Plasmid	Human	ehxCABD	27
GCA_001282025	E	Netherlands	11	O157:H7	Not determined	Human	ehxCABD	20
GCA_003755005	E	USA	11	O157:H7	Plasmid	Unknown	ehxCABD	20
GCA_003340675	B1	USA	29	O26:H11	Plasmid	Cattle	ehxCABD	27
GCA_003764385	E	USA	11	?:H7	Plasmid	Unknown	ehxCABD	20
GCA_003027175	E	Austria	11	O157:H7	Plasmid	Human	ehxCABD	20
GCA_003914115	B1	USA	21	O26:H11	Plasmid	Unknown	ehxCABD	27
GCA_004231045	E	USA	11	O157:H7	Plasmid	Unknown	ehxCABD	20
GCA_002530885	B1	Canada	16	O111:NA	Not determined	Unknown	ehxCABD	27
GCA_003760145	B1	USA	357	?:H7	Not determined	Unknown	ehxCABD	25
GCA_000616465	E	USA	11	O157:H7	Plasmid	Unknown	ehxCABD	20
GCA_004164235	D	United Kingdom	32	?:H28	Plasmid	Human	ehxCABD	8
GCA_000619745	B1	USA	655	O121:H19	Plasmid	Unknown	ehxCABD	18
GCA_004233365	B1	USA	16	?:H8	Not determined	Unknown	ehxCABD	27
GCA_003752795	B1	USA	17	O103:H2	Not determined	Unknown	ehxCABD	15
GCA_000618985	E	USA	11	O157:H7	Plasmid	Unknown	ehxCABD	20
GCA_004254895	B1	United Kingdom	21	O26:H11	Plasmid	Human	ehxCABD	27
GCA_004257845	E	United Kingdom	11	O157:H7	Plasmid	Human	ehxCABD	20
GCA_005045035	B1	Denmark	350	O91:H21	Plasmid	Unknown	ehxCABD	17
GCA_004230765	B1	USA	-	O103:H11	Not determined	Unknown	ehxCABD	27
GCA_001609315	B1	Netherlands	33	O91:H14	Plasmid	Human	ehxCABD	12
GCA_004264685	B1	United Kingdom	5822	Onovel27:H7	Plasmid	Human	ehxCABD	25
GCA_000619765	B1	USA	17	O103:H2	Not determined	Unknown	ehxCABD	15
GCA_002765455	B1	Japan	21	O26:H11	Plasmid	Human	ehxCABD	27
GCA_002467465	B1	Canada	17	O103:H2	Not determined	Human	ehxCABD	15
GCA_004161955	E	United Kingdom	628	O157:H7	Plasmid	Human	ehxCABD	20
GCA_002764855	B1	Japan	21	O26:H11	Plasmid	Human	ehxCABD	27
GCA_004165255	B1	United Kingdom	675	O76:H19	Not determined	Human	ehxCABD	25
GCA_004182495	B1	United Kingdom	388	?:H2	Plasmid	Human	ehxCABD	25
GCA_002923815	B1	Japan	21	O26:H11	Plasmid	Human	ehxCABD	27
GCA_000617725	E	USA	11	O157:H7	Plasmid	Unknown	ehxCABD	20
GCA_001607955	B1	Netherlands	25	O128:H2	Plasmid	Human	ehxCABD	13
GCA_000473725	D	Unknown	32	?:H28	Plasmid	Unknown	ehxCABD	8
GCA_004270345	B1	United Kingdom	21	O26:H11	Plasmid	Human	ehxCABD	27
GCA_004264605	B1	United Kingdom	8537	O26:H11	Plasmid	Human	ehxCABD	27
GCA_001191125	D	USA	32	O145:H28	Plasmid	Human	ehxCABD	8
GCA_001012265	B1	USA	16	O111:H8	Not determined	Wild animals	ehxCABD	27
GCA_001660305	E	Japan	11	O157:H7	Plasmid	Cattle	ehxCABD	20
GCA_003878895	E	United Kingdom	11	O157:H7	Plasmid	Human	ehxCABD	20
GCA_002764455	B1	Japan	21	O26:H11	Plasmid	Cattle	ehxCABD	27
GCA_003122855	A	France	301	O80:H2	Not determined	Human	ehxCABD	19
GCA_002766335	B1	Japan	21	O26:H11	Plasmid	Human	ehxCABD	27
GCA_002837305	B1	USA	29	O26:H11	Plasmid	Cattle	ehxCABD	27
GCA_000303955	E	Unknown	11	O157:H7	Plasmid	Unknown	ehxCABD	20
GCA_005042175	B1	Canada	718	?:H8	Not determined	Unknown	ehxCABD	25
GCA_003361815	E	Canada	11	O157:H7	Plasmid	Cattle	ehxCABD	20
GCA_008634505	D	Japan	32	O145:H28	Plasmid	Human	ehxCABD	8
GCA_002769775	B1	Japan	21	O26:H11	Plasmid	Human	ehxCABD	27
GCA_008634165	D	Japan	32	O145:H28	Plasmid	Human	ehxCABD	8
GCA_002462035	A	USA	342	O5:NA	Plasmid	Human	ehxCABD	26
GCA_001191455	B1	USA	1817	O104:H7	Plasmid	Unknown	ehxCABD	25
GCA_002769635	B1	Japan	21	O26:H11	Plasmid	Human	ehxCABD	27
GCA_002766115	B1	Japan	21	O26:H11	Plasmid	Human	ehxCABD	27
GCA_000462845	E	Unknown	11	O157:H7	Plasmid	Unknown	ehxCABD	20
GCA_005044435	B1	Canada	655	O121:H19	Plasmid	Human	ehxCABD	18

GCA_002767435	B1	Japan	21	O26:H11	Plasmid	Human	ehxCABD	27
GCA_005045685	B1	Denmark	33	O91:H14	Not determined	Unknown	ehxCABD	12
GCA_000965625	B1	Norway	655	O121:H19	Plasmid	Human	ehxCABD	18
GCA_000462525	E	Unknown	11	O157:H7	Plasmid	Unknown	ehxCABD	20
GCA_005038135	B1	Canada	302	O139:H19	Plasmid	Cattle	ehxCABD	25
GCA_002463845	B1	USA	297	O130:H11	Plasmid	Pig	ehxCABD	25
GCA_004269745	E	United Kingdom	11	O157:H7	Plasmid	Human	ehxCABD	20
GCA_001607025	B1	Netherlands	21	O26:H11	Plasmid	Human	ehxCABD	27
GCA_003881995	E	United Kingdom	11	O157:H7	Plasmid	Human	ehxCABD	20
GCA_000304835	E	Unknown	11	O157:H7	Plasmid	Unknown	ehxCABD	20
GCA_001012345	B1	USA	679	O163:H19	Plasmid	Unknown	ehxCABD	10
GCA_004267805	A	United Kingdom	301	O80:H2	Not determined	Human	ehxCABD	19
GCA_005045625	B1	Canada	21	O26:H11	Plasmid	Human	ehxCABD	27
GCA_002518445	B1	USA	442	?:H21	Not determined	Pig	ehxCABD	17
GCA_000614565	B1	USA	17	O153var1:H2	Plasmid	Unknown	ehxCABD	15
GCA_002176275	E	USA	11	O157:H7	Not determined	Cattle	ehxCABD	20
GCA_003741425	B1	USA	679	?:H19	Plasmid	Unknown	ehxCABD	10
GCA_003753985	B1	USA	17	?:H2	Plasmid	Unknown	ehxCABD	15
GCA_004266535	E	United Kingdom	11	O157:H7	Plasmid	Human	ehxCABD	20
GCA_005042965	B1	Canada	723	O103:H11	Plasmid	Unknown	ehxCABD	27
GCA_000616485	E	USA	11	O157:H7	Plasmid	Unknown	ehxCABD	20
GCA_003360235	E	Canada	11	O157:H7	Plasmid	Human	ehxCABD	20
GCA_001616585	B1	China	13	O174:H8	Plasmid	Sheep	ehxCABD	25
GCA_001660275	B1	Japan	223	O113:H21	Plasmid	Cattle	ehxCABD	22
GCA_005041865	B1	Canada	679	O163:H19	Plasmid	Unknown	ehxCABD	10
GCA_005042135	A	Canada	342	O5:NA	Plasmid	Unknown	ehxCABD	26
GCA_000695175	D	USA	32	O145:H28	Plasmid	Unknown	ehxCABD	8
GCA_005041455	E	Canada	11	O157:H7	Plasmid	Unknown	ehxCABD	20
GCA_002531025	B1	Canada	21	O111:H11	Plasmid	Unknown	ehxCABD	27
GCA_004157305	E	United Kingdom	11	O157:H7	Plasmid	Human	ehxCABD	20
GCA_003905255	E	USA	11	O157:H7	Plasmid	Unknown	ehxCABD	20
GCA_000616525	E	USA	11	O157:H7	Plasmid	Unknown	ehxCABD	20
GCA_005042895	B1	Canada	21	?:H11	Plasmid	Unknown	ehxCABD	27
GCA_001263035	B1	USA	21	O151.Gp3/O118.Gp3:H16	Plasmid	Human	ehxCABD	27
GCA_003755245	E	USA	11	O157:H7	Plasmid	Unknown	ehxCABD	20
GCA_003293905	B1	USA	16	O111:H8	Not determined	Cattle	ehxCABD	27
GCA_008635525	D	Japan	32	?:H28	Plasmid	Human	ehxCABD	8
GCA_001262905	G	USA	385	O183.Gp16:H18	Plasmid	Unknown	ehxCABD	29
GCA_003895785	B1	USA	17	?:H2	Not determined	Unknown	ehxCABD	15
GCA_004231025	B1	USA	17	O103:H2	Not determined	Unknown	ehxCABD	15
GCA_003896915	B1	USA	21	O26:H11	Plasmid	Unknown	ehxCABD	27
GCA_003738485	E	USA	11	?:H7	Plasmid	Unknown	ehxCABD	20
GCA_005381165	B1	Japan	655	O121:H19	Plasmid	Human	ehxCABD	18
GCA_004275585	E	United Kingdom	11	O157:H7	Plasmid	Human	ehxCABD	20
GCA_003360675	E	Canada	11	O157:H7	Plasmid	Human	ehxCABD	20
GCA_005039795	E	Canada	724	Onovel3:H20	Plasmid	Cattle	ehxCABD	11
GCA_002769915	B1	Japan	21	O26:H11	Plasmid	Human	ehxCABD	27
GCA_000614345	E	USA	11	O157:H7	Plasmid	Unknown	ehxCABD	20
GCA_002764415	B1	Japan	21	O26:H11	Plasmid	Cattle	ehxCABD	27
GCA_000335235	E	Unknown	11	?:H7	Plasmid	Unknown	ehxCABD	20
GCA_003908195	B1	United Kingdom	442	O146:H21	Plasmid	Human	ehxCABD	17
GCA_001607895	A	Netherlands	10	O113:H4	Plasmid	Human	ehxCABD	24
GCA_003755705	B1	USA	17	?:H2	Plasmid	Unknown	ehxCABD	15
GCA_002766455	B1	Japan	21	O26:H11	Plasmid	Human	ehxCABD	27
GCA_007648965	B1	Brazil	21	O26:H11	Plasmid	Human	ehxCABD	27
GCA_002764995	B1	Japan	21	O26:H11	Plasmid	Human	ehxCABD	27
GCA_001262785	B1	USA	397	O22:H8	Plasmid	Unknown	ehxCABD	25
GCA_002769475	B1	Japan	21	O26:H11	Plasmid	Human	ehxCABD	27
GCA_004173965	B1	United Kingdom	442	O146:H21	Plasmid	Human	ehxCABD	17
GCA_002769035	B1	Belgium	21	O26:H11	Plasmid	Human	ehxCABD	27
GCA_002176415	E	USA	11	O157:H7	Not determined	Cattle	ehxCABD	20
GCA_005394585	A	Japan	6126	?:H25	Not determined	Cattle	ehxCABD	26
GCA_003864855	B1	Japan	655	O121:H19	Plasmid	Human	ehxCABD	18
GCA_004276335	B1	United Kingdom	21	O26:H11	Plasmid	Human	ehxCABD	27
GCA_003360495	E	Canada	11	O157:H7	Plasmid	Human	ehxCABD	20
GCA_000619125	B1	USA	655	O121:H19	Plasmid	Unknown	ehxCABD	18
GCA_000614885	E	USA	11	O157:H7	Plasmid	Unknown	ehxCABD	20
GCA_003028075	A	Switzerland	301	O80:H2	Not determined	Unknown	ehxCABD	19
GCA_002475865	E	USA	11	O157:H7	Plasmid	Unknown	ehxCABD	20
GCA_005043185	E	Canada	724	Onovel3:H20	Plasmid	Cattle	ehxCABD	11
GCA_000622635	B1	USA	21	O26:H11	Plasmid	Unknown	ehxCABD	27
GCA_000335415	E	Unknown	11	O157:H7	Plasmid	Unknown	ehxCABD	20
GCA_001677625	E	Argentina	11	O157:H7	Plasmid	Human	ehxCABD	20
GCA_003360515	E	Canada	11	O157:H7	Plasmid	Human	ehxCABD	20
GCA_004270715	E	United Kingdom	628	O157:H7	Plasmid	Human	ehxCABD	20
GCA_005042255	B1	Canada	679	O163:H19	Plasmid	Unknown	ehxCABD	10
GCA_004256265	E	United Kingdom	11	O157:H7	Plasmid	Human	ehxCABD	20
GCA_002768415	B1	Japan	21	O26:H11	Plasmid	Human	ehxCABD	27

GCA_001607835	A	Netherlands	659	O177:H25	Plasmid	Human	ehxCABD	26
GCA_002766915	B1	Japan	21	O26:H11	Plasmid	Human	ehxCABD	27
GCA_008753725	E	USA	11	O157:H7	Plasmid	Human	ehxCABD	20
GCA_004181485	E	United Kingdom	11	O157:H7	Plasmid	Human	ehxCABD	20
GCA_005044375	B1	Canada	21	O69:H11	Plasmid	Human	ehxCABD	27
GCA_009495455	E	France	11	O157:H7	Plasmid	Unknown	ehxCABD	20
GCA_002164415	E	USA	5560	O157:H7	Plasmid	Cattle	ehxCABD	20
GCA_001012175	B1	USA	1611	O96:H19	Plasmid	Unknown	ehxCABD	25
GCA_004274975	B1	United Kingdom	21	O26:H11	Plasmid	Human	ehxCABD	27
GCA_000194395	B1	Unknown	655	O121:H19	Plasmid	Unknown	ehxCABD	18
GCA_002475935	E	USA	11	O157:H7	Plasmid	Unknown	ehxCABD	20
GCA_001609735	B1	Netherlands	300	O182:H25	Not determined	Human	ehxCABD	14
GCA_003920235	B1	USA	17	O103:H2	Not determined	Unknown	ehxCABD	15
GCA_005392265	A	Japan	329	O136:H16	Plasmid	Cattle	ehxCABD	23
GCA_000617245	B1	USA	16	O111:H8	Not determined	Unknown	ehxCABD	27
GCA_004231465	A	USA	119	?:H25	Plasmid	Unknown	ehxCABD	26
GCA_008633845	D	Japan	32	O145:H28	Plasmid	Human	ehxCABD	8
GCA_003362335	E	Canada	11	O157:H7	Plasmid	Human	ehxCABD	20
GCA_002770175	B1	Japan	21	O26:H11	Plasmid	Human	ehxCABD	27
GCA_003924775	E	United Kingdom	11	O157:H7	Plasmid	Human	ehxCABD	20
GCA_003910915	B1	USA	21	?:H11	Plasmid	Unknown	ehxCABD	27
GCA_001606625	B1	Netherlands	447	O5:H19	Not determined	Human	ehxCABD	25
GCA_002379235	B1	USA	5975	O113:H21	Plasmid	Pig	ehxCABD	22
GCA_002767095	B1	Japan	21	O26:H11	Plasmid	Human	ehxCABD	27
GCA_005046485	B1	Canada	655	O121:H19	Plasmid	Cattle	ehxCABD	18
GCA_000616195	B1	USA	16	O111:H8	Not determined	Unknown	ehxCABD	27
GCA_000513035	E	USA	11	O157:H7	Not determined	Human	ehxCABD	20
GCA_002837335	B1	USA	29	O26:H11	Plasmid	Cattle	ehxCABD	27
GCA_002460705	D	USA	32	?:H28	Plasmid	Human	ehxCABD	8
GCA_008635935	D	Japan	32	?:H28	Plasmid	Human	ehxCABD	8
GCA_005041835	B1	Canada	679	O163:H19	Plasmid	Unknown	ehxCABD	10
GCA_004253505	B1	United Kingdom	8637	O21:H2	Plasmid	Human	ehxCABD	13
GCA_003360855	E	Canada	11	O157:H7	Plasmid	Sheep	ehxCABD	20
GCA_002795085	E	USA	11	?:H7	Plasmid	Human	ehxCABD	20
GCA_005043235	B1	Canada	17	O103:H2	Not determined	Unknown	ehxCABD	15
GCA_003905275	B1	United Kingdom	33	O91:H14	Not determined	Human	ehxCABD	12
GCA_004797875	D	USA	32	?:H28	Plasmid	Cattle	ehxCABD	8
GCA_003905005	B1	United Kingdom	21	O26:H11	Plasmid	Human	ehxCABD	27
GCA_005400765	A	France	342	O177:H25	Plasmid	Cattle	ehxCABD	26
GCA_005040555	B1	Canada	17	O103:H2	Plasmid	Unknown	ehxCABD	15
GCA_003770445	B1	USA	655	O121:H19	Plasmid	Unknown	ehxCABD	18
GCA_004797565	D	USA	32	O145:H28	Plasmid	Cattle	ehxCABD	8
GCA_000616665	D	USA	32	O145:H28	Plasmid	Unknown	ehxCABD	8
GCA_005045595	A	Canada	5486	O165:H25	Plasmid	Human	ehxCABD	26
GCA_001572455	B1	USA	29	O26:H11	Plasmid	Cattle	ehxCABD	27
GCA_008634125	D	Japan	32	O145:H28	Plasmid	Human	ehxCABD	8
GCA_003361395	E	Canada	11	O157:H7	Plasmid	Unknown	ehxCABD	20
GCA_004230705	B1	USA	21	O26:H11	Plasmid	Unknown	ehxCABD	27
GCA_002770295	B1	Japan	21	O26:H11	Plasmid	Human	ehxCABD	27
GCA_001677695	E	Argentina	11	O157:H7	Plasmid	Human	ehxCABD	20
GCA_002766895	B1	Japan	21	O26:H11	Plasmid	Human	ehxCABD	27
GCA_002475795	E	USA	11	O157:H7	Plasmid	Unknown	ehxCABD	20
GCA_002134285	E	Canada	11	O157:H7	Plasmid	Unknown	ehxCABD	20
GCA_005392105	A	Japan	6126	O10:H25	Not determined	Cattle	ehxCABD	26
GCA_005380765	B1	Japan	655	O121:H19	Plasmid	Human	ehxCABD	18
GCA_004215785	Unknown	United Kingdom	1819	?:H28	Plasmid	Human	ehxCABD	23
GCA_001266015	D	Gambia	137	O145:H28	Plasmid	Human	ehxCABD	8
GCA_003361775	E	Canada	11	O157:H7	Plasmid	Unknown	ehxCABD	20
GCA_000506845	B1	Unknown	17	O45:H2	Not determined	Unknown	ehxCABD	15
GCA_005042715	B1	Canada	679	O163:H19	Plasmid	Unknown	ehxCABD	10
GCA_002475975	E	USA	11	O157:H7	Plasmid	Unknown	ehxCABD	20
GCA_003770105	E	USA	11	O157:H7	Plasmid	Unknown	ehxCABD	20
GCA_005042875	B1	Canada	58	O153var1:H25	Plasmid	Cattle	ehxCABD	22
GCA_000614805	E	USA	11	O157:H7	Plasmid	Unknown	ehxCABD	20
GCA_004265985	E	United Kingdom	11	O157:H7	Plasmid	Human	ehxCABD	20
GCA_005395045	B1	Japan	679	?:H19	Plasmid	Cattle	ehxCABD	10
GCA_002770435	B1	Japan	21	O26:H11	Not determined	Human	ehxCABD	27
GCA_003362595	E	Canada	11	O157:H7	Plasmid	Human	ehxCABD	20
GCA_004232825	A	USA	-	?:H25	Plasmid	Unknown	ehxCABD	26
GCA_000334995	E	Unknown	11	?:H7	Plasmid	Unknown	ehxCABD	20
GCA_004156855	B1	United Kingdom	21	O26:H11	Plasmid	Human	ehxCABD	27
GCA_005380645	B1	Japan	5536	O121:H19	Plasmid	Human	ehxCABD	18
GCA_005045975	D	Switzerland	32	O145:H28	Plasmid	Human	ehxCABD	8
GCA_000617605	B1	USA	655	O121:H19	Plasmid	Unknown	ehxCABD	18
GCA_003362695	E	Canada	11	O157:H7	Plasmid	Human	ehxCABD	20
GCA_004268445	E	United Kingdom	11	O157:H7	Plasmid	Human	ehxCABD	20
GCA_002175805	E	USA	11	O157:H7	Plasmid	Cattle	ehxCABD	20
GCA_005038625	B1	Canada	302	O139:H19	Plasmid	Cattle	ehxCABD	25

GCA_005042275	B1	Canada	679	O163:H19	Plasmid	Unknown	ehxCABD	10
GCA_005397085	B1	Japan	481	?:H11	Plasmid	Cattle	ehxCABD	27
GCA_003752825	E	USA	11	?:H7	Plasmid	Unknown	ehxCABD	20
GCA_003307155	E	Japan	11	O157:H7	Plasmid	Human	ehxCABD	20
GCA_002474125	E	USA	11	O157:H7	Plasmid	Unknown	ehxCABD	20
GCA_008756245	B1	USA	16	O111:H8	Not determined	Human	ehxCABD	27
GCA_002457965	B1	United Kingdom	16	O111:H8	Not determined	Cattle	ehxCABD	27
GCA_004163655	B1	United Kingdom	21	O26:H11	Plasmid	Human	ehxCABD	27
GCA_000462425	E	Unknown	11	O157:H7	Plasmid	Unknown	ehxCABD	20
GCA_002764575	B1	Japan	21	O26:H11	Plasmid	Human	ehxCABD	27
GCA_003777565	E	USA	11	O157:H7	Plasmid	Unknown	ehxCABD	20
GCA_005380885	B1	Japan	655	O121:H19	Plasmid	Human	ehxCABD	18
GCA_002924245	B1	Japan	21	O26:H11	Plasmid	Cattle	ehxCABD	27
GCA_002766675	B1	Japan	21	O26:H11	Plasmid	Human	ehxCABD	27
GCA_003361825	E	Canada	628	O157:H7	Plasmid	Unknown	ehxCABD	20
GCA_004796915	D	USA	32	O145:H28	Plasmid	Cattle	ehxCABD	8
GCA_005380905	B1	Japan	655	O121:H19	Plasmid	Human	ehxCABD	18
GCA_002164455	E	USA	11	O157:H7	Plasmid	Cattle	ehxCABD	20
GCA_005394145	B1	Japan	-	O6:H34	Plasmid	Cattle	ehxCABD	25
GCA_001281855	E	Netherlands	11	O157:H7	Plasmid	Human	ehxCABD	20
GCA_003902165	E	United Kingdom	11	O157:H7	Plasmid	Human	ehxCABD	20
GCA_002766515	B1	Japan	21	O26:H11	Plasmid	Human	ehxCABD	27
GCA_004255345	B1	United Kingdom	300	O182:H25	Not determined	Human	ehxCABD	14
GCA_004176215	B1	United Kingdom	442	?:H21	Plasmid	Human	ehxCABD	17
GCA_003754205	E	USA	11	?:H7	Plasmid	Unknown	ehxCABD	20
GCA_002474205	E	USA	11	O157:H7	Plasmid	Unknown	ehxCABD	20
GCA_008755455	B1	USA	655	O121:H19	Plasmid	Human	ehxCABD	18
GCA_004232585	B1	USA	21	?:H16	Not determined	Unknown	ehxCABD	27
GCA_002530855	B1	Canada	16	O111:H8	Not determined	Unknown	ehxCABD	27
GCA_002837475	B1	USA	29	O26:H11	Plasmid	Cattle	ehxCABD	27
GCA_003884135	B1	United Kingdom	25	O128:H2	Plasmid	Human	ehxCABD	13
GCA_004230905	E	USA	11	O157:H7	Plasmid	Unknown	ehxCABD	20
GCA_000616325	B1	USA	480	O111:H8	Not determined	Unknown	ehxCABD	27
GCA_003362665	E	Canada	628	O157:H7	Plasmid	Human	ehxCABD	20
GCA_005045235	B1	USA	297	O130:H11	Plasmid	Unknown	ehxCABD	25
GCA_000618285	E	USA	11	O157:H7	Plasmid	Unknown	ehxCABD	20
GCA_002460035	B1	USA	343	O103:H25	Not determined	Human	ehxCABD	14
GCA_003361405	E	Canada	11	O157:H7	Plasmid	Unknown	ehxCABD	20
GCA_002531395	B1	Canada	17	O103:H2	Not determined	Unknown	ehxCABD	15
GCA_005038275	B1	Canada	306	O84:H2	Not determined	Unknown	ehxCABD	14
GCA_003359855	E	Canada	11	?:H7	Plasmid	Human	ehxCABD	20
GCA_005394425	B1	Japan	679	?:H19	Plasmid	Cattle	ehxCABD	10
GCA_005390485	B1	Japan	327	O177:H11	Plasmid	Cattle	ehxCABD	27
GCA_002924165	B1	Japan	21	O26:H11	Plasmid	Human	ehxCABD	27
GCA_004230665	B1	USA	655	O121:H19	Plasmid	Unknown	ehxCABD	18
GCA_005381005	B1	Japan	655	O121:H19	Plasmid	Human	ehxCABD	18
GCA_003767145	A	USA	5309	O145:H25	Not determined	Unknown	ehxCABD	26
GCA_004181505	E	United Kingdom	11	O157:H7	Plasmid	Human	ehxCABD	20
GCA_008755735	B1	USA	17	O103:H2	Not determined	Human	ehxCABD	15
GCA_000615605	B1	USA	16	O111:H8	Not determined	Unknown	ehxCABD	27
GCA_003764205	B1	USA	16	?:H8	Not determined	Unknown	ehxCABD	27
GCA_008755545	B1	USA	16	?:H8	Not determined	Human	ehxCABD	27
GCA_005393035	A	Japan	342	O5:NA	Plasmid	Cattle	ehxCABD	26
GCA_005045955	B1	Canada	16	O111:H8	Not determined	Cattle	ehxCABD	27
GCA_003757565	B1	USA	21	O26:H11	Plasmid	Unknown	ehxCABD	27
GCA_002144165	B1	Canada	21	O26:H11	Plasmid	Human	ehxCABD	27
GCA_002475705	E	USA	11	O157:H7	Plasmid	Unknown	ehxCABD	20
GCA_005040135	E	Canada	724	?:H20	Plasmid	Unknown	ehxCABD	11
GCA_003756505	E	USA	11	O157:H7	Plasmid	Unknown	ehxCABD	20
GCA_004256225	A	United Kingdom	10	?:H26	Plasmid	Human	ehxCABD	24
GCA_003755365	B1	USA	21	?:H11	Plasmid	Unknown	ehxCABD	27
GCA_004215775	A	United Kingdom	10	O38:H26	Plasmid	Human	ehxCABD	24
GCA_001607575	B1	Netherlands	661	O174:H2	Plasmid	Human	ehxCABD	25
GCA_005381145	B1	Japan	655	O121:H19	Plasmid	Human	ehxCABD	18
GCA_000616425	E	USA	11	O157:H7	Plasmid	Unknown	ehxCABD	20
GCA_002462695	B1	USA	442	O91:H21	Plasmid	Unknown	ehxCABD	17
GCA_003361855	E	Canada	11	O157:H7	Plasmid	Unknown	ehxCABD	20
GCA_009821015	B1	Bangladesh	223	O113:H21	Plasmid	Cattle	ehxCABD	22
GCA_003113635	B1	Japan	21	O26:H11	Plasmid	Human	ehxCABD	27
GCA_004276295	E	United Kingdom	11	O157:H7	Plasmid	Human	ehxCABD	20
GCA_000617185	B1	USA	655	O121:H19	Plasmid	Unknown	ehxCABD	18
GCA_003895475	B1	USA	17	O45:H2	Not determined	Unknown	ehxCABD	15
GCA_004255145	E	United Kingdom	11	O157:NA	Plasmid	Human	ehxCABD	20
GCA_000965545	B1	Norway	17	O123.O186.Gp5/O123.Gp5:H2	Not determined	Human	ehxCABD	15
GCA_005393965	D	Japan	32	?:H28	Plasmid	Cattle	ehxCABD	8
GCA_005393945	B1	Japan	17	O103:H2	Plasmid	Cattle	ehxCABD	15
GCA_003878795	E	United Kingdom	11	O157:H7	Plasmid	Human	ehxCABD	20
GCA_002486695	E	USA	11	O157:H7	Plasmid	Unknown	ehxCABD	20

GCA_005394305	B1	Japan	58	?:H25	Plasmid	Cattle	ehxCABD	22
GCA_002770275	B1	Japan	21	O26:H11	Plasmid	Human	ehxCABD	27
GCA_001012255	B1	USA	16	O111:H8	Not determined	Unknown	ehxCABD	27
GCA_002768975	B1	Belgium	21	O26:H11	Plasmid	Human	ehxCABD	27
GCA_002175715	E	USA	11	O157:H7	Plasmid	Cattle	ehxCABD	20
GCA_003359595	E	Canada	11	O157:H7	Plasmid	Human	ehxCABD	20
GCA_003765945	E	USA	11	?:H7	Plasmid	Unknown	ehxCABD	20
GCA_004233205	E	USA	11	?:H7	Plasmid	Unknown	ehxCABD	20
GCA_003736145	E	USA	11	O157:H7	Plasmid	Unknown	ehxCABD	20
GCA_001677775	E	Argentina	11	O157:H7	Plasmid	Human	ehxCABD	20
GCA_005045855	B1	Canada	306	O84:H2	Plasmid	Unknown	ehxCABD	14
GCA_004270545	E	United Kingdom	11	O157:H7	Plasmid	Human	ehxCABD	20
GCA_005041275	B1	Canada	17	O103:H2	Not determined	Unknown	ehxCABD	15
GCA_002810695	cladeI	USA	3692	O2.Gp7/O2.O50.Gp7:H45	Plasmid	Cattle	ehxCABD	29
GCA_000414155	B1	Germany	794	O76:H7	Not determined	Pig	ehxCABD	25
GCA_008635895	D	Japan	32	?:H28	Plasmid	Human	ehxCABD	8
GCA_001191195	D	USA	32	O145:H28	Plasmid	Unknown	ehxCABD	8
GCA_002164205	E	USA	11	O157:H7	Plasmid	Cattle	ehxCABD	20
GCA_000215245	B1	Unknown	223	O113:H21	Plasmid	Human	ehxCABD	22
GCA_004232065	B1	USA	17	?:H2	Plasmid	Unknown	ehxCABD	15
GCA_002133915	B1	Canada	2103	O48:H21	Plasmid	Unknown	ehxCABD	25
GCA_005390505	B1	Japan	223	O113:H21	Plasmid	Cattle	ehxCABD	22
GCA_004180795	B1	United Kingdom	17	?:H2	Not determined	Human	ehxCABD	15
GCA_000622465	B1	USA	21	O26:H11	Plasmid	Unknown	ehxCABD	27
GCA_003736365	B1	USA	17	O103:H2	Plasmid	Unknown	ehxCABD	15
GCA_000619585	B1	USA	16	O111:H8	Not determined	Unknown	ehxCABD	27
GCA_005381125	B1	Japan	655	O121:H19	Plasmid	Human	ehxCABD	18
GCA_005391125	B1	Japan	154	O88:H25	Plasmid	Cattle	ehxCABD	25
GCA_004232565	B1	USA	21	?:H11	Plasmid	Unknown	ehxCABD	27
GCA_001677545	E	Argentina	11	O157:H7	Plasmid	Human	ehxCABD	20
GCA_000618705	B1	USA	655	O121:H19	Plasmid	Unknown	ehxCABD	18
GCA_002520335	B1	USA	679	O163:H19	Not determined	Cattle	ehxCABD	10
GCA_001607445	B1	Netherlands	17	O103:H2	Not determined	Human	ehxCABD	15
GCA_002769935	B1	Japan	21	O26:H11	Plasmid	Human	ehxCABD	27
GCA_002379265	B1	USA	5975	O113:H21	Plasmid	Pig	ehxCABD	22
GCA_004280875	E	United Kingdom	11	O157:H7	Plasmid	Human	ehxCABD	20
GCA_003901155	E	United Kingdom	11	O157:H7	Plasmid	Human	ehxCABD	20
GCA_002319335	B1	Poland	21	O26:H11	Plasmid	Pig	ehxCABD	27
GCA_002764255	B1	Belgium	21	O26:H11	Plasmid	Cattle	ehxCABD	27
GCA_000618725	B1	USA	655	O121:H19	Plasmid	Unknown	ehxCABD	18
GCA_004181705	E	United Kingdom	11	O157:H7	Plasmid	Human	ehxCABD	20
GCA_005394205	B1	Japan	87	O28ac.O42.Gp2:H25	Plasmid	Cattle	ehxCABD	22
GCA_002768695	B1	Japan	21	O26:H11	Plasmid	Human	ehxCABD	27
GCA_003294005	B1	Canada	16	O111:H8	Not determined	Human	ehxCABD	27
GCA_004269705	E	United Kingdom	11	O157:H7	Plasmid	Human	ehxCABD	20
GCA_000632595	B1	USA	16	O111:H8	Not determined	Unknown	ehxCABD	27
GCA_000619005	B1	USA	16	O111:H8	Not determined	Unknown	ehxCABD	27
GCA_003360775	E	Canada	11	O157:H7	Plasmid	Human	ehxCABD	20
GCA_008633565	D	Japan	32	O145:H28	Plasmid	Human	ehxCABD	8
GCA_004164575	E	United Kingdom	11	O157:H7	Plasmid	Human	ehxCABD	20
GCA_008756635	B1	USA	16	O111:H8	Not determined	Human	ehxCABD	27
GCA_000615865	E	USA	11	O157:H7	Plasmid	Unknown	ehxCABD	20
GCA_003916635	E	USA	11	O157:H7	Plasmid	Unknown	ehxCABD	20
GCA_005044025	B1	Canada	655	O121:H19	Plasmid	Human	ehxCABD	18
GCA_003770505	B1	USA	655	O121:H19	Plasmid	Unknown	ehxCABD	18
GCA_004230925	E	USA	11	O157:H7	Plasmid	Unknown	ehxCABD	20
GCA_005040255	B1	Canada	21	?:H11	Not determined	Unknown	ehxCABD	27
GCA_003907435	B1	United Kingdom	300	O156:H25	Not determined	Human	ehxCABD	14
GCA_005044325	B1	Canada	21	O26:H11	Plasmid	Human	ehxCABD	27
GCA_003881875	A	United Kingdom	10	?:H4	Plasmid	Human	ehxCABD	24
GCA_002515905	B1	USA	649	?:H19	Not determined	Unknown	ehxCABD	21
GCA_000234215	E	Germany	11	O157:H7	Not determined	Human	ehxCABD	20
GCA_004174735	B1	United Kingdom	675	O76:H19	Not determined	Human	ehxCABD	25
GCA_004165375	E	United Kingdom	2966	O157:H7	Plasmid	Human	ehxCABD	20
GCA_003906795	B1	United Kingdom	442	O146:H21	Plasmid	Human	ehxCABD	17
GCA_004264305	E	United Kingdom	11	O157:H7	Plasmid	Human	ehxCABD	20
GCA_008755975	B1	USA	16	O111:H8	Not determined	Human	ehxCABD	27
GCA_002769455	B1	Japan	21	O26:H11	Plasmid	Human	ehxCABD	27
GCA_000616605	E	USA	11	O157:H7	Plasmid	Unknown	ehxCABD	20
GCA_002735025	B1	USA	29	O26:H11	Plasmid	Cattle	ehxCABD	27
GCA_002764435	B1	Japan	21	O26:H11	Plasmid	Cattle	ehxCABD	27
GCA_002515145	B1	USA	17	O103:H2	Not determined	Human	ehxCABD	15
GCA_001297985	E	Netherlands	11	O157:H7	Plasmid	Human	ehxCABD	20
GCA_002518395	B1	USA	135	O103:H2	Not determined	Wild animals	ehxCABD	15
GCA_005042105	B1	Canada	21	?:H11	Plasmid	Unknown	ehxCABD	27
GCA_002173125	B1	USA	1817	O104:H7	Plasmid	Cattle	ehxCABD	25
GCA_003754645	A	USA	342	?:NA	Plasmid	Unknown	ehxCABD	26
GCA_002766795	B1	Japan	21	O26:H11	Plasmid	Human	ehxCABD	27

GCA_900449275	E	United Kingdom	-	O157:H7	Plasmid	Unknown	ehxCABD	20
GCA_004166115	B1	United Kingdom	442	?:H21	Plasmid	Human	ehxCABD	17
GCA_001950735	E	Japan	11	O157:H7	Plasmid	Wild animals	ehxCABD	20
GCA_000619625	B1	USA	16	O111:H8	Not determined	Unknown	ehxCABD	27
GCA_003880935	E	United Kingdom	11	O157:H7	Plasmid	Human	ehxCABD	20
GCA_003757205	E	USA	11	O157:H7	Plasmid	Unknown	ehxCABD	20
GCA_001012015	B1	USA	655	O121:H19	Plasmid	Human	ehxCABD	18
GCA_001309925	E	Canada	11	O157:H7	Plasmid	Unknown	ehxCABD	20
GCA_000617965	E	USA	11	O157:H7	Plasmid	Unknown	ehxCABD	20
GCA_002176025	E	USA	11	O157:H7	Not determined	Cattle	ehxCABD	20
GCA_000615415	D	USA	32	O145:H28	Plasmid	Unknown	ehxCABD	8
GCA_004262605	E	United Kingdom	11	O157:H7	Plasmid	Human	ehxCABD	20
GCA_003915475	E	United Kingdom	11	O157:H7	Plasmid	Human	ehxCABD	20
GCA_005393545	A	Japan	10	?:H27	Not determined	Cattle	ehxCABD	24
GCA_000267365	E	USA	11	O157:H7	Plasmid	Plants	ehxCABD	20
GCA_000335055	E	Unknown	11	O157:H7	Plasmid	Unknown	ehxCABD	20
GCA_000496345	E	Unknown	11	O157:H7	Plasmid	Unknown	ehxCABD	20
GCA_002016065	B1	USA	17	O103:H2	Plasmid	Cattle	ehxCABD	15
GCA_000948815	B1	Unknown	21	O26:H11	Plasmid	Unknown	ehxCABD	27
GCA_005392205	B1	Japan	327	O177:H11	Plasmid	Cattle	ehxCABD	27
GCA_000335455	E	Unknown	11	O157:H7	Plasmid	Unknown	ehxCABD	20
GCA_001191345	B1	USA	58	O116:H21	Plasmid	Unknown	ehxCABD	22
GCA_005386205	B1	Japan	56	?:H21	Not determined	Human	ehxCABD	22
GCA_002173195	B1	USA	1817	O104:H7	Plasmid	Cattle	ehxCABD	25
GCA_002173265	B1	USA	1817	O104:H7	Not determined	Cattle	ehxCABD	25
GCA_005390885	B1	Japan	205	?:H19	Plasmid	Cattle	ehxCABD	25
GCA_002765535	B1	Japan	21	O26:H11	Plasmid	Human	ehxCABD	27
GCA_002164275	E	USA	5560	O157:H7	Plasmid	Cattle	ehxCABD	20
GCA_004234045	B1	USA	21	O26:H11	Plasmid	Unknown	ehxCABD	27
GCA_008756135	B1	USA	655	O121:H19	Plasmid	Human	ehxCABD	18
GCA_000618165	B1	USA	655	O121:H19	Plasmid	Unknown	ehxCABD	18
GCA_005392745	B1	Japan	327	O153var1:H11	Plasmid	Cattle	ehxCABD	27
GCA_005038665	B1	Canada	-	O103:H2	Not determined	Cattle	ehxCABD	15
GCA_003361275	E	Canada	11	O157:H7	Plasmid	Cattle	ehxCABD	20
GCA_002923595	B1	Japan	21	O26:H11	Plasmid	Human	ehxCABD	27
GCA_002915105	E	New Zealand	11	O157:H7	Plasmid	Wild animals	ehxCABD	20
GCA_003359415	E	Canada	11	O157:H7	Plasmid	Unknown	ehxCABD	20
GCA_000965575	B1	Norway	17	O123.O186.Gp5/O123.Gp5:H2	Plasmid	Human	ehxCABD	15
GCA_000316485	E	Unknown	11	O157:H7	Plasmid	Unknown	ehxCABD	20
GCA_005041665	B1	Canada	21	O26:H11	Plasmid	Unknown	ehxCABD	27
GCA_007644155	B1	Brazil	21	O26:H11	Plasmid	Human	ehxCABD	27
GCA_004183705	B1	United Kingdom	21	O26:H11	Plasmid	Human	ehxCABD	27
GCA_002768285	B1	Japan	21	O26:H11	Plasmid	Human	ehxCABD	27
GCA_004183355	B1	United Kingdom	33	O91:H14	Not determined	Human	ehxCABD	12
GCA_001191135	B1	USA	223	O113:H21	Plasmid	Unknown	ehxCABD	22
GCA_004273565	B1	United Kingdom	21	O123.O186.Gp5/O123.Gp5:H11	Plasmid	Human	ehxCABD	27
GCA_005041165	B1	Canada	447	O5:H19	Plasmid	Unknown	ehxCABD	25
GCA_004260985	B1	United Kingdom	21	?:H11	Plasmid	Human	ehxCABD	27
GCA_004280725	E	United Kingdom	11	O157:H7	Plasmid	Human	ehxCABD	20
GCA_003740605	E	USA	11	O157:H7	Plasmid	Unknown	ehxCABD	20
GCA_002769015	B1	Belgium	1705	O26:H11	Plasmid	Human	ehxCABD	27
GCA_002768655	B1	Japan	21	O26:H11	Plasmid	Human	ehxCABD	27
GCA_002770235	B1	Japan	21	O26:H11	Plasmid	Human	ehxCABD	27
GCA_008634185	D	Japan	32	O145:H28	Plasmid	Human	ehxCABD	8
GCA_005041155	B1	Unknown	17	O103:H2	Not determined	Human	ehxCABD	15
GCA_005044395	B1	Canada	21	O26:H11	Plasmid	Human	ehxCABD	27
GCA_003361015	E	Canada	11	O157:H7	Plasmid	Unknown	ehxCABD	20
GCA_003915515	E	United Kingdom	11	O157:H7	Plasmid	Human	ehxCABD	20
GCA_001191185	D	USA	32	O145:H28	Plasmid	Human	ehxCABD	8
GCA_003361155	E	Canada	11	O157:H7	Plasmid	Unknown	ehxCABD	20
GCA_000478705	B1	Australia	294	O111:H8	Not determined	Unknown	ehxCABD	27
GCA_001606445	D	Netherlands	32	O145:H28	Plasmid	Human	ehxCABD	8
GCA_008634365	D	Japan	32	O145:H28	Plasmid	Human	ehxCABD	8
GCA_005042655	B1	Canada	2385	?:H19	Plasmid	Unknown	ehxCABD	21
GCA_000614215	B1	USA	655	O121:H19	Plasmid	Unknown	ehxCABD	18
GCA_003880875	E	United Kingdom	11	O157:H7	Plasmid	Human	ehxCABD	20
GCA_007647045	D	Brazil	32	O145:H28	Not determined	Human	ehxCABD	8
GCA_001677555	E	Argentina	11	O157:H7	Plasmid	Human	ehxCABD	20
GCA_003360475	E	Canada	11	O157:H7	Plasmid	Human	ehxCABD	20
GCA_004161155	B1	United Kingdom	442	?:H21	Plasmid	Human	ehxCABD	17
GCA_002766375	B1	Japan	21	O26:H11	Plasmid	Human	ehxCABD	27
GCA_002531035	B1	Canada	21	O111:H11	Plasmid	Unknown	ehxCABD	27
GCA_008122425	E	United Kingdom	11	O157:H7	Plasmid	Human	ehxCABD	20
GCA_002768875	B1	Belgium	21	O26:H11	Plasmid	Human	ehxCABD	27
GCA_003359615	E	Canada	11	O157:H7	Plasmid	Unknown	ehxCABD	20
GCA_000616545	E	USA	11	O157:H7	Plasmid	Unknown	ehxCABD	20
GCA_002769415	B1	Japan	21	O26:H11	Plasmid	Human	ehxCABD	27
GCA_001884955	E	USA	11	?:H7	Plasmid	Cattle	ehxCABD	20

GCA_002194835	E	USA	11	O157:H7	Plasmid	Cattle	ehxCABD	20
GCA_003735965	B1	USA	17	O103:H2	Not determined	Unknown	ehxCABD	15
GCA_000615745	E	USA	11	O157:H7	Plasmid	Unknown	ehxCABD	20
GCA_003753605	E	USA	11	O157:H7	Plasmid	Unknown	ehxCABD	20
GCA_002462285	B1	Canada	16	O111:H8	Not determined	Human	ehxCABD	27
GCA_008755645	B1	USA	16	O111:H8	Not determined	Human	ehxCABD	27
GCA_002457955	B1	Canada	223	O113:H21	Plasmid	Human	ehxCABD	22
GCA_001262975	B1	USA	21	O103:H11	Not determined	Unknown	ehxCABD	27
GCA_003123175	A	Switzerland	301	O80:H2	Plasmid	Human	ehxCABD	19
GCA_002144095	E	Canada	11	O157:NA	Plasmid	Human	ehxCABD	20
GCA_000316505	E	Unknown	11	?:H7	Plasmid	Unknown	ehxCABD	20
GCA_001660185	E	Japan	11	O157:H7	Plasmid	Cattle	ehxCABD	20
GCA_002923655	B1	Japan	21	O26:H11	Plasmid	Human	ehxCABD	27
GCA_002554325	E	Unknown	11	O157:H7	Plasmid	Human	ehxCABD	20
GCA_004766755	B1	USA	21	O26:H11	Plasmid	Pig	ehxCABD	27
GCA_004231845	B1	USA	17	O45:H2	Not determined	Unknown	ehxCABD	15
GCA_000616005	E	USA	11	O157:H7	Plasmid	Unknown	ehxCABD	20
GCA_003418305	B1	Unknown	16	?:H8	Not determined	Unknown	ehxCABD	27
GCA_001012195	B1	USA	16	O111:H8	Not determined	Human	ehxCABD	27
GCA_001297995	E	Netherlands	11	O157:H7	Plasmid	Human	ehxCABD	20
GCA_002766055	B1	Japan	21	O26:H11	Plasmid	Human	ehxCABD	27
GCA_001191265	B1	USA	16	?:H8	Not determined	Human	ehxCABD	27
GCA_005044405	B1	Canada	135	O103:H2	Not determined	Human	ehxCABD	15
GCA_000618265	E	USA	11	O157:H7	Plasmid	Unknown	ehxCABD	20
GCA_001281985	E	Netherlands	11	O157:H7	Plasmid	Human	ehxCABD	20
GCA_005381205	B1	Japan	655	O121:H19	Plasmid	Human	ehxCABD	18
GCA_005041255	E	Canada	11	O157:H7	Plasmid	Unknown	ehxCABD	20
GCA_008633705	D	Belgium	32	O145:H28	Plasmid	Human	ehxCABD	8
GCA_002765715	B1	Japan	21	O26:H11	Plasmid	Human	ehxCABD	27
GCA_001262895	B1	USA	58	O153var1:H25	Plasmid	Unknown	ehxCABD	22
GCA_002164535	E	USA	11	O157:H7	Plasmid	Cattle	ehxCABD	20
GCA_002923855	B1	Japan	21	O26:H11	Plasmid	Human	ehxCABD	27
GCA_003884455	E	United Kingdom	11	O157:H7	Plasmid	Human	ehxCABD	20
GCA_000335015	E	Unknown	11	?:H7	Plasmid	Unknown	ehxCABD	20
GCA_002837435	B1	USA	29	O26:H11	Plasmid	Cattle	ehxCABD	27
GCA_002923985	B1	Japan	21	O26:H11	Not determined	Human	ehxCABD	27
GCA_002768955	B1	Belgium	21	O26:H11	Plasmid	Human	ehxCABD	27
GCA_004231745	E	USA	11	O157:H7	Plasmid	Unknown	ehxCABD	20
GCA_001677795	E	Argentina	11	O157:H7	Plasmid	Human	ehxCABD	20
GCA_001262965	B1	USA	154	O134.Gp6:H38	Plasmid	Unknown	ehxCABD	25
GCA_002923695	B1	Japan	21	O26:H11	Plasmid	Human	ehxCABD	27
GCA_003767395	E	USA	11	O157:H7	Plasmid	Unknown	ehxCABD	20
GCA_002765435	B1	Japan	21	O26:H11	Plasmid	Human	ehxCABD	27
GCA_002766715	B1	Japan	21	O26:H11	Plasmid	Human	ehxCABD	27
GCA_003360955	E	Canada	11	O157:NA	Plasmid	Unknown	ehxCABD	20
GCA_002770455	B1	Japan	21	O26:H11	Plasmid	Human	ehxCABD	27
GCA_003913255	E	USA	11	O157:H7	Plasmid	Unknown	ehxCABD	20
GCA_003864875	B1	Japan	655	O121:H19	Plasmid	Human	ehxCABD	18
GCA_004230825	B1	USA	723	O103:H11	Plasmid	Unknown	ehxCABD	27
GCA_002767755	B1	Japan	21	O26:H11	Plasmid	Human	ehxCABD	27
GCA_003779885	A	USA	540	O15:H40	Plasmid	Unknown	ehxCABD	23
GCA_002734965	B1	USA	29	O26:H11	Plasmid	Cattle	ehxCABD	27
GCA_005046735	B1	Canada	21	O26:H11	Plasmid	Human	ehxCABD	27
GCA_004232125	B1	USA	21	O26:H11	Not determined	Unknown	ehxCABD	27
GCA_002144065	E	Canada	11	O157:H7	Plasmid	Human	ehxCABD	20
GCA_005392805	B1	Japan	327	O177:H11	Plasmid	Cattle	ehxCABD	27
GCA_000622875	B1	USA	29	O26:H11	Plasmid	Unknown	ehxCABD	27
GCA_003122955	A	France	301	O80:H2	Not determined	Cattle	ehxCABD	19
GCA_005046865	B1	USA	350	O91:H21	Plasmid	Human	ehxCABD	17
GCA_001677615	E	Argentina	11	O157:H7	Plasmid	Human	ehxCABD	20
GCA_003878855	B1	United Kingdom	17	?:H2	Not determined	Human	ehxCABD	15
GCA_005040145	B1	Canada	723	O103:H11	Plasmid	Unknown	ehxCABD	27
GCA_000617665	E	USA	11	O157:H7	Plasmid	Unknown	ehxCABD	20
GCA_004264665	B1	United Kingdom	21	O26:H11	Plasmid	Human	ehxCABD	27
GCA_005045145	B1	Canada	2602	?:H16	Plasmid	Human	ehxCABD	25
GCA_000618185	E	USA	11	O157:H7	Plasmid	Unknown	ehxCABD	20
GCA_004273705	E	United Kingdom	11	O157:H7	Plasmid	Human	ehxCABD	20
GCA_003914415	D	USA	32	?:H28	Plasmid	Unknown	ehxCABD	8
GCA_001608055	B1	Netherlands	16	O111:H8	Not determined	Human	ehxCABD	27
GCA_002734805	B1	USA	21	O26:H11	Plasmid	Cattle	ehxCABD	27
GCA_004173635	B1	United Kingdom	442	O146:H21	Plasmid	Human	ehxCABD	17
GCA_002924225	B1	Japan	21	O26:H11	Plasmid	Cattle	ehxCABD	27
GCA_001614905	B1	China	415	O76:H19	Not determined	Sheep	ehxCABD	25
GCA_003742705	E	USA	11	?:H7	Plasmid	Unknown	ehxCABD	20
GCA_000965715	A	Norway	342	O177:H25	Plasmid	Human	ehxCABD	26
GCA_004228865	B1	USA	723	O103:H11	Plasmid	Unknown	ehxCABD	27
GCA_002765015	B1	Japan	21	O26:H11	Plasmid	Human	ehxCABD	27
GCA_008634145	D	Japan	32	O145:H28	Plasmid	Human	ehxCABD	8

GCA_002770535	B1	Japan	21	O26:H11	Plasmid	Cattle	ehxCABD	27
GCA_004232465	E	USA	11	O157:H7	Plasmid	Unknown	ehxCABD	20
GCA_002224665	E	France	11	O157:H7	Plasmid	Cattle	ehxCABD	20
GCA_000614925	B1	USA	655	O121:H19	Plasmid	Unknown	ehxCABD	18
GCA_005038345	B1	Canada	397	O22:H8	Plasmid	Cattle	ehxCABD	25
GCA_002175455	E	USA	11	O157:H7	Plasmid	Cattle	ehxCABD	20
GCA_008635545	D	Japan	32	?:H28	Plasmid	Human	ehxCABD	8
GCA_001606985	B1	Netherlands	21	O26:H11	Plasmid	Human	ehxCABD	27
GCA_001012165	B1	USA	442	O91:H21	Plasmid	Unknown	ehxCABD	17
GCA_005045315	B1	USA	19	O110:H28	Plasmid	Unknown	ehxCABD	25
GCA_004269685	E	United Kingdom	11	O157:H7	Plasmid	Human	ehxCABD	20
GCA_001432175	E	Germany	11	O157:H7	Plasmid	Unknown	ehxCABD	20
GCA_002461345	B1	Canada	223	O113:H21	Plasmid	Cattle	ehxCABD	22
GCA_003917045	B1	USA	16	?:H8	Not determined	Unknown	ehxCABD	27
GCA_003775855	D	USA	32	?:H28	Plasmid	Unknown	ehxCABD	8
GCA_002810765	B1	USA	29	O26:H11	Plasmid	Cattle	ehxCABD	27
GCA_008753535	E	USA	11	O157:H7	Plasmid	Human	ehxCABD	20
GCA_004232185	E	USA	11	O157:H7	Plasmid	Unknown	ehxCABD	20
GCA_005398505	B1	USA	17	O103:H2	Plasmid	Cattle	ehxCABD	15
GCA_005043145	B1	Canada	17	O103:H2	Not determined	Unknown	ehxCABD	15
GCA_002835175	B1	USA	29	O26:H11	Plasmid	Cattle	ehxCABD	27
GCA_008756765	G	USA	658	O185:H28	Plasmid	Human	ehxCABD	29
GCA_005044555	G	Canada	657	O183.Gp16:H18	Plasmid	Human	ehxCABD	29
GCA_004269365	E	United Kingdom	11	O157:H7	Plasmid	Human	ehxCABD	20
GCA_002530955	B1	Canada	16	O111:H8	Not determined	Unknown	ehxCABD	27
GCA_002319185	B1	Uruguay	443	O178.Gp11/O153.O178.Gp11:H19	Plasmid	Unknown	ehxCABD	25
GCA_003881435	E	United Kingdom	11	O157:H7	Plasmid	Human	ehxCABD	20
GCA_005392325	B1	Japan	205	O178.Gp11/O153.O178.Gp11:H19	Plasmid	Cattle	ehxCABD	25
GCA_002474185	E	USA	11	O157:H7	Plasmid	Unknown	ehxCABD	20
GCA_002768635	B1	Japan	21	O26:H11	Plasmid	Human	ehxCABD	27
GCA_001606915	B1	Netherlands	6043	O181:H49	Plasmid	Human	ehxCABD	25
GCA_002835135	B1	USA	29	O26:H11	Plasmid	Cattle	ehxCABD	27
GCA_000215145	B1	Unknown	2385	?:H19	Plasmid	Cattle	ehxCABD	21
GCA_000462825	E	Unknown	11	O157:H7	Plasmid	Unknown	ehxCABD	20
GCA_000351685	G	Denmark	738	O146:H28	Plasmid	Human	ehxCABD	29
GCA_000617585	A	USA	119	O165:H25	Plasmid	Unknown	ehxCABD	26
GCA_005395705	B1	Japan	300	?:H25	Not determined	Cattle	ehxCABD	14
GCA_000462485	E	Unknown	11	O157:H7	Plasmid	Unknown	ehxCABD	20
GCA_000462385	E	Unknown	11	O157:H7	Plasmid	Unknown	ehxCABD	20
GCA_008633885	D	Japan	32	O145:H28	Plasmid	Human	ehxCABD	8
GCA_003757825	E	USA	11	O157:H7	Plasmid	Unknown	ehxCABD	20
GCA_003122805	A	France	301	O80:H2	Plasmid	Cattle	ehxCABD	19
GCA_003883855	B1	United Kingdom	17	O4:H2	Not determined	Human	ehxCABD	15
GCA_002164355	E	USA	5560	O157:H7	Plasmid	Cattle	ehxCABD	20
GCA_000462105	E	Unknown	11	O157:H7	Plasmid	Unknown	ehxCABD	20
GCA_000614845	B1	USA	21	O26:H11	Plasmid	Unknown	ehxCABD	27
GCA_005039305	B1	Canada	21	O26:H11	Plasmid	Cattle	ehxCABD	27
GCA_001606695	B1	Netherlands	17	O103:H2	Not determined	Human	ehxCABD	15
GCA_004164935	B1	United Kingdom	21	O26:H11	Plasmid	Human	ehxCABD	27
GCA_004264505	A	United Kingdom	659	O177:H25	Plasmid	Human	ehxCABD	26
GCA_005043575	B1	Canada	17	O103:H2	Not determined	Unknown	ehxCABD	15
GCA_000462225	E	Unknown	11	O157:H7	Plasmid	Unknown	ehxCABD	20
GCA_008633625	D	Belgium	32	O145:H28	Plasmid	Human	ehxCABD	8
GCA_004230985	E	USA	11	?:H7	Plasmid	Unknown	ehxCABD	20
GCA_002766615	B1	Japan	21	O26:H11	Not determined	Human	ehxCABD	27
GCA_002769855	B1	Japan	21	O26:H11	Plasmid	Human	ehxCABD	27
GCA_900449705	B1	United Kingdom	-	O26:H11	Plasmid	Unknown	ehxCABD	27
GCA_002164175	E	USA	11	O157:H7	Plasmid	Unknown	ehxCABD	20
GCA_005045805	B1	Canada	9053	O75:H8	Plasmid	Human	ehxCABD	25
GCA_005038265	B1	Canada	524	O93:H28	Plasmid	Unknown	ehxCABD	25
GCA_002835095	B1	USA	29	O26:H11	Plasmid	Cattle	ehxCABD	27
GCA_002765035	B1	Japan	21	O26:H11	Plasmid	Human	ehxCABD	27
GCA_003882735	B1	United Kingdom	675	?:H19	Not determined	Human	ehxCABD	25
GCA_002765975	B1	Japan	21	O26:H11	Plasmid	Human	ehxCABD	27
GCA_001607365	B1	Netherlands	415	O76:H19	Plasmid	Human	ehxCABD	25
GCA_003362615	E	Canada	11	O157:H7	Plasmid	Human	ehxCABD	20
GCA_005390265	B1	Japan	357	?:H7	Plasmid	Cattle	ehxCABD	25
GCA_002764295	B1	Belgium	21	O26:H11	Plasmid	Cattle	ehxCABD	27
GCA_001012395	A	USA	660	O172:H25	Plasmid	Unknown	ehxCABD	26
GCA_004181795	B1	United Kingdom	306	O84:H2	Plasmid	Human	ehxCABD	14
GCA_004257445	B1	United Kingdom	21	O26:H11	Plasmid	Human	ehxCABD	27
GCA_004230505	E	USA	11	O157:H7	Plasmid	Unknown	ehxCABD	20
GCA_000618105	E	USA	11	O157:H7	Plasmid	Unknown	ehxCABD	20
GCA_001297975	E	Netherlands	11	O157:H7	Plasmid	Human	ehxCABD	20
GCA_004164535	B1	United Kingdom	675	O76:H19	Not determined	Human	ehxCABD	25
GCA_002769795	B1	Japan	21	O26:H11	Plasmid	Human	ehxCABD	27
GCA_004173615	E	United Kingdom	11	O157:H7	Plasmid	Human	ehxCABD	20
GCA_001571805	B1	USA	29	O26:H11	Plasmid	Cattle	ehxCABD	27

GCA_002520985	A	USA	206	O49:H10	Not determined	Cattle	ehxCABD	23
GCA_005396945	A	Japan	301	O180:H2	Plasmid	Cattle	ehxCABD	19
GCA_000948875	B1	Unknown	655	O121:H19	Plasmid	Unknown	ehxCABD	18
GCA_003907275	E	United Kingdom	11	O157:H7	Plasmid	Human	ehxCABD	20
GCA_002144055	E	Canada	11	O157:H7	Plasmid	Human	ehxCABD	20
GCA_005043105	B1	Canada	21	O26:H11	Plasmid	Unknown	ehxCABD	27
GCA_002767725	B1	Japan	21	O26:H11	Plasmid	Human	ehxCABD	27
GCA_003763965	B1	USA	17	?:H2	Plasmid	Unknown	ehxCABD	15
GCA_003362495	E	Canada	11	O157:H7	Plasmid	Human	ehxCABD	20
GCA_005045665	B1	Canada	481	O26:H11	Plasmid	Human	ehxCABD	27
GCA_008633585	D	Japan	32	O145:H28	Plasmid	Human	ehxCABD	8
GCA_000618945	E	USA	11	O157:H7	Plasmid	Unknown	ehxCABD	20
GCA_000615925	E	USA	11	O157:H7	Plasmid	Unknown	ehxCABD	20
GCA_004263105	E	United Kingdom	11	O157:H7	Plasmid	Human	ehxCABD	20
GCA_002834495	B1	USA	29	O26:H11	Plasmid	Cattle	ehxCABD	27
GCA_002770115	B1	Japan	21	O26:H11	Plasmid	Human	ehxCABD	27
GCA_002764615	B1	Japan	21	O26:H11	Plasmid	Human	ehxCABD	27
GCA_005041295	A	Canada	342	?:NA	Plasmid	Unknown	ehxCABD	26
GCA_004177165	B1	United Kingdom	811	O128:H2	Plasmid	Human	ehxCABD	13
GCA_001039135	B1	France	29	O26:H11	Plasmid	Human	ehxCABD	27
GCA_000462765	E	Unknown	11	O157:H7	Plasmid	Unknown	ehxCABD	20
GCA_003907345	E	United Kingdom	11	?:H7	Plasmid	Human	ehxCABD	20
GCA_001607605	B1	Netherlands	370	O146:H10	Plasmid	Human	ehxCABD	25
GCA_002486685	E	USA	11	O157:H7	Plasmid	Unknown	ehxCABD	20
GCA_004158395	D	United Kingdom	32	?:H28	Plasmid	Human	ehxCABD	8
GCA_004165655	A	United Kingdom	301	O80:H2	Not determined	Human	ehxCABD	19
GCA_002770495	B1	Japan	21	O26:H11	Plasmid	Cattle	ehxCABD	27
GCA_003768925	E	USA	11	O157:H7	Plasmid	Unknown	ehxCABD	20
GCA_003921295	E	United Kingdom	11	O157:H7	Plasmid	Human	ehxCABD	20
GCA_001884975	E	USA	11	O157:H7	Plasmid	Cattle	ehxCABD	20
GCA_003362075	E	Canada	11	O157:H7	Plasmid	Human	ehxCABD	20
GCA_005046365	B1	Germany	25	O128:H2	Plasmid	Human	ehxCABD	13
GCA_005045575	B1	Canada	17	O103:H2	Plasmid	Human	ehxCABD	15
GCA_003893975	B1	United Kingdom	21	O26:H11	Plasmid	Human	ehxCABD	27
GCA_002769435	B1	Japan	21	O26:H11	Plasmid	Human	ehxCABD	27
GCA_003758605	B1	USA	21	O151.Gp3/O118.Gp3:H16	Plasmid	Unknown	ehxCABD	27
GCA_003741065	E	USA	11	O157:H7	Plasmid	Unknown	ehxCABD	20
GCA_003757785	E	USA	11	O157:H7	Plasmid	Unknown	ehxCABD	20
GCA_000617765	D	USA	32	O145:H28	Plasmid	Unknown	ehxCABD	8
GCA_004766775	E	USA	11	O157:H7	Plasmid	Pig	ehxCABD	20
GCA_001606355	D	Netherlands	32	O145:H28	Plasmid	Human	ehxCABD	8
GCA_004233055	E	USA	11	O157:H7	Plasmid	Unknown	ehxCABD	20
GCA_003893935	B1	United Kingdom	21	O26:H11	Plasmid	Human	ehxCABD	27
GCA_002769815	B1	Japan	21	O26:H11	Plasmid	Human	ehxCABD	27
GCA_000462185	E	Unknown	11	O157:H7	Plasmid	Unknown	ehxCABD	20
GCA_004231885	B1	USA	17	O103:H2	Not determined	Unknown	ehxCABD	15
GCA_004231965	E	USA	5516	?:H7	Plasmid	Unknown	ehxCABD	20
GCA_005040335	B1	Canada	223	?:H21	Plasmid	Unknown	ehxCABD	22
GCA_004175435	E	United Kingdom	628	?:H7	Plasmid	Human	ehxCABD	20
GCA_002764215	B1	Belgium	21	O26:H11	Plasmid	Human	ehxCABD	27
GCA_004162125	B1	United Kingdom	8296	O43:H2	Plasmid	Human	ehxCABD	25
GCA_005046675	B1	Canada	17	O103:H2	Not determined	Human	ehxCABD	15
GCA_005044105	B1	Canada	5536	O121:H19	Plasmid	Human	ehxCABD	18
GCA_004234415	E	USA	11	O157:H7	Plasmid	Unknown	ehxCABD	20
GCA_004264205	Unknown	United Kingdom	1819	O166:H28	Plasmid	Human	ehxCABD	23
GCA_003027135	E	Austria	11	?:H7	Plasmid	Goat	ehxCABD	20
GCA_002144185	B1	Canada	21	O26:H11	Plasmid	Human	ehxCABD	27
GCA_002509905	B1	USA	16	O111:H8	Not determined	Human	ehxCABD	27
GCA_005042995	B1	Canada	350	O91:H21	Plasmid	Cattle	ehxCABD	17
GCA_004796605	D	USA	32	O145:H28	Plasmid	Cattle	ehxCABD	8
GCA_004232145	E	USA	11	O157:H7	Plasmid	Unknown	ehxCABD	20
GCA_005391085	B1	Japan	-	?:H25	Plasmid	Cattle	ehxCABD	22
GCA_005038355	B1	Canada	21	O26:H11	Plasmid	Cattle	ehxCABD	27
GCA_003360835	E	Canada	11	O157:H7	Plasmid	Unknown	ehxCABD	20
GCA_003362635	E	Canada	11	O157:H7	Plasmid	Human	ehxCABD	20
GCA_003754625	E	USA	11	?:H7	Plasmid	Unknown	ehxCABD	20
GCA_008757195	B1	USA	17	O103:H2	Not determined	Human	ehxCABD	15
GCA_003921585	B1	USA	21	O26:H11	Plasmid	Unknown	ehxCABD	27
GCA_003740925	B1	USA	1792	O111:H8	Not determined	Unknown	ehxCABD	27
GCA_001607655	B1	Netherlands	25	O128:H2	Plasmid	Human	ehxCABD	13
GCA_003361675	E	Canada	11	O157:H7	Plasmid	Cattle	ehxCABD	20
GCA_001309635	B1	Canada	17	O45:H2	Not determined	Human	ehxCABD	15
GCA_005390905	B1	Japan	300	?:H25	Not determined	Cattle	ehxCABD	14
GCA_000617005	B1	USA	655	O121:H19	Plasmid	Unknown	ehxCABD	18
GCA_002133695	B1	Canada	16	?:H8	Not determined	Unknown	ehxCABD	27
GCA_005046905	B1	Canada	21	O26:H11	Plasmid	Human	ehxCABD	27
GCA_002765695	B1	Japan	21	O26:H11	Plasmid	Human	ehxCABD	27
GCA_000155125	E	Unknown	11	O157:H7	Plasmid	Unknown	ehxCABD	20

GCA_002473795	E	USA	11	O157:H7	Plasmid	Unknown	ehxCABD	20
GCA_002133505	B1	Canada	350	O91:H21	Plasmid	Unknown	ehxCABD	17
GCA_003761245	E	USA	11	?:H7	Plasmid	Unknown	ehxCABD	20
GCA_004270725	E	United Kingdom	11	O157:H7	Plasmid	Human	ehxCABD	20
GCA_000335435	E	Unknown	11	O157:H7	Plasmid	Unknown	ehxCABD	20
GCA_003879015	E	United Kingdom	11	O157:H7	Plasmid	Human	ehxCABD	20
GCA_002462215	B1	USA	58	O116:H21	Plasmid	Human	ehxCABD	22
GCA_000614765	B1	USA	655	O121:H19	Plasmid	Unknown	ehxCABD	18
GCA_001191395	B1	USA	17	O103:H2	Plasmid	Human	ehxCABD	15
GCA_005392485	A	Japan	206	O109:H10	Not determined	Cattle	ehxCABD	23
GCA_003763725	E	USA	11	O157:H7	Plasmid	Unknown	ehxCABD	20
GCA_004164995	B1	United Kingdom	29	O26:H11	Plasmid	Human	ehxCABD	27
GCA_004176455	E	United Kingdom	11	?:H7	Plasmid	Human	ehxCABD	20
GCA_000617565	B1	USA	655	O121:H19	Plasmid	Unknown	ehxCABD	18
GCA_005041765	B1	Canada	2385	?:H19	Plasmid	Unknown	ehxCABD	21
GCA_000215205	B1	Unknown	17	O103:H2	Plasmid	Horse	ehxCABD	15
GCA_005392665	D	Japan	32	O145:H28	Plasmid	Cattle	ehxCABD	8
GCA_005043705	B1	Canada	17	O103:H2	Not determined	Unknown	ehxCABD	15
GCA_002133985	B1	Canada	17	O103:H2	Not determined	Unknown	ehxCABD	15
GCA_004160215	E	United Kingdom	11	?:H7	Plasmid	Human	ehxCABD	20
GCA_001039125	B1	France	21	O26:H11	Plasmid	Human	ehxCABD	27
GCA_003362575	E	Canada	11	O157:H7	Plasmid	Human	ehxCABD	20
GCA_001191295	B1	USA	1817	O104:H7	Plasmid	Unknown	ehxCABD	25
GCA_005041095	E	Canada	724	Onovel3:H20	Plasmid	Human	ehxCABD	11
GCA_000617485	B1	USA	480	O111:H8	Not determined	Unknown	ehxCABD	27
GCA_002476045	E	USA	11	O157:H7	Plasmid	Unknown	ehxCABD	20
GCA_001616735	B1	China	13	O174:H8	Plasmid	Sheep	ehxCABD	25
GCA_900449115	E	United Kingdom	-	O157:H7	Plasmid	Unknown	ehxCABD	20
GCA_002766575	B1	Japan	21	O26:H11	Plasmid	Human	ehxCABD	27
GCA_003740725	B1	USA	17	?:H2	Not determined	Unknown	ehxCABD	15
GCA_002735105	B1	France	481	O26:H11	Plasmid	Human	ehxCABD	27
GCA_005392645	A	Japan	206	O35:H10	Not determined	Cattle	ehxCABD	23
GCA_005394845	B1	Japan	327	O70:H11	Plasmid	Cattle	ehxCABD	27
GCA_005044725	B1	Canada	17	O103:H2	Plasmid	Unknown	ehxCABD	15
GCA_005397825	A	USA	325	O15:H16	Plasmid	Cattle	ehxCABD	23
GCA_004258045	E	United Kingdom	11	O157:H7	Plasmid	Human	ehxCABD	20
GCA_000615785	E	USA	11	O157:H7	Plasmid	Unknown	ehxCABD	20
GCA_004174975	B1	United Kingdom	278	O178.Gp11/O153.O178.Gp11:H7	Plasmid	Human	ehxCABD	25
GCA_003113195	B1	Japan	29	O26:H11	Plasmid	Human	ehxCABD	27
GCA_000616445	E	USA	11	O157:H7	Plasmid	Unknown	ehxCABD	20
GCA_002195805	E	USA	11	O157:H7	Plasmid	Cattle	ehxCABD	20
GCA_002519505	D	USA	32	?:H28	Not determined	Cattle	ehxCABD	8
GCA_004289035	A	United Kingdom	10	O113:H4	Plasmid	Human	ehxCABD	24
GCA_001282065	E	Netherlands	11	O157:H7	Plasmid	Human	ehxCABD	20
GCA_003361435	E	Canada	11	O157:H7	Plasmid	Unknown	ehxCABD	20
GCA_000462565	E	Unknown	11	O157:H7	Plasmid	Unknown	ehxCABD	20
GCA_003360865	E	Canada	11	O157:H7	Plasmid	Unknown	ehxCABD	20
GCA_003756025	E	USA	11	?:H7	Plasmid	Unknown	ehxCABD	20
GCA_002473945	E	USA	11	O157:H7	Plasmid	Unknown	ehxCABD	20
GCA_001677855	E	Argentina	628	O157:H7	Plasmid	Human	ehxCABD	20
GCA_008756205	B1	USA	16	?:H8	Not determined	Human	ehxCABD	27
GCA_002286515	B1	Israel	481	O26:H11	Plasmid	Human	ehxCABD	27
GCA_002766595	B1	Japan	21	O26:H11	Plasmid	Human	ehxCABD	27
GCA_005045135	B1	USA	7616	O6:H34	Plasmid	Unknown	ehxCABD	25
GCA_002175885	E	USA	11	O157:H7	Not determined	Cattle	ehxCABD	20
GCA_008634345	D	Japan	32	O145:H28	Plasmid	Human	ehxCABD	8
GCA_004234445	E	USA	11	O157:H7	Plasmid	Unknown	ehxCABD	20
GCA_003755825	E	USA	11	O157:H7	Plasmid	Unknown	ehxCABD	20
GCA_000965705	B1	Norway	29	O26:H11	Plasmid	Human	ehxCABD	27
GCA_003918825	E	USA	11	?:H7	Plasmid	Unknown	ehxCABD	20
GCA_004232715	B1	USA	16	O111:H8	Not determined	Unknown	ehxCABD	27
GCA_004228915	E	USA	11	O157:H7	Plasmid	Unknown	ehxCABD	20
GCA_003914195	B1	United Kingdom	21	O26:H11	Plasmid	Human	ehxCABD	27
GCA_002770155	B1	Japan	21	O26:H11	Plasmid	Human	ehxCABD	27
GCA_003739505	E	USA	11	O157:H7	Plasmid	Unknown	ehxCABD	20
GCA_005045995	B1	Switzerland	16	O111:NA	Not determined	Human	ehxCABD	27
GCA_004255505	E	United Kingdom	11	O157:H7	Plasmid	Human	ehxCABD	20
GCA_005040735	B1	Canada	343	O103:H25	Not determined	Unknown	ehxCABD	14
GCA_004158115	E	United Kingdom	11	O157:H7	Plasmid	Human	ehxCABD	20
GCA_002767595	B1	Japan	21	O26:H11	Plasmid	Human	ehxCABD	27
GCA_001606515	B1	Netherlands	25	O128:H2	Plasmid	Human	ehxCABD	13
GCA_002834355	B1	USA	29	?:H11	Plasmid	Cattle	ehxCABD	27
GCA_003362115	E	Canada	11	O157:H7	Plasmid	Human	ehxCABD	20
GCA_003919895	D	USA	-	?:H28	Plasmid	Unknown	ehxCABD	8
GCA_008636025	D	Japan	32	?:H28	Plasmid	Human	ehxCABD	8
GCA_003113695	B1	Japan	481	O26:H11	Plasmid	Human	ehxCABD	27
GCA_009647465	E	China	799	O108:H9	Not determined	Dog	ehxCABD	23
GCA_002458655	D	USA	32	O145:H28	Plasmid	Human	ehxCABD	8

GCA_002195195	E	USA	11	O157:H7	Plasmid	Cattle	ehxCABD	20
GCA_000616685	B1	USA	16	O111:H8	Not determined	Unknown	ehxCABD	27
GCA_002734865	B1	USA	21	O26:H11	Plasmid	Cattle	ehxCABD	27
GCA_001677645	E	Argentina	11	O157:H7	Plasmid	Human	ehxCABD	20
GCA_002531275	E	Canada	11	O157:H7	Plasmid	Unknown	ehxCABD	20
GCA_005045385	B1	Canada	300	O182:H25	Not determined	Unknown	ehxCABD	14
GCA_004256725	B1	United Kingdom	737	?:H21	Plasmid	Human	ehxCABD	25
GCA_003361735	E	Canada	11	O157:H7	Plasmid	Unknown	ehxCABD	20
GCA_003361655	E	Canada	11	O157:H7	Plasmid	Unknown	ehxCABD	20
GCA_003361465	E	Canada	11	O157:H7	Plasmid	Unknown	ehxCABD	20
GCA_003739585	B1	USA	480	O111:H8	Not determined	Unknown	ehxCABD	27
GCA_003740545	B1	USA	17	O103:H2	Not determined	Unknown	ehxCABD	15
GCA_003741085	E	USA	11	O157:H7	Plasmid	Unknown	ehxCABD	20
GCA_005045815	B1	Canada	300	O156:H25	Not determined	Human	ehxCABD	14
GCA_002734945	B1	USA	21	O26:H11	Plasmid	Cattle	ehxCABD	27
GCA_003770165	E	USA	11	?:H7	Plasmid	Unknown	ehxCABD	20
GCA_000335155	E	Unknown	11	O157:H7	Plasmid	Unknown	ehxCABD	20
GCA_003361375	E	Canada	11	O157:H7	Plasmid	Unknown	ehxCABD	20
GCA_002176505	E	USA	11	?:H7	Plasmid	Cattle	ehxCABD	20
GCA_004255945	E	United Kingdom	11	O157:H7	Plasmid	Human	ehxCABD	20
GCA_005037985	B1	Canada	21	O26:H11	Plasmid	Unknown	ehxCABD	27
GCA_003759805	D	USA	32	?:H28	Plasmid	Unknown	ehxCABD	8
GCA_003360555	E	Canada	11	O157:H7	Plasmid	Human	ehxCABD	20
GCA_002173115	B1	USA	1817	?:H7	Not determined	Cattle	ehxCABD	25
GCA_003113675	B1	Japan	21	O26:H11	Plasmid	Human	ehxCABD	27
GCA_000614725	B1	USA	655	O121:H19	Plasmid	Unknown	ehxCABD	18
GCA_005391065	B1	Japan	205	?:H19	Plasmid	Cattle	ehxCABD	25
GCA_002764755	B1	Japan	21	O26:H11	Plasmid	Human	ehxCABD	27
GCA_000462705	E	Unknown	11	O157:H7	Plasmid	Unknown	ehxCABD	20
GCA_002769375	B1	Japan	21	O26:H11	Plasmid	Human	ehxCABD	27
GCA_003027415	E	Austria	11	?:H7	Plasmid	Sheep	ehxCABD	20
GCA_000408445	A	Denmark	330	?:H33	Not determined	Human	ehxCABD	24
GCA_002766135	B1	Japan	21	O26:H11	Plasmid	Human	ehxCABD	27
GCA_002134625	B1	Canada	21	?:H11	Plasmid	Unknown	ehxCABD	27
GCA_004281135	E	United Kingdom	11	?:H7	Plasmid	Human	ehxCABD	20
GCA_000215185	B1	Unknown	350	O91:H21	Plasmid	Human	ehxCABD	17
GCA_002476005	E	USA	11	O157:H7	Plasmid	Unknown	ehxCABD	20
GCA_008753455	E	USA	11	O157:H7	Plasmid	Human	ehxCABD	20
GCA_001012635	B1	USA	17	O103:H2	Not determined	Unknown	ehxCABD	15
GCA_003908535	A	United Kingdom	1889	O10:H25	Not determined	Human	ehxCABD	26
GCA_004766745	E	USA	11	O157:H7	Plasmid	Pig	ehxCABD	20
GCA_003362355	E	Canada	11	O157:H7	Plasmid	Human	ehxCABD	20
GCA_004796715	D	USA	32	O145:H28	Plasmid	Cattle	ehxCABD	8
GCA_000619645	B1	USA	16	O111:H8	Not determined	Unknown	ehxCABD	27
GCA_004263405	E	United Kingdom	11	O157:H7	Plasmid	Human	ehxCABD	20
GCA_002765195	B1	Japan	21	O26:H11	Not determined	Human	ehxCABD	27
GCA_004181805	E	United Kingdom	11	O157:H7	Plasmid	Human	ehxCABD	20
GCA_005045415	A	Denmark	10	O38:H26	Plasmid	Human	ehxCABD	24
GCA_000618145	E	USA	11	O157:H7	Plasmid	Unknown	ehxCABD	20
GCA_003887335	E	United Kingdom	11	O157:H7	Plasmid	Human	ehxCABD	20
GCA_003736345	B1	USA	16	?:H8	Not determined	Unknown	ehxCABD	27
GCA_002319415	B1	Uruguay	679	O163:H19	Plasmid	Unknown	ehxCABD	10
GCA_001677785	E	Argentina	628	O157:H7	Plasmid	Human	ehxCABD	20
GCA_004175415	E	United Kingdom	11	O157:H7	Plasmid	Human	ehxCABD	20
GCA_001607185	B1	Netherlands	300	O182:H25	Not determined	Human	ehxCABD	14
GCA_002164095	E	USA	11	O157:H7	Plasmid	Cattle	ehxCABD	20
GCA_001191435	B1	USA	723	O103:H11	Plasmid	Human	ehxCABD	27
GCA_003901435	B1	United Kingdom	675	O76:H19	Plasmid	Human	ehxCABD	25
GCA_002195175	E	USA	11	O157:H7	Plasmid	Cattle	ehxCABD	20
GCA_002769135	B1	Belgium	1705	O26:H11	Plasmid	Human	ehxCABD	27
GCA_003419045	B1	Unknown	655	O121:H19	Plasmid	Unknown	ehxCABD	18
GCA_005392585	A	Japan	206	O35:H10	Not determined	Cattle	ehxCABD	23
GCA_005383905	A	Japan	10	O2.Gp7/O2.O50.Gp7:H27	Not determined	Cattle	ehxCABD	24
GCA_005389865	B1	Japan	336	?:H16	Plasmid	Cattle	ehxCABD	25
GCA_000616265	B1	USA	16	O111:H8	Not determined	Unknown	ehxCABD	27
GCA_002379285	B1	USA	5975	O113:H21	Plasmid	Unknown	ehxCABD	22
GCA_002769555	B1	Japan	21	O26:H11	Plasmid	Human	ehxCABD	27
GCA_002923615	B1	Japan	21	O26:H11	Plasmid	Human	ehxCABD	27
GCA_003735545	B1	USA	17	O103:H2	Plasmid	Unknown	ehxCABD	15
GCA_000462125	E	Unknown	11	O157:H7	Plasmid	Unknown	ehxCABD	20
GCA_002473655	E	USA	11	O157:H7	Plasmid	Unknown	ehxCABD	20
GCA_001012475	B1	USA	1817	O104:H7	Plasmid	Unknown	ehxCABD	25
GCA_003919575	E	United Kingdom	11	O157:H7	Plasmid	Human	ehxCABD	20
GCA_004281235	E	United Kingdom	11	O157:H7	Plasmid	Human	ehxCABD	20
GCA_004259865	E	United Kingdom	11	O157:H7	Plasmid	Human	ehxCABD	20
GCA_005043655	B1	Canada	17	O103:H2	Not determined	Unknown	ehxCABD	15
GCA_002144105	E	Canada	11	O157:H7	Plasmid	Human	ehxCABD	20
GCA_005395005	B1	Japan	-	?:H19	Plasmid	Cattle	ehxCABD	10

GCA_003359735	E	Canada	11	O157:H7	Plasmid	Human	ehxCABD	20
GCA_001467005	B1	South Korea	111	?:H49	Plasmid	Human	ehxCABD	25
GCA_003113035	B1	Japan	29	?:H11	Plasmid	Human	ehxCABD	27
GCA_005046665	B1	Canada	17	O103:H2	Plasmid	Cattle	ehxCABD	15
GCA_005394885	B1	Japan	679	O163:H19	Plasmid	Cattle	ehxCABD	10
GCA_002765055	B1	Japan	21	O26:H11	Plasmid	Human	ehxCABD	27
GCA_004261185	E	United Kingdom	11	O157:H7	Plasmid	Human	ehxCABD	20
GCA_002765595	B1	Japan	21	O26:H11	Plasmid	Human	ehxCABD	27
GCA_003767085	B1	USA	16	?:H8	Not determined	Unknown	ehxCABD	27
GCA_003742745	B1	USA	21	?:H11	Plasmid	Unknown	ehxCABD	27
GCA_000462885	E	Unknown	11	O157:H7	Plasmid	Unknown	ehxCABD	20
GCA_003917035	B1	USA	21	O26:H11	Plasmid	Unknown	ehxCABD	27
GCA_005040845	B1	Canada	306	O98:H21	Plasmid	Unknown	ehxCABD	14
GCA_002769335	B1	Japan	21	O26:H11	Plasmid	Human	ehxCABD	27
GCA_003756525	B1	USA	17	O45:H2	Not determined	Unknown	ehxCABD	15
GCA_003362265	E	Canada	11	O157:H7	Plasmid	Cattle	ehxCABD	20
GCA_004254845	E	United Kingdom	11	O157:H7	Plasmid	Human	ehxCABD	20
GCA_002515545	B1	USA	4496	?:H28	Not determined	Unknown	ehxCABD	21
GCA_001607105	B1	Netherlands	16	O111:H8	Not determined	Human	ehxCABD	27
GCA_002734765	B1	USA	21	O26:H11	Plasmid	Cattle	ehxCABD	27
GCA_005042955	B1	Canada	223	?:H21	Plasmid	Cattle	ehxCABD	22
GCA_004159475	A	United Kingdom	10	O38:H26	Plasmid	Human	ehxCABD	24
GCA_001677875	E	Argentina	11	O157:H7	Plasmid	Human	ehxCABD	20
GCA_003882595	E	United Kingdom	11	O157:H7	Plasmid	Human	ehxCABD	20
GCA_002767305	B1	Japan	21	O26:H11	Plasmid	Human	ehxCABD	27
GCA_008753655	E	USA	11	?:H7	Plasmid	Human	ehxCABD	20
GCA_003887615	E	United Kingdom	11	O157:H7	Plasmid	Human	ehxCABD	20
GCA_000462625	E	Unknown	11	O157:H7	Plasmid	Unknown	ehxCABD	20
GCA_002486645	E	USA	11	O157:H7	Plasmid	Unknown	ehxCABD	20
GCA_002164015	E	USA	11	O157:H7	Plasmid	Cattle	ehxCABD	20
GCA_003361035	E	Canada	11	O157:H7	Plasmid	Unknown	ehxCABD	20
GCA_003918195	E	United Kingdom	11	O157:H7	Plasmid	Human	ehxCABD	20
GCA_004797745	D	USA	32	?:H28	Not determined	Cattle	ehxCABD	8
GCA_000617345	D	USA	32	O145:H28	Plasmid	Unknown	ehxCABD	8
GCA_002164375	E	USA	11	O157:H7	Plasmid	Cattle	ehxCABD	20
GCA_000260475	E	South Korea	11	O157:H7	Plasmid	Human	ehxCABD	20
GCA_005044315	B1	Canada	481	O26:H11	Plasmid	Unknown	ehxCABD	27
GCA_005392125	B1	Japan	443	O178.Gp11/O153.O178.Gp11:H19	Plasmid	Cattle	ehxCABD	25
GCA_005037915	B1	Unknown	16	O111:H8	Not determined	Cattle	ehxCABD	27
GCA_005043215	E	Canada	9027	?:H20	Plasmid	Unknown	ehxCABD	11
GCA_001606785	B1	Netherlands	25	O128:H2	Plasmid	Human	ehxCABD	13
GCA_005046455	A	Switzerland	342	O145:H25	Not determined	Human	ehxCABD	26
GCA_002768015	B1	Japan	21	O26:H11	Plasmid	Human	ehxCABD	27
GCA_003776525	E	USA	11	O157:H7	Plasmid	Unknown	ehxCABD	20
GCA_002765935	B1	Japan	21	O26:H11	Plasmid	Human	ehxCABD	27
GCA_005044275	B1	Canada	343	O103:H25	Not determined	Human	ehxCABD	14
GCA_004165935	E	United Kingdom	11	O157:H7	Plasmid	Human	ehxCABD	20
GCA_003899885	B1	USA	21	O26:H11	Plasmid	Unknown	ehxCABD	27
GCA_008634065	D	Japan	32	O145:H28	Plasmid	Human	ehxCABD	8
GCA_003864795	B1	Japan	655	O121:H19	Plasmid	Human	ehxCABD	18
GCA_005044225	B1	Canada	17	O103:H2	Not determined	Human	ehxCABD	15
GCA_002175575	E	USA	11	O157:H7	Plasmid	Cattle	ehxCABD	20
GCA_002924025	B1	Japan	21	O26:H11	Plasmid	Human	ehxCABD	27
GCA_003761465	B1	USA	21	?:H11	Plasmid	Unknown	ehxCABD	27
GCA_002530875	B1	Canada	16	O111:H8	Not determined	Unknown	ehxCABD	27
GCA_002810955	B1	USA	29	O26:H11	Plasmid	Cattle	ehxCABD	27
GCA_005044455	B1	Canada	343	O103:H25	Not determined	Human	ehxCABD	14
GCA_003360135	E	Canada	11	O157:H7	Plasmid	Human	ehxCABD	20
GCA_000316545	E	Unknown	11	O157:H7	Plasmid	Unknown	ehxCABD	20
GCA_004165795	E	United Kingdom	11	O157:H7	Plasmid	Human	ehxCABD	20
GCA_003924275	B1	USA	16	?:H8	Not determined	Unknown	ehxCABD	27
GCA_008634325	D	Japan	32	O145:H28	Plasmid	Human	ehxCABD	8
GCA_002923455	B1	USA	21	O26:H11	Plasmid	Cattle	ehxCABD	27
GCA_002768995	B1	Belgium	21	O26:H11	Plasmid	Human	ehxCABD	27
GCA_003883195	B1	United Kingdom	29	?:H11	Plasmid	Human	ehxCABD	27
GCA_003359515	E	Canada	11	O157:H7	Plasmid	Unknown	ehxCABD	20
GCA_005392955	B1	Japan	327	O177:H11	Plasmid	Cattle	ehxCABD	27
GCA_004767115	E	USA	11	O157:H7	Plasmid	Pig	ehxCABD	20
GCA_005394865	B1	Japan	297	?:H11	Plasmid	Cattle	ehxCABD	25
GCA_003765465	B1	USA	17	O103:H2	Not determined	Unknown	ehxCABD	15
GCA_004233865	E	USA	11	?:H7	Plasmid	Unknown	ehxCABD	20
GCA_005395685	B1	Japan	101	?:H8	Plasmid	Cattle	ehxCABD	25
GCA_003361065	E	Canada	11	O157:H7	Plasmid	Unknown	ehxCABD	20
GCA_000616585	E	USA	11	O157:H7	Plasmid	Unknown	ehxCABD	20
GCA_005038675	B1	Unknown	17	O103:H2	Not determined	Human	ehxCABD	15
GCA_004230685	D	USA	32	O145:H28	Plasmid	Unknown	ehxCABD	8
GCA_002770315	B1	Japan	21	O26:H11	Plasmid	Human	ehxCABD	27
GCA_005039205	B1	Canada	21	O111:H11	Not determined	Cattle	ehxCABD	27

GCA_004158235	E	United Kingdom	11	O157:H7	Plasmid	Human	ehxCABD	20
GCA_005044145	B1	Canada	-	O121:H19	Plasmid	Human	ehxCABD	18
GCA_000614645	E	USA	11	O157:H7	Plasmid	Unknown	ehxCABD	20
GCA_004173675	E	United Kingdom	11	O157:H7	Plasmid	Human	ehxCABD	20
GCA_005044645	B1	Denmark	2103	O48:H21	Plasmid	Unknown	ehxCABD	25
GCA_003757525	E	USA	11	O157:H7	Plasmid	Unknown	ehxCABD	20
GCA_004176985	E	United Kingdom	11	O157:H7	Plasmid	Human	ehxCABD	20
GCA_003902425	E	USA	11	?:H7	Plasmid	Unknown	ehxCABD	20
GCA_002530945	B1	Canada	16	O111:H8	Not determined	Unknown	ehxCABD	27
GCA_004165515	E	United Kingdom	11	O157:H7	Plasmid	Human	ehxCABD	20
GCA_003361135	E	Canada	11	O157:H7	Plasmid	Unknown	ehxCABD	20
GCA_003899675	B1	USA	17	O45:H2	Not determined	Unknown	ehxCABD	15
GCA_003756445	B1	USA	655	?:H19	Not determined	Unknown	ehxCABD	18
GCA_003361885	E	Canada	11	O157:H7	Plasmid	Human	ehxCABD	20
GCA_004230485	B1	USA	21	O26:H11	Plasmid	Unknown	ehxCABD	27
GCA_003921655	E	USA	11	?:H7	Not determined	Unknown	ehxCABD	20
GCA_002770415	B1	Japan	21	O26:H11	Plasmid	Human	ehxCABD	27
GCA_000462505	E	Unknown	11	O157:H7	Plasmid	Unknown	ehxCABD	20
GCA_003742965	E	USA	11	?:H7	Plasmid	Unknown	ehxCABD	20
GCA_005394045	B1	Japan	795	?:H7	Plasmid	Cattle	ehxCABD	25
GCA_003360195	E	Canada	11	O157:H7	Plasmid	Human	ehxCABD	20
GCA_000622535	B1	USA	21	O26:H11	Plasmid	Unknown	ehxCABD	27
GCA_003923635	B1	USA	21	O26:H11	Plasmid	Unknown	ehxCABD	27
GCA_002475745	E	USA	11	O157:H7	Plasmid	Unknown	ehxCABD	20
GCA_005042785	B1	Canada	16	O111:H8	Not determined	Unknown	ehxCABD	27
GCA_004234285	B1	USA	16	O111:H8	Not determined	Unknown	ehxCABD	27
GCA_003770525	B1	USA	21	?:H11	Plasmid	Unknown	ehxCABD	27
GCA_003754245	E	USA	11	?:H7	Plasmid	Unknown	ehxCABD	20
GCA_001309815	D	Canada	32	O145:H28	Plasmid	Human	ehxCABD	8
GCA_001606865	B1	Netherlands	6042	O84:H2	Plasmid	Human	ehxCABD	14
GCA_004273625	E	United Kingdom	11	?:H7	Plasmid	Human	ehxCABD	20
GCA_005392245	A	Japan	206	O49:H10	Not determined	Cattle	ehxCABD	23
GCA_002175935	E	USA	11	O157:H7	Plasmid	Cattle	ehxCABD	20
GCA_003755885	E	USA	11	?:H7	Plasmid	Unknown	ehxCABD	20
GCA_003893615	E	United Kingdom	11	O157:H7	Plasmid	Human	ehxCABD	20
GCA_003027605	B1	USA	13	O75:H8	Plasmid	Wild animals	ehxCABD	25
GCA_001607555	B1	Netherlands	201	O112:H19	Plasmid	Human	ehxCABD	21
GCA_003360415	E	Canada	11	O157:H7	Plasmid	Human	ehxCABD	20
GCA_001607925	B1	Netherlands	33	O91:H14	Plasmid	Human	ehxCABD	12
GCA_005041595	B1	Canada	343	O103:H25	Not determined	Unknown	ehxCABD	14
GCA_002133595	A	Canada	10	O113:H4	Plasmid	Unknown	ehxCABD	24
GCA_002459635	B1	USA	154	O88:H25	Plasmid	Human	ehxCABD	25
GCA_004176675	E	United Kingdom	11	O157:H7	Plasmid	Human	ehxCABD	20
GCA_000616075	E	USA	11	O157:H7	Plasmid	Unknown	ehxCABD	20
GCA_002134385	E	Canada	11	O157:H7	Plasmid	Unknown	ehxCABD	20
GCA_003738445	E	USA	11	?:H7	Plasmid	Unknown	ehxCABD	20
GCA_004228795	E	USA	11	?:H7	Not determined	Unknown	ehxCABD	20
GCA_004265605	E	United Kingdom	11	O157:H7	Plasmid	Human	ehxCABD	20
GCA_001607735	E	Netherlands	11	O157:H7	Plasmid	Human	ehxCABD	20
GCA_003760405	B1	USA	723	O103:H11	Plasmid	Unknown	ehxCABD	27
GCA_005397165	B1	Japan	223	O113:H21	Plasmid	Cattle	ehxCABD	22
GCA_005400905	B1	France	327	O177:H11	Plasmid	Cattle	ehxCABD	27
GCA_004215755	E	United Kingdom	11	O157:H7	Plasmid	Human	ehxCABD	20
GCA_002515175	B1	USA	655	O121:H19	Plasmid	Human	ehxCABD	18
GCA_002767275	B1	Japan	21	O26:H11	Plasmid	Human	ehxCABD	27
GCA_002462275	B1	Canada	223	O113:H21	Plasmid	Cattle	ehxCABD	22
GCA_001191525	D	USA	32	O145:H28	Plasmid	Unknown	ehxCABD	8
GCA_000615455	B1	USA	655	O121:H19	Not determined	Unknown	ehxCABD	18
GCA_005040215	B1	Canada	33	O91:H14	Plasmid	Unknown	ehxCABD	12
GCA_003360715	E	Canada	11	O157:H7	Plasmid	Human	ehxCABD	20
GCA_002770355	B1	Japan	21	O26:H11	Plasmid	Human	ehxCABD	27
GCA_003361195	E	Canada	11	O157:H7	Plasmid	Unknown	ehxCABD	20
GCA_004230385	B1	USA	21	O69:H11	Plasmid	Unknown	ehxCABD	27
GCA_001445675	A	Belgium	342	?:NA	Plasmid	Cattle	ehxCABD	26
GCA_003892255	E	United Kingdom	11	O157:H7	Plasmid	Human	ehxCABD	20
GCA_003898255	B1	USA	21	?:H11	Plasmid	Unknown	ehxCABD	27
GCA_003908275	E	United Kingdom	11	O157:H7	Plasmid	Human	ehxCABD	20
GCA_000181775	E	Unknown	11	O157:H7	Plasmid	Unknown	ehxCABD	20
GCA_000614085	E	USA	11	O157:H7	Plasmid	Unknown	ehxCABD	20
GCA_003362095	E	Canada	11	O157:H7	Plasmid	Cattle	ehxCABD	20
GCA_004234325	B1	USA	21	O26:H11	Plasmid	Unknown	ehxCABD	27
GCA_002379215	B1	USA	223	O113:H21	Plasmid	Cattle	ehxCABD	22
GCA_008634225	D	Japan	32	O145:H28	Plasmid	Human	ehxCABD	8
GCA_003784565	E	USA	11	O157:H7	Plasmid	Unknown	ehxCABD	20
GCA_005384105	A	Japan	206	O49:H10	Not determined	Cattle	ehxCABD	23
GCA_000462725	E	Unknown	11	O157:H7	Plasmid	Unknown	ehxCABD	20
GCA_002164335	E	USA	11	O157:H7	Plasmid	Cattle	ehxCABD	20
GCA_003741125	E	USA	11	O157:H7	Plasmid	Unknown	ehxCABD	20

GCA_003361235	E	Canada	11	O157:H7	Plasmid	Cattle	ehxCABD	20
GCA_007647955	B1	Brazil	300	O182:H25	Not determined	Human	ehxCABD	14
GCA_003923135	B1	United Kingdom	21	O26:H11	Plasmid	Human	ehxCABD	27
GCA_005040415	B1	Canada	343	O103:H25	Not determined	Unknown	ehxCABD	14
GCA_003123015	A	France	301	O80:H2	Plasmid	Human	ehxCABD	19
GCA_008122375	E	United Kingdom	11	O157:H7	Plasmid	Human	ehxCABD	20
GCA_002923735	B1	Japan	21	O26:H11	Plasmid	Human	ehxCABD	27
GCA_004257945	E	United Kingdom	11	O157:H7	Plasmid	Human	ehxCABD	20
GCA_003113855	B1	Japan	21	O26:H11	Plasmid	Human	ehxCABD	27
GCA_002770335	B1	Japan	21	O26:H11	Plasmid	Human	ehxCABD	27
GCA_005038465	B1	Canada	21	O111:H11	Plasmid	Cattle	ehxCABD	27
GCA_005040755	B1	Canada	-	O103:H2	Not determined	Unknown	ehxCABD	15
GCA_000614595	B1	USA	655	O121:H19	Plasmid	Unknown	ehxCABD	18
GCA_004234065	B1	USA	21	?:H16	Plasmid	Unknown	ehxCABD	27
GCA_002767015	B1	Japan	21	O26:H11	Plasmid	Human	ehxCABD	27
GCA_005038015	A	Canada	329	O136:H16	Plasmid	Unknown	ehxCABD	23
GCA_003360815	E	Canada	11	O157:H7	Plasmid	Unknown	ehxCABD	20
GCA_003361295	E	Canada	11	O157:H7	Plasmid	Cattle	ehxCABD	20
GCA_004231205	B1	USA	21	?:H11	Not determined	Unknown	ehxCABD	27
GCA_002473855	E	USA	11	O157:H7	Plasmid	Unknown	ehxCABD	20
GCA_003754845	A	USA	342	O5:NA	Plasmid	Unknown	ehxCABD	26
GCA_002164105	E	USA	11	O157:H7	Plasmid	Cattle	ehxCABD	20
GCA_004181785	E	United Kingdom	11	?:H7	Plasmid	Human	ehxCABD	20
GCA_003741385	E	USA	11	?:H7	Plasmid	Unknown	ehxCABD	20
GCA_000462905	E	Unknown	11	O157:H7	Plasmid	Unknown	ehxCABD	20
GCA_004276095	B1	United Kingdom	21	O26:H11	Plasmid	Human	ehxCABD	27
GCA_005380805	B1	Japan	655	O121:H19	Plasmid	Human	ehxCABD	18
GCA_001677865	E	Argentina	11	O157:H7	Plasmid	Human	ehxCABD	20
GCA_005040525	B1	Canada	17	O103:H2	Plasmid	Unknown	ehxCABD	15
GCA_003879075	E	United Kingdom	11	O157:H7	Plasmid	Human	ehxCABD	20
GCA_003770605	E	USA	11	O157:H7	Plasmid	Unknown	ehxCABD	20
GCA_004158615	E	United Kingdom	11	O157:H7	Plasmid	Human	ehxCABD	20
GCA_002514585	B1	USA	135	?:H2	Plasmid	Cattle	ehxCABD	15
GCA_002834415	B1	USA	29	O26:H11	Plasmid	Cattle	ehxCABD	27
GCA_000316565	E	Unknown	11	O157:H7	Plasmid	Unknown	ehxCABD	20
GCA_004258785	B1	United Kingdom	350	O146:H21	Plasmid	Human	ehxCABD	17
GCA_002923535	B1	Japan	21	O26:H11	Plasmid	Human	ehxCABD	27
GCA_002176355	E	USA	11	O157:H7	Not determined	Cattle	ehxCABD	20
GCA_002458025	B1	Canada	350	O91:H21	Plasmid	Human	ehxCABD	17
GCA_002734545	B1	Japan	21	O26:H11	Plasmid	Cattle	ehxCABD	27
GCA_003027335	E	Austria	11	O157:H7	Plasmid	Human	ehxCABD	20
GCA_002766315	B1	Japan	21	O26:H11	Plasmid	Human	ehxCABD	27
GCA_005040725	A	Canada	119	O165:H25	Plasmid	Unknown	ehxCABD	26
GCA_003889795	D	United Kingdom	11	O157:H7	Plasmid	Human	ehxCABD	20
GCA_003360255	E	Canada	11	O157:H7	Plasmid	Human	ehxCABD	20
GCA_004157195	E	United Kingdom	11	O157:H7	Plasmid	Human	ehxCABD	20
GCA_005037905	B1	Canada	223	O113:H21	Plasmid	Unknown	ehxCABD	22
GCA_001606675	B1	Netherlands	33	O91:H14	Plasmid	Human	ehxCABD	12
GCA_003760945	B1	USA	21	?:H11	Plasmid	Unknown	ehxCABD	27
GCA_004230605	E	USA	11	?:H7	Plasmid	Unknown	ehxCABD	20
GCA_000647455	B1	Germany	661	O174:H2	Plasmid	Human	ehxCABD	25
GCA_002764695	B1	Japan	21	O26:H11	Plasmid	Human	ehxCABD	27
GCA_004157135	E	United Kingdom	11	?:H7	Plasmid	Human	ehxCABD	20
GCA_005038405	A	Canada	342	O5:NA	Plasmid	Cattle	ehxCABD	26
GCA_003123355	A	France	301	O80:H2	Plasmid	Human	ehxCABD	19
GCA_003916585	E	USA	11	?:H7	Plasmid	Unknown	ehxCABD	20
GCA_003359915	E	Canada	11	O157:H7	Plasmid	Human	ehxCABD	20
GCA_005041675	E	Canada	11	O157:H7	Plasmid	Unknown	ehxCABD	20
GCA_000619065	B1	USA	442	O91:H21	Plasmid	Unknown	ehxCABD	17
GCA_002515585	A	USA	342	O5:NA	Plasmid	Unknown	ehxCABD	26
GCA_004174815	E	United Kingdom	11	O157:H7	Plasmid	Human	ehxCABD	20
GCA_000462745	E	Unknown	11	O157:H7	Plasmid	Unknown	ehxCABD	20
GCA_004257985	E	United Kingdom	11	O157:H7	Plasmid	Human	ehxCABD	20
GCA_005391105	B1	Japan	297	?:H11	Plasmid	Cattle	ehxCABD	25
GCA_002549365	B1	USA	655	O121:H19	Plasmid	Sheep	ehxCABD	18
GCA_004164245	B1	United Kingdom	21	O26:H11	Plasmid	Human	ehxCABD	27
GCA_004182055	E	United Kingdom	11	O157:H7	Plasmid	Human	ehxCABD	20
GCA_004797065	D	USA	32	O145:H28	Plasmid	Cattle	ehxCABD	8
GCA_005044505	A	Canada	9055	O177:H25	Plasmid	Human	ehxCABD	26
GCA_003360315	E	Canada	11	O157:H7	Plasmid	Human	ehxCABD	20
GCA_001266095	D	Gambia	137	O145:H28	Plasmid	Human	ehxCABD	8
GCA_004232545	B1	USA	17	O151.Gp3/O118.Gp3:H2	Plasmid	Unknown	ehxCABD	15
GCA_002531445	E	Canada	11	O157:H7	Plasmid	Unknown	ehxCABD	20
GCA_004233945	E	USA	11	O157:H7	Plasmid	Unknown	ehxCABD	20
GCA_002767155	B1	Japan	21	O26:H11	Not determined	Human	ehxCABD	27
GCA_002458635	D	USA	32	?:H28	Plasmid	Human	ehxCABD	8
GCA_004796595	D	USA	32	O145:H28	Not determined	Human	ehxCABD	8
GCA_004156655	B1	United Kingdom	17	O103:H2	Not determined	Human	ehxCABD	15

GCA_005045475	D	Denmark	137	O145:H28	Not determined	Human	ehxCABD	8
GCA_002134585	E	Canada	11	O157:H7	Plasmid	Unknown	ehxCABD	20
GCA_008755835	B1	USA	480	O111:H8	Not determined	Human	ehxCABD	27
GCA_004174755	E	United Kingdom	11	O157:H7	Plasmid	Human	ehxCABD	20
GCA_002766155	B1	Japan	21	O26:H11	Plasmid	Human	ehxCABD	27
GCA_009648055	A	China	5044	O123.O186.Gp5/O123.Gp5:H12	Not determined	Dog	ehxCABD	23
GCA_002770515	B1	Japan	21	O26:H11	Plasmid	Cattle	ehxCABD	27
GCA_005045535	B1	Canada	154	O88:H25	Plasmid	Unknown	ehxCABD	25
GCA_001191505	B1	USA	655	O121:H19	Plasmid	Human	ehxCABD	18
GCA_003924875	E	USA	11	O157:H7	Plasmid	Unknown	ehxCABD	20
GCA_003764345	B1	USA	21	O151.Gp3/O118.Gp3:H16	Not determined	Unknown	ehxCABD	27
GCA_002206405	E	USA	11	O157:H7	Plasmid	Human	ehxCABD	20
GCA_002768815	B1	Japan	21	O26:H11	Plasmid	Human	ehxCABD	27
GCA_000462065	E	Unknown	11	O157:H7	Plasmid	Unknown	ehxCABD	20
GCA_004796885	D	USA	32	?:H28	Plasmid	Cattle	ehxCABD	8
GCA_004183075	E	United Kingdom	11	O157:H7	Plasmid	Human	ehxCABD	20
GCA_008753715	E	USA	11	O157:H7	Plasmid	Human	ehxCABD	20
GCA_002476355	B1	USA	131	O81:H21	Plasmid	Unknown	ehxCABD	25
GCA_002767035	B1	Japan	21	O26:H11	Plasmid	Human	ehxCABD	27
GCA_003362715	E	Canada	11	O157:H7	Plasmid	Human	ehxCABD	20
GCA_000618225	E	USA	11	O157:H7	Plasmid	Unknown	ehxCABD	20
GCA_003361635	E	Canada	11	O157:H7	Plasmid	Unknown	ehxCABD	20
GCA_003739485	E	USA	11	O157:H7	Plasmid	Unknown	ehxCABD	20
GCA_004231125	E	USA	11	?:H7	Plasmid	Unknown	ehxCABD	20
GCA_001617135	B1	China	29	O177:H11	Plasmid	Sheep	ehxCABD	27
GCA_001309895	E	Canada	11	O157:H7	Plasmid	Unknown	ehxCABD	20
GCA_001950695	D	Japan	32	O145:H28	Plasmid	Wild animals	ehxCABD	8
GCA_008634525	D	Japan	32	O145:H28	Plasmid	Human	ehxCABD	8
GCA_000617625	B1	USA	16	O111:H8	Not determined	Unknown	ehxCABD	27
GCA_004796905	D	USA	32	?:H28	Plasmid	Cattle	ehxCABD	8
GCA_003907375	B1	United Kingdom	300	?:H25	Not determined	Human	ehxCABD	14
GCA_003740085	B1	USA	17	O103:H2	Plasmid	Unknown	ehxCABD	15
GCA_002765955	B1	Japan	21	O26:H11	Plasmid	Human	ehxCABD	27
GCA_003907855	E	United Kingdom	11	O157:H7	Plasmid	Human	ehxCABD	20
GCA_003362295	E	Canada	11	O157:H7	Plasmid	Cattle	ehxCABD	20
GCA_005044135	B1	Canada	655	O121:H19	Not determined	Human	ehxCABD	18
GCA_003896275	B1	USA	1967	O103:H2	Plasmid	Unknown	ehxCABD	15
GCA_003763345	E	USA	11	O157:H7	Plasmid	Unknown	ehxCABD	20
GCA_002474035	E	USA	11	O157:H7	Plasmid	Unknown	ehxCABD	20
GCA_004163855	A	United Kingdom	10	O113:H4	Plasmid	Human	ehxCABD	24
GCA_001310005	E	Canada	11	O157:H7	Plasmid	Human	ehxCABD	20
GCA_002770075	B1	Japan	21	O26:H11	Plasmid	Human	ehxCABD	27
GCA_002027605	E	Canada	11	O157:H7	Plasmid	Cattle	ehxCABD	20
GCA_002195255	E	USA	11	O157:H7	Plasmid	Cattle	ehxCABD	20
GCA_008753435	E	USA	11	O157:H7	Plasmid	Human	ehxCABD	20
GCA_000460595	B1	Sweden	336	Onovel27:H16	Plasmid	Human	ehxCABD	25
GCA_008633545	D	Japan	32	O145:H28	Not determined	Human	ehxCABD	8
GCA_003362395	E	Canada	11	O157:H7	Plasmid	Human	ehxCABD	20
GCA_000316465	E	Unknown	11	O157:H7	Plasmid	Unknown	ehxCABD	20
GCA_001012075	D	USA	32	O145:H28	Plasmid	Unknown	ehxCABD	8
GCA_000616645	E	USA	11	O157:H7	Plasmid	Unknown	ehxCABD	20
GCA_002164295	E	USA	11	O157:H7	Plasmid	Cattle	ehxCABD	20
GCA_004165695	B1	United Kingdom	442	O146:H21	Plasmid	Human	ehxCABD	17
GCA_003361695	E	Canada	11	O157:H7	Plasmid	Unknown	ehxCABD	20
GCA_000462545	E	Unknown	11	O157:H7	Plasmid	Unknown	ehxCABD	20
GCA_002164545	E	USA	11	O157:H7	Plasmid	Cattle	ehxCABD	20
GCA_003360395	E	Canada	11	O157:H7	Plasmid	Human	ehxCABD	20
GCA_001281845	E	Netherlands	11	O157:H7	Plasmid	Human	ehxCABD	20
GCA_000622675	B1	USA	21	O26:H11	Plasmid	Unknown	ehxCABD	27
GCA_005381225	B1	Japan	655	O121:H19	Plasmid	Human	ehxCABD	18
GCA_005043035	B1	Canada	21	O151.Gp3/O118.Gp3:H16	Plasmid	Unknown	ehxCABD	27
GCA_004273505	D	United Kingdom	32	O145:H28	Plasmid	Human	ehxCABD	8
GCA_003903535	E	United Kingdom	11	O157:H7	Plasmid	Human	ehxCABD	20
GCA_003359445	E	Canada	11	O157:H7	Plasmid	Unknown	ehxCABD	20
GCA_002460175	A	USA	342	O5:NA	Plasmid	Human	ehxCABD	26
GCA_000462865	E	Unknown	11	O157:H7	Plasmid	Unknown	ehxCABD	20
GCA_004766705	B1	Australia	21	O26:H11	Plasmid	Human	ehxCABD	27
GCA_005041565	B1	Canada	16	O111:H8	Not determined	Unknown	ehxCABD	27
GCA_002531015	B1	Canada	21	O111:H11	Plasmid	Unknown	ehxCABD	27
GCA_003123495	A	France	301	O80:H2	Plasmid	Human	ehxCABD	19
GCA_002198005	E	USA	11	O157:H7	Plasmid	Human	ehxCABD	20
GCA_000462585	E	Unknown	11	O157:H7	Plasmid	Unknown	ehxCABD	20
GCA_003918765	E	USA	11	O157:H7	Plasmid	Unknown	ehxCABD	20
GCA_003754985	B1	USA	135	O103:H2	Plasmid	Unknown	ehxCABD	15
GCA_000447025	E	Japan	11	O157:H7	Plasmid	Cattle	ehxCABD	20
GCA_008635605	D	Japan	32	?:H28	Plasmid	Human	ehxCABD	8
GCA_005044695	B1	Denmark	7657	O146:H21	Not determined	Unknown	ehxCABD	17
GCA_002473995	E	USA	11	O157:H7	Plasmid	Unknown	ehxCABD	20

GCA_002175875	E	USA	11	O157:H7	Plasmid	Cattle	ehxCABD	20
GCA_002531045	B1	Canada	16	O111:H8	Not determined	Unknown	ehxCABD	27
GCA_003764505	B1	USA	17	?:H2	Not determined	Unknown	ehxCABD	15
GCA_001677805	E	Argentina	11	O157:H7	Plasmid	Human	ehxCABD	20
GCA_003360755	E	Canada	11	O157:H7	Plasmid	Human	ehxCABD	20
GCA_005040245	E	Canada	11	?:H7	Plasmid	Unknown	ehxCABD	20
GCA_005384225	A	Japan	344	O109:H48	Not determined	Cattle	ehxCABD	23
GCA_002516285	D	USA	1176	O36:H14	Plasmid	Unknown	ehxCABD	29
GCA_002765115	B1	Japan	481	O26:H11	Plasmid	Human	ehxCABD	27
GCA_005046745	B1	Switzerland	21	O26:H11	Plasmid	Human	ehxCABD	27
GCA_001571485	B1	USA	21	O26:H11	Not determined	Cattle	ehxCABD	27
GCA_003122995	A	France	301	O80:H2	Plasmid	Human	ehxCABD	19
GCA_003362175	E	Canada	11	O157:H7	Plasmid	Cattle	ehxCABD	20
GCA_003123555	A	France	301	O80:H2	Plasmid	Human	ehxCABD	19
GCA_005381245	B1	Japan	655	O121:H19	Plasmid	Human	ehxCABD	18
GCA_002767235	B1	Japan	21	O26:H11	Plasmid	Human	ehxCABD	27
GCA_002810655	B1	USA	29	O26:H11	Plasmid	Cattle	ehxCABD	27
GCA_003885595	B1	United Kingdom	25	O128:H2	Plasmid	Human	ehxCABD	13
GCA_004163475	E	United Kingdom	11	O157:H7	Plasmid	Human	ehxCABD	20
GCA_003362645	E	Canada	11	O157:H7	Plasmid	Human	ehxCABD	20
GCA_005044825	B1	Canada	655	O121:H19	Plasmid	Human	ehxCABD	18
GCA_002764555	B1	Japan	21	O26:H11	Plasmid	Human	ehxCABD	27
GCA_003360935	E	Canada	11	O157:H7	Plasmid	Unknown	ehxCABD	20
GCA_003123215	A	Spain	301	O80:H2	Plasmid	Cattle	ehxCABD	19
GCA_005395805	B1	Japan	87	O28ac.O42.Gp2/O42.Gp2:H25	Plasmid	Cattle	ehxCABD	22
GCA_004272375	B1	United Kingdom	297	O3:H21	Plasmid	Human	ehxCABD	25
GCA_003891235	E	United Kingdom	11	O157:H7	Plasmid	Human	ehxCABD	20
GCA_001607875	B1	Netherlands	21	O26:H11	Plasmid	Human	ehxCABD	27
GCA_008634665	D	Japan	32	O145:H28	Plasmid	Human	ehxCABD	8
GCA_002164045	E	USA	11	O157:H7	Plasmid	Cattle	ehxCABD	20
GCA_003901575	B1	United Kingdom	8097	O174:H16	Plasmid	Human	ehxCABD	25
GCA_004259825	E	United Kingdom	11	O157:H7	Plasmid	Human	ehxCABD	20
GCA_002517145	A	USA	10	O2.Gp7/O2.O50.Gp7:H27	Not determined	Cattle	ehxCABD	24
GCA_004230585	B1	USA	21	?:H11	Plasmid	Unknown	ehxCABD	27
GCA_002175655	E	USA	11	O157:H7	Plasmid	Cattle	ehxCABD	20
GCA_004161315	E	United Kingdom	11	O157:H7	Plasmid	Human	ehxCABD	20
GCA_003742885	B1	USA	17	O103:H2	Not determined	Unknown	ehxCABD	15
GCA_003876425	B1	USA	21	O26:H11	Plasmid	Unknown	ehxCABD	27
GCA_003737535	E	USA	11	?:H7	Plasmid	Unknown	ehxCABD	20
GCA_005038475	B1	Canada	21	O26:H11	Plasmid	Cattle	ehxCABD	27
GCA_004175385	E	United Kingdom	11	O157:H7	Plasmid	Human	ehxCABD	20
GCA_002764475	B1	Japan	21	O26:H11	Plasmid	Cattle	ehxCABD	27
GCA_000619685	E	USA	11	O157:H7	Plasmid	Unknown	ehxCABD	20
GCA_002923965	B1	Japan	21	O26:H11	Plasmid	Human	ehxCABD	27
GCA_004231825	E	USA	11	?:H7	Plasmid	Unknown	ehxCABD	20
GCA_002765555	B1	Japan	21	O26:H11	Plasmid	Human	ehxCABD	27
GCA_003882575	E	United Kingdom	11	O157:H7	Plasmid	Human	ehxCABD	20
GCA_005043555	E	Canada	724	?:H20	Plasmid	Cattle	ehxCABD	11
GCA_002164115	E	USA	11	O157:H7	Plasmid	Cattle	ehxCABD	20
GCA_000617265	B1	USA	16	O111:H8	Not determined	Unknown	ehxCABD	27
GCA_008635765	D	Japan	32	?:H28	Plasmid	Human	ehxCABD	8
GCA_005040625	B1	Canada	16	O111:H8	Plasmid	Unknown	ehxCABD	27
GCA_000462465	E	Unknown	11	O157:H7	Plasmid	Unknown	ehxCABD	20
GCA_005040325	E	Canada	11	O157:H7	Plasmid	Unknown	ehxCABD	20
GCA_000797715	A	USA	329	O136:H16	Plasmid	Unknown	ehxCABD	23
GCA_002164465	E	USA	11	?:H7	Plasmid	Cattle	ehxCABD	20
GCA_004276275	E	United Kingdom	11	O157:H7	Plasmid	Human	ehxCABD	20
GCA_003360565	E	Canada	11	O157:H7	Plasmid	Human	ehxCABD	20
GCA_005394085	B1	Japan	655	O121:H19	Plasmid	Cattle	ehxCABD	18
GCA_003361515	E	Canada	11	O157:H7	Plasmid	Unknown	ehxCABD	20
GCA_000461955	E	Unknown	11	O157:H7	Plasmid	Unknown	ehxCABD	20
GCA_003879515	B1	United Kingdom	25	?:H2	Plasmid	Human	ehxCABD	13
GCA_001616985	B1	China	29	O177:H11	Plasmid	Sheep	ehxCABD	27
GCA_005394505	B1	Japan	297	?:H11	Plasmid	Cattle	ehxCABD	25
GCA_000619045	E	USA	11	O157:H7	Plasmid	Unknown	ehxCABD	20
GCA_004268265	E	United Kingdom	11	O157:H7	Plasmid	Human	ehxCABD	20
GCA_004766575	E	USA	11	O157:H7	Plasmid	Pig	ehxCABD	20
GCA_002835125	B1	USA	29	O26:H11	Plasmid	Cattle	ehxCABD	27
GCA_000615385	D	USA	32	O145:H28	Plasmid	Unknown	ehxCABD	8
GCA_002286545	E	Israel	11	?:H7	Plasmid	Human	ehxCABD	20
GCA_002286455	B1	Israel	481	O26:H11	Plasmid	Human	ehxCABD	27
GCA_000619225	B1	USA	16	O111:H8	Not determined	Unknown	ehxCABD	27
GCA_008635965	D	Japan	32	?:H28	Plasmid	Human	ehxCABD	8
GCA_001012505	A	USA	344	O109:H48	Plasmid	Unknown	ehxCABD	23
GCA_005392385	A	Japan	206	O35:H10	Not determined	Cattle	ehxCABD	23
GCA_002923915	B1	Japan	21	O26:H11	Plasmid	Human	ehxCABD	27
GCA_000615815	E	USA	11	O157:H7	Plasmid	Unknown	ehxCABD	20
GCA_002765295	B1	Japan	21	O26:H11	Not determined	Human	ehxCABD	27

GCA_005038095	B1	Canada	300	O182:H25	Plasmid	Unknown	ehxCABD	14
GCA_005041585	B1	Canada	17	O103:H2	Plasmid	Unknown	ehxCABD	15
GCA_001281905	E	Netherlands	11	O157:H7	Plasmid	Human	ehxCABD	20
GCA_004233345	B1	USA	655	O121:H19	Not determined	Unknown	ehxCABD	18
GCA_002734885	B1	USA	21	O26:H11	Plasmid	Cattle	ehxCABD	27
GCA_004164095	B1	United Kingdom	21	?:H11	Plasmid	Human	ehxCABD	27
GCA_002769275	B1	Japan	21	O26:H11	Plasmid	Cattle	ehxCABD	27
GCA_002164035	E	USA	11	O157:H7	Plasmid	Cattle	ehxCABD	20
GCA_001571925	B1	USA	29	O26:H11	Plasmid	Cattle	ehxCABD	27
GCA_003360895	E	Canada	11	O157:H7	Plasmid	Unknown	ehxCABD	20
GCA_002764395	B1	Japan	21	O26:H11	Plasmid	Cattle	ehxCABD	27
GCA_002764975	B1	Japan	21	O26:H11	Plasmid	Human	ehxCABD	27
GCA_002764915	B1	Japan	21	O26:H11	Plasmid	Human	ehxCABD	27
GCA_002195115	E	USA	11	O157:H7	Plasmid	Cattle	ehxCABD	20
GCA_003757005	B1	USA	17	?:H2	Plasmid	Unknown	ehxCABD	15
GCA_000616405	E	USA	11	O157:H7	Plasmid	Unknown	ehxCABD	20
GCA_004265965	E	United Kingdom	11	O157:H7	Plasmid	Human	ehxCABD	20
GCA_002457935	B1	USA	17	O45:H2	Not determined	Cattle	ehxCABD	15
GCA_003753865	B1	USA	17	O103:H2	Not determined	Unknown	ehxCABD	15
GCA_002766855	B1	Japan	21	O26:H11	Plasmid	Human	ehxCABD	27
GCA_003891495	B1	United Kingdom	655	O121:H19	Plasmid	Human	ehxCABD	18
GCA_002768445	B1	Japan	21	O26:H11	Plasmid	Human	ehxCABD	27
GCA_005043055	B1	Canada	2773	?:H7	Plasmid	Unknown	ehxCABD	25
GCA_000462345	E	Unknown	11	O157:H7	Plasmid	Unknown	ehxCABD	20
GCA_001607845	B1	Netherlands	300	O182:H25	Not determined	Human	ehxCABD	14
GCA_002195825	E	USA	11	O157:H7	Plasmid	Cattle	ehxCABD	20
GCA_003912155	B1	USA	17	?:H2	Not determined	Unknown	ehxCABD	15
GCA_002475875	E	USA	11	O157:H7	Plasmid	Unknown	ehxCABD	20
GCA_004163815	E	United Kingdom	11	O157:H7	Plasmid	Human	ehxCABD	20
GCA_002767405	B1	Japan	21	O26:H11	Plasmid	Human	ehxCABD	27
GCA_008633725	D	Belgium	32	O145:H28	Plasmid	Human	ehxCABD	8
GCA_003899475	B1	USA	21	?:H11	Plasmid	Unknown	ehxCABD	27
GCA_005045015	B1	Canada	21	O26:H11	Not determined	Human	ehxCABD	27
GCA_004184375	E	United Kingdom	11	?:H7	Plasmid	Human	ehxCABD	20
GCA_003361255	E	Canada	11	O157:H7	Plasmid	Cattle	ehxCABD	20
GCA_000615575	B1	USA	16	O111:H8	Not determined	Unknown	ehxCABD	27
GCA_001309805	B1	Canada	655	O121:H19	Plasmid	Human	ehxCABD	18
GCA_001730745	E	USA	11	?:H7	Plasmid	Human	ehxCABD	20
GCA_003360615	E	Canada	11	O157:H7	Plasmid	Human	ehxCABD	20
GCA_003760205	E	USA	11	?:H7	Plasmid	Unknown	ehxCABD	20
GCA_000618065	E	USA	11	O157:H7	Plasmid	Unknown	ehxCABD	20
GCA_005042755	B1	Switzerland	655	O121:H19	Plasmid	Human	ehxCABD	18
GCA_002769875	B1	Japan	21	O26:H11	Plasmid	Human	ehxCABD	27
GCA_003758665	E	USA	11	O157:H7	Plasmid	Unknown	ehxCABD	20
GCA_005395565	B1	Japan	-	?:H7	Plasmid	Cattle	ehxCABD	25
GCA_003361335	E	Canada	11	O157:H7	Plasmid	Unknown	ehxCABD	20
GCA_005390185	B1	Japan	7895	?:H2	Not determined	Cattle	ehxCABD	25
GCA_000618045	E	USA	11	O157:H7	Plasmid	Unknown	ehxCABD	20
GCA_002765775	B1	Japan	21	O26:H11	Plasmid	Human	ehxCABD	27
GCA_005392825	A	Japan	6126	O10:H25	Not determined	Cattle	ehxCABD	26
GCA_003922815	B1	USA	17	O103:H2	Not determined	Unknown	ehxCABD	15
GCA_001281795	E	Netherlands	11	O157:H7	Plasmid	Human	ehxCABD	20
GCA_002476085	E	USA	628	O157:H7	Plasmid	Unknown	ehxCABD	20
GCA_001616695	B1	China	29	O177:H11	Plasmid	Sheep	ehxCABD	27
GCA_002515915	B1	USA	4496	?:H28	Not determined	Unknown	ehxCABD	21
GCA_005384165	B1	Japan	205	?:H19	Plasmid	Cattle	ehxCABD	25
GCA_002164185	E	USA	11	O157:H7	Plasmid	Cattle	ehxCABD	20
GCA_002770035	B1	Japan	21	O26:H11	Plasmid	Human	ehxCABD	27
GCA_005041805	B1	Canada	679	O163:H19	Plasmid	Unknown	ehxCABD	10
GCA_001997405	B1	Unknown	655	O121:H19	Plasmid	Unknown	ehxCABD	18
GCA_003900715	B1	USA	655	O121:H19	Plasmid	Unknown	ehxCABD	18
GCA_002734725	B1	USA	21	O26:H11	Not determined	Cattle	ehxCABD	27
GCA_003347215	E	Romania	628	O157:H7	Plasmid	Human	ehxCABD	20
GCA_005045325	B1	Canada	21	O26:H11	Plasmid	Human	ehxCABD	27
GCA_002474105	E	USA	11	O157:H7	Plasmid	Unknown	ehxCABD	20
GCA_002770135	B1	Japan	21	O26:H11	Plasmid	Human	ehxCABD	27
GCA_000617125	B1	USA	17	O45:H2	Not determined	Unknown	ehxCABD	15
GCA_003768565	B1	USA	17	O45:H2	Not determined	Unknown	ehxCABD	15
GCA_001606955	B1	Netherlands	350	O146:H21	Plasmid	Human	ehxCABD	17
GCA_001608085	B1	Netherlands	21	O69:H11	Plasmid	Human	ehxCABD	27
GCA_004256205	E	United Kingdom	11	O157:H7	Plasmid	Unknown	ehxCABD	20
GCA_002924065	B1	Belgium	21	O26:H11	Plasmid	Human	ehxCABD	27
GCA_003360785	E	Canada	11	O157:H7	Plasmid	Human	ehxCABD	20
GCA_003887435	B1	United Kingdom	675	?:H19	Not determined	Human	ehxCABD	25
GCA_002810705	B1	USA	29	O26:H11	Plasmid	Cattle	ehxCABD	27
GCA_003887395	E	United Kingdom	11	O157:H7	Plasmid	Human	ehxCABD	20
GCA_008635835	D	Japan	32	?:H28	Plasmid	Human	ehxCABD	8
GCA_003362475	E	Canada	11	O157:H7	Plasmid	Human	ehxCABD	20

GCA_005042035	B1	Canada	2385	?:H19	Plasmid	Unknown	ehxCABD	21
GCA_002476165	E	USA	11	O157:H7	Plasmid	Unknown	ehxCABD	20
GCA_002765155	B1	Japan	21	O26:H11	Plasmid	Human	ehxCABD	27
GCA_005394985	B1	Japan	5978	O108var1:H8	Plasmid	Cattle	ehxCABD	25
GCA_008755765	B1	USA	480	O111:H8	Not determined	Human	ehxCABD	27
GCA_003897355	E	United Kingdom	11	O157:H7	Plasmid	Human	ehxCABD	20
GCA_008122355	E	United Kingdom	11	O157:H7	Plasmid	Human	ehxCABD	20
GCA_002473915	E	USA	11	O157:H7	Plasmid	Unknown	ehxCABD	20
GCA_005042075	A	Canada	342	O5:NA	Plasmid	Unknown	ehxCABD	26
GCA_003757575	E	USA	5516	?:H7	Plasmid	Unknown	ehxCABD	20
GCA_005044175	B1	Canada	16	O111:H8	Not determined	Human	ehxCABD	27
GCA_002924205	B1	Japan	21	O26:H11	Plasmid	Human	ehxCABD	27
GCA_003764045	B1	USA	21	O26:H11	Plasmid	Unknown	ehxCABD	27
GCA_002133535	B1	Canada	7657	O146:H21	Not determined	Unknown	ehxCABD	17
GCA_001309965	E	Canada	11	O157:H7	Plasmid	Cattle	ehxCABD	20
GCA_002764795	B1	Japan	21	O26:H11	Plasmid	Human	ehxCABD	27
GCA_005384025	B1	Japan	5937	O8:H8	Plasmid	Cattle	ehxCABD	25
GCA_003902475	C	United Kingdom	3101	O78:H4	Not determined	Human	ehxCABD	16
GCA_000462145	E	Unknown	11	O157:H7	Plasmid	Unknown	ehxCABD	20
GCA_002475725	E	USA	11	O157:H7	Plasmid	Unknown	ehxCABD	20
GCA_008753635	E	USA	11	O157:H7	Plasmid	Human	ehxCABD	20
GCA_004160955	E	United Kingdom	11	O157:H7	Plasmid	Human	ehxCABD	20
GCA_005043335	B1	Canada	480	O111:H8	Not determined	Unknown	ehxCABD	27
GCA_002765235	B1	Japan	21	O26:H11	Plasmid	Human	ehxCABD	27
GCA_004767135	B1	Unknown	33	O91:H14	Not determined	Unknown	ehxCABD	12
GCA_005381305	B1	Japan	655	O121:H19	Plasmid	Human	ehxCABD	18
GCA_003027755	B1	USA	300	O182:H25	Not determined	Wild animals	ehxCABD	14
GCA_002195075	E	USA	11	O157:H7	Plasmid	Cattle	ehxCABD	20
GCA_003361095	E	Canada	11	O157:H7	Plasmid	Unknown	ehxCABD	20
GCA_005394465	B1	Japan	795	?:H7	Not determined	Cattle	ehxCABD	25
GCA_002770195	B1	Japan	21	O26:H11	Plasmid	Human	ehxCABD	27
GCA_003917095	B1	USA	17	?:H2	Plasmid	Unknown	ehxCABD	15
GCA_002834425	B1	USA	29	O26:H11	Plasmid	Cattle	ehxCABD	27
GCA_003740485	E	USA	11	?:H7	Plasmid	Unknown	ehxCABD	20
GCA_002476195	E	USA	11	O157:H7	Plasmid	Unknown	ehxCABD	20
GCA_008634305	D	Japan	32	O145:H28	Plasmid	Human	ehxCABD	8
GCA_008755775	B1	USA	16	O111:H8	Not determined	Human	ehxCABD	27
GCA_008757055	B1	USA	17	O103:H2	Not determined	Human	ehxCABD	15
GCA_004234365	E	USA	11	O157:H7	Plasmid	Unknown	ehxCABD	20
GCA_000267045	E	USA	11	O157:H7	Plasmid	Human	ehxCABD	20
GCA_002458785	B1	USA	16	O111:H8	Not determined	Human	ehxCABD	27
GCA_003360535	E	Canada	11	O157:H7	Plasmid	Human	ehxCABD	20
GCA_001012495	A	USA	344	O109:H48	Plasmid	Unknown	ehxCABD	23
GCA_005038445	B1	Canada	21	O111:H11	Not determined	Cattle	ehxCABD	27
GCA_003882795	B1	United Kingdom	442	?:H21	Plasmid	Human	ehxCABD	17
GCA_000695135	B1	France	17	O103:H2	Not determined	Unknown	ehxCABD	15
GCA_002767215	B1	Japan	21	O26:H11	Plasmid	Human	ehxCABD	27
GCA_005046415	B1	Canada	350	O91:H21	Plasmid	Cattle	ehxCABD	17
GCA_002769075	B1	Belgium	21	O26:H11	Plasmid	Human	ehxCABD	27
GCA_002765495	B1	Japan	21	O26:H11	Plasmid	Human	ehxCABD	27
GCA_002764375	B1	Japan	21	O26:H11	Plasmid	Cattle	ehxCABD	27
GCA_005390225	E	Japan	691	?:H20	Plasmid	Cattle	ehxCABD	11
GCA_002766095	B1	Japan	21	O26:H11	Plasmid	Human	ehxCABD	27
GCA_003359575	E	Canada	11	O157:H7	Plasmid	Unknown	ehxCABD	20
GCA_004188555	E	United Kingdom	11	O157:H7	Plasmid	Human	ehxCABD	20
GCA_001609765	B1	Netherlands	21	O26:H11	Plasmid	Human	ehxCABD	27
GCA_003759285	B1	USA	21	O71:H11	Plasmid	Unknown	ehxCABD	27
GCA_003739565	E	USA	11	?:H7	Plasmid	Unknown	ehxCABD	20
GCA_003887495	B1	United Kingdom	300	O156:H25	Not determined	Human	ehxCABD	14
GCA_002473875	E	USA	11	O157:H7	Plasmid	Unknown	ehxCABD	20
GCA_002531205	B1	Canada	21	O111:H11	Plasmid	Unknown	ehxCABD	27
GCA_003920375	B1	USA	21	?:H11	Plasmid	Unknown	ehxCABD	27
GCA_002765215	B1	Japan	21	O26:H11	Plasmid	Human	ehxCABD	27
GCA_003914035	E	United Kingdom	11	O157:H7	Plasmid	Human	ehxCABD	20
GCA_005040045	A	Canada	342	O5:NA	Plasmid	Unknown	ehxCABD	26
GCA_000616035	E	USA	11	O157:H7	Plasmid	Unknown	ehxCABD	20
GCA_005041605	B1	Canada	679	O163:H19	Plasmid	Cattle	ehxCABD	10
GCA_000335075	E	Unknown	11	O157:H7	Plasmid	Unknown	ehxCABD	20
GCA_003362215	E	Canada	11	O157:H7	Plasmid	Cattle	ehxCABD	20
GCA_004230525	E	USA	628	?:H7	Plasmid	Unknown	ehxCABD	20
GCA_002923715	B1	Japan	21	O26:H11	Plasmid	Human	ehxCABD	27
GCA_004161475	E	United Kingdom	11	O157:H7	Plasmid	Human	ehxCABD	20
GCA_001281885	E	Netherlands	11	O157:H7	Plasmid	Human	ehxCABD	20
GCA_008756055	B1	USA	16	?:H8	Not determined	Human	ehxCABD	27
GCA_003747865	B1	USA	16	O111:NA	Not determined	Unknown	ehxCABD	27
GCA_003884915	E	United Kingdom	11	O157:H7	Plasmid	Human	ehxCABD	20
GCA_000617445	E	USA	11	O157:H7	Plasmid	Unknown	ehxCABD	20
GCA_003896215	B1	USA	21	?:H16	Plasmid	Unknown	ehxCABD	27

GCA_005041695	E	Canada	11	O157:H7	Plasmid	Unknown	ehxCABD	20
GCA_003362425	E	Canada	11	O157:H7	Plasmid	Human	ehxCABD	20
GCA_003767945	B1	USA	655	O121:H19	Plasmid	Unknown	ehxCABD	18
GCA_003360735	E	Canada	11	O157:H7	Plasmid	Human	ehxCABD	20
GCA_003919995	B1	USA	21	?:H11	Not determined	Unknown	ehxCABD	27
GCA_003916615	B1	USA	21	O26:H11	Plasmid	Unknown	ehxCABD	27
GCA_005038705	B1	Unknown	135	O103:H2	Plasmid	Human	ehxCABD	15
GCA_002770395	B1	Japan	21	O26:H11	Plasmid	Human	ehxCABD	27
GCA_003113555	B1	Japan	21	?:H11	Plasmid	Human	ehxCABD	27
GCA_004231255	B1	USA	17	O103:H2	Not determined	Unknown	ehxCABD	15
GCA_001660245	B1	Japan	223	O113:H21	Plasmid	Cattle	ehxCABD	22
GCA_003880615	B1	United Kingdom	16	O111:H8	Not determined	Human	ehxCABD	27
GCA_002766395	B1	Japan	21	O26:H11	Plasmid	Human	ehxCABD	27
GCA_003759605	E	USA	11	O157:H7	Not determined	Unknown	ehxCABD	20
GCA_003922595	B1	USA	17	?:H2	Plasmid	Unknown	ehxCABD	15
GCA_000335375	E	Unknown	11	O157:H7	Plasmid	Unknown	ehxCABD	20
GCA_003736505	B1	USA	16	?:H8	Not determined	Unknown	ehxCABD	27
GCA_005043725	B1	Canada	442	O91:H21	Plasmid	Unknown	ehxCABD	17
GCA_002765275	B1	Japan	21	O26:H11	Plasmid	Human	ehxCABD	27
GCA_003769865	B1	USA	16	?:H8	Not determined	Unknown	ehxCABD	27
GCA_000303755	E	Unknown	11	O157:H7	Plasmid	Unknown	ehxCABD	20
GCA_002765675	B1	Japan	21	O26:H11	Not determined	Human	ehxCABD	27
GCA_001606815	B1	Netherlands	21	O26:H11	Plasmid	Human	ehxCABD	27
GCA_002735085	B1	France	29	O26:H11	Plasmid	Human	ehxCABD	27
GCA_008635785	D	Japan	32	?:H28	Plasmid	Human	ehxCABD	8
GCA_003362255	E	Canada	11	O157:H7	Plasmid	Cattle	ehxCABD	20
GCA_001606375	B1	Netherlands	21	O69:H11	Plasmid	Human	ehxCABD	27
GCA_003880675	B1	United Kingdom	21	O26:H11	Not determined	Human	ehxCABD	27
GCA_004254885	E	United Kingdom	11	O157:H7	Plasmid	Human	ehxCABD	20
GCA_005395605	B1	Japan	-	Onovel30:H7	Plasmid	Cattle	ehxCABD	25
GCA_002769595	B1	Japan	21	O26:H11	Plasmid	Human	ehxCABD	27
GCA_005046595	B1	USA	17	O45:H2	Not determined	Human	ehxCABD	15
GCA_000467695	B1	Australia	294	O111:H8	Not determined	Unknown	ehxCABD	27
GCA_005043615	B1	Canada	21	O26:H11	Plasmid	Unknown	ehxCABD	27
GCA_004182095	E	United Kingdom	11	O157:H7	Plasmid	Human	ehxCABD	20
GCA_001660325	B1	Japan	21	O26:H11	Plasmid	Cattle	ehxCABD	27
GCA_005397405	B1	Japan	5973	?:H2	Plasmid	Cattle	ehxCABD	25
GCA_003362195	E	Canada	11	O157:H7	Plasmid	Cattle	ehxCABD	20
GCA_001012545	A	USA	329	O136:H16	Plasmid	Unknown	ehxCABD	23
GCA_003113835	B1	Japan	481	O26:H11	Plasmid	Human	ehxCABD	27
GCA_002765735	B1	Japan	21	O26:H11	Plasmid	Human	ehxCABD	27
GCA_900448275	E	Austria	11	O157:H7	Plasmid	Unknown	ehxCABD	20
GCA_000615695	E	USA	11	O157:H7	Plasmid	Unknown	ehxCABD	20
GCA_003762945	E	USA	628	O157:H7	Plasmid	Unknown	ehxCABD	20
GCA_004232905	B1	USA	17	O103:H2	Not determined	Unknown	ehxCABD	15
GCA_002476135	E	USA	11	O157:H7	Plasmid	Unknown	ehxCABD	20
GCA_004228425	E	USA	11	O157:H7	Plasmid	Unknown	ehxCABD	20
GCA_005044995	B1	Canada	481	O26:H11	Plasmid	Unknown	ehxCABD	27
GCA_002834445	B1	USA	29	O26:H11	Plasmid	Cattle	ehxCABD	27
GCA_002179755	E	USA	11	?:H7	Plasmid	Cattle	ehxCABD	20
GCA_005044785	B1	Canada	16	O111:H8	Not determined	Human	ehxCABD	27
GCA_004272705	E	United Kingdom	11	O157:H7	Plasmid	Human	ehxCABD	20
GCA_005041035	B1	Canada	679	O163:H19	Plasmid	Unknown	ehxCABD	10
GCA_005045505	B1	Denmark	21	O26:H11	Plasmid	Human	ehxCABD	27
GCA_002765095	B1	Japan	21	O26:H11	Plasmid	Human	ehxCABD	27
GCA_005392845	B1	Japan	517	O153var1:H19	Plasmid	Cattle	ehxCABD	21
GCA_003893835	B1	United Kingdom	442	?:H21	Plasmid	Human	ehxCABD	17
GCA_004232225	B1	USA	350	?:H21	Plasmid	Unknown	ehxCABD	17
GCA_005396985	D	Japan	32	?:H28	Plasmid	Cattle	ehxCABD	8
GCA_001885025	E	USA	11	?:H7	Plasmid	Cattle	ehxCABD	20
GCA_004264415	B1	United Kingdom	17	O103:H2	Not determined	Human	ehxCABD	15
GCA_002766495	B1	Japan	21	O26:H11	Plasmid	Human	ehxCABD	27
GCA_003360295	E	Canada	11	O157:H7	Plasmid	Human	ehxCABD	20
GCA_005040005	B1	Canada	295	?:H11	Plasmid	Unknown	ehxCABD	25
GCA_002767675	B1	Japan	21	O26:H11	Plasmid	Human	ehxCABD	27
GCA_000619085	B1	USA	655	O121:H19	Plasmid	Unknown	ehxCABD	18
GCA_003361215	E	Canada	11	O157:H7	Plasmid	Cattle	ehxCABD	20
GCA_004270275	E	United Kingdom	11	O157:H7	Plasmid	Human	ehxCABD	20
GCA_002769975	B1	Japan	21	O26:H11	Plasmid	Human	ehxCABD	27
GCA_000303695	E	Unknown	628	O157:H7	Plasmid	Unknown	ehxCABD	20
GCA_001012235	B1	USA	16	O111:H8	Not determined	Unknown	ehxCABD	27
GCA_008755385	E	USA	11	O157:H7	Plasmid	Human	ehxCABD	20
GCA_004252885	B1	United Kingdom	21	O26:H11	Plasmid	Human	ehxCABD	27
GCA_004254515	B1	United Kingdom	21	O26:H11	Plasmid	Human	ehxCABD	27
GCA_005383985	B1	Japan	679	?:H19	Plasmid	Cattle	ehxCABD	10
GCA_005043175	D	Canada	32	?:H28	Plasmid	Unknown	ehxCABD	8
GCA_003905505	B1	USA	17	O45:H2	Plasmid	Unknown	ehxCABD	15
GCA_002164025	E	USA	11	O157:H7	Plasmid	Cattle	ehxCABD	20

GCA_003361615	E	Canada	11	O157:H7	Plasmid	Cattle	ehxCABD	20
GCA_002176745	E	USA	11	O157:H7	Not determined	Unknown	ehxCABD	20
GCA_004228985	B1	USA	8794	?:H2	Not determined	Unknown	ehxCABD	15
GCA_004230565	B1	USA	655	O121:H19	Plasmid	Unknown	ehxCABD	18
GCA_000234275	B1	USA	655	O121:H19	Plasmid	Human	ehxCABD	18
GCA_005039315	B1	Canada	7715	O130:H38	Plasmid	Cattle	ehxCABD	25
GCA_004767145	B1	Canada	2385	?:H19	Plasmid	Human	ehxCABD	21
GCA_002531375	E	Canada	11	O157:H7	Plasmid	Unknown	ehxCABD	20
GCA_002923635	B1	Japan	21	O26:H11	Plasmid	Human	ehxCABD	27
GCA_005392045	A	Japan	119	O165:H25	Plasmid	Cattle	ehxCABD	26
GCA_004231535	B1	USA	16	O111:H8	Not determined	Unknown	ehxCABD	27
GCA_001609235	B1	Netherlands	350	O146:H21	Plasmid	Human	ehxCABD	17
GCA_003361955	E	Canada	11	O157:H7	Plasmid	Human	ehxCABD	20
GCA_008635565	D	Japan	32	?:H28	Plasmid	Human	ehxCABD	8
GCA_002764935	B1	Japan	21	O26:H11	Plasmid	Human	ehxCABD	27
GCA_001607815	E	Netherlands	6041	Onovel3:H20	Plasmid	Human	ehxCABD	11
GCA_004233265	E	USA	5516	O157:H7	Plasmid	Unknown	ehxCABD	20
GCA_003362555	E	Canada	11	O157:H7	Plasmid	Human	ehxCABD	20
GCA_002924145	B1	Japan	21	O26:H11	Plasmid	Human	ehxCABD	27
GCA_003900655	B1	USA	21	?:H11	Not determined	Unknown	ehxCABD	27
GCA_005392975	B1	Japan	223	O175:H21	Plasmid	Cattle	ehxCABD	22
GCA_001608105	B1	Netherlands	17	O103:H2	Not determined	Human	ehxCABD	15
GCA_004231685	B1	USA	21	?:H16	Plasmid	Unknown	ehxCABD	27
GCA_002165415	E	USA	11	?:H7	Plasmid	Cattle	ehxCABD	20
GCA_002016165	B1	USA	17	O103:H2	Plasmid	Cattle	ehxCABD	15
GCA_003773905	B1	USA	17	?:H2	Not determined	Unknown	ehxCABD	15
GCA_005044445	B1	Canada	17	O103:H2	Not determined	Human	ehxCABD	15
GCA_003362455	E	Canada	11	O157:H7	Plasmid	Human	ehxCABD	20
GCA_003901125	B1	USA	480	O111:H8	Not determined	Unknown	ehxCABD	27
GCA_002486775	E	USA	11	O157:H7	Plasmid	Unknown	ehxCABD	20
GCA_003418605	E	Unknown	11	O157:H7	Plasmid	Unknown	ehxCABD	20
GCA_005038525	B1	Canada	21	O26:H11	Not determined	Cattle	ehxCABD	27
GCA_003905575	B1	USA	21	O26:H11	Plasmid	Unknown	ehxCABD	27
GCA_004796635	D	USA	32	O145:H28	Plasmid	Cattle	ehxCABD	8
GCA_004273465	E	United Kingdom	11	O157:H7	Plasmid	Human	ehxCABD	20
GCA_003361875	E	Canada	11	O157:H7	Plasmid	Unknown	ehxCABD	20
GCA_005039995	A	Canada	342	O5:NA	Plasmid	Unknown	ehxCABD	26
GCA_004162095	C	United Kingdom	3101	?:H4	Not determined	Human	ehxCABD	16
GCA_001608245	A	Netherlands	548	O38:H26	Plasmid	Human	ehxCABD	24
GCA_003760905	E	USA	11	?:H7	Plasmid	Unknown	ehxCABD	20
GCA_000462645	E	Unknown	11	O157:H7	Plasmid	Unknown	ehxCABD	20
GCA_000622555	B1	USA	21	O26:H11	Plasmid	Unknown	ehxCABD	27
GCA_003754925	E	USA	11	O157:H7	Not determined	Unknown	ehxCABD	20
GCA_002766275	B1	Japan	21	O26:H11	Plasmid	Human	ehxCABD	27
GCA_005043245	B1	Canada	21	O26:H11	Plasmid	Unknown	ehxCABD	27
GCA_000215285	B1	Unknown	58	O153var1:H25	Plasmid	Cattle	ehxCABD	22
GCA_000462045	E	Unknown	11	O157:H7	Plasmid	Unknown	ehxCABD	20
GCA_001012405	B1	USA	17	O45:H2	Not determined	Human	ehxCABD	15
GCA_005392545	B1	Japan	300	O182:H25	Not determined	Cattle	ehxCABD	14
GCA_003759535	E	USA	822	O157:H7	Plasmid	Unknown	ehxCABD	20
GCA_002806745	E	USA	11	O157:H7	Plasmid	Human	ehxCABD	20
GCA_003864655	B1	Japan	655	O121:H19	Plasmid	Human	ehxCABD	18
GCA_004767025	B1	USA	6661	?:H19	Plasmid	Unknown	ehxCABD	21
GCA_005039985	B1	Unknown	21	O26:H11	Plasmid	Human	ehxCABD	27
GCA_003360265	E	Canada	-	O157:H7	Plasmid	Human	ehxCABD	20
GCA_003766005	E	USA	11	?:H7	Plasmid	Unknown	ehxCABD	20
GCA_002476215	E	USA	11	O157:H7	Plasmid	Unknown	ehxCABD	20
GCA_001309685	B1	Canada	343	O103:H25	Not determined	Unknown	ehxCABD	14
GCA_001606635	B1	Netherlands	17	O103:H2	Not determined	Human	ehxCABD	15
GCA_002834485	B1	USA	29	O26:H11	Plasmid	Cattle	ehxCABD	27
GCA_004262885	E	United Kingdom	11	O157:H7	Plasmid	Human	ehxCABD	20
GCA_002764875	B1	Japan	21	O26:H11	Plasmid	Human	ehxCABD	27
GCA_004257585	B1	United Kingdom	21	O26:H11	Plasmid	Human	ehxCABD	27
GCA_000614685	E	USA	11	O157:H7	Plasmid	Unknown	ehxCABD	20
GCA_000632675	B1	USA	16	O111:H8	Not determined	Unknown	ehxCABD	27
GCA_002286435	B1	Israel	481	O26:H11	Plasmid	Human	ehxCABD	27
GCA_002015385	B1	USA	7211	O103:H2	Plasmid	Cattle	ehxCABD	15
GCA_004232705	B1	USA	16	?:H8	Not determined	Unknown	ehxCABD	27
GCA_002475845	E	USA	11	O157:H7	Plasmid	Unknown	ehxCABD	20
GCA_004265505	E	United Kingdom	628	O157:H7	Plasmid	Human	ehxCABD	20
GCA_002133775	E	Canada	11	O157:H7	Plasmid	Unknown	ehxCABD	20
GCA_002194915	E	USA	11	O157:H7	Plasmid	Cattle	ehxCABD	20
GCA_002764495	B1	Japan	21	O26:H11	Not determined	Cattle	ehxCABD	27
GCA_004174775	B1	United Kingdom	16	O111:H8	Not determined	Human	ehxCABD	27
GCA_003362535	E	Canada	11	O157:H7	Plasmid	Human	ehxCABD	20
GCA_001309995	E	Canada	11	O157:H7	Plasmid	Cattle	ehxCABD	20
GCA_005041935	B1	Canada	679	O163:H19	Plasmid	Unknown	ehxCABD	10
GCA_004232485	A	USA	10	O113:H4	Plasmid	Unknown	ehxCABD	24

GCA_004281265	B1	United Kingdom	442	O146:H21	Plasmid	Human	ehxCABD	17
GCA_003764265	B1	USA	17	?:H2	Not determined	Unknown	ehxCABD	15
GCA_005040305	B1	Canada	302	O139:H19	Plasmid	Unknown	ehxCABD	25
GCA_003359495	E	Canada	11	O157:H7	Plasmid	Unknown	ehxCABD	20
GCA_000622855	B1	USA	21	O26:H11	Plasmid	Unknown	ehxCABD	27
GCA_000617525	B1	USA	16	O111:H8	Not determined	Unknown	ehxCABD	27
GCA_002531175	B1	Canada	16	O111:H8	Not determined	Unknown	ehxCABD	27
GCA_004232645	E	USA	11	O157:H7	Plasmid	Unknown	ehxCABD	20
GCA_005390605	B1	Japan	679	?:H19	Plasmid	Cattle	ehxCABD	10
GCA_009495495	E	Malaysia	11	O157:H7	Plasmid	Unknown	ehxCABD	20
GCA_003738305	B1	USA	655	O121:H19	Plasmid	Unknown	ehxCABD	18
GCA_005390585	B1	Japan	295	?:H16	Plasmid	Cattle	ehxCABD	25
GCA_005041735	B1	Canada	25	O128:H2	Plasmid	Unknown	ehxCABD	13
GCA_002735165	B1	Belgium	481	O26:H11	Plasmid	Cattle	ehxCABD	27
GCA_003876975	B1	United Kingdom	442	?:H21	Plasmid	Human	ehxCABD	17
GCA_002923555	B1	Japan	21	O26:H11	Plasmid	Human	ehxCABD	27
GCA_005045745	A	Denmark	10	O113:H4	Plasmid	Unknown	ehxCABD	24
GCA_004261245	E	United Kingdom	11	O157:H7	Plasmid	Human	ehxCABD	20
GCA_005044195	B1	Canada	16	O111:H8	Not determined	Human	ehxCABD	27
GCA_003881135	E	United Kingdom	11	O157:H7	Plasmid	Human	ehxCABD	20
GCA_004261385	B1	United Kingdom	21	O26:H11	Plasmid	Human	ehxCABD	27
GCA_002476015	E	USA	11	O157:H7	Plasmid	Unknown	ehxCABD	20
GCA_005044495	A	Canada	6629	O49:NA	Plasmid	Human	ehxCABD	25
GCA_001571915	B1	USA	29	O26:H11	Plasmid	Cattle	ehxCABD	27
GCA_003766745	B1	USA	17	?:H2	Not determined	Unknown	ehxCABD	15
GCA_009896495	E	USA	11	O157:H7	Plasmid	Cattle	ehxCABD	20
GCA_004233825	E	USA	11	O157:H7	Plasmid	Unknown	ehxCABD	20
GCA_008756325	B1	USA	16	O111:H8	Not determined	Human	ehxCABD	27
GCA_003864895	B1	Japan	655	O121:H19	Plasmid	Human	ehxCABD	18
GCA_002474135	E	USA	11	O157:H7	Plasmid	Unknown	ehxCABD	20
GCA_003895485	E	USA	11	O157:H7	Plasmid	Unknown	ehxCABD	20
GCA_003879255	E	United Kingdom	11	O157:H7	Plasmid	Human	ehxCABD	20
GCA_001309835	D	Canada	137	O145:H28	Not determined	Unknown	ehxCABD	8
GCA_003908235	Unknown	United Kingdom	1819	?:H28	Plasmid	Human	ehxCABD	23
GCA_004232205	E	USA	11	O157:H7	Plasmid	Unknown	ehxCABD	20
GCA_001606385	B1	Netherlands	17	O103:H2	Not determined	Human	ehxCABD	15
GCA_001607755	B1	Netherlands	442	O91:H21	Plasmid	Human	ehxCABD	17
GCA_000335095	E	Unknown	11	O157:H7	Plasmid	Unknown	ehxCABD	20
GCA_005042575	E	Canada	11	O157:H7	Plasmid	Unknown	ehxCABD	20
GCA_000447085	B1	Guinea-Bissau	17	O103:H2	Not determined	Pig	ehxCABD	15
GCA_001606545	B1	Netherlands	17	O103:H2	Not determined	Human	ehxCABD	15
GCA_003758425	B1	USA	17	O45:H2	Plasmid	Unknown	ehxCABD	15
GCA_005046005	B1	Switzerland	25	O128:H2	Plasmid	Human	ehxCABD	13
GCA_005040355	E	Canada	11	O157:H7	Plasmid	Unknown	ehxCABD	20
GCA_002923875	B1	Japan	21	O26:H11	Plasmid	Human	ehxCABD	27
GCA_005042485	B1	Canada	655	O121:H19	Plasmid	Cattle	ehxCABD	18
GCA_002735145	B1	United Kingdom	21	O26:H11	Plasmid	Human	ehxCABD	27
GCA_002734785	B1	USA	21	O26:H11	Plasmid	Cattle	ehxCABD	27
GCA_003766465	E	USA	11	O157:H7	Plasmid	Unknown	ehxCABD	20
GCA_004281195	B1	United Kingdom	8393	O140:H21	Plasmid	Unknown	ehxCABD	25
GCA_001607355	B1	Netherlands	33	O91:H14	Plasmid	Human	ehxCABD	12
GCA_005397045	B1	Japan	327	O177:H11	Plasmid	Cattle	ehxCABD	27
GCA_004231085	B1	USA	21	O26:H11	Plasmid	Unknown	ehxCABD	27
GCA_003753925	B1	USA	16	?:H8	Not determined	Unknown	ehxCABD	27
GCA_003756605	B1	USA	21	O26:H11	Plasmid	Unknown	ehxCABD	27
GCA_005040855	B1	Canada	306	O98:H21	Plasmid	Unknown	ehxCABD	14
GCA_003340725	B1	USA	29	O26:H11	Plasmid	Cattle	ehxCABD	27
GCA_003923535	E	USA	822	O157:H7	Plasmid	Unknown	ehxCABD	20
GCA_004231395	E	USA	11	O157:H7	Plasmid	Unknown	ehxCABD	20
GCA_008633505	D	Japan	32	?:H28	Plasmid	Human	ehxCABD	8
GCA_003881915	E	United Kingdom	11	O157:H7	Plasmid	Human	ehxCABD	20
GCA_003767865	B1	USA	16	?:H8	Not determined	Unknown	ehxCABD	27
GCA_003360175	E	Canada	11	O157:H7	Plasmid	Human	ehxCABD	20
GCA_000316905	E	Unknown	11	O157:H7	Plasmid	Unknown	ehxCABD	20
GCA_003924115	E	USA	11	?:H7	Plasmid	Unknown	ehxCABD	20
GCA_005392225	B1	Japan	718	O168:H8	Plasmid	Cattle	ehxCABD	25
GCA_005390445	B1	Japan	295	Onovel1:H16	Plasmid	Cattle	ehxCABD	25
GCA_002765355	B1	Japan	21	O26:H11	Plasmid	Human	ehxCABD	27
GCA_005384005	B1	Japan	5973	Onovel21:H2	Plasmid	Cattle	ehxCABD	25
GCA_002768535	B1	Japan	21	O26:H11	Plasmid	Human	ehxCABD	27
GCA_003916435	B1	United Kingdom	8650	O152:H8	Plasmid	Human	ehxCABD	25
GCA_002175865	E	USA	11	O157:H7	Not determined	Cattle	ehxCABD	20
GCA_005040155	B1	Canada	21	O26:H11	Plasmid	Unknown	ehxCABD	27
GCA_003303975	B1	China	88	O109:H40	Plasmid	Human	ehxCABD	25
GCA_002923675	B1	Japan	21	O26:H11	Plasmid	Human	ehxCABD	27
GCA_005046855	D	Argentina	32	O145:H28	Plasmid	Human	ehxCABD	8
GCA_000617805	B1	USA	16	O111:H8	Not determined	Unknown	ehxCABD	27
GCA_003922435	E	United Kingdom	11	O157:H7	Plasmid	Unknown	ehxCABD	20

GCA_005380505	B1	Japan	655	O121:H19	Plasmid	Human	ehxCABD	18
GCA_001677715	E	Argentina	11	O157:H7	Plasmid	Human	ehxCABD	20
GCA_002924005	B1	Japan	21	O26:H11	Plasmid	Human	ehxCABD	27
GCA_003347195	E	Romania	628	O157:H7	Plasmid	Human	ehxCABD	20
GCA_003787565	E	USA	11	O157:H7	Plasmid	Unknown	ehxCABD	20
GCA_003884075	B1	United Kingdom	200	O187:H28	Not determined	Human	ehxCABD	25
GCA_002766835	B1	Japan	21	O26:H11	Plasmid	Human	ehxCABD	27
GCA_002768595	B1	Japan	21	O26:H11	Plasmid	Human	ehxCABD	27
GCA_005045795	E	Canada	9054	Onovel3:H20	Plasmid	Human	ehxCABD	11
GCA_003362375	E	Canada	11	O157:H7	Plasmid	Human	ehxCABD	20
GCA_004228945	E	USA	11	?:H7	Plasmid	Unknown	ehxCABD	20
GCA_003361495	E	Canada	11	O157:H7	Plasmid	Unknown	ehxCABD	20
GCA_003741145	B1	USA	21	?:H16	Plasmid	Unknown	ehxCABD	27
GCA_008634465	D	Japan	32	O145:H28	Plasmid	Human	ehxCABD	8
GCA_001616755	B1	China	13	O174:H8	Plasmid	Sheep	ehxCABD	25
GCA_000965565	D	Norway	-	O145:H28	Plasmid	Human	ehxCABD	8
GCA_003361795	E	Canada	11	O157:H7	Plasmid	Unknown	ehxCABD	20
GCA_001012275	B1	USA	16	O111:H8	Not determined	Unknown	ehxCABD	27
GCA_002766355	B1	Japan	21	O26:H11	Plasmid	Human	ehxCABD	27
GCA_000615655	B1	USA	16	O111:H8	Not determined	Unknown	ehxCABD	27
GCA_005038535	B1	Canada	154	O88:H25	Plasmid	Cattle	ehxCABD	25
GCA_005039175	B1	Canada	21	O111:H11	Not determined	Cattle	ehxCABD	27
GCA_004275675	B1	United Kingdom	21	O26:H11	Plasmid	Human	ehxCABD	27
GCA_001884945	E	USA	11	?:H7	Plasmid	Cattle	ehxCABD	20
GCA_008635855	D	Japan	32	?:H28	Plasmid	Human	ehxCABD	8
GCA_000617285	B1	USA	655	O121:H19	Plasmid	Unknown	ehxCABD	18
GCA_004160775	B1	United Kingdom	25	O128:H2	Plasmid	Human	ehxCABD	13
GCA_002766875	B1	Japan	21	O26:H11	Plasmid	Human	ehxCABD	27
GCA_002379295	B1	USA	223	O113:H21	Plasmid	Cattle	ehxCABD	22
GCA_001608265	A	Netherlands	119	O165:H25	Plasmid	Human	ehxCABD	26
GCA_005044075	B1	Canada	16	O111:H8	Not determined	Human	ehxCABD	27
GCA_004252705	B1	United Kingdom	17	O103:H2	Not determined	Human	ehxCABD	15
GCA_002770095	B1	Japan	21	O26:H11	Plasmid	Human	ehxCABD	27
GCA_004183215	E	United Kingdom	11	O157:H7	Plasmid	Human	ehxCABD	20
GCA_003903655	E	United Kingdom	11	O157:H7	Plasmid	Human	ehxCABD	20
GCA_005045285	B1	Denmark	415	O76:H19	Plasmid	Unknown	ehxCABD	25
GCA_005045155	A	USA	119	O165:H25	Plasmid	Unknown	ehxCABD	26
GCA_001884965	E	USA	11	O157:H7	Plasmid	Cattle	ehxCABD	20
GCA_005397145	B1	Japan	205	?:H19	Plasmid	Cattle	ehxCABD	25
GCA_004228785	E	USA	11	O157:H7	Plasmid	Unknown	ehxCABD	20
GCA_002179775	E	USA	11	O157:H7	Plasmid	Cattle	ehxCABD	20
GCA_002834405	B1	USA	29	?:H11	Plasmid	Cattle	ehxCABD	27
GCA_002765255	B1	Japan	21	O26:H11	Plasmid	Human	ehxCABD	27
GCA_003768465	E	USA	11	?:H7	Plasmid	Unknown	ehxCABD	20
GCA_004230965	E	USA	628	?:H7	Plasmid	Unknown	ehxCABD	20
GCA_002164525	E	USA	11	O157:H7	Plasmid	Cattle	ehxCABD	20
GCA_003885435	E	United Kingdom	11	O157:H7	Plasmid	Human	ehxCABD	20
GCA_005380745	B1	Japan	655	O121:H19	Plasmid	Human	ehxCABD	18
GCA_003347255	E	Romania	1804	O157:H7	Plasmid	Human	ehxCABD	20
GCA_002176015	E	USA	11	O157:H7	Plasmid	Cattle	ehxCABD	20
GCA_004273745	E	United Kingdom	11	?:H7	Plasmid	Unknown	ehxCABD	20
GCA_005395645	B1	Japan	101	?:H8	Plasmid	Cattle	ehxCABD	25
GCA_005390325	B1	Japan	7883	?:H19	Plasmid	Cattle	ehxCABD	25
GCA_003123335	A	Spain	301	O80:H2	Not determined	Human	ehxCABD	19
GCA_000616135	B1	USA	5536	O121:H19	Not determined	Unknown	ehxCABD	18
GCA_003756625	D	USA	32	?:H28	Plasmid	Unknown	ehxCABD	8
GCA_000335115	E	Unknown	11	O157:H7	Plasmid	Unknown	ehxCABD	20
GCA_002475825	E	USA	11	O157:H7	Plasmid	Unknown	ehxCABD	20
GCA_005390865	D	Japan	137	OgN9:H28	Plasmid	Cattle	ehxCABD	8
GCA_002164435	E	USA	11	O157:H7	Plasmid	Cattle	ehxCABD	20
GCA_003737825	E	USA	11	O157:H7	Plasmid	Unknown	ehxCABD	20
GCA_005044845	B1	Canada	21	O26:H11	Plasmid	Human	ehxCABD	27
GCA_002765335	B1	Japan	21	O26:H11	Plasmid	Human	ehxCABD	27
GCA_005394905	B1	Japan	156	?:H28	Plasmid	Cattle	ehxCABD	25
GCA_000335255	E	Unknown	11	O157:H7	Plasmid	Unknown	ehxCABD	20
GCA_000616505	E	USA	11	O157:H7	Plasmid	Unknown	ehxCABD	20
GCA_003917495	E	United Kingdom	1804	O157:H7	Plasmid	Human	ehxCABD	20
GCA_003360695	E	Canada	11	O157:H7	Plasmid	Human	ehxCABD	20
GCA_002767825	B1	Japan	21	O26:H11	Plasmid	Human	ehxCABD	27
GCA_004234305	E	USA	11	O157:H7	Plasmid	Unknown	ehxCABD	20
GCA_002770015	B1	Japan	21	O26:H11	Plasmid	Human	ehxCABD	27
GCA_003908595	B1	United Kingdom	16	?:H8	Not determined	Human	ehxCABD	27
GCA_000619545	B1	USA	16	O111:H8	Not determined	Unknown	ehxCABD	27
GCA_900450535	E	Unknown	11	O157:H7	Plasmid	Human	ehxCABD	20
GCA_003293975	B1	Canada	16	O111:H8	Not determined	Human	ehxCABD	27
GCA_002766535	B1	Japan	21	O26:H11	Plasmid	Human	ehxCABD	27
GCA_002164285	E	USA	11	O157:H7	Plasmid	Cattle	ehxCABD	20
GCA_002844415	B1	USA	29	O26:H11	Plasmid	Cattle	ehxCABD	27

GCA_002461735	E	USA	11	O157:H7	Plasmid	Human	ehxCABD	20
GCA_002767555	B1	Japan	21	O26:H11	Plasmid	Human	ehxCABD	27
GCA_000462025	E	Unknown	11	O157:H7	Plasmid	Unknown	ehxCABD	20
GCA_003917295	B1	USA	480	?:H8	Not determined	Unknown	ehxCABD	27
GCA_008634445	D	Japan	32	O145:H28	Plasmid	Human	ehxCABD	8
GCA_005046615	B1	Switzerland	17	O103:H2	Not determined	Human	ehxCABD	15
GCA_003903035	E	United Kingdom	11	O157:H7	Plasmid	Human	ehxCABD	20
GCA_002475775	E	USA	11	O157:H7	Plasmid	Unknown	ehxCABD	20
GCA_003920015	B1	United Kingdom	21	O26:H11	Plasmid	Human	ehxCABD	27
GCA_003360425	E	Canada	11	O157:H7	Plasmid	Human	ehxCABD	20
GCA_002531305	E	Canada	11	O157:H7	Plasmid	Unknown	ehxCABD	20
GCA_005045715	B1	Denmark	811	O128:H2	Not determined	Unknown	ehxCABD	13
GCA_002764895	B1	Japan	21	O26:H11	Plasmid	Human	ehxCABD	27
GCA_001608315	B1	Netherlands	33	O91:H14	Plasmid	Human	ehxCABD	12
GCA_003770545	B1	USA	21	O71:H11	Plasmid	Unknown	ehxCABD	27
GCA_003760985	D	USA	32	O145:H28	Plasmid	Unknown	ehxCABD	8
GCA_008634485	D	Japan	32	O145:H28	Plasmid	Human	ehxCABD	8
GCA_001606595	A	Netherlands	342	O5:NA	Plasmid	Human	ehxCABD	26
GCA_001607075	B1	Netherlands	29	O26:H11	Plasmid	Human	ehxCABD	27
GCA_002769895	B1	Japan	21	O26:H11	Plasmid	Human	ehxCABD	27
GCA_001608155	B1	Netherlands	26	O8:H8	Plasmid	Human	ehxCABD	25
GCA_004232985	B1	USA	21	O26:H11	Plasmid	Unknown	ehxCABD	27
GCA_002286535	B1	Israel	481	O71:H8	Not determined	Human	ehxCABD	27
GCA_003761025	B1	USA	16	?:H8	Not determined	Unknown	ehxCABD	27
GCA_004157695	B1	United Kingdom	25	O128:H2	Plasmid	Human	ehxCABD	13
GCA_000477495	E	Unknown	11	O157:H7	Plasmid	Unknown	ehxCABD	20
GCA_002837355	B1	USA	29	?:H11	Plasmid	Cattle	ehxCABD	27
GCA_000614305	E	USA	11	O157:H7	Plasmid	Unknown	ehxCABD	20
GCA_002486575	E	USA	11	O157:H7	Plasmid	Unknown	ehxCABD	20
GCA_000617645	B1	USA	655	O121:H19	Plasmid	Unknown	ehxCABD	18
GCA_004234225	B1	USA	655	O121:H19	Plasmid	Unknown	ehxCABD	18
GCA_003755565	E	USA	11	O157:H7	Plasmid	Unknown	ehxCABD	20
GCA_004232625	E	USA	11	?:H7	Plasmid	Unknown	ehxCABD	20
GCA_002769055	B1	Belgium	21	O26:H11	Plasmid	Human	ehxCABD	27
GCA_000461915	E	Unknown	11	O157:H7	Plasmid	Unknown	ehxCABD	20
GCA_002768575	B1	Japan	21	O26:H11	Plasmid	Human	ehxCABD	27
GCA_002765575	B1	Japan	21	O26:H11	Plasmid	Human	ehxCABD	27
GCA_005039015	B1	Canada	7715	O130:H38	Plasmid	Unknown	ehxCABD	25
GCA_002549265	B1	USA	655	O121:H19	Plasmid	Wild animals	ehxCABD	18
GCA_000234235	B1	Canada	223	O113:H21	Plasmid	Human	ehxCABD	22
GCA_000267105	E	Unknown	11	O157:H7	Plasmid	Unknown	ehxCABD	20
GCA_001997045	E	Unknown	11	O157:H7	Plasmid	Unknown	ehxCABD	20
GCA_000940035	B1	Norway	295	?:H11	Plasmid	Human	ehxCABD	25
GCA_002164515	E	USA	11	O157:H7	Plasmid	Cattle	ehxCABD	20
GCA_005038655	B1	Canada	17	O103:H2	Not determined	Cattle	ehxCABD	15
GCA_002795105	E	USA	11	?:H7	Plasmid	Human	ehxCABD	20
GCA_001607675	B1	Netherlands	1786	O103:H2	Not determined	Human	ehxCABD	15
GCA_007648995	B1	Brazil	21	O26:H11	Plasmid	Human	ehxCABD	27
GCA_005391005	B1	Japan	327	?:H11	Plasmid	Cattle	ehxCABD	27
GCA_003027145	E	Austria	11	?:H7	Plasmid	Goat	ehxCABD	20
GCA_005042225	B1	Canada	2385	?:H19	Plasmid	Unknown	ehxCABD	21
GCA_000462785	E	Unknown	11	O157:H7	Plasmid	Unknown	ehxCABD	20
GCA_001660195	B1	Germany	6837	O146:H21	Plasmid	Sheep	ehxCABD	17
GCA_002764335	B1	Belgium	21	O26:H11	Plasmid	Cattle	ehxCABD	27
GCA_001281815	E	Netherlands	11	O157:H7	Plasmid	Human	ehxCABD	20
GCA_003766785	E	USA	11	O157:H7	Plasmid	Unknown	ehxCABD	20
GCA_001309885	E	Canada	11	O157:H7	Plasmid	Unknown	ehxCABD	20
GCA_005044215	E	Canada	691	Onovel3:H20	Plasmid	Human	ehxCABD	11
GCA_008755475	B1	USA	16	O111:H8	Not determined	Human	ehxCABD	27
GCA_004264465	B1	United Kingdom	25	O21:H2	Plasmid	Human	ehxCABD	13
GCA_005392165	A	Japan	206	O49:H10	Not determined	Cattle	ehxCABD	23
GCA_005380945	B1	Japan	655	O121:H19	Plasmid	Human	ehxCABD	18
GCA_002844475	B1	USA	29	O26:H11	Plasmid	Cattle	ehxCABD	27
GCA_002769515	B1	Japan	21	O26:H11	Plasmid	Human	ehxCABD	27
GCA_004231785	E	USA	11	?:H7	Plasmid	Unknown	ehxCABD	20
GCA_002458325	E	Unknown	11	O157:H7	Plasmid	Human	ehxCABD	20
GCA_003774025	D	USA	32	?:H28	Plasmid	Unknown	ehxCABD	8
GCA_003360655	E	Canada	11	O157:H7	Plasmid	Human	ehxCABD	20
GCA_003740765	E	USA	11	O157:H7	Plasmid	Unknown	ehxCABD	20
GCA_005394365	B1	Japan	111	?:H49	Plasmid	Cattle	ehxCABD	25
GCA_000335135	E	Unknown	11	O157:H7	Plasmid	Unknown	ehxCABD	20
GCA_005039135	A	Canada	5486	O165:H25	Plasmid	Cattle	ehxCABD	26
GCA_005046685	B1	Canada	17	O103:H2	Not determined	Unknown	ehxCABD	15
GCA_005046555	B1	Canada	17	O45:H2	Not determined	Human	ehxCABD	15
GCA_002765475	B1	Japan	21	O26:H11	Plasmid	Human	ehxCABD	27
GCA_003894455	B1	United Kingdom	21	?:H11	Plasmid	Human	ehxCABD	27
GCA_002473955	E	USA	11	O157:H7	Plasmid	Unknown	ehxCABD	20
GCA_005042395	B1	Canada	679	O163:H19	Plasmid	Unknown	ehxCABD	10

GCA_005038425	B1	Canada	21	O111:H11	Not determined	Cattle	ehxCABD	27
GCA_001607155	B1	Netherlands	25	O128:H2	Plasmid	Human	ehxCABD	13
GCA_005393905	B1	Japan	29	?:H11	Plasmid	Cattle	ehxCABD	27
GCA_002915085	B1	New Zealand	17	O103:H2	Not determined	Wild animals	ehxCABD	15
GCA_002764835	B1	Japan	21	O26:H11	Plasmid	Human	ehxCABD	27
GCA_003123565	A	France	301	O80:H2	Plasmid	Human	ehxCABD	19
GCA_003113015	B1	Japan	29	?:H11	Plasmid	Human	ehxCABD	27
GCA_002765635	B1	Japan	21	O26:H11	Plasmid	Human	ehxCABD	27
GCA_005040545	E	Canada	11	O157:H7	Plasmid	Unknown	ehxCABD	20
GCA_005042585	B1	Canada	223	?:H21	Plasmid	Cattle	ehxCABD	22
GCA_001607285	B1	Netherlands	33	O91:H14	Plasmid	Human	ehxCABD	12
GCA_001616775	B1	China	17	O45:H2	Not determined	Sheep	ehxCABD	15
GCA_003769285	D	USA	32	?:NA	Plasmid	Unknown	ehxCABD	8
GCA_003340715	B1	USA	29	O26:H11	Plasmid	Cattle	ehxCABD	27
GCA_002027645	E	Canada	11	?:H7	Plasmid	Cattle	ehxCABD	20
GCA_001616105	C	China	7	?:H4	Not determined	Pig	ehxCABD	16
GCA_002765915	B1	Japan	21	O26:H11	Plasmid	Human	ehxCABD	27
GCA_003736465	B1	USA	16	?:H8	Not determined	Unknown	ehxCABD	27
GCA_003893555	E	United Kingdom	11	O157:H7	Plasmid	Human	ehxCABD	20
GCA_002768515	B1	Japan	21	O26:H11	Plasmid	Human	ehxCABD	27
GCA_005042055	B1	Canada	679	?:H19	Plasmid	Unknown	ehxCABD	10
GCA_002924105	B1	Japan	21	O26:H11	Plasmid	Human	ehxCABD	27
GCA_005042475	B1	Canada	655	O121:H19	Plasmid	Cattle	ehxCABD	18
GCA_005039325	B1	Canada	21	O26:H11	Plasmid	Cattle	ehxCABD	27
GCA_007648325	A	Brazil	301	O76:H2	Plasmid	Human	ehxCABD	19
GCA_008634575	D	Japan	32	O145:H28	Plasmid	Human	ehxCABD	8
GCA_003896335	D	USA	137	?:H28	Not determined	Unknown	ehxCABD	8
GCA_003887535	B1	United Kingdom	442	O146:H21	Plasmid	Human	ehxCABD	17
GCA_003741105	E	USA	11	?:H7	Plasmid	Unknown	ehxCABD	20
GCA_000965635	B1	Norway	655	O121:H19	Plasmid	Human	ehxCABD	18
GCA_004258745	A	United Kingdom	10	O113:H4	Plasmid	Human	ehxCABD	24
GCA_003360355	E	Canada	11	O157:H7	Plasmid	Human	ehxCABD	20
GCA_000234315	D	Germany	32	O145:H28	Plasmid	Human	ehxCABD	8
GCA_005042625	B1	Canada	343	O103:H25	Not determined	Cattle	ehxCABD	14
GCA_005400705	B1	France	327	O177:H11	Plasmid	Cattle	ehxCABD	27
GCA_000335315	E	Unknown	11	?:H7	Plasmid	Unknown	ehxCABD	20
GCA_002134405	B1	Canada	17	O103:H2	Not determined	Unknown	ehxCABD	15
GCA_004230805	B1	USA	21	O26:H11	Not determined	Unknown	ehxCABD	27
GCA_008635915	B1	Japan	591	?:H25	Not determined	Human	ehxCABD	14
GCA_005042505	B1	Canada	21	O26:H11	Plasmid	Unknown	ehxCABD	27
GCA_003777385	B1	USA	17	O103:H2	Not determined	Unknown	ehxCABD	15
GCA_003359775	E	Canada	11	?:H7	Plasmid	Human	ehxCABD	20
GCA_003362235	E	Canada	11	O157:H7	Plasmid	Cattle	ehxCABD	20
GCA_005037875	B1	Unknown	7715	O130:H38	Plasmid	Pig	ehxCABD	25
GCA_001608015	B1	Netherlands	21	O26:H11	Plasmid	Human	ehxCABD	27
GCA_002764535	B1	Japan	21	O26:H11	Plasmid	Human	ehxCABD	27
GCA_003361755	E	Canada	11	O157:H7	Plasmid	Cattle	ehxCABD	20
GCA_000615225	D	USA	32	O145:H28	Plasmid	Unknown	ehxCABD	8
GCA_005039215	B1	Canada	21	O111:H11	Not determined	Cattle	ehxCABD	27
GCA_002923435	B1	Japan	21	O26:H11	Plasmid	Sheep	ehxCABD	27
GCA_003361535	E	Canada	11	O157:H7	Plasmid	Unknown	ehxCABD	20
GCA_002133755	B1	Canada	17	O103:H2	Not determined	Unknown	ehxCABD	15
GCA_000616725	B1	USA	655	O121:H19	Plasmid	Unknown	ehxCABD	18
GCA_001990935	B1	Germany	223	O113:H21	Plasmid	Unknown	ehxCABD	22
GCA_008756255	B1	USA	16	?:H8	Not determined	Human	ehxCABD	27
GCA_002476305	B1	USA	415	O76:H19	Not determined	Unknown	ehxCABD	25
GCA_003359555	E	Canada	11	O157:H7	Plasmid	Unknown	ehxCABD	20
GCA_008122325	E	United Kingdom	11	O157:H7	Plasmid	Human	ehxCABD	20
GCA_003740565	E	USA	11	O157:H7	Plasmid	Unknown	ehxCABD	20
GCA_003920995	B1	United Kingdom	675	O76:H19	Not determined	Human	ehxCABD	25
GCA_003361355	E	Canada	11	O157:H7	Plasmid	Unknown	ehxCABD	20
GCA_000462165	E	Unknown	11	O157:H7	Plasmid	Unknown	ehxCABD	20
GCA_003028095	A	Switzerland	301	?:H2	Not determined	Unknown	ehxCABD	19
GCA_002459225	B1	USA	655	O121:H19	Plasmid	Human	ehxCABD	18
GCA_000462245	E	Unknown	11	O157:H7	Plasmid	Unknown	ehxCABD	20
GCA_000619285	B1	USA	21	O69:H11	Plasmid	Unknown	ehxCABD	27
GCA_003765865	E	USA	11	O157:H7	Plasmid	Unknown	ehxCABD	20
GCA_003879155	E	United Kingdom	11	O157:H7	Plasmid	Human	ehxCABD	20
GCA_002765815	B1	Japan	21	O26:H11	Plasmid	Human	ehxCABD	27
GCA_002768915	B1	Belgium	21	O26:H11	Plasmid	Human	ehxCABD	27
GCA_001262805	B1	USA	2385	?:H19	Plasmid	Unknown	ehxCABD	21
GCA_003741205	B1	USA	21	O26:H11	Plasmid	Unknown	ehxCABD	27
GCA_002766695	B1	Japan	21	O26:H11	Plasmid	Human	ehxCABD	27
GCA_005040745	B1	Canada	154	O88:H25	Plasmid	Unknown	ehxCABD	25
GCA_002734925	B1	USA	21	O26:H11	Plasmid	Cattle	ehxCABD	27
GCA_001012445	B1	USA	17	O103:H2	Not determined	Human	ehxCABD	15
GCA_002286445	B1	Israel	481	O26:H11	Plasmid	Human	ehxCABD	27
GCA_003113135	B1	Japan	29	O26:H11	Plasmid	Human	ehxCABD	27

GCA_005042705	B1	Canada	679	O163:H19	Plasmid	Unknown	ehxCABD	10
GCA_002768315	B1	Japan	21	O26:H11	Plasmid	Human	ehxCABD	27
GCA_002766295	B1	Japan	21	O26:H11	Plasmid	Human	ehxCABD	27
GCA_003768445	B1	USA	2836	O117.Gp8:H8	Not determined	Unknown	ehxCABD	27
GCA_002134685	B1	Canada	655	O121:H19	Plasmid	Unknown	ehxCABD	18
GCA_005041905	B1	Canada	679	O163:H19	Plasmid	Unknown	ehxCABD	10
GCA_001990845	B1	Germany	442	O91:H21	Plasmid	Unknown	ehxCABD	17
GCA_002734905	B1	USA	29	O26:H11	Not determined	Cattle	ehxCABD	27
GCA_003360435	E	Canada	11	O157:H7	Plasmid	Human	ehxCABD	20
GCA_000615175	D	USA	32	O145:H28	Plasmid	Unknown	ehxCABD	8
GCA_002768775	B1	Japan	21	O26:H11	Plasmid	Human	ehxCABD	27
GCA_003757805	E	USA	11	O157:H7	Plasmid	Unknown	ehxCABD	20
GCA_000335355	E	Unknown	11	O157:H7	Plasmid	Unknown	ehxCABD	20
GCA_003361305	E	Canada	11	O157:H7	Plasmid	Cattle	ehxCABD	20
GCA_004162015	B1	United Kingdom	306	O84:H2	Plasmid	Human	ehxCABD	14
GCA_000619665	E	USA	11	O157:H7	Plasmid	Unknown	ehxCABD	20
GCA_002460005	B1	USA	58	O153var1:H25	Plasmid	Human	ehxCABD	22
GCA_001262935	B1	USA	58	O153var1:H25	Plasmid	Unknown	ehxCABD	22
GCA_005380825	B1	Japan	655	O121:H19	Plasmid	Human	ehxCABD	18
GCA_004161095	E	United Kingdom	11	O157:H7	Plasmid	Human	ehxCABD	20
GCA_000181735	E	Unknown	11	O157:H7	Plasmid	Unknown	ehxCABD	20
GCA_002768715	B1	Japan	21	O26:H11	Plasmid	Human	ehxCABD	27
GCA_005381185	B1	Japan	655	O121:H19	Plasmid	Human	ehxCABD	18
GCA_002764815	B1	Japan	21	O26:H11	Plasmid	Human	ehxCABD	27
GCA_001696245	B1	Canada	21	O26:H11	Plasmid	Human	ehxCABD	27
GCA_004766955	B1	New Zealand	21	O26:H11	Plasmid	Unknown	ehxCABD	27
GCA_002769715	B1	Japan	21	O26:H11	Plasmid	Human	ehxCABD	27
GCA_001191275	B1	USA	7543	O96:H19	Plasmid	Unknown	ehxCABD	25
GCA_002767875	B1	Japan	21	O26:H11	Plasmid	Human	ehxCABD	27
GCA_005380785	B1	Japan	655	O121:H19	Plasmid	Human	ehxCABD	18
GCA_005250655	A	France	301	O80:H2	Not determined	Human	ehxCABD	19
GCA_000335335	E	Unknown	11	O157:H7	Plasmid	Unknown	ehxCABD	20
GCA_004258065	E	United Kingdom	11	O157:H7	Plasmid	Human	ehxCABD	20
GCA_002473765	E	USA	11	O157:H7	Plasmid	Unknown	ehxCABD	20
GCA_004234385	E	USA	11	O157:H7	Plasmid	Unknown	ehxCABD	20
GCA_000462265	E	Unknown	11	O157:H7	Plasmid	Unknown	ehxCABD	20
GCA_008635685	D	Japan	32	?:H28	Plasmid	Human	ehxCABD	8
GCA_001677705	E	Argentina	11	O157:H7	Plasmid	Human	ehxCABD	20
GCA_002764675	B1	Japan	21	O26:H11	Plasmid	Human	ehxCABD	27
GCA_004190035	B1	United Kingdom	442	O146:H21	Plasmid	Human	ehxCABD	17
GCA_005043135	E	Canada	724	Onovel3:H20	Plasmid	Cattle	ehxCABD	11
GCA_003898495	B1	USA	17	O103:H2	Not determined	Unknown	ehxCABD	15
GCA_002164445	E	USA	11	O157:H7	Plasmid	Cattle	ehxCABD	20
GCA_003123395	A	France	301	O80:H2	Not determined	Cattle	ehxCABD	19
GCA_002134455	B1	Canada	33	?:H14	Not determined	Unknown	ehxCABD	12
GCA_002766755	B1	Japan	21	O26:H11	Plasmid	Human	ehxCABD	27
GCA_004259945	E	United Kingdom	11	O157:H7	Plasmid	Human	ehxCABD	20
GCA_004260635	B1	United Kingdom	21	O26:H11	Plasmid	Human	ehxCABD	27
GCA_002734825	B1	USA	21	O26:H11	Not determined	Cattle	ehxCABD	27
GCA_003359475	E	Canada	11	O157:H7	Plasmid	Unknown	ehxCABD	20
GCA_008633525	D	Japan	32	O145:H28	Plasmid	Human	ehxCABD	8
GCA_000619205	B1	USA	655	O121:H19	Plasmid	Unknown	ehxCABD	18
GCA_008634265	D	Japan	32	O145:H28	Plasmid	Human	ehxCABD	8
GCA_005044015	B1	Canada	655	O121:H19	Plasmid	Human	ehxCABD	18
GCA_005043095	B1	Canada	21	O26:H11	Plasmid	Unknown	ehxCABD	27
GCA_000462685	E	Unknown	11	O157:H7	Plasmid	Unknown	ehxCABD	20
GCA_000632575	B1	USA	21	O26:H11	Plasmid	Unknown	ehxCABD	27
GCA_003362045	E	Canada	11	O157:H7	Plasmid	Unknown	ehxCABD	20
GCA_003362315	E	Canada	11	O157:H7	Plasmid	Human	ehxCABD	20
GCA_002461565	B1	USA	16	O111:H8	Not determined	Human	ehxCABD	27
GCA_002766195	B1	Japan	21	O26:H11	Not determined	Human	ehxCABD	27
GCA_003768605	E	USA	11	O157:H7	Plasmid	Unknown	ehxCABD	20
GCA_003896675	E	USA	11	O157:H7	Plasmid	Unknown	ehxCABD	20
GCA_002765375	B1	Japan	21	O26:H11	Plasmid	Human	ehxCABD	27
GCA_005042385	B1	Canada	679	O163:H19	Plasmid	Unknown	ehxCABD	10
GCA_003912995	D	United Kingdom	32	O145:H28	Plasmid	Human	ehxCABD	8
GCA_004230845	E	USA	11	O157:H7	Plasmid	Unknown	ehxCABD	20
GCA_002764315	B1	Belgium	21	O26:H11	Plasmid	Cattle	ehxCABD	27
GCA_003765485	A	USA	119	O165:H25	Plasmid	Unknown	ehxCABD	26
GCA_000701125	B1	USA	16	O111:H8	Not determined	Unknown	ehxCABD	27
GCA_000622595	B1	USA	21	O26:H11	Plasmid	Unknown	ehxCABD	27
GCA_001607995	A	Netherlands	342	O5:NA	Plasmid	Human	ehxCABD	26
GCA_005046895	B1	Canada	17	O45:H2	Not determined	Cattle	ehxCABD	15
GCA_002765075	B1	Japan	21	O26:H11	Plasmid	Human	ehxCABD	27
GCA_004160135	E	United Kingdom	11	O157:H7	Plasmid	Human	ehxCABD	20
GCA_002175405	E	USA	11	O157:H7	Not determined	Cattle	ehxCABD	20
GCA_002769355	B1	Japan	21	O26:H11	Plasmid	Human	ehxCABD	27
GCA_005392005	A	Japan	329	O136:H16	Plasmid	Cattle	ehxCABD	23

GCA_002476125	E	USA	11	O157:H7	Plasmid	Unknown	ehxCABD	20
GCA_004256645	A	United Kingdom	301	O80:H2	Not determined	Human	ehxCABD	19
GCA_005397845	B1	USA	300	O108:H25	Plasmid	Cattle	ehxCABD	14
GCA_003359815	E	Canada	11	?:H7	Plasmid	Human	ehxCABD	20
GCA_002764715	B1	Japan	21	O26:H11	Plasmid	Human	ehxCABD	27
GCA_003879815	E	United Kingdom	11	O157:H7	Plasmid	Human	ehxCABD	20
GCA_004232775	B1	USA	21	O69:H11	Plasmid	Unknown	ehxCABD	27
GCA_003757305	E	USA	11	O157:H7	Plasmid	Unknown	ehxCABD	20
GCA_005396825	B1	Japan	17	O15:H2	Not determined	Cattle	ehxCABD	15
GCA_005038955	B1	Canada	302	O139:H19	Plasmid	Cattle	ehxCABD	25
GCA_002473745	E	USA	11	O157:H7	Plasmid	Unknown	ehxCABD	20
GCA_005393415	B1	Japan	19	?:H28	Plasmid	Cattle	ehxCABD	25
GCA_003864695	B1	Japan	655	O121:H19	Plasmid	Human	ehxCABD	18
GCA_005041175	E	Canada	11	O157:H7	Plasmid	Unknown	ehxCABD	20
GCA_003362035	E	Canada	11	O157:H7	Plasmid	Unknown	ehxCABD	20
GCA_004270045	E	United Kingdom	11	O157:H7	Plasmid	Human	ehxCABD	20
GCA_000695155	D	Germany	32	O145:H28	Plasmid	Unknown	ehxCABD	8
GCA_001012315	B1	USA	297	O130:H11	Plasmid	Human	ehxCABD	25
GCA_900448875	B1	Unknown	21	O26:H11	Plasmid	Unknown	ehxCABD	27
GCA_005043025	B1	Canada	17	O103:H2	Not determined	Unknown	ehxCABD	15
GCA_004163835	E	United Kingdom	11	O157:H7	Plasmid	Human	ehxCABD	20
GCA_004767125	B1	USA	21	O26:H11	Plasmid	Pig	ehxCABD	27
GCA_003361575	E	Canada	11	O157:H7	Plasmid	Cattle	ehxCABD	20
GCA_003360055	E	Canada	11	?:H7	Plasmid	Human	ehxCABD	20
GCA_004264285	B1	United Kingdom	442	O146:H21	Plasmid	Human	ehxCABD	17
GCA_005042375	B1	Canada	679	O163:H19	Plasmid	Unknown	ehxCABD	10
GCA_001606725	B1	Netherlands	17	O103:H2	Not determined	Human	ehxCABD	15
GCA_004255305	E	United Kingdom	11	O157:H7	Plasmid	Unknown	ehxCABD	20
GCA_004232505	A	USA	342	?:NA	Plasmid	Unknown	ehxCABD	26
GCA_001677725	E	Argentina	11	O157:H7	Plasmid	Human	ehxCABD	20
GCA_005041265	A	Canada	342	O5:NA	Plasmid	Unknown	ehxCABD	26
GCA_002134445	E	Canada	11	?:H7	Plasmid	Unknown	ehxCABD	20
GCA_000617405	B1	USA	21	O151.Gp3/O118.Gp3:H16	Plasmid	Unknown	ehxCABD	27
GCA_004274045	E	United Kingdom	11	O157:H7	Plasmid	Human	ehxCABD	20
GCA_003907675	B1	United Kingdom	33	?:H14	Not determined	Human	ehxCABD	12
GCA_004183115	E	United Kingdom	11	O157:H7	Plasmid	Human	ehxCABD	20
GCA_002770375	B1	Japan	21	O26:H11	Plasmid	Human	ehxCABD	27
GCA_004158215	B1	United Kingdom	21	O26:H11	Plasmid	Human	ehxCABD	27
GCA_005042025	B1	Canada	679	O163:H19	Plasmid	Unknown	ehxCABD	10
GCA_003123155	A	France	301	O80:H2	Plasmid	Human	ehxCABD	19
GCA_005039375	B1	Canada	21	O26:H11	Plasmid	Human	ehxCABD	27
GCA_004256945	E	United Kingdom	11	O157:H7	Plasmid	Human	ehxCABD	20
GCA_000616705	E	USA	11	O157:H7	Plasmid	Unknown	ehxCABD	20
GCA_008753445	E	USA	11	O157:H7	Plasmid	Human	ehxCABD	20
GCA_005042305	B1	Canada	679	O163:H19	Plasmid	Unknown	ehxCABD	10
GCA_004766845	E	USA	11	O157:H7	Plasmid	Pig	ehxCABD	20
GCA_004766695	B1	USA	201	?:H19	Not determined	Unknown	ehxCABD	21
GCA_003360915	E	Canada	11	O157:H7	Plasmid	Unknown	ehxCABD	20
GCA_002923575	B1	Japan	21	O26:H11	Plasmid	Human	ehxCABD	27
GCA_002164195	E	USA	11	O157:H7	Plasmid	Unknown	ehxCABD	20
GCA_002768895	B1	Belgium	21	O26:H11	Plasmid	Human	ehxCABD	27
GCA_003361115	E	Canada	11	O157:H7	Plasmid	Unknown	ehxCABD	20
GCA_003360335	E	Canada	11	O157:H7	Plasmid	Human	ehxCABD	20
GCA_003361935	E	Canada	11	O157:H7	Plasmid	Unknown	ehxCABD	20
GCA_005041345	B1	Canada	17	O103:H2	Not determined	Unknown	ehxCABD	15
GCA_004232685	B1	USA	1792	O111:H8	Not determined	Unknown	ehxCABD	27
GCA_003347155	E	Romania	11	O157:H7	Plasmid	Human	ehxCABD	20
GCA_002769695	B1	Japan	21	O26:H11	Plasmid	Human	ehxCABD	27
GCA_003361175	E	Canada	11	O157:H7	Plasmid	Cattle	ehxCABD	20
GCA_003923235	E	United Kingdom	11	O157:H7	Plasmid	Human	ehxCABD	20
GCA_003741235	E	USA	11	O157:H7	Plasmid	Unknown	ehxCABD	20
GCA_002531515	B1	Canada	21	?:H11	Plasmid	Unknown	ehxCABD	27
GCA_001660335	B1	Japan	21	O26:H11	Plasmid	Cattle	ehxCABD	27
GCA_003361995	E	Canada	11	O157:H7	Plasmid	Unknown	ehxCABD	20
GCA_001191045	A	USA	329	O136:H16	Plasmid	Unknown	ehxCABD	23
GCA_003361705	E	Canada	11	O157:H7	Plasmid	Unknown	ehxCABD	20
GCA_002766435	B1	Japan	21	O26:H11	Plasmid	Human	ehxCABD	27
GCA_000181755	E	Unknown	11	O157:H7	Plasmid	Unknown	ehxCABD	20
GCA_005043695	B1	Canada	16	O111:H8	Not determined	Unknown	ehxCABD	27
GCA_003027185	E	Austria	11	O157:H7	Plasmid	Human	ehxCABD	20
GCA_002766215	B1	Japan	21	O26:H11	Plasmid	Human	ehxCABD	27
GCA_008756525	B1	USA	655	O121:H19	Plasmid	Human	ehxCABD	18
GCA_002768675	B1	Japan	21	O26:H11	Plasmid	Human	ehxCABD	27
GCA_004231145	E	USA	11	O157:H7	Plasmid	Unknown	ehxCABD	20
GCA_003907455	B1	United Kingdom	25	O128:H2	Plasmid	Human	ehxCABD	13
GCA_003901175	B1	USA	16	?:H8	Not determined	Unknown	ehxCABD	27
GCA_008755365	E	USA	11	O157:H7	Plasmid	Human	ehxCABD	20
GCA_000618245	E	USA	11	O157:H7	Plasmid	Unknown	ehxCABD	20

GCA_000622575	B1	USA	21	O26:H11	Plasmid	Unknown	ehxCABD	27
GCA_005390525	B1	Japan	2385	?:H19	Plasmid	Cattle	ehxCABD	21
GCA_005045645	B1	USA	2385	?:H19	Plasmid	Unknown	ehxCABD	21
GCA_004233955	E	USA	11	O157:H7	Plasmid	Unknown	ehxCABD	20
GCA_005390545	B1	Japan	223	O113:H21	Plasmid	Cattle	ehxCABD	22
GCA_005042825	B1	Canada	16	O111:H8	Not determined	Unknown	ehxCABD	27
GCA_002764595	B1	Japan	21	O26:H11	Plasmid	Human	ehxCABD	27
GCA_005383925	B1	Japan	135	?:H2	Not determined	Cattle	ehxCABD	15
GCA_001660345	B1	Japan	350	?:H21	Plasmid	Cattle	ehxCABD	17
GCA_005044945	B1	Denmark	397	O22:H8	Plasmid	Unknown	ehxCABD	25
GCA_002734845	B1	USA	21	O26:H11	Not determined	Cattle	ehxCABD	27
GCA_000619265	B1	USA	17	O45:H2	Not determined	Unknown	ehxCABD	15
GCA_000622485	B1	USA	21	O26:H11	Plasmid	Unknown	ehxCABD	27
GCA_000461975	E	Unknown	11	O157:H7	Plasmid	Unknown	ehxCABD	20
GCA_005042885	A	Canada	9021	O177:H25	Plasmid	Unknown	ehxCABD	26
GCA_005042205	B1	Canada	17	O151.Gp3/O118.Gp3:H2	Plasmid	Unknown	ehxCABD	15
GCA_005380865	B1	Japan	655	O121:H19	Plasmid	Human	ehxCABD	18
GCA_002515605	B1	Norway	17	O103:H2	Not determined	Unknown	ehxCABD	15
GCA_004183375	E	United Kingdom	11	O157:H7	Plasmid	Human	ehxCABD	20
GCA_002486565	E	USA	11	O157:H7	Plasmid	Unknown	ehxCABD	20
GCA_004271775	E	United Kingdom	11	O157:H7	Plasmid	Human	ehxCABD	20
GCA_002764355	B1	Japan	21	O26:H11	Plasmid	Cattle	ehxCABD	27
GCA_002770055	B1	Japan	21	O26:H11	Plasmid	Human	ehxCABD	27
GCA_002554675	E	Unknown	11	O157:H7	Plasmid	Human	ehxCABD	20
GCA_001191315	B1	USA	5013	O74:H42	Not determined	Unknown	ehxCABD	25
GCA_002766775	B1	Japan	21	O26:H11	Plasmid	Human	ehxCABD	27
GCA_000622505	B1	USA	21	O26:H11	Plasmid	Unknown	ehxCABD	27
GCA_002134505	E	Canada	11	O157:H7	Plasmid	Unknown	ehxCABD	20
GCA_002319155	B1	USA	679	O163:H19	Plasmid	Unknown	ehxCABD	10
GCA_004157635	B1	United Kingdom	33	O91:H14	Not determined	Human	ehxCABD	12
GCA_004183055	B1	United Kingdom	33	O91:H14	Not determined	Human	ehxCABD	12
GCA_004262065	E	United Kingdom	11	O157:H7	Plasmid	Human	ehxCABD	20
GCA_004163875	B1	United Kingdom	300	O182:H25	Not determined	Human	ehxCABD	14
GCA_002810665	B1	USA	29	O26:H11	Plasmid	Cattle	ehxCABD	27
GCA_003027235	E	Austria	11	?:H7	Plasmid	Human	ehxCABD	20
GCA_003360635	E	Canada	11	O157:H7	Plasmid	Human	ehxCABD	20
GCA_004183855	B1	United Kingdom	135	O103:H2	Plasmid	Human	ehxCABD	15
GCA_001262855	B1	USA	111	?:H49	Plasmid	Unknown	ehxCABD	25
GCA_003113055	B1	Japan	29	O26:H11	Plasmid	Human	ehxCABD	27
GCA_002176385	E	USA	11	O157:H7	Not determined	Cattle	ehxCABD	20
GCA_000616245	B1	USA	655	O121:H19	Plasmid	Unknown	ehxCABD	18
GCA_003361455	E	Canada	11	O157:H7	Plasmid	Unknown	ehxCABD	20
GCA_000632615	B1	USA	16	O111:H8	Not determined	Unknown	ehxCABD	27
GCA_002735065	B1	France	21	O26:H11	Plasmid	Human	ehxCABD	27
GCA_003916975	E	USA	11	O157:H7	Plasmid	Unknown	ehxCABD	20
GCA_002806795	E	USA	11	O157:H7	Plasmid	Human	ehxCABD	20
GCA_000616225	B1	USA	655	O121:H19	Plasmid	Unknown	ehxCABD	18
GCA_002458345	E	Unknown	11	O157:H7	Plasmid	Human	ehxCABD	20
GCA_001607175	B1	Netherlands	17	O103:H2	Not determined	Human	ehxCABD	15
GCA_004261405	A	United Kingdom	10	O38:H26	Plasmid	Human	ehxCABD	24
GCA_008634205	D	Japan	32	O145:H28	Plasmid	Human	ehxCABD	8
GCA_004231425	E	USA	11	O157:H7	Plasmid	Unknown	ehxCABD	20
GCA_002767505	B1	Japan	21	O26:H11	Plasmid	Human	ehxCABD	27
GCA_002765515	B1	Japan	21	O26:H11	Plasmid	Human	ehxCABD	27
GCA_005397465	B1	Japan	443	O178.Gp11/O153.O178.Gp11:H19	Plasmid	Cattle	ehxCABD	25
GCA_004162475	E	United Kingdom	11	O157:H7	Plasmid	Human	ehxCABD	20
GCA_008633645	D	Belgium	32	O145:H28	Plasmid	Human	ehxCABD	8
GCA_003880175	E	United Kingdom	11	O157:H7	Plasmid	Human	ehxCABD	20
GCA_004232005	E	USA	11	O157:H7	Plasmid	Unknown	ehxCABD	20
GCA_000617465	B1	USA	16	O111:H8	Not determined	Unknown	ehxCABD	27
GCA_004175535	B1	United Kingdom	21	O26:H11	Plasmid	Human	ehxCABD	27
GCA_002768195	B1	Japan	21	O26:H11	Plasmid	Human	ehxCABD	27
GCA_005039195	B1	Canada	21	O26:H11	Plasmid	Cattle	ehxCABD	27
GCA_004274765	E	United Kingdom	11	O157:H7	Plasmid	Human	ehxCABD	20
GCA_000617705	E	USA	11	O157:H7	Plasmid	Unknown	ehxCABD	20
GCA_001607635	B1	Netherlands	33	O91:H14	Not determined	Human	ehxCABD	12
GCA_001309905	E	Canada	11	O157:H7	Plasmid	Unknown	ehxCABD	20
GCA_003882215	E	United Kingdom	11	O157:H7	Plasmid	Human	ehxCABD	20
GCA_000965665	B1	Norway	29	O26:H11	Plasmid	Human	ehxCABD	27
GCA_000462305	E	Unknown	11	O157:H7	Plasmid	Unknown	ehxCABD	20
GCA_000316765	E	Unknown	11	O157:H7	Plasmid	Unknown	ehxCABD	20
GCA_001677535	E	Argentina	11	O157:H7	Plasmid	Human	ehxCABD	20
GCA_001608005	B1	Netherlands	21	O69:H11	Plasmid	Human	ehxCABD	27
GCA_004258765	E	United Kingdom	11	O157:H7	Plasmid	Human	ehxCABD	20
GCA_003740945	A	USA	342	O5:NA	Not determined	Unknown	ehxCABD	26
GCA_002765175	B1	Japan	21	O26:H11	Plasmid	Human	ehxCABD	27
GCA_004165155	E	United Kingdom	11	O157:H7	Plasmid	Human	ehxCABD	20
GCA_000316745	E	Unknown	11	O157:H7	Plasmid	Unknown	ehxCABD	20

GCA_004766795	B1	Unknown	16	O111:H8	Not determined	Pig	ehxCABD	27
GCA_007649015	B1	Brazil	29	O26:H11	Plasmid	Human	ehxCABD	27
GCA_004181115	B1	United Kingdom	442	?:H21	Plasmid	Human	ehxCABD	17
GCA_003759245	D	USA	32	?:H28	Plasmid	Unknown	ehxCABD	8
GCA_003787305	B1	USA	17	O45:H2	Plasmid	Unknown	ehxCABD	15
GCA_001616825	A	China	2	?:H9	Not determined	Sheep	ehxCABD	24
GCA_005045225	B1	USA	672	O104:H19	Plasmid	Unknown	ehxCABD	25
GCA_005040425	B1	Canada	33	O91:H14	Plasmid	Unknown	ehxCABD	12
GCA_003782205	B1	USA	481	O26:H11	Plasmid	Unknown	ehxCABD	27
GCA_003900275	B1	USA	655	?:H19	Not determined	Unknown	ehxCABD	18
GCA_002923895	B1	Japan	21	O26:H11	Plasmid	Human	ehxCABD	27
GCA_001281925	E	Netherlands	11	O157:H7	Plasmid	Human	ehxCABD	20
GCA_008635145	D	Japan	32	?:H28	Plasmid	Human	ehxCABD	8
GCA_008633665	D	Belgium	32	O145:H28	Plasmid	Human	ehxCABD	8
GCA_002460335	E	USA	11	O157:H7	Not determined	Human	ehxCABD	20
GCA_000614035	B1	USA	16	O111:H8	Not determined	Unknown	ehxCABD	27
GCA_003591355	A	Czech Republic	6126	O10:H25	Not determined	Cattle	ehxCABD	26
GCA_003882255	B1	United Kingdom	25	O128:H2	Plasmid	Human	ehxCABD	13
GCA_003902695	E	USA	11	O157:H7	Plasmid	Unknown	ehxCABD	20
GCA_003920215	B1	USA	17	O103:H2	Not determined	Unknown	ehxCABD	15
GCA_004164475	A	United Kingdom	10	?:H4	Not determined	Human	ehxCABD	24
GCA_004796695	D	USA	32	?:H28	Not determined	Cattle	ehxCABD	8
GCA_003770085	B1	USA	21	?:H11	Plasmid	Unknown	ehxCABD	27
GCA_003899855	E	United Kingdom	11	O157:H7	Plasmid	Human	ehxCABD	20
GCA_002769835	B1	Japan	21	O26:H11	Plasmid	Human	ehxCABD	27
GCA_003756385	E	USA	11	?:H7	Plasmid	Unknown	ehxCABD	20
GCA_002735205	B1	Japan	21	O26:H11	Plasmid	Cattle	ehxCABD	27
GCA_001607495	B1	Netherlands	25	O128:H2	Plasmid	Human	ehxCABD	13
GCA_001615175	B1	China	13	O75:H8	Not determined	Sheep	ehxCABD	25
GCA_005046525	B1	Canada	16	O111:H8	Not determined	Cattle	ehxCABD	27
GCA_002473685	E	USA	11	O157:H7	Plasmid	Unknown	ehxCABD	20
GCA_000618305	E	USA	11	O157:H7	Plasmid	Unknown	ehxCABD	20
GCA_001677565	E	Argentina	11	O157:H7	Plasmid	Human	ehxCABD	20
GCA_003760195	B1	USA	17	O103:H2	Not determined	Unknown	ehxCABD	15
GCA_005380965	B1	Japan	655	O121:H19	Plasmid	Human	ehxCABD	18
GCA_000935075	E	Canada	11	O157:H7	Plasmid	Human	ehxCABD	20
GCA_003906675	E	United Kingdom	11	O157:H7	Plasmid	Human	ehxCABD	20
GCA_003922035	B1	United Kingdom	21	O26:H11	Plasmid	Human	ehxCABD	27
GCA_004273585	E	United Kingdom	11	O157:H7	Plasmid	Human	ehxCABD	20
GCA_004161655	E	United Kingdom	11	?:H7	Plasmid	Human	ehxCABD	20
GCA_002924045	B1	Belgium	21	O26:H11	Plasmid	Human	ehxCABD	27
GCA_002769955	B1	Japan	21	O26:H11	Plasmid	Human	ehxCABD	27
GCA_004258025	B1	United Kingdom	17	O103:H2	Plasmid	Human	ehxCABD	15
GCA_004231505	B1	USA	17	O103:H2	Not determined	Unknown	ehxCABD	15
GCA_003293935	B1	Canada	16	O111:H8	Not determined	Human	ehxCABD	27
GCA_005394785	A	Japan	6126	O10:H25	Not determined	Cattle	ehxCABD	26
GCA_005045115	B1	Canada	306	O98:H21	Plasmid	Human	ehxCABD	14
GCA_002175305	E	USA	11	O157:H7	Not determined	Cattle	ehxCABD	20
GCA_002475895	E	USA	11	O157:H7	Plasmid	Unknown	ehxCABD	20
GCA_004182795	E	United Kingdom	11	O157:H7	Plasmid	Human	ehxCABD	20
GCA_002015535	B1	USA	17	O103:H2	Not determined	Cattle	ehxCABD	15
GCA_003887215	E	United Kingdom	11	O157:H7	Plasmid	Human	ehxCABD	20
GCA_002768855	B1	Belgium	21	O26:H11	Plasmid	Human	ehxCABD	27
GCA_003760045	B1	USA	17	O123.O186.Gp5/O123.Gp5:H2	Not determined	Unknown	ehxCABD	15
GCA_000619705	B1	USA	21	O69:H11	Plasmid	Unknown	ehxCABD	27
GCA_003918885	B1	USA	21	?:H11	Plasmid	Unknown	ehxCABD	27
GCA_002475675	E	USA	11	O157:H7	Plasmid	Unknown	ehxCABD	20
GCA_005046505	B1	Switzerland	655	O121:H19	Plasmid	Human	ehxCABD	18
GCA_005038595	B1	Canada	2385	?:H19	Plasmid	Cattle	ehxCABD	21
GCA_000614405	E	USA	11	O157:H7	Plasmid	Unknown	ehxCABD	20
GCA_004231665	B1	USA	17	O103:H2	Not determined	Unknown	ehxCABD	15
GCA_008755395	E	USA	11	O157:H7	Plasmid	Human	ehxCABD	20
GCA_003757625	B1	USA	17	O103:H2	Not determined	Unknown	ehxCABD	15
GCA_005041375	E	Canada	11	?:H7	Plasmid	Unknown	ehxCABD	20
GCA_000234255	B1	Canada	350	O91:H21	Plasmid	Human	ehxCABD	17
GCA_003293985	B1	USA	16	O111:H8	Not determined	Human	ehxCABD	27
GCA_003736485	E	USA	11	O157:H7	Plasmid	Unknown	ehxCABD	20
GCA_002509765	E	USA	11	O157:H7	Plasmid	Unknown	ehxCABD	20
GCA_004234025	B1	USA	21	?:H11	Plasmid	Unknown	ehxCABD	27
GCA_002810845	B1	USA	29	O26:H11	Plasmid	Cattle	ehxCABD	27
GCA_003741045	B1	USA	21	?:H11	Plasmid	Unknown	ehxCABD	27
GCA_003362415	E	Canada	11	O157:H7	Plasmid	Human	ehxCABD	20
GCA_003113075	B1	Japan	29	?:H11	Plasmid	Human	ehxCABD	27
GCA_003915275	E	USA	11	O157:H7	Plasmid	Unknown	ehxCABD	20
GCA_003921555	E	USA	11	O157:H7	Plasmid	Unknown	ehxCABD	20
GCA_000617025	B1	USA	16	O111:H8	Not determined	Unknown	ehxCABD	27
GCA_002474225	E	USA	11	O157:H7	Plasmid	Unknown	ehxCABD	20
GCA_002915115	B1	New Zealand	8892	O121:H19	Plasmid	Wild animals	ehxCABD	18

GCA_003881175	E	United Kingdom	11	O157:H7	Plasmid	Human	ehxCABD	20
GCA_002767335	B1	Japan	21	O26:H11	Plasmid	Human	ehxCABD	27
GCA_001571495	B1	USA	21	O26:H11	Not determined	Cattle	ehxCABD	27
GCA_001607015	B1	Netherlands	21	O26:H11	Plasmid	Human	ehxCABD	27
GCA_004162315	E	United Kingdom	11	O157:H7	Plasmid	Human	ehxCABD	20
GCA_005044935	B1	Canada	21	O71:H11	Not determined	Human	ehxCABD	27
GCA_004232365	B1	USA	17	O151.Gp3/O118.Gp3:H2	Plasmid	Unknown	ehxCABD	15
GCA_003360985	E	Canada	11	O157:H7	Plasmid	Unknown	ehxCABD	20
GCA_003362155	E	Canada	11	O157:H7	Plasmid	Cattle	ehxCABD	20
GCA_003360215	E	Canada	11	O157:H7	Plasmid	Human	ehxCABD	20
GCA_003360595	E	Canada	11	O157:H7	Plasmid	Human	ehxCABD	20
GCA_003304035	B1	China	88	O109:H40	Plasmid	Human	ehxCABD	25
GCA_003864675	B1	Japan	655	O121:H19	Plasmid	Human	ehxCABD	18
GCA_001660255	B1	Japan	223	O113:H21	Plasmid	Cattle	ehxCABD	22
GCA_000616305	B1	USA	16	O111:H8	Not determined	Unknown	ehxCABD	27
GCA_003418635	E	Unknown	11	O157:H7	Plasmid	Unknown	ehxCABD	20
GCA_005390425	D	Japan	32	?:H28	Plasmid	Cattle	ehxCABD	8
GCA_000335175	E	Unknown	11	O157:H7	Plasmid	Unknown	ehxCABD	20
GCA_000632635	B1	USA	16	O111:H8	Not determined	Unknown	ehxCABD	27
GCA_002473775	E	USA	11	O157:H7	Plasmid	Unknown	ehxCABD	20
GCA_003362135	E	Canada	11	O157:H7	Plasmid	Human	ehxCABD	20
GCA_003113715	B1	Japan	21	O26:H11	Plasmid	Human	ehxCABD	27
GCA_002458885	B1	USA	6175	O146:H21	Not determined	Human	ehxCABD	17
GCA_003764885	E	USA	11	O157:H7	Plasmid	Unknown	ehxCABD	20
GCA_004215555	B1	USA	16	O111:H8	Not determined	Unknown	ehxCABD	27
GCA_003916985	E	United Kingdom	628	O157:H7	Plasmid	Human	ehxCABD	20
GCA_001607975	B1	Netherlands	300	O108:H25	Not determined	Human	ehxCABD	14
GCA_001012335	B1	USA	-	O130:H38	Plasmid	Unknown	ehxCABD	25
GCA_002810885	B1	USA	29	O26:H11	Plasmid	Cattle	ehxCABD	27
GCA_003294015	B1	USA	16	O111:H8	Not determined	Human	ehxCABD	27
GCA_001466815	B1	South Korea	111	?:H49	Plasmid	Human	ehxCABD	25
GCA_003360375	E	Canada	11	O157:H7	Plasmid	Human	ehxCABD	20
GCA_001608205	B1	Netherlands	17	O103:H2	Not determined	Human	ehxCABD	15
GCA_001011995	B1	USA	16	O111:H8	Not determined	Wild animals	ehxCABD	27
GCA_002486625	E	USA	11	O157:H7	Plasmid	Unknown	ehxCABD	20
GCA_005044895	B1	Canada	21	O151.Gp3/O118.Gp3:H16	Plasmid	Human	ehxCABD	27
GCA_003882335	B1	United Kingdom	336	?:H16	Plasmid	Human	ehxCABD	25
GCA_004165535	E	United Kingdom	11	O157:H7	Plasmid	Human	ehxCABD	20
GCA_005398325	E	USA	11	O157:H7	Plasmid	Cattle	ehxCABD	20
GCA_002923475	B1	USA	21	O26:H11	Plasmid	Human	ehxCABD	27
GCA_002768755	B1	Japan	21	O26:H11	Plasmid	Human	ehxCABD	27
GCA_005042935	B1	Canada	343	O103:H25	Not determined	Cattle	ehxCABD	14
GCA_000462805	E	Unknown	11	O157:H7	Plasmid	Unknown	ehxCABD	20
GCA_008633985	D	Japan	32	O145:H28	Plasmid	Human	ehxCABD	8
GCA_008635295	D	Japan	32	?:H28	Plasmid	Human	ehxCABD	8
GCA_002134175	B1	Canada	655	O121:H19	Plasmid	Unknown	ehxCABD	18
GCA_003768825	B1	USA	17	O123.O186.Gp5/O123.Gp5:H2	Plasmid	Unknown	ehxCABD	15
GCA_003895455	E	USA	11	?:H7	Plasmid	Unknown	ehxCABD	20
GCA_003884535	E	United Kingdom	11	?:H7	Plasmid	Human	ehxCABD	20
GCA_008635875	D	Japan	32	?:H28	Plasmid	Human	ehxCABD	8
GCA_004173655	B1	United Kingdom	33	O91:H14	Not determined	Human	ehxCABD	12
GCA_008633605	D	Japan	32	O145:H28	Plasmid	Human	ehxCABD	8
GCA_001606365	B1	Netherlands	21	O26:H11	Plasmid	Human	ehxCABD	27
GCA_004273525	E	United Kingdom	11	O157:H7	Plasmid	Human	ehxCABD	20
GCA_005397025	B1	Japan	679	O163:H19	Plasmid	Cattle	ehxCABD	10
GCA_003361055	E	Canada	11	O157:H7	Plasmid	Cattle	ehxCABD	20
GCA_005384045	B1	Japan	21	O26:H11	Plasmid	Cattle	ehxCABD	27
GCA_003907775	E	United Kingdom	11	O157:H7	Plasmid	Human	ehxCABD	20
GCA_004181095	B1	United Kingdom	33	?:H14	Not determined	Human	ehxCABD	12
GCA_001607265	A	Netherlands	342	O5:NA	Plasmid	Human	ehxCABD	26
GCA_001950755	D	Japan	32	O145:H28	Not determined	Wild animals	ehxCABD	8
GCA_004174175	E	United Kingdom	11	O157:H7	Plasmid	Human	ehxCABD	20
GCA_000446365	A	Slovenia	342	O177:H25	Not determined	Human	ehxCABD	26
GCA_001606615	B1	Netherlands	415	O76:H19	Not determined	Human	ehxCABD	25
GCA_002133435	E	Canada	11	O157:H7	Plasmid	Unknown	ehxCABD	20
GCA_003914855	E	United Kingdom	11	O157:H7	Plasmid	Human	ehxCABD	20
GCA_000462605	E	Unknown	11	O157:H7	Plasmid	Unknown	ehxCABD	20
GCA_001440735	A	Belgium	342	O5:NA	Plasmid	Human	ehxCABD	26
GCA_004263345	D	United Kingdom	11	O157:H7	Plasmid	Human	ehxCABD	20
GCA_003760785	B1	USA	655	O121:H19	Plasmid	Unknown	ehxCABD	18
GCA_001607205	B1	Netherlands	21	O26:H11	Plasmid	Human	ehxCABD	27
GCA_004234535	E	USA	11	?:H7	Plasmid	Unknown	ehxCABD	20
GCA_002769575	B1	Japan	21	O26:H11	Plasmid	Human	ehxCABD	27
GCA_003361915	E	Canada	11	O157:H7	Plasmid	Unknown	ehxCABD	20
GCA_005380985	B1	Japan	655	O121:H19	Plasmid	Human	ehxCABD	18
GCA_005045125	B1	USA	591	O156:H25	Not determined	Unknown	ehxCABD	14
GCA_005394065	B1	Japan	2385	?:H19	Plasmid	Cattle	ehxCABD	21
GCA_005040345	E	Canada	11	O157:H7	Plasmid	Unknown	ehxCABD	20

GCA_002766035	B1	Japan	21	O26:H11	Plasmid	Human	ehxCABD	27
GCA_001309645	B1	Canada	415	O76:H19	Plasmid	Unknown	ehxCABD	25
GCA_004166095	B1	United Kingdom	33	?:H14	Not determined	Human	ehxCABD	12
GCA_005040835	B1	Canada	679	O163:H19	Plasmid	Unknown	ehxCABD	10
GCA_008755965	B1	USA	16	?:H8	Not determined	Human	ehxCABD	27
GCA_000615965	E	USA	11	O157:H7	Plasmid	Unknown	ehxCABD	20
GCA_003892655	E	United Kingdom	11	O157:H7	Plasmid	Human	ehxCABD	20
GCA_005042795	B1	Canada	21	O26:H11	Plasmid	Unknown	ehxCABD	27
GCA_004163955	E	United Kingdom	11	O157:H7	Plasmid	Human	ehxCABD	20
GCA_003891915	E	United Kingdom	11	O157:H7	Plasmid	Human	ehxCABD	20
GCA_002516265	A	USA	119	O165:H25	Plasmid	Unknown	ehxCABD	26
GCA_000695115	B1	USA	21	O26:H11	Plasmid	Unknown	ehxCABD	27
