# Supplementary material for: Genomic characterization of enterohaemolysin-encoding haemolytic Escherichia coli of animal and human origin
Source: Microb Genom. 2023 Apr 27;9(4):mgen000999. doi: 10.1099/mgen.0.000999 (PMC10210957; doi:10.1099/mgen.0.000999)
Supplement: Supplementary material 3 [file mgen-9-999-s003.pdf]

Plasmid	Strain	percentage_idenity	alignment_length	plasmid_length	contig_length	coverage_of_contig_length	mismatch	gapopen	qstart	qend	sstart	send	evalue	bitscore
NZ_CP008958.1	GCA_000006665	99,998	92077	92076	92077	100	1	1	1	92076	1	92077	0	170000
NZ_CP028599.1	GCA_000181735	99,978	91854	94607	94707	97	2	3	1	91847	2865	94707	0	169500
NZ_CP034800.1	GCA_000181755	99,99	92246	94606	94753	97	0	3	2363	94606	1	92239	0	170300
NZ_CP027343.1	GCA_000215145	99,968	12397	131410	12395	100	1	3	41299	53694	1	12395	0	22868
NZ_CP038413.1	GCA_000234215	100	43762	121214	43762	100	0	0	15337	59098	1	43762	0	80814
NZ_CP031899.1	GCA_000234235	100	59279	160712	59279	100	0	0	69696	128974	1	59279	0	109500
CP042949.1	GCA_000234255	100	19760	118482	19760	100	0	0	59564	79323	1	19760	0	36490
NZ_CP022408.1	GCA_000234275	99,987	15464	81950	15464	100	2	0	51722	67185	1	15464	0	28546
AP019709.1	GCA_000234315	100	22164	86874	22164	100	0	0	16759	38922	22164	1	0	40930
NZ_CP028121.1	GCA_000259385	99,793	35750	109466	35863	100	28	6	25373	61112	150	35863	0	65566
NZ_CP028684.1	GCA_000260475	99,984	31425	92725	32880	96	3	1	19466	50890	31423	1	0	58002
NZ_CP040573.1	GCA_000267045	99,991	70130	94177	73088	96	2	2	1	70130	2963	73088	0	129500
NZ_CP015844.2	GCA_000302715	99,992	39284	95598	39283	100	2	1	1585	40868	39283	1	0	72526
NZ_CP038495.1	GCA_000303955	99,994	34205	92707	34205	100	0	2	52069	86271	1	34205	0	63152
CP043016.1	GCA_000316465	99,964	27597	91446	28155	98	3	1	12567	40156	78	27674	0	50900
NZ_CP044144.1	GCA_000316485	99,991	22483	92690	23097	97	0	2	10528	33008	1	22483	0	41506
NC_013010.1	GCA_000316545	100	29306	94601	30181	97	0	0	1	29306	29306	1	0	54119
NC_017907.1	GCA_000316565	100	30914	92728	31453	98	0	0	1	30914	30914	1	0	57088
NZ_CP038308.1	GCA_000316745	99,971	28049	93167	28507	98	6	2	10632	38678	236	28284	0	51751
NC_013010.1	GCA_000316905	99,99	29094	94601	30306	96	3	0	1	29094	470	29563	0	53710
NZ_CP038320.1	GCA_000334995	100	23579	95338	24102	98	0	0	1	23579	321	23899	0	43543
NZ_CP032794.1	GCA_000335055	99,979	28784	92653	28784	100	6	0	56978	85761	28784	1	0	53121
NC_013010.1	GCA_000335075	99,997	29385	94601	29581	99	1	0	1	29385	29390	6	0	54259
NZ_CP017437.1	GCA_000335115	99,997	38605	92565	38605	100	1	0	15260	53864	38605	1	0	71285
NZ_CP032792.1	GCA_000335135	99,983	42320	90310	43443	97	3	2	8077	50395	42542	226	0	78108
NZ_CP038341.1	GCA_000335155	100	30989	92839	31511	98	0	0	1	30989	30989	1	0	57226
NZ_CP034793.1	GCA_000335175	99,993	29473	95421	30794	96	1	1	4729	34201	29861	390	0	54414
NC_013010.1	GCA_000335255	99,997	29440	94601	29628	99	1	0	1	29440	189	29628	0	54360
NC_013010.1	GCA_000335335	100	29182	94601	29996	97	0	0	1	29182	815	29996	0	53890
NC_017907.1	GCA_000335375	100	30959	92728	31476	98	0	0	1	30959	30959	1	0	57171
NZ_CP028655.1	GCA_000335415	100	38268	92732	38268	100	0	0	12809	51076	38268	1	0	70668
NZ_CP040310.1	GCA_000335435	99,97	43165	93175	43163	100	8	4	7861	51022	1	43163	0	79634
NZ_CP038341.1	GCA_000335455	100	30926	92839	31485	98	0	0	1	30926	30926	1	0	57110
NC_013354.1	GCA_000414155	99,987	14879	75546	15565	96	2	0	1	14879	595	15473	0	27466
NZ_CP006263.1	GCA_000446365	99,966	8830	98066	8830	100	3	0	84080	92909	1	8830	0	16290
NC_017907.1	GCA_000447025	99,997	30748	92728	31316	98	1	0	1	30748	30748	1	0	56776
NZ_CP027389.1	GCA_000447085	99,973	18299	68062	18354	100	5	0	28551	46849	56	18354	0	33765
NZ_CP062161.1	GCA_000461915	99,959	94002	104844	94010	100	10	4	2602	96602	37	94010	0	173300
NZ_CP062161.1	GCA_000461955	99,947	43428	104844	43428	100	17	1	3656	47077	43428	1	0	80064
NZ_CP038341.1	GCA_000461975	100	30888	92839	32031	96	0	0	1	30888	30888	1	0	57040
NZ_CP028701.1	GCA_000462025	99,998	40339	92738	40339	100	1	0	7746	48084	40339	1	0	74487
NZ_CP038341.1	GCA_000462065	100	30944	92839	31488	98	0	0	1	30944	545	31488	0	57143
NZ_CP038341.1	GCA_000462105	100	30967	92839	32058	97	0	0	1	30967	30967	1	0	57186
NZ_CP038341.1	GCA_000462125	100	30932	92839	31975	97	0	0	1	30932	30932	1	0	57121
NZ_CP038314.1	GCA_000462145	99,989	18913	97630	18913	100	2	0	28741	47653	18913	1	0	34915
NZ_CP017250.1	GCA_000462165	99,949	27228	92690	27221	100	7	1	11522	38749	27221	1	0	50196
NZ_CP034805.1	GCA_000462185	99,995	40401	94014	40401	100	1	1	36167	76566	1	40401	0	74594
NZ_CP038343.1	GCA_000462225	99,958	35590	92743	35724	100	7	3	12489	48072	137	35724	0	65632
NZ_CP031914.1	GCA_000462245	100	28217	92754	28525	99	0	0	19618	47834	2	28218	0	52108
NZ_CP032794.1	GCA_000462305	99,964	16709	92653	16898	99	6	0	39660	56368	16709	1	0	30823
CP058232.1	GCA_000462325	99,995	38549	94605	38549	100	2	0	7000	45548	1	38549	0	71176
NZ_CP040317.1	GCA_000462385	99,995	38676	93331	38676	100	2	0	12523	51198	38676	1	0	71411
NZ_CP032796.1	GCA_000462425	99,99	49386	90978	51543	96	3	1	3372	52757	1	49384	0	91170
NZ_CP035546.1	GCA_000462465	99,987	93497	94581	94610	99	3	9	1	93494	1120	94610	0	172600
NZ_CP038323.1	GCA_000462485	99,994	34007	92701	34007	100	2	0	53383	87389	34007	1	0	62789
NZ_CP031914.1	GCA_000462505	100	28217	92754	28453	99	0	0	19618	47834	2	28218	0	52108
NZ_CP041624.1	GCA_000462525	99,989	18939	95081	18940	100	2	0	46667	65605	2	18940	0	34963
NZ_CP012803.1	GCA_000462545	99,968	27721	92739	28241	98	0	1	1	27721	530	28241	0	51133
NZ_CP046526.1	GCA_000462565	99,989	18936	98304	18936	100	2	0	31615	50550	1	18936	0	34958
NZ_CP031914.1	GCA_000462585	100	28211	92754	28412	99	0	0	19624	47834	1	28211	0	52096
NZ_CP032794.1	GCA_000462605	99,714	16785	92653	17561	96	41	7	57730	74510	17302	521	0	30723
NZ_CP031914.1	GCA_000462625	99,982	28474	92754	28946	98	3	1	19361	47834	269	28740	0	52553
NZ_CP031914.1	GCA_000462645	99,986	28474	92754	28946	98	3	1	19361	47834	279	28751	0	52558
NZ_CP017445.1	GCA_000462665	99,993	40347	92726	40604	99	1	2	7817	48161	258	40604	0	74489
NC_017907.1	GCA_000462685	100	30939	92728	31490	98	0	0	1	30939	30939	1	0	57134
NZ_CP062161.1	GCA_000462705	99,97	43352	104844	43352	100	5	1	3660	47003	43352	1	0	79977
NZ_CP038327.1	GCA_000462725	99,994	33930	92704	33930	100	2	0	51890	85819	1	33930	0	62646
NZ_CP062161.1	GCA_000462745	99,945	43415	104844	43660	99	5	1	3615	47010	43660	246	0	80021
NZ_CP012803.1	GCA_000462765	99,968	27983	92739	28512	98	0	1	1	27983	539	28512	0	51616
NZ_CP028608.1	GCA_000462785	100	26870	92745	28212	95	0	0	1	26870	26870	1	0	49620
NZ_CP012803.1	GCA_000462805	99,968	27781	92739	28361	98	0	1	1	27781	590	28361	0	51243
NZ_CP012803.1	GCA_000462825	99,968	27857	92739	28383	98	0	1	1	27857	536	28383	0	51384
NZ_CP031914.1	GCA_000462845	99,972	28474	92754	28935	98	5	1	19361	47834	267	28737	0	52534
NZ_CP038341.1	GCA_000462865	99,997	30978	92839	32037	97	1	0	1	30978	30978	1	0	57201
NZ_CP017437.1	GCA_000462885	99,982	38807	92565	38807	100	7	0	15214	54020	38807	1	0	71625
NC_013010.1	GCA_000462905	99,997	29437	94601	30371	97	1	0	1	29437	935	30371	0	54355
NZ_CP027385.1	GCA_000467695	100	10377	118259	10377	100	0	0	66261	76637	1	10377	0	19163
NZ_CP027339.1	GCA_000473725	99,944	15944	92644	15944	100	1	8	29029	44964	1	15944	0	29386
NZ_CP038341.1	GCA_000477495	100	30892	92839	31979	97	0	0	1	30892	30892	1	0	57047
NZ_CP027385.1	GCA_000478705	99,989	9298	118259	9298	100	1	0	66582	75879	9298	1	0	17165
NZ_CP038406.1	GCA_000496345	99,998	42932	92740	42932	100	0	1	8060	50990	1	42932	0	79274
NZ_CP027389.1	GCA_000506845	100	20269	68062	20269	100	0	0	26612	46880	20269	1	0	37430
NC_013366.1	GCA_000614035	100	13745	77690	13745	100	0	0	52676	66420	13745	1	0	25383
CP051632.1	GCA_000614215	100	15365	81965	15554	99	0	0	20918	36282	15554	190	0	28374
NZ_CP038362.1	GCA_000614305	99,964	27900	95928	28824	97	1	1	1	27900	934	28824	0	51458
NZ_CP038362.1	GCA_000614345	99,964	27918	95928	28727	97	1	1	1	27918	819	28727	0	51491
AP019709.1	GCA_000614565	100	17171	86874	17171	100	0	0	16812	33982	17171	1	0	31709
NZ_CP022408.1	GCA_000614595	99,993	15358	81950	15357	100	0	1	51775	67132	1	15357	0	28354
NZ_CP028597.1	GCA_000614685	99,965	28544	94610	29852	96	1	1	22087	50630	380	28914	0	52647
NZ_CP022408.1	GCA_000614725	100	15358	81950	15358	100	0	0	51775	67132	1	15358	0	28361
NZ_CP022408.1	GCA_000614765	99,994	15469	81950	15468	100	0	1	51775	67243	1	15468	0	28559
NZ_CP027386.1	GCA_000614845	99,966	17704	54452	18010	98	3	1	25917	43617	1	17704	0	32657

NZ_CP022408.1	GCA_000614925	100	15358	81950	15358	100	0	0	51775	67132	1	15358	0	28361
NZ_CP028111.1	GCA_000615175	99,851	12095	83211	12533	97	18	0	17399	29493	12533	439	0	22236
NZ_CP027339.1	GCA_000615225	99,906	15961	92644	15961	100	7	8	29013	44965	15961	1	0	29385
NZ_CP027339.1	GCA_000615415	99,943	15868	92644	16117	98	1	8	29105	44964	15868	1	0	29246
NZ_CP022408.1	GCA_000615455	99,993	15140	81950	15140	100	1	0	51775	66914	1	15140	0	27953
NC_013366.1	GCA_000615575	100	14710	77690	15474	95	0	0	52677	67386	15474	765	0	27165
NZ_CP027385.1	GCA_000615605	100	8484	118259	8484	100	0	0	67082	75565	8484	1	0	15668
NC_013366.1	GCA_000615655	100	14710	77690	14710	100	0	0	52677	67386	14710	1	0	27165
NZ_CP031914.1	GCA_000615745	99,996	28217	92754	28829	98	1	0	19618	47834	359	28575	0	52102
NZ_CP031914.1	GCA_000615785	99,993	28221	92754	28822	98	1	1	19614	47834	342	28561	0	52102
NZ_CP031914.1	GCA_000615865	99,996	28217	92754	28741	98	1	0	19618	47834	341	28557	0	52102
NZ_CP031914.1	GCA_000615965	99,996	28217	92754	28796	98	1	0	19618	47834	28436	220	0	52102
NZ_CP031914.1	GCA_000616005	99,996	28217	92754	28662	98	1	0	19618	47834	28324	108	0	52102
NZ_CP031911.1	GCA_000616135	100	15024	80681	15382	98	0	0	38506	53529	1	15024	0	27745
NZ_CP027385.1	GCA_000616195	100	8484	118259	8484	100	0	0	67082	75565	1	8484	0	15668
NZ_CP022408.1	GCA_000616245	100	15358	81950	15358	100	0	0	51775	67132	1	15358	0	28361
NC_013366.1	GCA_000616265	100	14875	77690	15248	98	0	0	52676	67550	15248	374	0	27470
NZ_CP027385.1	GCA_000616305	100	8484	118259	8484	100	0	0	67082	75565	8484	1	0	15668
NC_013366.1	GCA_000616325	100	14710	77690	14710	100	0	0	52677	67386	1	14710	0	27165
NC_011350.1	GCA_000616645	99,947	17071	94644	17083	100	3	1	21610	38680	17065	1	0	31469
NZ_CP027354.1	GCA_000616665	99,878	13079	57720	13079	100	13	3	19925	33000	13079	1	0	24061
NC_013366.1	GCA_000616685	100	13745	77690	13745	100	0	0	52676	66420	13745	1	0	25383
NZ_CP022408.1	GCA_000616725	100	15462	81950	15462	100	0	0	51775	67236	1	15462	0	28554
NZ_CP022408.1	GCA_000617005	99,993	15358	81950	15358	100	1	0	51775	67132	1	15358	0	28356
NC_013366.1	GCA_000617025	100	14711	77690	14711	100	0	0	52676	67386	1	14711	0	27167
NZ_CP027389.1	GCA_000617125	100	16797	68062	16797	100	0	0	26678	43474	1	16797	0	31019
NZ_CP022408.1	GCA_000617185	99,967	15358	81950	15358	100	5	0	51775	67132	1	15358	0	28334
NC_013366.1	GCA_000617245	100	14710	77690	14710	100	0	0	52677	67386	14710	1	0	27165
NC_013366.1	GCA_000617265	100	13038	77690	13038	100	0	0	52676	65713	1	13038	0	24077
NZ_CP022408.1	GCA_000617285	100	15483	81950	15483	100	0	0	51775	67257	1	15483	0	28592
AP019709.1	GCA_000617345	100	17171	86874	17171	100	0	0	16812	33982	17171	1	0	31709
NZ_CP027339.1	GCA_000617405	99,944	15982	92644	16003	100	1	8	29015	44988	16003	22	0	29457
NZ_CP038362.1	GCA_000617445	99,964	27915	95928	28913	97	1	1	1	27915	27973	68	0	51485
NC_013366.1	GCA_000617465	100	13752	77690	13752	100	0	0	52676	66427	13752	1	0	25396
NZ_CP027385.1	GCA_000617485	100	8503	118259	8503	100	0	0	67063	75565	8503	1	0	15703
NC_013366.1	GCA_000617525	100	14711	77690	15197	97	0	0	52676	67386	1	14711	0	27167
NC_013366.1	GCA_000617545	100	13037	77690	13037	100	0	0	52677	65713	13037	1	0	24075
NZ_CP022408.1	GCA_000617565	100	15358	81950	15358	100	0	0	51775	67132	1	15358	0	28361
NZ_CP027326.1	GCA_000617585	99,934	16760	74671	16750	100	0	2	6480	23238	1	16750	0	30878
NZ_CP022408.1	GCA_000617605	99,922	15358	81950	15358	100	12	0	51775	67132	15358	1	0	28295
NC_013366.1	GCA_000617625	100	13751	77690	13751	100	0	0	52677	66427	13751	1	0	25394
NZ_CP022408.1	GCA_000617645	99,993	15140	81950	15140	100	1	0	51775	66914	15140	1	0	27953
NZ_CP028597.1	GCA_000617725	100	28488	94610	28906	99	0	0	22087	50574	419	28906	0	52608
AP019709.1	GCA_000617765	100	17171	86874	17171	100	0	0	16812	33982	1	17171	0	31709
NZ_CP027385.1	GCA_000617805	100	8549	118259	8549	100	0	0	67017	75565	8549	1	0	15788
NZ_CP038314.1	GCA_000617965	99,944	16174	97630	16174	100	2	1	30766	46932	1	16174	0	29811
NZ_CP028597.1	GCA_000618065	100	28487	94610	28871	99	0	0	22087	50573	385	28871	0	52606
NZ_CP031914.1	GCA_000618105	99,982	28474	92754	28742	99	3	1	19361	47834	21	28492	0	52553
NZ_CP038362.1	GCA_000618145	99,964	27908	95928	28847	97	1	1	1	27908	949	28847	0	51472
NZ_CP022408.1	GCA_000618165	99,98	15360	81950	15360	100	3	0	51774	67133	1	15360	0	28349
NZ_CP022408.1	GCA_000618705	100	15459	81950	15459	100	0	0	51775	67233	1	15459	0	28548
NZ_CP022408.1	GCA_000618725	100	15459	81950	15459	100	0	0	51775	67233	1	15459	0	28548
NZ_CP017445.1	GCA_000618885	99,952	39397	92726	39392	100	14	1	8504	47900	1	39392	0	72642
NZ_CP017445.1	GCA_000618925	99,98	40015	92726	40010	100	3	1	8058	48072	1	40010	0	73844
NZ_CP038362.1	GCA_000618945	99,964	27917	95928	28730	97	1	1	1	27917	27908	1	0	51489
NC_013366.1	GCA_000619005	100	13745	77690	13745	100	0	0	52676	66420	13745	1	0	25383
NZ_CP031907.1	GCA_000619065	99,997	36055	121036	36055	100	0	1	39004	75057	36055	1	0	66574
NZ_CP022408.1	GCA_000619085	99,993	15358	81950	15358	100	1	0	51775	67132	15358	1	0	28356
NZ_CP022408.1	GCA_000619125	100	15358	81950	15358	100	0	0	51775	67132	1	15358	0	28361
NZ_CP022408.1	GCA_000619205	99,993	15140	81950	15140	100	1	0	51775	66914	1	15140	0	27953
NC_013366.1	GCA_000619225	100	13288	77690	13760	97	0	0	52676	65963	13760	473	0	24539
NZ_CP051657.1	GCA_000619265	100	16599	74390	16599	100	0	0	19328	35926	1	16599	0	30653
AP019709.1	GCA_000619285	99,994	17376	86874	17376	100	1	0	16812	34187	17376	1	0	32082
NC_013366.1	GCA_000619465	100	13745	77690	13745	100	0	0	52676	66420	13745	1	0	25383
NC_013366.1	GCA_000619545	100	13041	77690	13058	100	0	0	52677	65717	1	13041	0	24083
NC_013366.1	GCA_000619565	100	13288	77690	13753	97	0	0	52676	65963	1	13288	0	24539
NC_013366.1	GCA_000619585	100	13038	77690	13038	100	0	0	52676	65713	1	13038	0	24077
NC_013366.1	GCA_000619625	100	13038	77690	13038	100	0	0	52676	65713	1	13038	0	24077
NC_013366.1	GCA_000619645	100	13745	77690	13745	100	0	0	52676	66420	13745	1	0	25383
NZ_CP027354.1	GCA_000619705	99,898	15703	57720	15696	100	2	9	17510	33205	1	15696	0	28897
NZ_CP022408.1	GCA_000619745	100	15459	81950	15459	100	0	0	51775	67233	15459	1	0	28548
NZ_CP024480.1	GCA_000619765	100	33431	77062	33431	100	0	0	24026	57456	33431	1	0	61736
AP019709.1	GCA_000622465	99,994	17376	86874	17376	100	1	0	16812	34187	1	17376	0	32082
NZ_CP027386.1	GCA_000622485	99,972	17677	54452	17677	100	2	1	25917	43590	17677	1	0	32612
NZ_CP027386.1	GCA_000622505	99,966	17704	54452	18013	98	3	1	25917	43617	1	17704	0	32657
AP019709.1	GCA_000622535	99,96	17385	86874	17385	100	0	2	16810	34187	1	17385	0	32058
NZ_CP027339.1	GCA_000622555	99,937	15957	92644	15957	100	2	8	29017	44965	1	15957	0	29405
AP019709.1	GCA_000622575	99,954	17376	86874	17369	100	1	4	16812	34187	1	17369	0	32036
NZ_CP027386.1	GCA_000622595	99,955	17704	54452	18012	98	5	1	25917	43617	1	17704	0	32646
AP019709.1	GCA_000622635	99,994	17376	86874	17376	100	1	0	16812	34187	1	17376	0	32082
AP019709.1	GCA_000622675	99,994	17376	86874	17376	100	1	0	16812	34187	17376	1	0	32082
NZ_CP037942.1	GCA_000622755	99,991	11691	157534	11808	99	1	0	40416	52106	1	11691	0	21584
AP019709.1	GCA_000622855	99,994	17376	86874	17376	100	1	0	16812	34187	1	17376	0	32082
NZ_CP037942.1	GCA_000622875	100	11545	157534	11545	100	0	0	61649	73193	11545	1	0	21320
AP019709.1	GCA_000632575	99,988	17378	86874	17378	100	2	0	16811	34188	17378	1	0	32081
NC_013366.1	GCA_000632595	100	14784	77690	14784	100	0	0	52603	67386	1	14784	0	27301
NC_013366.1	GCA_000632615	100	13038	77690	13038	100	0	0	52676	65713	13038	1	0	24077
NC_013366.1	GCA_000632635	100	13038	77690	13038	100	0	0	52676	65713	13038	1	0	24077
NC_013366.1	GCA_000632675	100	13288	77690	13768	97	0	0	52676	65963	13768	481	0	24539
NZ_CP024291.1	GCA_000647455	99,934	16638	111697	16638	100	8	1	56523	73157	1	16638	0	30661
AP019704.1	GCA_000695115	99,991	22253	92337	22253	100	2	0	16651	38903	1	22253	0	41083
NZ_CP024480.1	GCA_000695135	100	36791	77062	36971	100	0	0	20709	57499	181	36971	0	67941
AP019709.1	GCA_000695155	100	22340	86874	22340	100	0	0	16770	39109	1	22340	0	41255

NZ_CP006028.1	GCA_000695175	100	22348	87120	22348	100	0	0	47137	69484	1	22348	0	41270
NZ_CP027385.1	GCA_000701125	100	8483	118259	8483	100	0	0	67084	75566	1	8483	0	15666
NZ_CP012501.1	GCA_000797715	100	25964	242187	25964	100	0	0	95078	121041	1	25964	0	47947
NZ_CP038406.1	GCA_000935075	99,998	43439	92740	43439	100	0	1	7587	51024	43439	1	0	80210
NZ_CP016626.1	GCA_000948765	100	32269	95598	32269	100	0	0	18026	50294	32269	1	0	59590
NZ_CP031923.1	GCA_000948815	100	19709	95298	19709	100	0	0	64172	83880	19709	1	0	36396
NZ_CP023674.1	GCA_000948825	99,762	11324	87524	11484	99	16	2	70821	82134	11484	162	0	20751
NZ_CP022408.1	GCA_000948875	99,994	15798	81950	15798	100	1	0	51542	67339	1	15798	0	29168
NZ_CP024480.1	GCA_000965545	99,957	36783	77062	37126	99	14	1	20681	57463	244	37024	0	67835
NC_013728.1	GCA_000965565	99,984	19144	111481	19270	99	0	2	87034	106177	19270	130	0	35333
NZ_CP006263.1	GCA_000965655	99,955	17701	98066	17971	98	8	0	50939	68639	17701	1	0	32644
NZ_CP027545.1	GCA_000965665	99,893	21485	101089	21738	99	19	4	17710	39191	128	21611	0	39545
NZ_CP027545.1	GCA_000965705	99,887	21272	101089	21398	99	20	4	17710	38978	128	21398	0	39146
NC_013366.1	GCA_001011995	100	13150	77690	13150	100	0	0	52564	65713	13150	1	0	24284
NZ_CP022408.1	GCA_001012015	100	15265	81950	15265	100	0	0	51649	66913	15265	1	0	28190
NZ_CP035771.1	GCA_001012075	99,973	22255	88752	22255	100	5	1	15508	37761	1	22255	0	41063
NZ_CP061760.1	GCA_001012095	99,971	41318	172576	41318	100	12	0	83420	124737	1	41318	0	76234
NZ_CP009107.1	GCA_001012175	99,824	66374	161447	66326	100	65	18	42581	108950	1	66326	0	121900
NC_013366.1	GCA_001012195	100	13152	77690	13152	100	0	0	52562	65713	13152	1	0	24288
NC_013366.1	GCA_001012235	100	13035	77690	13035	100	0	0	52678	65712	1	13035	0	24072
NC_013366.1	GCA_001012255	100	13024	77690	13119	99	0	0	52689	65712	96	13119	0	24051
NC_013366.1	GCA_001012265	99,992	13210	77690	13210	100	1	0	52503	65712	1	13210	0	24389
NC_013366.1	GCA_001012275	100	13149	77690	13149	100	0	0	52564	65712	1	13149	0	24282
NZ_CP027451.1	GCA_001012315	99,946	53321	173649	53321	100	3	3	43349	96643	1	53321	0	98281
NZ_CP027370.1	GCA_001012335	99,975	31876	176149	31875	100	3	5	30876	62747	1	31875	0	58815
NZ_CP031899.1	GCA_001012345	99,965	42715	160712	42715	100	15	0	86894	129608	1	42715	0	78797
NZ_CP027460.1	GCA_001012395	100	16159	74247	16159	100	0	0	52383	68541	1	16159	0	29841
NZ_CP027389.1	GCA_001012405	100	16846	68062	16846	100	0	0	26628	43473	1	16846	0	31109
NZ_CP031909.1	GCA_001012445	100	33560	73224	33560	100	0	0	15366	48925	1	33560	0	61974
NC_007365.1	GCA_001012475	99,324	17604	165548	17637	100	100	6	103420	121022	22	17607	0	31831
NZ_CP042296.1	GCA_001012495	99,995	21629	167256	21629	100	1	0	66966	88594	1	21629	0	39936
NZ_CP042296.1	GCA_001012505	99,977	21599	167256	21599	100	5	0	66996	88594	1	21599	0	39859
NZ_CP012501.1	GCA_001012545	99,969	26122	242187	26223	100	4	3	94920	121041	26118	1	0	48191
NZ_CP045828.1	GCA_001012635	99,954	8624	100778	8624	100	4	0	57352	65975	8624	1	0	15904
NZ_AP018805.1	GCA_001191025	99,985	13020	78434	13107	99	1	1	10332	23350	13107	88	0	24031
NZ_CP012501.1	GCA_001191045	100	25964	242187	25964	100	0	0	95078	121041	25964	1	0	47947
AP019709.1	GCA_001191125	100	22219	86874	22219	100	0	0	16813	39031	22219	1	0	41031
NZ_CP028382.1	GCA_001191135	99,986	36731	160675	36731	100	1	1	92591	129317	1	36731	0	67799
NZ_CP035771.1	GCA_001191185	99,996	22254	88752	22254	100	1	0	15508	37761	1	22254	0	41090
AP019709.1	GCA_001191195	100	16830	86874	16830	100	0	0	16813	33642	16830	1	0	31080
NC_013366.1	GCA_001191265	100	12719	77690	12719	100	0	0	54668	67386	12719	1	0	23488
NZ_CP023164.1	GCA_001191275	99,881	18562	122641	18580	100	2	20	53502	72043	18580	19	0	34138
NZ_CP027343.1	GCA_001191295	99,449	10533	131410	10547	100	54	2	53775	64306	1	10530	0	19126
NZ_CP027343.1	GCA_001191315	99,975	12131	131410	12132	100	2	1	62356	74485	2	12132	0	22384
NZ_CP027585.1	GCA_001191345	99,989	18561	160576	18561	100	2	0	120624	139184	18561	1	0	34265
NZ_CP027339.1	GCA_001191395	99,781	15991	92644	15989	100	19	15	28988	44964	15989	1	0	29322
NZ_CP027339.1	GCA_001191435	99,918	15848	92644	15848	100	5	8	29124	44963	1	15848	0	29187
CP027581.1	GCA_001191455	99,236	16223	118822	16246	100	87	11	37821	54018	18	16228	0	29237
NZ_CP022408.1	GCA_001191505	100	15138	81950	15138	100	0	0	51776	66913	1	15138	0	27955
NZ_CP035771.1	GCA_001191525	100	23983	88752	23983	100	0	0	15393	39375	1	23983	0	44289
NZ_CP023164.1	GCA_001262785	99,872	22692	122641	22692	100	2	26	58198	80862	22692	1	0	41718
NZ_CP027343.1	GCA_001262805	99,993	29699	131410	29699	100	0	2	40573	70269	1	29699	0	54831
CP027581.1	GCA_001262855	99,973	25598	118822	25598	100	7	0	33605	59202	25598	1	0	47232
NZ_CP027372.1	GCA_001262895	99,978	77418	175427	77418	100	16	1	82102	159518	77418	1	0	142900
NZ_CP027372.1	GCA_001262935	99,963	45730	175427	45730	100	16	1	114160	159888	45730	1	0	84352
NZ_CP027370.1	GCA_001262965	99,609	24031	176149	24030	100	88	6	123463	147488	1	24030	0	43851
NZ_CP027583.1	GCA_001263035	99,991	22282	88339	22282	100	2	0	42246	64527	1	22282	0	41137
NZ_CP006263.1	GCA_001266015	99,951	12204	98066	12366	99	6	0	35689	47892	72	12275	0	22504
NZ_CP006263.1	GCA_001266095	99,969	12724	98066	12821	99	4	0	52173	64896	12724	1	0	23475
NZ_CP028653.1	GCA_001281725	99,993	42721	92739	42768	100	1	2	8050	50770	1	42719	0	78873
NZ_CP040317.1	GCA_001281795	99,988	42884	93331	42884	100	4	1	7993	50875	1	42884	0	79163
NZ_CP015844.2	GCA_001281815	99,987	38390	95598	38572	100	3	2	2063	40450	1	38390	0	70864
NZ_CP038401.1	GCA_001281845	99,984	31157	92513	31270	100	3	2	32233	63387	31157	1	0	57507
NZ_CP028653.1	GCA_001281855	99,986	43007	92739	43006	100	5	1	7993	50999	43006	1	0	79384
NZ_CP018244.1	GCA_001281885	99,997	30719	92522	31461	98	1	0	1	30719	30719	1	0	56722
NZ_CP032792.1	GCA_001281905	99,887	51382	90310	52945	97	14	4	18702	70049	27	51398	0	94523
NZ_CP018244.1	GCA_001281925	99,997	30719	92522	31462	98	1	0	1	30719	744	31462	0	56722
NZ_CP028612.1	GCA_001281985	99,99	40516	92725	40536	100	3	1	10384	50898	21	40536	0	74795
NZ_CP038413.1	GCA_001282025	100	43676	121214	43676	100	0	0	15380	59055	43676	1	0	80655
NZ_CP018242.1	GCA_001282065	99,998	41041	92495	41041	100	1	0	31980	73020	1	41041	0	75783
NZ_CP040317.1	GCA_001297975	99,986	44174	93331	44173	100	3	2	7990	52161	1	44173	0	81538
NZ_CP028686.1	GCA_001297985	99,994	31433	92724	31457	100	2	0	19466	50898	25	31457	0	58035
NZ_CP040317.1	GCA_001297995	99,993	42217	93331	42242	100	2	1	8039	50254	1	42217	0	77942
NZ_CP027389.1	GCA_001309635	100	20219	68062	20219	100	0	0	26635	46853	20219	1	0	37338
NZ_CP027545.1	GCA_001309685	99,983	28931	101089	28930	100	0	5	9987	38913	1	28930	0	53393
NZ_CP028686.1	GCA_001309715	99,998	42919	92724	42918	100	0	1	8016	50934	42918	1	0	79250
NC_013366.1	GCA_001309775	100	13120	77690	13120	100	0	0	52635	65754	13120	1	0	24229
NZ_CP022408.1	GCA_001309805	99,994	15856	81950	15856	100	1	0	51318	67173	1	15856	0	29276
NZ_CP027354.1	GCA_001309815	99,94	15126	57720	15126	100	2	7	15806	30924	1	15126	0	27876
NC_013354.1	GCA_001309835	99,974	15303	75546	15944	96	0	1	1	15299	15303	1	0	28234
NZ_CP038341.1	GCA_001309885	100	30753	92839	32323	95	0	0	1	30753	30753	1	0	56791
NC_019041.1	GCA_001309895	100	42224	92077	42919	98	0	0	45189	87412	1	42224	0	77974
NZ_CP017445.1	GCA_001309905	99,986	42913	92726	42910	100	3	1	8017	50929	1	42910	0	79209
NZ_CP034805.1	GCA_001309925	99,998	41825	94014	41885	100	1	0	34455	76279	41825	1	0	77231
NZ_CP028686.1	GCA_001309985	99,995	42919	92724	42918	100	1	1	8016	50934	42918	1	0	79244
NZ_CP038348.1	GCA_001309995	100	31575	89216	31575	100	0	0	21756	53330	1	31575	0	58309
NZ_CP008958.1	GCA_001432175	100	42526	92076	42526	100	0	0	10977	53502	1	42526	0	78531
NZ_CP027588.1	GCA_001440725	99,991	11426	58109	11426	100	1	0	44294	55719	11426	1	0	21095
NZ_CP027588.1	GCA_001440735	99,975	28128	58109	28184	100	6	1	1	28127	57	28184	0	51903
NZ_CP027588.1	GCA_001445675	99,968	28208	58109	29294	96	8	1	1	28207	1087	29294	0	52039
NZ_CP027339.1	GCA_001571485	99,906	15957	92644	15957	100	7	8	29017	44965	15957	1	0	29377
NZ_CP027339.1	GCA_001571495	99,907	16100	92644	16126	100	6	9	28875	44965	27	16126	0	29639
NZ_CP027334.1	GCA_001571805	99,978	13489	181066	13487	100	1	2	12110	25598	1	13487	0	24892

NZ_CP037942.1	GCA_001571915	99,966	11794	157534	11794	100	4	0	61399	73192	11794	1	0	21758
NZ_CP037942.1	GCA_001571925	99,835	12147	157534	12148	100	20	0	93320	105466	1	12147	0	22321
NZ_CP037942.1	GCA_001572455	99,957	11704	157534	11704	100	5	0	40417	52120	11704	1	0	21586
NC_013728.1	GCA_001606365	99,984	19212	111481	19283	100	3	0	86966	106177	1	19212	0	35462
AP019704.1	GCA_001606375	99,991	22381	92337	22381	100	2	0	16685	39065	22381	1	0	41319
NZ_CP024480.1	GCA_001606385	99,995	36786	77062	36786	100	2	0	20679	57464	36786	1	0	67920
AP019709.1	GCA_001606445	99,996	22276	86874	22275	100	0	1	16802	39077	1	22275	0	41129
NC_013354.1	GCA_001606545	100	15262	75546	15865	96	0	0	1	15262	15262	1	0	28184
NZ_CP027598.1	GCA_001606595	99,869	18274	74505	18270	100	6	2	15920	34179	18270	1	0	33597
CP027674.1	GCA_001606615	99,96	32468	133420	32464	100	3	5	30986	63447	1	32464	0	59876
CP027674.1	GCA_001606625	99,74	8455	133420	8463	100	8	4	20464	28918	8463	23	0	15479
NC_013354.1	GCA_001606635	100	15263	75546	15866	96	0	0	1	15263	604	15866	0	28186
NC_013354.1	GCA_001606695	100	15262	75546	15865	96	0	0	1	15262	604	15865	0	28184
NC_013354.1	GCA_001606725	100	15262	75546	15865	96	0	0	1	15262	604	15865	0	28184
CP027641.1	GCA_001606785	99,397	14093	126957	14123	100	76	5	53901	67986	1	14091	0	25545
NZ_CP027583.1	GCA_001606815	99,984	19127	88339	19358	99	2	1	18382	37507	1	19127	0	35303
CP027581.1	GCA_001606915	99,123	21883	118822	21870	100	154	16	34228	56085	21870	1	0	39312
NZ_CP027391.1	GCA_001606985	99,99	19513	98724	19512	100	1	1	57375	76887	1	19512	0	36021
CP027321.1	GCA_001607015	99,963	18957	84276	18956	100	6	1	46678	65634	1	18956	0	34967
NZ_CP031923.1	GCA_001607025	99,995	19772	95298	19772	100	1	0	64010	83781	1	19772	0	36507
NZ_CP027545.1	GCA_001607075	99,887	21171	101089	21197	100	20	4	17710	38877	28	21197	0	38959
NZ_CP027385.1	GCA_001607105	100	8679	118259	8679	100	0	0	66893	75571	1	8679	0	16028
NC_013354.1	GCA_001607175	100	15263	75546	15867	96	0	0	1	15263	15263	1	0	28186
NZ_CP027549.1	GCA_001607185	99,966	11776	94116	11776	100	2	2	57697	69470	11776	1	0	21723
NC_013369.1	GCA_001607205	99,995	19508	85167	19508	100	1	0	47878	67385	19508	1	0	36020
NZ_CP027386.1	GCA_001607255	100	17737	54452	17737	100	0	0	25909	43645	17737	1	0	32755
NZ_CP027588.1	GCA_001607265	99,991	11622	58109	11622	100	1	0	44283	55904	1	11622	0	21457
CP027378.1	GCA_001607355	99,982	32502	113102	32502	100	5	1	1397	33897	32502	1	0	59985
NC_013354.1	GCA_001607445	100	15468	75546	16071	96	0	0	1	15468	15468	1	0	28565
CP057835.1	GCA_001607605	99,96	55022	94579	55016	100	16	1	29020	84041	55016	1	0	101500
CP027641.1	GCA_001607635	99,817	10368	126957	10392	100	5	2	21586	31940	10367	1	0	19028
CP027641.1	GCA_001607655	99,305	12513	126957	12539	100	78	5	55481	67986	1	12511	0	22617
NZ_CP027389.1	GCA_001607675	99,995	20395	68062	20395	100	1	0	26671	47065	1	20395	0	37658
NC_019041.1	GCA_001607735	99,984	30665	92077	30715	100	2	1	56638	87299	30691	27	0	56597
CP042949.1	GCA_001607755	99,99	19670	118482	19670	100	2	0	59609	79278	19670	1	0	36313
NZ_CP024055.1	GCA_001607835	99,991	11530	88839	11772	98	1	0	55646	67175	243	11772	0	21287
NZ_CP027549.1	GCA_001607845	99,966	11776	94116	11776	100	2	2	57697	69470	1	11776	0	21723
NZ_CP031923.1	GCA_001607875	99,984	19335	95298	19335	100	0	2	5560	24891	1	19335	0	35685
NZ_CP006263.1	GCA_001607975	99,989	9108	98066	9108	100	1	0	83777	92884	1	9108	0	16814
NZ_CP027588.1	GCA_001607995	99,991	11340	58109	11383	100	1	0	44325	55664	11383	44	0	20936
AP019704.1	GCA_001608005	99,991	22377	92337	22377	100	2	0	16687	39063	22377	1	0	41312
NZ_CP031923.1	GCA_001608015	99,995	19507	95298	19507	100	1	0	64272	83778	19507	1	0	36018
NC_013366.1	GCA_001608055	99,977	13050	77690	13050	100	3	0	52670	65719	1	13050	0	24083
NZ_CP027220.1	GCA_001608085	99,995	19076	94104	19076	100	1	0	39763	58838	1	19076	0	35222
NZ_CP024480.1	GCA_001608105	99,997	36782	77062	36782	100	1	0	20681	57462	1	36782	0	67919
NZ_CP024480.1	GCA_001608205	99,99	30765	77062	30793	100	3	0	20526	51290	30793	29	0	56796
CP027378.1	GCA_001608245	99,783	7849	113102	7846	100	12	4	45839	53685	7846	1	0	14395
NZ_CP013028.1	GCA_001608265	99,956	9041	74656	9041	100	4	0	5995	15035	1	9041	0	16674
CP027641.1	GCA_001609215	99,832	10098	126957	10124	100	3	2	21810	31894	10097	1	0	18541
CP027641.1	GCA_001609235	99,855	10370	126957	10369	100	14	1	33184	43553	10369	1	0	19065
CP027378.1	GCA_001609315	99,98	29788	113102	29788	100	6	0	4372	34159	1	29788	0	54975
NC_013354.1	GCA_001609335	100	15263	75546	15867	96	0	0	1	15263	15263	1	0	28186
NZ_CP027549.1	GCA_001609735	99,958	11777	94116	11777	100	3	2	57697	69471	11777	1	0	21719
AP019707.1	GCA_001609765	100	19508	91036	19508	100	0	0	48784	68291	1	19508	0	36025
NZ_CP027588.1	GCA_001609815	99,954	28371	58109	29426	96	12	1	1	28370	1056	29426	0	52318
NC_013728.1	GCA_001609855	99,979	19116	111481	19394	99	3	1	87063	106177	36	19151	0	35277
CP027674.1	GCA_001614905	99,913	32242	133420	32215	100	1	4	31276	63517	32215	1	0	59359
NZ_CP027389.1	GCA_001616775	99,982	16259	68062	16455	99	3	0	26696	42954	16259	1	0	30009
NZ_CP027588.1	GCA_001616985	99,54	11082	58109	11304	98	33	8	44351	55416	225	11304	0	20166
NZ_CP027389.1	GCA_001617215	99,975	16275	68062	16487	99	4	0	26696	42970	16275	1	0	30033
NC_011350.1	GCA_001660185	99,986	21585	94644	21646	100	3	0	37908	59492	21646	62	0	39844
CP027674.1	GCA_001660195	99,928	12525	133420	12524	100	8	1	6605	19129	12524	1	0	23078
NZ_CP027585.1	GCA_001660245	99,995	36814	160576	36814	100	2	0	39108	75921	36814	1	0	67972
NZ_CP031899.1	GCA_001660255	99,987	60266	160712	60266	100	8	0	69386	129651	60266	1	0	111200
NZ_CP028382.1	GCA_001660275	100	36465	160675	36465	100	0	0	92547	129011	36465	1	0	67339
NC_019041.1	GCA_001660305	99,993	42226	92077	42922	98	2	1	45188	87412	1	42226	0	77959
NZ_CP027391.1	GCA_001660325	99,994	17805	98724	17805	100	1	0	22066	39870	17805	1	0	32875
NC_013369.1	GCA_001660335	100	22333	85167	22333	100	0	0	17192	39524	22333	1	0	41242
CP042949.1	GCA_001660345	99,981	10606	118482	10606	100	2	0	47826	58431	1	10606	0	19575
NC_013010.1	GCA_001677535	99,993	29183	94601	29896	98	2	0	1	29183	29183	1	0	53880
NC_013010.1	GCA_001677545	99,993	29183	94601	29986	97	2	0	1	29183	29183	1	0	53880
NZ_CP038341.1	GCA_001677555	99,987	30783	92839	31691	97	4	0	1	30783	909	31691	0	56824
NC_013010.1	GCA_001677565	99,99	29200	94601	30053	97	3	0	1	29200	854	30053	0	53906
NZ_CP038341.1	GCA_001677615	99,987	30805	92839	31776	97	4	0	1	30805	30805	1	0	56865
NZ_CP040315.1	GCA_001677625	99,954	30192	95631	30418	99	4	2	22017	52207	87	30269	0	55668
NC_017907.1	GCA_001677645	99,994	30779	92728	31530	98	2	0	1	30779	30779	1	0	56828
NC_013010.1	GCA_001677695	99,986	29183	94601	29899	98	4	0	1	29183	717	29899	0	53869
NZ_CP038341.1	GCA_001677705	99,987	30783	92839	31671	97	4	0	1	30783	889	31671	0	56824
NZ_CP034795.1	GCA_001677715	99,99	31329	94987	31756	99	2	1	55964	87291	31329	1	0	57836
NZ_CP038341.1	GCA_001677725	99,99	30783	92839	31671	97	3	0	1	30783	30783	1	0	56829
NZ_CP038341.1	GCA_001677775	99,994	30799	92839	31688	97	2	0	1	30799	30799	1	0	56865
NZ_CP038352.1	GCA_001677785	99,987	30357	92209	31474	96	4	0	1	30357	30791	435	0	56039
NZ_CP038341.1	GCA_001677805	99,987	30782	92839	31688	97	4	0	1	30782	907	31688	0	56822
NC_013010.1	GCA_001677855	99,993	29183	94601	29940	97	2	0	1	29183	758	29940	0	53880
NZ_CP034795.1	GCA_001677865	99,987	31701	94987	31701	100	4	0	55623	87323	31701	1	0	58519
NC_017907.1	GCA_001677875	99,994	30782	92728	31461	98	2	0	1	30782	680	31461	0	56833
NC_012487.1	GCA_001696245	99,959	51705	168100	51719	100	17	3	75486	127190	1	51701	0	95362
NZ_CP008958.1	GCA_001730745	100	42779	92076	42779	100	0	0	10724	53502	42779	1	0	78998
NC_013010.1	GCA_001884945	100	29474	94601	29850	99	0	0	1	29474	377	29850	0	54429
NC_013010.1	GCA_001884955	100	29474	94601	29640	99	0	0	1	29474	167	29640	0	54429
NC_013010.1	GCA_001884965	100	29474	94601	29850	99	0	0	1	29474	377	29850	0	54429
NC_013010.1	GCA_001884975	99,993	29221	94601	29346	100	2	0	1	29221	126	29346	0	53950
NZ_CP015816.1	GCA_001885025	99,947	16859	95910	16859	100	9	0	61660	78518	1	16859	0	31083

NZ_CP027354.1	GCA_001950695	99,914	15052	57720	15051	100	5	8	15837	30881	15051	1	0	27717
NZ_CP016626.1	GCA_001950735	99,99	19549	95598	19549	100	2	0	30769	50317	19549	1	0	36090
NZ_CP027585.1	GCA_001990935	99,993	29617	160576	29617	100	1	1	40125	69740	1	29617	0	54680
NZ_CP008958.1	GCA_001997045	100	42342	92076	42342	100	0	0	10945	53286	42342	1	0	78191
NZ_CP022408.1	GCA_001997405	100	15457	81950	15457	100	0	0	51776	67232	15457	1	0	28544
NZ_CP027343.1	GCA_002015385	99,823	13010	131410	13135	99	21	2	53762	66770	13135	127	0	23896
NZ_CP031909.1	GCA_002015535	100	36195	73224	36195	100	0	0	15143	51337	36195	1	0	66840
NZ_CP027220.1	GCA_002016065	99,984	19178	94104	19177	100	1	2	39761	58937	19177	1	0	35397
NC_013728.1	GCA_002016165	99,914	20948	111481	21493	97	10	4	86939	107882	21493	550	0	38577
NZ_CP015844.2	GCA_002027605	99,992	38633	95598	38633	100	1	2	1947	40577	1	38633	0	71324
NZ_CP038420.1	GCA_002027645	100	32634	98042	32634	100	0	0	31877	64510	32634	1	0	60264
NZ_CP028686.1	GCA_002133435	100	42290	92724	42525	99	0	0	7949	50238	42525	236	0	78095
CP042949.1	GCA_002133505	100	26274	118482	26274	100	0	0	59507	85780	26274	1	0	48519
CP027674.1	GCA_002133535	99,894	8512	133420	8632	99	3	3	20407	28918	8632	127	0	15664
NC_013366.1	GCA_002133695	100	13980	77690	13980	100	0	0	52552	66531	13980	1	0	25817
NC_013354.1	GCA_002133755	99,993	15364	75546	16069	96	1	0	1	15364	15364	1	0	28367
NZ_CP038382.1	GCA_002133775	100	40866	91912	40866	100	0	0	31519	72384	40866	1	0	75466
NZ_CP031899.1	GCA_002133915	99,917	59974	160712	59971	100	44	4	69643	129613	59971	1	0	110500
NC_013354.1	GCA_002133985	99,993	15364	75546	16069	96	1	0	1	15364	15364	1	0	28367
NZ_CP022408.1	GCA_002134175	99,956	15805	81950	15805	100	0	1	51542	67339	15805	1	0	29141
NZ_CP038362.1	GCA_002134285	99,986	41876	95928	41876	100	0	1	34513	76382	1	41876	0	77292
NC_011350.1	GCA_002134385	99,819	33710	94644	33685	100	8	3	25512	59193	33685	1	0	61863
NZ_CP024480.1	GCA_002134405	99,976	37201	77062	37200	100	8	1	20472	57672	37200	1	0	68646
CP058232.1	GCA_002134445	100	37598	94605	37598	100	0	0	7645	45242	37598	1	0	69431
NZ_CP027458.1	GCA_002134455	99,886	27254	107796	27253	100	25	4	12874	40122	27253	1	0	50152
NZ_CP038362.1	GCA_002134505	99,986	41876	95928	41876	100	0	1	34513	76382	1	41876	0	77292
NZ_CP040312.1	GCA_002134585	99,997	31392	92909	31393	100	1	0	22068	53459	2	31393	0	57965
NZ_CP027385.1	GCA_002134685	99,506	8907	118259	9335	95	30	11	10036	18932	1	8903	0	16192
NZ_CP038406.1	GCA_002144055	99,998	43076	92740	43076	100	0	1	7950	51024	1	43076	0	79540
NZ_CP038406.1	GCA_002144065	100	43075	92740	43075	100	0	0	7950	51024	1	43075	0	79545
NZ_CP038341.1	GCA_002144095	99,997	30835	92839	31788	97	1	0	1	30835	30835	1	0	56937
NZ_CP038341.1	GCA_002144105	99,997	30821	92839	31620	97	1	0	1	30821	30821	1	0	56911
NZ_CP031923.1	GCA_002144165	100	19990	95298	19990	100	0	0	63891	83880	19990	1	0	36915
NZ_CP031923.1	GCA_002144185	99,987	23163	95298	23163	100	3	0	60718	83880	23163	1	0	42758
NZ_CP028616.1	GCA_002164015	100	31316	94495	31316	100	0	0	19653	50968	31316	1	0	57830
NZ_CP017437.1	GCA_002164025	100	30972	92565	30972	100	0	0	22716	53687	1	30972	0	57195
NZ_CP040108.1	GCA_002164035	100	30785	93190	31112	99	0	0	1	30785	328	31112	0	56850
NZ_CP028616.1	GCA_002164045	100	31338	94495	31338	100	0	0	19631	50968	1	31338	0	57871
NZ_CP038345.1	GCA_002164095	100	31314	92577	31314	100	0	0	31698	63011	31314	1	0	57827
NZ_CP038314.1	GCA_002164105	99,977	17219	97630	17219	100	4	0	18815	36033	1	17219	0	31776
NZ_CP017437.1	GCA_002164115	100	38415	92565	38415	100	0	0	15273	53687	38415	1	0	70940
NZ_CP017437.1	GCA_002164125	100	38415	92565	38415	100	0	0	15273	53687	38415	1	0	70940
NZ_CP017437.1	GCA_002164175	99,995	38415	92565	38415	100	2	0	15273	53687	38415	1	0	70929
NZ_CP038345.1	GCA_002164185	99,997	30555	92577	30887	99	1	0	1	30555	333	30887	0	56419
NZ_CP017437.1	GCA_002164195	100	31120	92565	31120	100	0	0	15273	46392	31120	1	0	57468
NZ_CP017435.1	GCA_002164205	99,997	31132	92624	31132	100	1	0	15273	46404	1	31132	0	57485
NZ_CP038308.1	GCA_002164275	99,997	35429	93167	35429	100	1	0	3061	38489	1	35429	0	65420
NZ_CP045976.1	GCA_002164285	99,993	30079	94640	30078	100	1	1	3007	33085	30078	1	0	55533
NZ_CP017437.1	GCA_002164295	100	38415	92565	38415	100	0	0	15273	53687	38415	1	0	70940
NZ_CP028616.1	GCA_002164335	100	31338	94495	31338	100	0	0	19631	50968	1	31338	0	57871
NZ_CP038356.1	GCA_002164355	99,991	22918	98062	22918	100	2	0	36828	59745	22918	1	0	42311
NC_017907.1	GCA_002164375	100	31081	92728	31532	99	0	0	1	31081	31081	1	0	57396
NZ_CP040310.1	GCA_002164415	99,988	42958	93175	42956	100	2	2	7987	50943	1	42956	0	79298
NZ_CP038356.1	GCA_002164435	99,976	24714	98062	24714	100	6	0	36828	61541	24714	1	0	45605
NZ_CP028625.1	GCA_002164445	99,998	43223	94493	43223	100	0	1	7745	50966	1	43223	0	79811
NZ_CP017437.1	GCA_002164455	100	31004	92565	31004	100	0	0	22683	53686	1	31004	0	57254
NZ_CP017437.1	GCA_002164515	99,997	31107	92565	31107	100	1	0	22581	53687	31107	1	0	57439
NZ_CP017435.1	GCA_002164525	99,987	31160	92624	31160	100	4	0	15273	46432	31160	1	0	57520
NZ_CP038345.1	GCA_002164535	100	30555	92577	30824	99	0	0	1	30555	270	30824	0	56425
NZ_CP017437.1	GCA_002165415	100	38415	92565	38415	100	0	0	15273	53687	38415	1	0	70940
NZ_CP027585.1	GCA_002173115	98,2	20719	160576	20775	100	336	29	120025	140723	62	20763	0	36160
NC_007365.1	GCA_002173195	99,318	17604	165548	17934	98	101	6	103420	121022	56	17641	0	31826
NZ_CP044146.1	GCA_002175655	99,916	23732	92722	24072	99	13	2	57977	81708	348	24072	0	43707
NZ_CP016626.1	GCA_002175715	99,974	23200	95598	23200	100	0	1	18134	41327	1	23200	0	42804
NZ_CP041624.1	GCA_002175875	99,745	17651	95081	18428	96	16	2	38049	55671	432	18081	0	32319
NZ_CP016626.1	GCA_002176015	99,972	21137	95598	21137	100	0	1	18133	39263	21137	1	0	38994
NZ_CP016626.1	GCA_002176385	99,969	22905	95598	22906	100	1	1	18036	40934	22906	2	0	42254
NZ_CP041624.1	GCA_002176505	100	22389	95081	22443	100	0	0	31952	54340	22389	1	0	41345
NZ_CP016626.1	GCA_002176745	99,972	21257	95598	21257	100	0	1	18134	39384	21257	1	0	39216
NZ_CP016626.1	GCA_002194835	99,975	23556	95598	23556	100	0	1	17805	41354	1	23556	0	43462
NZ_CP038289.1	GCA_002194915	100	31279	96920	31939	98	0	0	1	31279	661	31939	0	57762
NC_013010.1	GCA_002195075	99,993	29554	94601	29581	100	2	0	1	29554	28	29581	0	54565
NC_013010.1	GCA_002195255	100	29426	94601	29784	99	0	0	1	29426	359	29784	0	54340
NC_013010.1	GCA_002195405	100	29426	94601	29677	99	0	0	1	29426	252	29677	0	54340
NC_013010.1	GCA_002195805	100	29554	94601	29846	99	0	0	1	29554	293	29846	0	54577
NC_013010.1	GCA_002195825	99,99	29554	94601	29697	100	3	0	1	29554	144	29697	0	54560
NZ_CP038341.1	GCA_002198005	100	30785	92839	31393	98	0	0	1	30785	609	31393	0	56850
NZ_CP018244.1	GCA_002224665	100	30835	92522	31663	97	0	0	1	30835	829	31663	0	56942
NZ_CP031923.1	GCA_002286445	99,98	19770	95298	19770	100	4	0	64010	83779	19770	1	0	36487
NZ_CP027767.1	GCA_002286535	99,943	8711	66545	8711	100	4	1	10373	19082	1	8711	0	16057
NZ_CP031899.1	GCA_002319155	99,977	51160	160712	51160	100	12	0	78332	129491	1	51160	0	94409
NZ_CP061760.1	GCA_002319415	99,97	59972	172576	59971	100	15	2	64875	124844	59971	1	0	110600
NZ_CP027585.1	GCA_002379215	99,983	30078	160576	30078	100	1	1	40146	70219	1	30078	0	55513
NZ_CP027585.1	GCA_002379235	99,997	35395	160576	35395	100	1	0	40375	75769	35395	1	0	65357
NZ_CP031899.1	GCA_002379285	99,99	59132	160712	59132	100	6	0	69770	128901	1	59132	0	109200
NZ_CP028382.1	GCA_002379295	99,986	35719	160675	35719	100	1	1	92610	128324	35719	1	0	65930
NZ_CP028382.1	GCA_002379315	99,986	35715	160675	35715	100	1	1	92610	128320	1	35715	0	65922
NZ_CP027389.1	GCA_002457935	99,994	15626	68062	15625	100	0	1	48368	63993	15625	1	0	28849
NZ_CP027585.1	GCA_002457955	100	30314	160576	30314	100	0	0	39608	69921	1	30314	0	55980
NC_013366.1	GCA_002457965	99,965	11399	77690	11512	99	4	0	54677	66075	1	11399	0	21028
CP042949.1	GCA_002458025	99,991	11704	118482	11703	100	0	1	31674	43377	1	11703	0	21606
NZ_CP038341.1	GCA_002458325	100	30785	92839	31419	98	0	0	1	30785	30785	1	0	56850
NC_017907.1	GCA_002458345	100	30785	92728	31394	98	0	0	1	30785	30785	1	0	56850

AP019709.1	GCA_002458635	100	18937	86874	18937	100	0	0	43927	62863	18937	1	0	34971
AP019709.1	GCA_002458655	100	18937	86874	18937	100	0	0	43927	62863	18937	1	0	34971
NZ_CP027385.1	GCA_002458785	100	8580	118259	8580	100	0	0	67043	75622	8580	1	0	15845
CP027674.1	GCA_002458885	99,937	23691	133420	23738	100	11	4	104045	127735	23687	1	0	43663
NZ_CP028111.1	GCA_002459225	99,975	12034	83211	12034	100	3	0	17741	29774	12034	1	0	22207
NZ_CP027441.1	GCA_002460005	99,983	77177	173714	77177	100	13	0	77033	154209	1	77177	0	142400
NZ_CP027545.1	GCA_002460035	99,982	27856	101089	27855	100	0	5	11077	38928	1	27855	0	51408
NZ_CP027588.1	GCA_002460175	99,971	27592	58109	29029	95	7	1	1	27591	27923	332	0	50907
AP019704.1	GCA_002460705	100	18743	92337	18922	99	0	0	50335	69077	18743	1	0	34612
NZ_CP027585.1	GCA_002461345	100	30314	160576	30314	100	0	0	39608	69921	1	30314	0	55980
NZ_CP027385.1	GCA_002461565	100	8580	118259	8580	100	0	0	67043	75622	8580	1	0	15845
NZ_CP038382.1	GCA_002461735	100	40766	91912	40766	100	0	0	31569	72334	1	40766	0	75281
NZ_CP027598.1	GCA_002462035	99,993	29579	74505	29579	100	0	2	4652	34228	1	29579	0	54610
NZ_CP027453.1	GCA_002462215	99,846	44023	159611	44077	100	40	5	58522	102538	1	44001	0	80893
NZ_CP027585.1	GCA_002462275	100	29654	160576	29654	100	0	0	40426	70079	1	29654	0	54761
NZ_CP027385.1	GCA_002462285	100	8580	118259	8580	100	0	0	67043	75622	8580	1	0	15845
NZ_CP027451.1	GCA_002463845	99,97	47457	173649	47457	100	4	2	43684	91130	1	47457	0	87550
NC_013354.1	GCA_002467465	99,987	15364	75546	16068	96	2	0	1	15364	15364	1	0	28361
NZ_CP017670.1	GCA_002469465	99,994	34192	92755	34191	100	1	1	25047	59238	34191	1	0	63128
NC_017907.1	GCA_002473655	100	30785	92728	31383	98	0	0	1	30785	599	31383	0	56850
NZ_CP028651.1	GCA_002473685	99,998	42250	94057	42981	98	0	1	7999	50247	42981	732	0	78014
NZ_CP038426.1	GCA_002473745	99,998	41489	92986	41489	100	1	0	31929	73417	41489	1	0	76611
NZ_CP017441.1	GCA_002473765	99,998	42972	92729	42971	100	0	1	7999	50970	42971	1	0	79347
NZ_CP040571.1	GCA_002473775	99,998	42979	92763	42979	100	1	0	23811	66789	42979	1	0	79362
NC_017907.1	GCA_002473795	100	30785	92728	31355	98	0	0	1	30785	571	31355	0	56850
NZ_CP038371.1	GCA_002473855	100	30785	92990	31405	98	0	0	1	30785	30785	1	0	56850
NC_013010.1	GCA_002473875	100	29171	94601	29584	99	0	0	1	29171	414	29584	0	53869
NZ_CP062161.1	GCA_002473915	99,988	43008	104844	43005	100	2	3	3821	46828	1	43005	0	79390
NC_013010.1	GCA_002473945	100	29171	94601	29506	99	0	0	1	29171	29171	1	0	53869
NC_013010.1	GCA_002473955	99,986	29185	94601	29568	99	1	1	1	29185	29182	1	0	53869
NC_013010.1	GCA_002473995	100	29171	94601	29887	98	0	0	1	29171	717	29887	0	53869
NC_019041.1	GCA_002474035	99,998	42243	92077	42970	98	0	1	45171	87412	42970	728	0	78001
NZ_CP064169.1	GCA_002474105	100	43673	95164	43673	100	0	0	30846	74518	43673	1	0	80649
NC_017907.1	GCA_002474125	99,997	30785	92728	31691	97	1	0	1	30785	907	31691	0	56844
NZ_CP038362.1	GCA_002474135	100	41770	95928	41770	100	0	0	34563	76332	41770	1	0	77135
NZ_CP017445.1	GCA_002474185	99,981	42963	92726	42957	100	2	1	7999	50961	42957	1	0	79288
NZ_CP028651.1	GCA_002474205	99,998	42250	94057	42981	98	0	1	7999	50247	42981	732	0	78014
NZ_CP028651.1	GCA_002474225	99,998	42250	94057	42981	98	0	1	7999	50247	42981	732	0	78014
NZ_CP028651.1	GCA_002475675	99,998	42250	94057	42981	98	0	1	7999	50247	42981	732	0	78014
NZ_CP038371.1	GCA_002475705	100	30785	92990	31405	98	0	0	1	30785	30785	1	0	56850
NC_019041.1	GCA_002475725	99,998	42242	92077	42969	98	1	0	45171	87412	42969	728	0	78001
NZ_CP038426.1	GCA_002475745	99,998	41490	92986	41490	100	1	0	31928	73417	1	41490	0	76613
NZ_CP038426.1	GCA_002475775	99,998	41490	92986	41490	100	1	0	31928	73417	1	41490	0	76613
NZ_CP038371.1	GCA_002475795	100	32009	92990	32009	100	0	0	31928	63936	32009	1	0	59110
NZ_CP038426.1	GCA_002475825	99,998	41489	92986	41489	100	1	0	31929	73417	1	41489	0	76611
NC_017907.1	GCA_002475845	99,994	30785	92728	31680	97	2	0	1	30785	30785	1	0	56839
NC_017907.1	GCA_002475865	100	30785	92728	31346	98	0	0	1	30785	30785	1	0	56850
NZ_CP034805.1	GCA_002475875	99,998	42965	94014	42965	100	1	0	33332	76296	1	42965	0	79336
NZ_CP064169.1	GCA_002475895	100	21069	95164	21069	100	0	0	34445	55513	1	21069	0	38908
NC_019041.1	GCA_002475935	99,967	42242	92077	42967	98	12	1	45171	87412	42967	728	0	77927
NZ_CP044146.1	GCA_002475965	99,959	43672	92722	43654	100	0	1	38113	81784	1	43654	0	80531
NZ_CP034805.1	GCA_002475975	99,998	42965	94014	42965	100	1	0	33332	76296	1	42965	0	79336
NZ_CP018626.1	GCA_002476005	99,995	43168	107692	43257	100	1	1	26287	69453	43168	1	0	79704
NZ_CP038341.1	GCA_002476015	99,997	30785	92839	31706	97	1	0	1	30785	30785	1	0	56844
NC_017907.1	GCA_002476045	100	30785	92728	31364	98	0	0	1	30785	30785	1	0	56850
NZ_CP038293.1	GCA_002476085	99,997	32412	95611	32613	99	1	0	31929	64340	32613	202	0	59849
NC_017907.1	GCA_002476125	100	30785	92728	31386	98	0	0	1	30785	30785	1	0	56850
NZ_CP038371.1	GCA_002476135	100	30785	92990	31405	98	0	0	1	30785	30785	1	0	56850
NC_017907.1	GCA_002476165	100	30785	92728	31394	98	0	0	1	30785	30785	1	0	56850
NZ_CP038323.1	GCA_002476195	99,977	43653	92701	43645	100	2	2	43456	87108	1	43645	0	80550
NC_017907.1	GCA_002476215	100	30785	92728	31394	98	0	0	1	30785	30785	1	0	56850
CP027674.1	GCA_002476305	99,909	31987	133420	31960	100	2	4	31296	63282	31960	1	0	58883
NZ_CP038304.1	GCA_002486565	100	32194	96937	32194	100	0	0	31927	64120	1	32194	0	59452
NC_017907.1	GCA_002486575	100	30785	92728	31319	98	0	0	1	30785	30785	1	0	56850
NC_017907.1	GCA_002486625	100	30785	92728	31824	97	0	0	1	30785	1040	31824	0	56850
NC_013010.1	GCA_002486645	99,986	29185	94601	29560	99	1	1	1	29185	29182	1	0	53869
NZ_CP040310.1	GCA_002486685	99,991	42958	93175	42957	100	2	2	7987	50943	1	42957	0	79305
NZ_CP040571.1	GCA_002486695	99,998	42979	92763	42979	100	1	0	23811	66789	42979	1	0	79362
NZ_CP062161.1	GCA_002486775	99,965	43018	104844	43018	100	5	2	3821	46828	1	43018	0	79347
NC_017907.1	GCA_002509765	100	30785	92728	31394	98	0	0	1	30785	30785	1	0	56850
NZ_CP027385.1	GCA_002509905	100	8580	118259	8580	100	0	0	67043	75622	8580	1	0	15845
NZ_CP028124.1	GCA_002514585	99,965	11578	83012	11654	99	4	0	49501	61078	77	11654	0	21359
NZ_CP006028.1	GCA_002515075	99,988	17285	87120	17285	100	2	0	52216	69500	1	17285	0	31909
NZ_CP031909.1	GCA_002515145	99,995	37099	73224	37101	100	2	0	15330	52428	1	37099	0	68498
NZ_CP027588.1	GCA_002515585	99,954	28381	58109	29491	96	1	2	1	28369	28381	1	0	52327
NC_013354.1	GCA_002515605	100	15314	75546	15969	96	0	0	1	15314	656	15969	0	28280
NZ_CP013028.1	GCA_002516265	100	8231	74656	8231	100	0	0	6688	14918	1	8231	0	15200
NZ_CP012498.1	GCA_002516285	99,992	48456	213847	48456	100	0	4	97569	146020	1	48456	0	89456
NZ_CP012499.1	GCA_002517145	99,676	12020	223952	12075	100	39	0	43992	56011	12020	1	0	21981
NZ_CP051657.1	GCA_002518395	99,981	15683	74390	15970	98	1	2	58710	74390	1	15683	0	28943
CP027674.1	GCA_002518445	99,902	8168	133420	8166	100	6	2	20384	28551	1	8166	0	15038
NZ_CP061760.1	GCA_002520335	99,973	59591	172576	59591	100	15	1	65205	124794	1	59591	0	110000
NZ_CP027576.1	GCA_002530855	100	13975	78427	13975	100	0	0	8003	21977	1	13975	0	25808
NC_013366.1	GCA_002530875	100	13958	77690	13958	100	0	0	52569	66526	13958	1	0	25776
NZ_AP018805.1	GCA_002530885	99,96	14932	78434	15184	98	2	2	10172	25102	15184	256	0	27538
NZ_CP027385.1	GCA_002530945	100	8880	118259	8880	100	0	0	66793	75672	1	8880	0	16399
NZ_CP027385.1	GCA_002530955	100	8880	118259	8880	100	0	0	66793	75672	8880	1	0	16399
NC_013728.1	GCA_002531015	99,986	21339	111481	21339	100	2	1	87069	108406	21339	1	0	39388
NZ_CP027318.1	GCA_002531025	99,921	16371	81954	16371	100	13	0	54633	71003	16371	1	0	30160
NC_013728.1	GCA_002531035	99,986	21339	111481	21339	100	2	1	87069	108406	1	21339	0	39388
NZ_CP027385.1	GCA_002531045	100	8880	118259	8880	100	0	0	66793	75672	8880	1	0	16399
NZ_CP027385.1	GCA_002531095	100	8880	118259	8880	100	0	0	66793	75672	1	8880	0	16399
NZ_CP027385.1	GCA_002531175	100	8682	118259	8682	100	0	0	66991	75672	1	8682	0	16033

NZ_CP024055.1	GCA_002531185	99,508	6504	88839	6780	96	20	4	16244	22736	149	6651	0	11823
NZ_CP037944.1	GCA_002531205	99,99	19742	88848	19742	100	2	0	50067	69808	1	19742	0	36446
NC_013010.1	GCA_002531275	100	29221	94601	29979	97	0	0	1	29221	29221	1	0	53962
NC_013010.1	GCA_002531305	100	29221	94601	29979	97	0	0	1	29221	29221	1	0	53962
NZ_CP017437.1	GCA_002531375	100	38703	92565	38844	100	0	0	15223	53925	38844	142	0	71472
NC_013354.1	GCA_002531395	99,993	15364	75546	16069	96	1	0	1	15364	15364	1	0	28367
NC_011350.1	GCA_002531445	99,822	33710	94644	33685	100	7	3	25512	59193	33685	1	0	61869
NZ_CP037944.1	GCA_002531515	100	19737	88848	19737	100	0	0	50072	69808	1	19737	0	36448
NZ_CP037944.1	GCA_002734545	99,99	19969	88848	20097	99	1	1	16255	36222	129	20097	0	36863
NZ_CP027354.1	GCA_002734725	99,957	13942	57720	13942	100	2	4	19490	33427	13942	1	0	25710
NZ_CP027601.1	GCA_002734765	99,995	19368	92590	19368	100	0	1	12626	31992	1	19368	0	35759
NZ_CP027547.1	GCA_002734785	99,976	16509	95367	16588	100	2	2	48738	65246	82	16588	0	30463
NZ_CP027601.1	GCA_002734805	99,995	19383	92590	19383	100	0	1	12610	31991	1	19383	0	35787
NZ_CP027354.1	GCA_002734825	99,937	15991	57720	15991	100	3	7	17444	33427	15991	1	0	29468
NZ_CP024055.1	GCA_002734845	99,523	6503	88839	6678	97	20	3	16244	22736	96	6597	0	11827
NZ_CP037944.1	GCA_002734865	100	19627	88848	19627	100	0	0	50126	69752	19627	1	0	36245
NZ_CP037944.1	GCA_002734885	100	19644	88848	19644	100	0	0	50126	69769	1	19644	0	36276
NZ_CP037942.1	GCA_002734905	99,991	11757	157534	11757	100	1	0	61491	73247	11757	1	0	21706
AP019709.1	GCA_002734925	99,994	17652	86874	17652	100	1	0	16758	34409	1	17652	0	32592
NZ_CP027391.1	GCA_002734945	99,995	19617	98724	19616	100	0	1	57316	76932	1	19616	0	36219
NZ_CP037942.1	GCA_002734965	99,991	11758	157534	11758	100	1	0	61491	73248	1	11758	0	21708
NZ_CP037942.1	GCA_002735025	99,992	12145	157534	12145	100	1	0	40031	52175	12145	1	0	22423
AP019709.1	GCA_002735065	99,994	17652	86874	17652	100	1	0	16758	34409	17652	1	0	32592
NZ_CP027576.1	GCA_002735085	99,875	16846	78427	17462	96	19	2	23514	40357	17462	617	0	30991
NZ_CP037944.1	GCA_002735105	99,995	19602	88848	19602	100	1	0	50126	69727	1	19602	0	36193
NZ_CP027354.1	GCA_002735145	99,96	14841	57720	14841	100	1	5	18592	33427	14841	1	0	27368
NZ_CP024055.1	GCA_002735165	99,493	6507	88839	6647	98	21	4	16244	22739	6552	47	0	11823
NZ_CP037944.1	GCA_002735205	99,979	19236	88848	19361	99	3	1	16255	35489	126	19361	0	35499
NZ_CP027354.1	GCA_002764215	99,97	13559	57720	13559	100	1	3	19872	33427	13559	1	0	25013
AP019707.1	GCA_002764255	100	19602	91036	19602	100	0	0	48737	68338	19602	1	0	36199
NZ_CP058683.1	GCA_002764315	99,99	19287	92578	19287	100	1	1	64598	83883	19287	1	0	35604
NZ_CP058683.1	GCA_002764335	99,979	23283	92578	23283	100	5	0	24198	47480	23283	1	0	42968
AP019709.1	GCA_002764355	99,928	16745	86874	16872	99	12	0	17768	34512	1	16745	0	30877
NC_013369.1	GCA_002764375	99,995	19863	85167	19988	99	1	0	47622	67484	19863	1	0	36675
NZ_CP027386.1	GCA_002764395	99,979	19147	54452	19145	100	0	4	835	19979	19145	1	0	35333
NZ_CP037946.1	GCA_002764415	99,979	19162	96016	19162	100	3	1	40809	59969	19162	1	0	35362
NZ_CP024055.1	GCA_002764435	99,508	6503	88839	6781	96	21	3	16244	22736	150	6651	0	11821
NZ_CP027601.1	GCA_002764455	99,956	20555	92590	21220	97	8	1	12676	33229	129	20683	0	37907
NZ_CP027391.1	GCA_002764475	99,995	19701	98724	19700	100	0	1	57281	76981	1	19700	0	36374
NC_013369.1	GCA_002764535	100	22357	85167	22357	100	0	0	17179	39535	1	22357	0	41286
AP019709.1	GCA_002764555	99,994	17652	86874	17652	100	1	0	16758	34409	1	17652	0	32592
AP019704.1	GCA_002764575	99,991	22157	92337	22157	100	2	0	16758	38914	1	22157	0	40906
NC_013369.1	GCA_002764595	99,996	22356	85167	22356	100	1	0	17180	39535	22356	1	0	41279
NZ_CP027391.1	GCA_002764615	99,995	19603	98724	19602	100	0	1	57330	76932	1	19602	0	36193
NZ_CP027391.1	GCA_002764675	99,995	19603	98724	19602	100	0	1	57330	76932	1	19602	0	36193
NZ_CP027391.1	GCA_002764695	99,995	19617	98724	19616	100	0	1	57316	76932	1	19616	0	36219
AP019709.1	GCA_002764715	99,994	17650	86874	17650	100	1	0	16758	34407	1	17650	0	32588
NZ_CP027391.1	GCA_002764755	99,99	19840	98724	19839	100	1	1	57093	76932	19839	1	0	36625
NZ_CP027391.1	GCA_002764795	99,995	19617	98724	19616	100	0	1	57316	76932	1	19616	0	36219
NZ_CP027391.1	GCA_002764815	99,995	19603	98724	19602	100	0	1	57330	76932	1	19602	0	36193
NZ_CP027601.1	GCA_002764835	99,995	19366	92590	19366	100	0	1	12627	31991	19366	1	0	35755
NZ_CP027391.1	GCA_002764855	99,995	19617	98724	19616	100	0	1	57316	76932	1	19616	0	36219
NZ_CP027391.1	GCA_002764875	99,995	19603	98724	19602	100	0	1	57330	76932	19602	1	0	36193
NZ_CP027391.1	GCA_002764895	99,995	19604	98724	19603	100	0	1	57329	76932	1	19603	0	36195
NZ_CP027391.1	GCA_002764915	99,99	19617	98724	19616	100	1	1	57316	76932	1	19616	0	36213
NZ_CP027386.1	GCA_002764935	100	17828	54452	17828	100	0	0	25863	43690	17828	1	0	32923
NZ_CP027386.1	GCA_002764955	100	17828	54452	17828	100	0	0	25863	43690	17828	1	0	32923
NZ_CP027386.1	GCA_002764975	100	17825	54452	17825	100	0	0	25863	43687	1	17825	0	32917
AP019709.1	GCA_002764995	99,994	17652	86874	17652	100	1	0	16758	34409	17652	1	0	32592
AP019709.1	GCA_002765015	99,994	17755	86874	17828	100	1	0	16758	34512	1	17755	0	32782
NC_013369.1	GCA_002765035	99,991	22157	85167	22157	100	2	0	17181	39337	22157	1	0	40906
CP027321.1	GCA_002765055	99,974	19054	84276	19053	100	2	2	46631	65682	19053	1	0	35155
NZ_CP027391.1	GCA_002765075	99,995	19603	98724	19602	100	0	1	57330	76932	1	19602	0	36193
NZ_CP027391.1	GCA_002765095	99,99	19840	98724	19839	100	1	1	57093	76932	19839	1	0	36625
AP019709.1	GCA_002765115	99,994	17652	86874	17652	100	1	0	16758	34409	1	17652	0	32592
NZ_CP027386.1	GCA_002765175	100	17828	54452	17828	100	0	0	25863	43690	17828	1	0	32923
NZ_CP006028.1	GCA_002765195	99,982	16691	87120	16757	100	2	1	51742	68432	16690	1	0	30805
NZ_CP027391.1	GCA_002765215	99,995	19603	98724	19602	100	0	1	57330	76932	1	19602	0	36193
NZ_CP027386.1	GCA_002765235	99,994	17828	54452	17828	100	1	0	25863	43690	1	17828	0	32917
NZ_CP006028.1	GCA_002765255	99,946	16691	87120	16757	100	2	4	51742	68432	16684	1	0	30766
NZ_CP037944.1	GCA_002765275	99,986	21152	88848	21152	100	1	1	14079	35228	1	21152	0	39042
NZ_CP027391.1	GCA_002765295	99,99	19617	98724	19616	100	1	1	57316	76932	19616	1	0	36213
CP027321.1	GCA_002765315	99,99	19052	84276	19051	100	1	1	46631	65682	1	19051	0	35170
NC_013369.1	GCA_002765335	99,996	22374	85167	22374	100	1	0	17162	39535	22374	1	0	41312
NZ_CP027391.1	GCA_002765355	99,934	19603	98724	19602	100	12	1	57330	76932	1	19602	0	36147
NZ_CP027391.1	GCA_002765375	99,99	19603	98724	19602	100	1	1	57330	76932	19602	1	0	36188
NZ_CP027386.1	GCA_002765415	99,994	17828	54452	17828	100	1	0	25863	43690	17828	1	0	32917
AP019709.1	GCA_002765435	99,989	17650	86874	17650	100	2	0	16758	34407	17650	1	0	32583
AP019709.1	GCA_002765455	99,994	17755	86874	17828	100	1	0	16758	34512	1	17755	0	32782
NC_013369.1	GCA_002765475	99,995	22160	85167	22160	100	1	0	17180	39339	22160	1	0	40917
NZ_CP027391.1	GCA_002765495	99,995	19603	98724	19602	100	0	1	57330	76932	1	19602	0	36193
NZ_CP027391.1	GCA_002765515	99,99	19603	98724	19602	100	1	1	57330	76932	1	19602	0	36188
NC_013369.1	GCA_002765535	99,996	22356	85167	22356	100	1	0	17180	39535	22356	1	0	41279
NZ_CP027391.1	GCA_002765555	99,995	19603	98724	19602	100	0	1	57330	76932	19602	1	0	36193
NZ_CP027386.1	GCA_002765575	99,994	17828	54452	17828	100	1	0	25863	43690	1	17828	0	32917
NC_013369.1	GCA_002765595	99,996	22356	85167	22356	100	1	0	17180	39535	22356	1	0	41279
NC_013369.1	GCA_002765635	99,987	22357	85167	22357	100	2	1	17180	39535	22357	1	0	41268
NZ_CP027391.1	GCA_002765695	99,99	19603	98724	19602	100	1	1	57330	76932	19602	1	0	36188
NZ_CP027391.1	GCA_002765715	99,98	19603	98724	19602	100	3	1	57330	76932	19602	1	0	36176
NZ_CP027391.1	GCA_002765735	99,995	19617	98724	19616	100	0	1	57316	76932	19616	1	0	36219
NC_013369.1	GCA_002765755	99,996	22356	85167	22356	100	1	0	17180	39535	22356	1	0	41279
AP019704.1	GCA_002765775	99,991	22157	92337	22157	100	2	0	16758	38914	22157	1	0	40906
NC_013369.1	GCA_002765815	99,995	19602	85167	19602	100	1	0	47831	67432	19602	1	0	36193

NZ_CP024055.1	GCA_002765915	99,508	6503	88839	6646	98	21	3	16244	22736	96	6597	0	11821
NZ_CP027391.1	GCA_002765935	99,994	17828	98724	17828	100	1	0	22054	39881	1	17828	0	32917
NC_013369.1	GCA_002765955	100	19336	85167	19414	100	0	0	47826	67161	1	19336	0	35707
NZ_CP027386.1	GCA_002765975	99,994	17828	54452	17828	100	1	0	25863	43690	1	17828	0	32917
NC_013369.1	GCA_002766035	99,991	22356	85167	22356	100	2	0	17180	39535	22356	1	0	41273
NZ_CP027386.1	GCA_002766055	99,983	17828	54452	17828	100	3	0	25863	43690	17828	1	0	32906
AP019709.1	GCA_002766095	99,983	17652	86874	17652	100	3	0	16758	34409	1	17652	0	32581
NC_013369.1	GCA_002766115	100	19602	85167	19602	100	0	0	47830	67431	19602	1	0	36199
AP019709.1	GCA_002766135	99,988	17266	86874	17339	100	2	0	17247	34512	17339	74	0	31874
NC_013369.1	GCA_002766155	99,996	22356	85167	22356	100	1	0	17180	39535	1	22356	0	41279
NZ_CP027391.1	GCA_002766195	99,99	19603	98724	19602	100	1	1	57330	76932	1	19602	0	36188
NC_013369.1	GCA_002766215	100	19590	85167	19590	100	0	0	47843	67432	19590	1	0	36176
NC_013369.1	GCA_002766275	100	22356	85167	22356	100	0	0	17180	39535	22356	1	0	41284
NZ_CP027386.1	GCA_002766295	99,994	17828	54452	17827	100	0	1	25863	43690	17827	1	0	32915
NC_013369.1	GCA_002766315	100	22356	85167	22356	100	0	0	17180	39535	22356	1	0	41284
NZ_CP027386.1	GCA_002766355	100	17653	54452	17653	100	0	0	25862	43514	17653	1	0	32600
AP019709.1	GCA_002766375	99,994	17755	86874	17828	100	1	0	16758	34512	17828	74	0	32782
NC_013369.1	GCA_002766395	99,991	22356	85167	22356	100	2	0	17180	39535	22356	1	0	41277
NZ_CP027391.1	GCA_002766435	99,995	19843	98724	20269	98	0	1	57090	76932	19842	1	0	36636
NZ_CP027386.1	GCA_002766455	100	17828	54452	17828	100	0	0	25863	43690	17828	1	0	32923
NZ_CP027386.1	GCA_002766475	100	17828	54452	17828	100	0	0	25863	43690	1	17828	0	32923
NZ_CP027391.1	GCA_002766495	99,995	19603	98724	19602	100	0	1	57330	76932	1	19602	0	36193
NZ_CP027391.1	GCA_002766515	99,995	19617	98724	19616	100	0	1	57316	76932	1	19616	0	36219
NZ_CP027386.1	GCA_002766535	99,984	19082	54452	19080	100	0	3	882	19962	19080	1	0	35218
AP019704.1	GCA_002766575	99,964	22159	92337	22159	100	7	1	16758	38915	22159	1	0	40884
NZ_CP006028.1	GCA_002766595	99,988	16691	87120	16764	100	2	0	51742	68432	16691	1	0	30812
NZ_CP027386.1	GCA_002766615	100	17828	54452	17828	100	0	0	25863	43690	1	17828	0	32923
NZ_CP027391.1	GCA_002766655	99,99	19603	98724	19602	100	1	1	57330	76932	1	19602	0	36188
NC_013369.1	GCA_002766695	100	22344	85167	22344	100	0	0	17192	39535	1	22344	0	41262
AP019704.1	GCA_002766715	99,991	22157	92337	22157	100	2	0	16758	38914	22157	1	0	40906
NZ_CP027391.1	GCA_002766775	99,995	19617	98724	19616	100	0	1	57316	76932	1	19616	0	36219
AP019704.1	GCA_002766795	99,991	22157	92337	22157	100	2	0	16758	38914	22157	1	0	40906
NC_013369.1	GCA_002766835	100	22356	85167	22356	100	0	0	17180	39535	22356	1	0	41284
NZ_CP037944.1	GCA_002766855	99,995	19600	88848	19600	100	1	0	50127	69726	19600	1	0	36189
NZ_CP027391.1	GCA_002766875	99,995	19645	98724	19644	100	0	1	57288	76932	19644	1	0	36271
NZ_CP027391.1	GCA_002766895	99,995	19530	98724	19884	98	0	1	57403	76932	19529	1	0	36058
NZ_CP027391.1	GCA_002766915	99,995	19617	98724	19616	100	0	1	57316	76932	19616	1	0	36219
NZ_CP027391.1	GCA_002767015	99,995	20257	98724	20256	100	0	1	56676	76932	1	20256	0	37401
NZ_CP027391.1	GCA_002767035	99,995	19617	98724	19616	100	0	1	57316	76932	1	19616	0	36219
NC_013369.1	GCA_002767095	100	22356	85167	22356	100	0	0	17180	39535	22356	1	0	41284
NZ_CP027391.1	GCA_002767155	99,995	19617	98724	19616	100	0	1	57316	76932	1	19616	0	36219
NZ_CP027386.1	GCA_002767215	100	17828	54452	17828	100	0	0	25863	43690	1	17828	0	32923
NC_013369.1	GCA_002767235	99,995	19602	85167	19602	100	1	0	47831	67432	1	19602	0	36193
NC_013369.1	GCA_002767275	100	19877	85167	19877	100	0	0	47556	67432	19877	1	0	36706
NZ_CP027386.1	GCA_002767305	100	17828	54452	17828	100	0	0	25863	43690	17828	1	0	32923
NZ_CP027391.1	GCA_002767335	99,995	19617	98724	19616	100	0	1	57316	76932	19616	1	0	36219
NZ_CP027391.1	GCA_002767405	99,995	19645	98724	19644	100	0	1	57288	76932	19644	1	0	36271
NZ_CP027391.1	GCA_002767435	99,99	19645	98724	19644	100	1	1	57288	76932	19644	1	0	36265
NZ_CP027391.1	GCA_002767505	99,995	19617	98724	19616	100	0	1	57316	76932	19616	1	0	36219
NZ_CP037944.1	GCA_002767555	100	19602	88848	19602	100	0	0	50126	69727	19602	1	0	36199
NZ_CP027386.1	GCA_002767595	100	17828	54452	17828	100	0	0	25863	43690	1	17828	0	32923
NC_013369.1	GCA_002767675	100	19601	85167	19601	100	0	0	47831	67431	19601	1	0	36197
NC_013369.1	GCA_002767725	100	19602	85167	19602	100	0	0	47831	67432	1	19602	0	36199
AP019704.1	GCA_002767755	99,991	22191	92337	22191	100	2	0	16724	38914	1	22191	0	40969
NZ_CP027391.1	GCA_002767825	99,995	19645	98724	19644	100	0	1	57288	76932	1	19644	0	36271
NZ_CP027601.1	GCA_002767875	99,995	19369	92590	19369	100	0	1	12624	31991	1	19369	0	35761
CP027321.1	GCA_002767935	99,99	19051	84276	19051	100	2	0	46632	65682	1	19051	0	35170
NZ_CP027386.1	GCA_002768015	99,994	17827	54452	17827	100	1	0	25863	43689	1	17827	0	32915
NC_013369.1	GCA_002768165	100	19602	85167	19602	100	0	0	47831	67432	19602	1	0	36199
NZ_CP027386.1	GCA_002768195	100	17652	54452	17652	100	0	0	25863	43514	1	17652	0	32598
NZ_CP027391.1	GCA_002768285	99,995	19840	98724	19839	100	0	1	57093	76932	1	19839	0	36631
CP027321.1	GCA_002768315	99,963	19035	84276	19034	100	6	1	46631	65665	1	19034	0	35111
NZ_CP027391.1	GCA_002768415	99,99	19617	98724	19616	100	1	1	57316	76932	1	19616	0	36213
CP027321.1	GCA_002768445	99,963	19052	84276	19051	100	6	1	46631	65682	1	19051	0	35142
AP019704.1	GCA_002768515	99,991	22158	92337	22158	100	1	1	16758	38914	22158	1	0	40906
NZ_CP027391.1	GCA_002768535	99,99	19617	98724	19615	100	0	2	57316	76932	1	19615	0	36213
NZ_CP027386.1	GCA_002768555	100	17828	54452	17828	100	0	0	25863	43690	1	17828	0	32923
NZ_CP031923.1	GCA_002768575	99,995	19840	95298	19840	100	1	0	5097	24936	19840	1	0	36633
NC_013369.1	GCA_002768595	100	17755	85167	17828	100	0	0	17180	34934	17828	74	0	32788
NZ_CP027391.1	GCA_002768635	99,99	19613	98724	19612	100	0	2	57316	76927	1	19612	0	36206
NC_013369.1	GCA_002768655	99,996	22356	85167	22356	100	1	0	17180	39535	22356	1	0	41279
CP027321.1	GCA_002768675	99,963	19052	84276	19051	100	6	1	46631	65682	1	19051	0	35142
AP019707.1	GCA_002768695	99,923	22175	91036	22168	100	9	5	16757	38930	22168	1	0	40849
NZ_CP027354.1	GCA_002768715	99,943	15828	57720	15901	100	2	7	17710	33530	1	15828	0	29172
AP019704.1	GCA_002768755	99,995	22157	92337	22157	100	1	0	16758	38914	22157	1	0	40911
NZ_CP006028.1	GCA_002768775	99,988	16691	87120	16764	100	2	0	51742	68432	74	16764	0	30812
NZ_CP027391.1	GCA_002768815	99,99	19840	98724	19839	100	1	1	57093	76932	19839	1	0	36625
NZ_CP027386.1	GCA_002768855	100	17952	54452	17952	100	0	0	25802	43753	1	17952	0	33152
NZ_CP031923.1	GCA_002768875	99,975	19948	95298	20076	99	5	0	63242	83189	20076	129	0	36810
AP019704.1	GCA_002768895	99,969	22474	92337	22467	100	0	1	16699	39172	22467	1	0	41456
NZ_CP031923.1	GCA_002768915	99,976	20644	95298	20645	100	5	0	63242	83885	20644	1	0	38095
AP019707.1	GCA_002768935	99,995	19877	91036	20010	99	1	0	48522	68398	134	20010	0	36701
AP019704.1	GCA_002768955	99,987	22278	92337	22277	100	2	1	16700	38977	22277	1	0	41122
AP019704.1	GCA_002768995	99,964	22476	92337	22469	100	1	1	16698	39173	1	22469	0	41454
NZ_CP037944.1	GCA_002769015	99,995	19716	88848	19716	100	1	0	50069	69784	1	19716	0	36404
AP019704.1	GCA_002769035	99,973	22464	92337	22464	100	6	0	16704	39167	1	22464	0	41451
AP019704.1	GCA_002769055	99,96	22490	92337	22483	100	2	1	16691	39180	1	22483	0	41475
AP019707.1	GCA_002769075	99,983	22893	91036	23144	99	4	0	45517	68409	252	23144	0	42254
NC_013728.1	GCA_002769135	99,954	21580	111481	22574	96	2	1	23011	44582	21580	1	0	39789
NZ_CP031920.1	GCA_002769275	99,949	19429	92498	19425	100	1	6	63788	83211	19425	1	0	35815
AP019709.1	GCA_002769295	99,994	17652	86874	17652	100	1	0	16758	34409	1	17652	0	32592
NZ_CP027354.1	GCA_002769315	99,944	16081	57720	16154	100	2	7	17457	33530	16154	74	0	29639
CP027321.1	GCA_002769335	99,984	19052	84276	19050	100	1	2	46631	65682	19050	1	0	35165

NZ_CP027391.1	GCA_002769355	99,995	19603	98724	19602	100	0	1	57330	76932	1	19602	0	36193
NC_013369.1	GCA_002769375	100	19529	85167	19615	100	0	0	47904	67432	19529	1	0	36064
AP019704.1	GCA_002769415	99,991	22157	92337	22157	100	2	0	16758	38914	22157	1	0	40906
AP019709.1	GCA_002769435	99,994	17110	86874	17110	100	1	0	16758	33867	1	17110	0	31591
NZ_CP031923.1	GCA_002769455	99,99	19602	95298	19602	100	2	0	64225	83826	1	19602	0	36188
NC_013369.1	GCA_002769475	99,996	22356	85167	22356	100	1	0	17180	39535	22356	1	0	41279
NZ_CP027391.1	GCA_002769515	99,99	19617	98724	19616	100	1	1	57316	76932	19616	1	0	36213
NZ_CP027391.1	GCA_002769555	99,995	19603	98724	19602	100	0	1	57330	76932	19602	1	0	36193
NZ_CP028111.1	GCA_002769575	99,925	12053	83211	12374	97	9	0	17441	29493	12374	322	0	22208
NZ_CP027391.1	GCA_002769595	99,995	19602	98724	19601	100	0	1	57330	76931	1	19601	0	36191
NZ_CP027354.1	GCA_002769615	99,944	15978	57720	15978	100	2	7	17457	33427	1	15978	0	29449
NZ_CP027386.1	GCA_002769635	100	17652	54452	17652	100	0	0	25863	43514	1	17652	0	32598
NZ_CP027391.1	GCA_002769695	99,99	19603	98724	19602	100	1	1	57330	76932	1	19602	0	36188
NC_013369.1	GCA_002769715	99,996	22356	85167	22356	100	1	0	17180	39535	22356	1	0	41279
NC_013369.1	GCA_002769775	99,995	19602	85167	19602	100	1	0	47831	67432	19602	1	0	36193
NC_013369.1	GCA_002769795	100	17755	85167	17827	100	0	0	17180	34934	1	17755	0	32788
NC_013369.1	GCA_002769815	100	19602	85167	19602	100	0	0	47831	67432	19602	1	0	36199
AP019709.1	GCA_002769835	99,994	17649	86874	17649	100	1	0	16759	34407	1	17649	0	32587
NC_013369.1	GCA_002769855	100	17755	85167	17828	100	0	0	17180	34934	17828	74	0	32788
NC_013369.1	GCA_002769875	100	22158	85167	22158	100	0	0	17180	39337	22158	1	0	40919
NZ_CP027391.1	GCA_002769895	99,995	19603	98724	19602	100	0	1	57330	76932	1	19602	0	36193
NC_013369.1	GCA_002769915	100	22160	85167	22160	100	0	0	17180	39339	22160	1	0	40922
NZ_CP024055.1	GCA_002769935	99,493	6507	88839	6647	98	21	4	16244	22739	6552	47	0	11823
NC_013728.1	GCA_002769955	99,995	19729	111481	20177	98	1	0	86449	106177	369	20097	0	36428
NC_013369.1	GCA_002769975	100	22356	85167	22356	100	0	0	17180	39535	22356	1	0	41284
NZ_CP027391.1	GCA_002770015	100	17821	98724	17821	100	0	0	22061	39881	1	17821	0	32910
NC_013369.1	GCA_002770035	100	19599	85167	19599	100	0	0	47831	67429	1	19599	0	36193
NZ_CP006028.1	GCA_002770055	99,988	16691	87120	16764	100	2	0	51742	68432	16691	1	0	30812
NZ_CP027391.1	GCA_002770075	99,99	19617	98724	19616	100	1	1	57316	76932	19616	1	0	36213
NZ_CP027391.1	GCA_002770095	99,99	19662	98724	20477	96	1	1	57271	76932	19661	1	0	36297
AP019709.1	GCA_002770115	99,994	16974	86874	16974	100	1	0	17436	34409	16974	1	0	31340
NZ_CP006028.1	GCA_002770135	99,988	16691	87120	16764	100	2	0	51742	68432	16691	1	0	30812
NZ_CP027391.1	GCA_002770155	99,99	19603	98724	19602	100	1	1	57330	76932	19602	1	0	36188
NZ_CP027391.1	GCA_002770175	99,99	19617	98724	19616	100	1	1	57316	76932	19616	1	0	36213
NZ_CP027386.1	GCA_002770195	100	17828	54452	17828	100	0	0	25863	43690	17828	1	0	32923
NC_013369.1	GCA_002770235	100	19602	85167	19602	100	0	0	47831	67432	19602	1	0	36199
NZ_CP027391.1	GCA_002770275	99,985	19603	98724	19602	100	2	1	57330	76932	19602	1	0	36182
NZ_CP027386.1	GCA_002770295	99,994	17828	54452	17828	100	1	0	25863	43690	17828	1	0	32917
NC_013369.1	GCA_002770315	100	22356	85167	22356	100	0	0	17180	39535	22356	1	0	41284
NC_013369.1	GCA_002770335	100	22356	85167	22356	100	0	0	17180	39535	22356	1	0	41284
NZ_CP006028.1	GCA_002770355	99,988	16691	87120	16764	100	2	0	51742	68432	16691	1	0	30812
NZ_CP027601.1	GCA_002770375	99,995	19371	92590	19371	100	0	1	12622	31991	1	19371	0	35765
NZ_CP027583.1	GCA_002770395	99,983	17748	88339	17821	100	3	0	42242	59989	17821	74	0	32758
NZ_CP027391.1	GCA_002770415	99,985	19617	98724	19616	100	2	1	57316	76932	19616	1	0	36208
NZ_CP027354.1	GCA_002770435	99,964	14044	57720	14117	99	1	4	19491	33530	14117	74	0	25904
CP027321.1	GCA_002770455	99,99	19052	84276	19051	100	1	1	46631	65682	1	19051	0	35170
NC_013369.1	GCA_002770495	100	22505	85167	22505	100	0	0	17106	39610	22505	1	0	41559
NZ_CP027354.1	GCA_002770515	99,8	22458	57720	22457	100	37	8	15560	38010	22457	1	0	41249
NC_013369.1	GCA_002770535	100	17827	85167	17974	99	0	0	17108	34934	1	17827	0	32921
NZ_CP038320.1	GCA_002795085	99,996	23579	95338	24136	98	1	0	1	23579	321	23899	0	43537
NC_019041.1	GCA_002795105	100	30752	92077	30753	100	0	0	56802	87553	30752	1	0	56789
NZ_CP040573.1	GCA_002806745	99,997	30928	94177	31045	100	1	0	38261	69188	1	30928	0	57108
NZ_CP038373.1	GCA_002806795	99,996	92726	92725	92725	100	2	2	1	92725	1	92725	0	171200
NZ_CP037942.1	GCA_002810665	99,992	12430	157534	12430	100	1	0	40054	52483	12430	1	0	22949
NZ_CP012501.1	GCA_002810695	99,963	13490	242187	13490	100	5	0	68193	81682	13490	1	0	24884
NZ_CP037942.1	GCA_002810705	99,992	12166	157534	12175	100	1	0	40309	52474	1	12166	0	22461
NZ_CP037942.1	GCA_002810755	99,992	11769	157534	11769	100	1	0	93357	105125	11769	1	0	21728
NZ_CP037942.1	GCA_002810765	99,991	11690	157534	11690	100	1	0	93436	105125	11690	1	0	21582
NZ_CP037942.1	GCA_002810845	99,992	12166	157534	12173	100	1	0	40309	52474	1	12166	0	22461
NZ_CP037942.1	GCA_002810885	99,992	12777	157534	12777	100	1	0	39707	52483	1	12777	0	23590
NZ_CP037942.1	GCA_002810955	99,991	11690	157534	11690	100	1	0	93436	105125	11690	1	0	21582
NZ_CP037942.1	GCA_002834355	99,975	11945	157534	11943	100	1	2	93181	105125	11943	1	0	22040
NZ_CP037942.1	GCA_002834405	99,992	12172	157534	12175	100	1	0	40312	52483	12172	1	0	22472
NZ_CP037942.1	GCA_002834415	99,992	12426	157534	12430	100	1	0	40054	52479	1	12426	0	22942
NZ_CP037942.1	GCA_002834425	99,91	12212	157534	12212	100	11	0	92914	105125	12212	1	0	22491
NZ_CP037942.1	GCA_002834445	99,992	12174	157534	12175	100	1	0	40309	52482	1	12174	0	22476
NZ_CP037942.1	GCA_002834485	99,992	12166	157534	12175	100	1	0	40309	52474	1	12166	0	22461
NZ_CP037942.1	GCA_002834495	99,991	11690	157534	11690	100	1	0	93436	105125	11690	1	0	21582
NZ_CP037942.1	GCA_002835085	99,992	12175	157534	12175	100	1	0	40309	52483	12175	1	0	22478
NZ_CP037942.1	GCA_002835095	99,992	12175	157534	12175	100	1	0	40309	52483	12175	1	0	22478
NZ_CP037942.1	GCA_002835125	99,992	12430	157534	12430	100	1	0	40054	52483	12430	1	0	22949
NZ_CP037942.1	GCA_002835135	99,992	12168	157534	12175	100	1	0	40309	52476	1	12168	0	22465
NZ_CP037942.1	GCA_002835175	99,992	12166	157534	12175	100	1	0	40309	52474	1	12166	0	22461
NZ_CP037942.1	GCA_002837305	99,992	12429	157534	12430	100	1	0	40054	52482	1	12429	0	22947
NZ_CP037942.1	GCA_002837335	99,992	12428	157534	12430	100	1	0	40054	52481	1	12428	0	22945
NZ_CP027334.1	GCA_002837355	99,975	12133	181066	12131	100	1	2	13817	25949	1	12131	0	22388
NZ_CP037942.1	GCA_002837435	99,992	12777	157534	12777	100	1	0	39707	52483	1	12777	0	23590
NZ_CP037942.1	GCA_002837475	99,992	12175	157534	12175	100	1	0	40309	52483	12175	1	0	22478
NZ_CP037942.1	GCA_002844415	99,992	12169	157534	12175	100	1	0	40315	52483	12169	1	0	22467
NZ_CP037942.1	GCA_002844475	99,992	12777	157534	12777	100	1	0	39707	52483	1	12777	0	23590
NC_013354.1	GCA_002915085	99,993	15349	75546	16048	96	1	0	1	15349	15349	1	0	28339
NC_013369.1	GCA_002923435	100	17843	85167	18006	99	0	0	17092	34934	18006	164	0	32950
NZ_CP037944.1	GCA_002923455	100	19638	88848	19638	100	0	0	50104	69741	19638	1	0	36265
NZ_CP037944.1	GCA_002923475	99,99	19624	88848	19624	100	2	0	50104	69727	1	19624	0	36228
AP019707.1	GCA_002923515	99,985	20581	91036	20581	100	3	0	47806	68386	20581	1	0	37990
NC_013369.1	GCA_002923535	100	19738	85167	19738	100	0	0	47695	67432	19738	1	0	36450
NZ_CP027386.1	GCA_002923555	99,983	17828	54452	17828	100	3	0	25863	43690	1	17828	0	32906
AP019709.1	GCA_002923595	99,994	17651	86874	17651	100	1	0	16759	34409	17651	1	0	32590
NZ_CP027386.1	GCA_002923615	99,994	17828	54452	17828	100	1	0	25863	43690	17828	1	0	32917
NZ_CP027391.1	GCA_002923635	99,985	19603	98724	19602	100	2	1	57330	76932	1	19602	0	36182
NZ_CP027391.1	GCA_002923655	99,989	17828	98724	17828	100	2	0	22054	39881	1	17828	0	32912
NZ_CP027391.1	GCA_002923675	99,99	19603	98724	19602	100	1	1	57330	76932	1	19602	0	36188
NC_013369.1	GCA_002923695	99,991	22356	85167	22356	100	2	0	17180	39535	22356	1	0	41273

NC_013369.1	GCA_002923715	100	19602	85167	19602	100	0	0	47831	67432	19602	1	0	36199
AP019709.1	GCA_002923735	99,988	17266	86874	17339	100	2	0	17247	34512	17339	74	0	31874
AP019709.1	GCA_002923815	99,988	17272	86874	17345	100	2	0	17241	34512	1	17272	0	31885
NC_013369.1	GCA_002923855	100	19603	85167	19603	100	0	0	47830	67432	1	19603	0	36200
AP019704.1	GCA_002923875	99,991	22157	92337	22157	100	2	0	16758	38914	22157	1	0	40906
NZ_CP027386.1	GCA_002923895	100	17828	54452	17828	100	0	0	25863	43690	1	17828	0	32923
NZ_CP027391.1	GCA_002923915	99,995	19603	98724	19602	100	0	1	57330	76932	19602	1	0	36193
NC_013728.1	GCA_002923965	99,995	19767	111481	19847	100	1	0	86411	106177	1	19767	0	36498
NZ_CP027391.1	GCA_002923985	99,99	19617	98724	19616	100	1	1	57316	76932	1	19616	0	36213
AP019704.1	GCA_002924005	99,995	22157	92337	22157	100	1	0	16758	38914	22157	1	0	40911
NC_013369.1	GCA_002924025	100	17755	85167	17828	100	0	0	17180	34934	17828	74	0	32788
AP019704.1	GCA_002924045	99,973	22466	92337	22466	100	6	0	16703	39168	1	22466	0	41454
NZ_CP028124.1	GCA_002924065	99,992	13178	83012	13856	95	1	0	47207	60384	679	13856	0	24330
NZ_CP027354.1	GCA_002924105	99,971	13598	57720	13598	100	1	3	19833	33427	13598	1	0	25086
NZ_CP027391.1	GCA_002924145	99,995	19603	98724	19602	100	0	1	57330	76932	1	19602	0	36193
NZ_CP027386.1	GCA_002924165	100	17656	54452	17656	100	0	0	25859	43514	17656	1	0	32605
NZ_CP027386.1	GCA_002924185	100	17828	54452	17828	100	0	0	25863	43690	1	17828	0	32923
NC_013369.1	GCA_002924205	100	22356	85167	22356	100	0	0	17180	39535	22356	1	0	41284
NC_013369.1	GCA_002924225	100	23397	85167	23397	100	0	0	17108	40504	1	23397	0	43207
NZ_CP027601.1	GCA_002924245	99,996	22920	92590	22920	100	0	1	12550	35468	22920	1	0	42318
NZ_CP040108.1	GCA_003027135	99,983	30125	93190	30751	98	5	0	1	30125	500	30624	0	55603
NZ_CP040306.1	GCA_003027175	99,99	38616	95621	38773	100	3	1	14810	53424	158	38773	0	71287
NZ_CP040306.1	GCA_003027185	99,99	38616	95621	38773	100	3	1	14810	53424	158	38773	0	71287
NZ_CP045976.1	GCA_003027235	99,991	31673	94640	31800	100	3	0	2472	34144	31800	128	0	58473
NZ_CP045976.1	GCA_003027335	99,994	31141	94640	31268	100	2	0	3004	34144	31268	128	0	57496
CP027641.1	GCA_003027605	99,873	16472	126957	16471	100	19	2	32868	49338	1	16471	0	30300
NZ_CP027549.1	GCA_003027755	99,967	11978	94116	11978	100	2	2	57596	69571	1	11978	0	22096
NZ_CP027356.1	GCA_003028095	99,85	14703	71714	14703	100	21	1	51765	66466	14703	1	0	27028
NZ_CP024055.1	GCA_003113015	99,359	7023	88839	7315	96	38	3	16396	23414	296	7315	0	12728
NZ_CP027545.1	GCA_003113055	99,893	21444	101089	21909	98	19	4	17710	39150	365	21807	0	39469
NZ_CP027576.1	GCA_003113075	99,871	17071	78427	17912	95	19	2	23290	40357	1	17071	0	31399
NZ_CP027545.1	GCA_003113195	99,89	20843	101089	21706	96	19	4	18313	39152	704	21545	0	38359
NC_013369.1	GCA_003113555	99,989	18907	85167	19204	98	2	0	48751	67657	18907	1	0	34904
NZ_CP027391.1	GCA_003113635	99,995	20048	98724	20052	100	0	1	57105	77152	20052	6	0	37015
NZ_CP027601.1	GCA_003113675	99,995	19819	92590	20642	96	0	1	12402	32219	1	19819	0	36592
NZ_CP027601.1	GCA_003113695	99,99	19851	92590	20051	99	1	1	12402	32251	1	19851	0	36647
AP019704.1	GCA_003113835	99,987	22606	92337	22606	100	3	0	16534	39139	22606	1	0	41731
NZ_CP028111.1	GCA_003113855	99,942	12157	83211	12703	96	7	0	17337	29493	12703	547	0	22412
AP019704.1	GCA_003113875	99,965	22606	92337	22606	100	8	0	16534	39139	22606	1	0	41713
NZ_CP027318.1	GCA_003122805	99,917	15603	81954	15602	100	12	1	55329	70931	15602	1	0	28740
NZ_CP027356.1	GCA_003122955	99,929	16939	71714	16939	100	10	2	16670	33606	16939	1	0	31213
NZ_CP027356.1	GCA_003123155	99,945	27180	71714	28554	95	12	3	44537	71714	28554	1376	0	50106
NZ_CP027583.1	GCA_003123215	99,872	17966	88339	18044	100	15	5	21668	39628	82	18044	0	33043
NZ_CP027436.1	GCA_003123355	99,919	16130	83611	16130	100	13	0	13925	30054	16130	1	0	29715
NZ_CP027356.1	GCA_003123395	99,917	16939	71714	16939	100	12	2	16670	33606	1	16939	0	31202
NZ_CP027356.1	GCA_003123565	99,921	11381	71714	11381	100	8	1	44537	55916	1	11381	0	20966
NZ_CP027576.1	GCA_003293905	100	13374	78427	13724	97	0	0	8153	21526	238	13611	0	24698
NZ_CP027385.1	GCA_003293935	100	8582	118259	8582	100	0	0	67041	75622	8582	1	0	15849
NZ_AP018800.1	GCA_003293975	99,994	15854	81004	15854	100	1	0	24498	40351	1	15854	0	29272
NC_013366.1	GCA_003293985	100	13458	77690	13571	99	0	0	52618	66075	1	13458	0	24853
NZ_AP019763.1	GCA_003294005	100	15835	78470	15835	100	0	0	24517	40351	1	15835	0	29242
NZ_CP027385.1	GCA_003294015	100	8582	118259	8582	100	0	0	67041	75622	8582	1	0	15849
NZ_AP018489.1	GCA_003307155	100	25718	94391	25855	99	0	0	27292	53009	1	25718	0	47493
NZ_CP037942.1	GCA_003340715	99,991	11687	157534	11687	100	1	0	93439	105125	11687	1	0	21577
NZ_CP037942.1	GCA_003340725	99,992	12425	157534	12430	100	1	0	40059	52483	12425	1	0	22940
NZ_CP040310.1	GCA_003347195	99,979	42997	93175	42993	100	4	3	7965	50960	42993	1	0	79346
NZ_CP040310.1	GCA_003347215	99,979	43256	93175	43253	100	4	3	7712	50965	43253	1	0	79824
NZ_CP040310.1	GCA_003347255	99,982	43250	93175	43247	100	4	2	7712	50960	43247	1	0	79820
NZ_CP031914.1	GCA_003359415	99,982	28476	92754	28789	99	3	1	19361	47834	125	28600	0	52556
NZ_CP028651.1	GCA_003359445	99,998	42551	94057	43801	97	0	1	7698	50247	43799	1249	0	78570
NC_011350.1	GCA_003359475	100	20087	94644	20337	99	0	0	17831	37917	20213	127	0	37094
NZ_CP040571.1	GCA_003359495	99,995	43931	92763	43941	100	1	1	23508	67438	1	43930	0	81113
NZ_CP038404.1	GCA_003359515	100	30820	95604	31388	98	0	0	1	30820	30820	1	0	56914
NC_011350.1	GCA_003359555	100	20087	94644	20333	99	0	0	17831	37917	121	20207	0	37094
NC_011350.1	GCA_003359575	100	20087	94644	20337	99	0	0	17831	37917	20213	127	0	37094
NZ_CP044144.1	GCA_003359595	99,965	22536	92690	23204	97	0	3	10481	33008	23204	669	0	41565
NZ_CP017441.1	GCA_003359615	99,998	43325	92729	43324	100	0	1	7696	51020	1	43324	0	79999
NZ_CP040571.1	GCA_003359635	99,998	43818	92763	43820	100	0	1	23629	67446	1	43817	0	80910
NZ_CP017445.1	GCA_003359735	99,979	43826	92726	43820	100	3	2	7746	51571	1	43820	0	80876
NZ_CP038352.1	GCA_003359815	100	30357	92209	31898	95	0	0	1	30357	31395	1039	0	56059
NC_013010.1	GCA_003359855	99,997	29171	94601	29391	99	1	0	1	29171	29171	1	0	53864
NC_013010.1	GCA_003359915	100	29171	94601	29487	99	0	0	1	29171	29171	1	0	53869
NZ_CP008958.1	GCA_003360135	99,997	30683	92076	30722	100	1	0	22654	53336	40	30722	0	56656
NZ_CP038373.1	GCA_003360155	99,984	43786	92725	43786	100	1	1	31862	75641	43786	1	0	80814
NC_017907.1	GCA_003360175	99,997	30835	92728	31447	98	0	1	1	30835	30834	1	0	56935
NC_019041.1	GCA_003360195	99,995	42292	92077	43068	98	1	1	45121	87412	43068	778	0	78086
NZ_CP017441.1	GCA_003360215	99,995	43499	92729	43499	100	2	0	7768	51266	43499	1	0	80317
NZ_CP028651.1	GCA_003360235	99,993	42300	94057	43079	98	0	3	7949	50247	43079	782	0	78094
NZ_CP017441.1	GCA_003360255	99,995	43325	92729	43325	100	2	0	7696	51020	43325	1	0	79997
NZ_CP028653.1	GCA_003360265	99,995	43314	92739	43315	100	0	2	7698	51010	43313	1	0	79973
NZ_CP017441.1	GCA_003360295	99,998	43371	92729	43370	100	0	1	7696	51066	43370	1	0	80084
NZ_CP034805.1	GCA_003360315	99,995	43850	94014	43864	100	2	0	33282	77131	1	43850	0	80965
NZ_CP034805.1	GCA_003360355	99,998	43918	94014	43929	100	0	1	32683	76599	12	43929	0	81094
NZ_CP022052.2	GCA_003360375	99,992	25071	95288	25366	99	2	0	70218	95288	1	25071	0	46287
NZ_CP062161.1	GCA_003360395	99,989	43827	104844	43826	100	4	1	3233	47059	1	43826	0	80904
NZ_CP034805.1	GCA_003360415	100	43065	94014	43065	100	0	0	33282	76346	1	43065	0	79527
NZ_CP008958.1	GCA_003360425	100	38596	92076	39515	98	0	0	14672	53267	39373	778	0	71274
NZ_CP018251.1	GCA_003360435	100	31435	91789	32193	98	0	0	1	31435	749	32183	0	58050
NZ_CP018626.1	GCA_003360475	100	28935	107692	28935	100	0	0	40569	69503	28935	1	0	53433
NZ_CP035546.1	GCA_003360495	99,981	41552	94581	41604	100	7	1	37508	79058	53	41604	0	76686
NZ_CP038359.1	GCA_003360515	99,973	40413	92704	40549	100	4	2	35215	75627	40406	1	0	74561
NZ_CP038406.1	GCA_003360535	99,998	43184	92740	43184	100	1	0	7769	50952	43184	1	0	79741
NZ_CP028608.1	GCA_003360555	100	28960	92745	28960	100	0	0	43405	72364	1	28960	0	53480

NC_017907.1	GCA_003360565	99,997	30763	92728	31332	98	1	0	1	30763	570	31332	0	56804
NZ_CP018626.1	GCA_003360595	100	28900	107692	28900	100	0	0	40604	69503	28900	1	0	53369
NZ_CP028653.1	GCA_003360615	99,998	43329	92739	43328	100	0	1	7696	51024	1	43328	0	80007
NZ_CP017441.1	GCA_003360655	99,998	43321	92729	43324	100	0	1	7700	51020	43320	1	0	79992
NZ_CP017441.1	GCA_003360675	99,998	43326	92729	43326	100	0	1	7696	51020	43326	1	0	80001
NZ_CP028651.1	GCA_003360695	99,997	36686	94057	36812	100	1	0	7696	44381	36812	127	0	67741
NZ_CP028608.1	GCA_003360715	99,997	28950	92745	28950	100	1	0	43405	72354	28950	1	0	53456
NC_017907.1	GCA_003360735	100	31443	92728	32012	98	0	0	1	31443	568	32010	0	58065
NZ_AP018692.1	GCA_003360755	99,998	43780	92722	43780	100	1	0	32021	75800	43780	1	0	80841
NZ_CP038359.1	GCA_003360775	99,979	43765	92704	43759	100	2	2	31863	75627	43758	1	0	80762
NZ_CP012803.1	GCA_003360785	99,997	30772	92739	31337	98	1	0	1	30772	566	31337	0	56820
NZ_AP018692.1	GCA_003360815	99,998	43780	92722	43780	100	1	0	32021	75800	43780	1	0	80841
NC_019041.1	GCA_003360835	100	43542	92077	43542	100	0	0	44788	88329	43542	1	0	80407
NZ_CP028608.1	GCA_003360855	99,997	28956	92745	28956	100	1	0	43405	72360	1	28956	0	53467
NC_019041.1	GCA_003360865	100	43375	92077	43376	100	0	0	44788	88162	1	43375	0	80099
NC_019041.1	GCA_003360895	100	43376	92077	43376	100	0	0	44788	88163	1	43376	0	80101
NZ_CP017250.1	GCA_003360915	99,997	38812	92690	40486	96	0	1	1	38811	38812	1	0	71665
NC_019041.1	GCA_003360955	100	42622	92077	42622	100	0	0	44860	87481	1	42622	0	78709
NZ_CP008958.1	GCA_003360965	99,998	43151	92076	43150	100	0	1	10796	53946	43150	1	0	79678
NC_019041.1	GCA_003360985	99,998	42542	92077	42541	100	0	1	44940	87481	42541	1	0	78553
NZ_CP062161.1	GCA_003361015	99,941	43773	104844	43773	100	6	2	3307	47059	43773	1	0	80672
NZ_CP028686.1	GCA_003361035	99,998	42923	92724	42923	100	1	0	8021	50943	42923	1	0	79259
NC_017907.1	GCA_003361055	99,997	31373	92728	31938	98	1	0	1	31373	566	31938	0	57930
NZ_CP032804.1	GCA_003361065	99,986	42370	91253	43900	97	3	2	9012	51380	42368	1	0	78206
NC_017907.1	GCA_003361095	100	31371	92728	31936	98	0	0	1	31371	566	31936	0	57932
NZ_CP028653.1	GCA_003361115	99,998	43439	92739	43438	100	0	1	7586	51024	1	43438	0	80210
NZ_CP008958.1	GCA_003361135	100	42671	92076	42671	100	0	0	10724	53394	42671	1	0	78799
NZ_CP008958.1	GCA_003361155	99,998	43151	92076	43151	100	1	0	10796	53946	43151	1	0	79680
NC_017907.1	GCA_003361195	100	31373	92728	31938	98	0	0	1	31373	566	31938	0	57936
NZ_CP038341.1	GCA_003361215	100	30763	92839	31328	98	0	0	1	30763	566	31328	0	56809
NZ_CP038341.1	GCA_003361235	100	30762	92839	31639	97	0	0	1	30762	877	31638	0	56807
NZ_CP038341.1	GCA_003361255	100	30763	92839	31656	97	0	0	1	30763	894	31656	0	56809
NC_017907.1	GCA_003361275	100	31373	92728	31938	98	0	0	1	31373	566	31938	0	57936
NZ_CP038293.1	GCA_003361295	99,997	32659	95611	32659	100	1	0	31800	64458	32659	1	0	60305
NZ_CP038341.1	GCA_003361305	100	30762	92839	31336	98	0	0	1	30762	574	31335	0	56807
NZ_CP038323.1	GCA_003361355	100	36561	92701	36561	100	0	0	39405	75965	36561	1	0	67516
NZ_CP008958.1	GCA_003361375	100	42341	92076	43046	98	0	0	10927	53267	43046	706	0	78190
NZ_CP008958.1	GCA_003361395	99,995	42219	92076	42924	98	2	0	11049	53267	42924	706	0	77953
NC_017907.1	GCA_003361405	100	30763	92728	31330	98	0	0	1	30763	568	31330	0	56809
NC_019041.1	GCA_003361435	99,998	42542	92077	42542	100	1	0	44940	87481	42542	1	0	78555
NZ_CP062161.1	GCA_003361455	99,896	38523	104844	38575	100	10	6	3843	42337	38575	55	0	70890
NZ_CP008958.1	GCA_003361465	100	42671	92076	42671	100	0	0	10724	53394	42671	1	0	78799
NC_017907.1	GCA_003361495	100	31371	92728	31937	98	0	0	1	31371	567	31937	0	57932
NZ_CP038289.1	GCA_003361515	99,994	32492	96920	32492	100	2	0	31947	64438	32492	1	0	59991
NZ_CP028606.1	GCA_003361535	99,978	32513	95648	32513	100	1	1	54594	87100	32513	1	0	59996
NZ_CP017441.1	GCA_003361555	100	43935	92729	43935	100	0	0	7696	51630	1	43935	0	81133
NZ_CP032804.1	GCA_003361575	99,986	42444	91253	44152	96	3	2	9012	51452	42444	1	0	78343
NZ_CP016626.1	GCA_003361585	100	32497	95598	32497	100	0	0	18098	50594	32497	1	0	60011
NZ_CP034805.1	GCA_003361615	99,995	43785	94014	43785	100	1	1	32744	76527	1	43785	0	80843
NZ_CP028686.1	GCA_003361635	99,998	42923	92724	42922	100	0	1	8021	50943	42922	1	0	79257
NZ_CP034805.1	GCA_003361655	99,998	43174	94014	43174	100	1	0	33354	76527	1	43174	0	79722
NC_017907.1	GCA_003361675	100	30762	92728	31328	98	0	0	1	30762	566	31327	0	56807
NZ_CP062161.1	GCA_003361695	99,981	43219	104844	43217	100	4	4	3843	47059	1	43217	0	79763
NZ_CP017441.1	GCA_003361705	99,998	43058	92729	43057	100	0	1	7949	51006	1	43057	0	79506
NC_017907.1	GCA_003361735	100	30763	92728	31381	98	0	0	1	30763	30763	1	0	56809
NZ_CP062161.1	GCA_003361755	99,947	43235	104844	43235	100	5	2	3843	47059	1	43235	0	79696
NZ_CP034805.1	GCA_003361775	100	43675	94014	43675	100	0	0	32672	76346	1	43675	0	80653
NC_017907.1	GCA_003361795	100	30763	92728	31326	98	0	0	1	30763	564	31326	0	56809
NC_017907.1	GCA_003361815	100	30763	92728	31334	98	0	0	1	30763	572	31334	0	56809
NZ_CP038289.1	GCA_003361825	100	31207	96920	31852	98	0	0	1	31207	646	31852	0	57629
NC_017907.1	GCA_003361855	100	30762	92728	31325	98	0	0	1	30762	563	31324	0	56807
NZ_CP008958.1	GCA_003361875	99,995	42473	92076	43178	98	1	1	10796	53267	43178	706	0	78420
NC_017907.1	GCA_003361885	100	30762	92728	31328	98	0	0	1	30762	566	31327	0	56807
NC_017907.1	GCA_003361915	100	30762	92728	31320	98	0	0	1	30762	558	31319	0	56807
NC_017907.1	GCA_003361935	99,997	30762	92728	31514	98	1	0	1	30762	752	31513	0	56802
NZ_CP016626.1	GCA_003361955	100	20333	95598	20401	100	0	0	17577	37909	1	20333	0	37549
NC_013010.1	GCA_003361965	100	29402	94601	29777	99	0	0	1	29402	29402	1	0	54296
NC_019041.1	GCA_003361995	99,998	42544	92077	43933	97	0	1	44870	87412	43931	1388	0	78557
NZ_CP038341.1	GCA_003362015	100	30763	92839	31328	98	0	0	1	30763	566	31328	0	56809
NZ_CP017445.1	GCA_003362035	99,995	43172	92726	43171	100	1	1	7768	50939	43171	1	0	79711
NZ_CP034805.1	GCA_003362045	99,995	43175	94014	43175	100	1	1	33354	76527	1	43175	0	79717
NZ_CP017445.1	GCA_003362075	99,988	43172	92726	43168	100	1	1	7768	50939	43168	1	0	79693
NZ_CP016626.1	GCA_003362095	100	32497	95598	32497	100	0	0	18098	50594	1	32497	0	60011
NZ_CP017445.1	GCA_003362115	99,977	43639	92726	43634	100	5	1	7768	51406	43634	1	0	80526
NZ_CP028653.1	GCA_003362135	100	43076	92739	43076	100	0	0	7949	51024	1	43076	0	79547
NC_017907.1	GCA_003362155	100	31243	92728	31808	98	0	0	1	31243	566	31808	0	57695
NZ_CP008958.1	GCA_003362175	99,998	42472	92076	43176	98	0	1	10796	53267	43176	706	0	78424
NZ_CP038289.1	GCA_003362195	99,993	30759	96920	31631	97	2	0	1	30759	867	31625	0	56791
NZ_CP062161.1	GCA_003362215	99,944	43236	104844	43236	100	5	2	3843	47059	1	43236	0	79691
NC_017907.1	GCA_003362235	100	30763	92728	31336	98	0	0	1	30763	574	31336	0	56809
NZ_CP016626.1	GCA_003362255	100	19812	95598	19880	100	0	0	18098	37909	1	19812	0	36586
NZ_CP016626.1	GCA_003362265	100	32495	95598	32497	100	0	0	18098	50592	1	32495	0	60008
NZ_CP034805.1	GCA_003362295	100	43318	94014	43318	100	0	0	33210	76527	1	43318	0	79994
NZ_CP038341.1	GCA_003362335	100	30762	92839	31627	97	0	0	1	30762	865	31626	0	56807
NZ_CP038341.1	GCA_003362355	100	30763	92839	31347	98	0	0	1	30763	585	31347	0	56809
NC_017907.1	GCA_003362375	100	30763	92728	31322	98	0	0	1	30763	560	31322	0	56809
NZ_CP044146.1	GCA_003362395	99,996	23176	92722	23176	100	1	0	58659	81834	1	23176	0	42793
NC_017907.1	GCA_003362415	99,997	30762	92728	31328	98	1	0	1	30762	566	31327	0	56802
NZ_CP028701.1	GCA_003362425	99,998	43937	92738	43937	100	1	0	7696	51632	1	43937	0	81131
NZ_CP016626.1	GCA_003362455	100	32497	95598	32497	100	0	0	18098	50594	32497	1	0	60011
NZ_CP038341.1	GCA_003362475	100	30821	92839	31746	97	0	0	1	30821	926	31746	0	56916
NZ_CP016626.1	GCA_003362495	99,995	19812	95598	19880	100	1	0	18098	37909	1	19812	0	36581
NZ_CP008958.1	GCA_003362505	99,995	42544	92076	43320	98	1	1	10724	53267	43320	778	0	78552

NC_017907.1	GCA_003362535	100	30763	92728	31335	98	0	0	1	30763	573	31335	0	56809
NZ_CP017445.1	GCA_003362555	99,981	43172	92726	43167	100	3	1	7768	50939	43167	1	0	79674
NZ_CP062161.1	GCA_003362575	99,949	43233	104844	43233	100	6	2	3843	47059	1	43233	0	79700
NZ_CP038341.1	GCA_003362595	99,993	30763	92839	31620	97	2	0	1	30763	858	31620	0	56798
NZ_CP017445.1	GCA_003362615	99,979	43172	92726	43167	100	4	1	7768	50939	43167	1	0	79669
NZ_CP038343.1	GCA_003362635	99,991	43198	92743	43198	100	2	1	7767	50962	43198	1	0	79748
NZ_CP028653.1	GCA_003362645	99,998	43329	92739	43328	100	0	1	7696	51024	43328	1	0	80007
NZ_CP038293.1	GCA_003362665	100	32495	95611	32495	100	0	0	31950	64444	32495	1	0	60008
NZ_CP040308.1	GCA_003362695	99,978	32523	95642	32523	100	1	1	54568	87084	32523	1	0	60015
NC_017907.1	GCA_003362715	100	30763	92728	31321	98	0	0	1	30763	559	31321	0	56809
NZ_CP027385.1	GCA_003418305	100	9932	118259	9932	100	0	0	66490	76421	1	9932	0	18342
NC_011350.1	GCA_003418635	100	18181	94644	18181	100	0	0	41013	59193	1	18181	0	33575
NZ_CP022408.1	GCA_003419045	100	21440	81950	21440	100	0	0	51542	72981	1	21440	0	39593
NZ_CP006263.1	GCA_003591355	99,966	8818	98066	8818	100	3	0	84165	92982	8818	1	0	16268
NZ_CP027339.1	GCA_003735545	99,945	16379	92644	16379	100	1	8	28573	44943	16379	1	0	30190
NZ_CP024480.1	GCA_003735965	99,997	36728	77062	36728	100	1	0	20709	57436	1	36728	0	67819
NZ_CP028651.1	GCA_003736145	99,99	30598	94057	30801	99	2	1	19651	50247	45	30642	0	56486
NC_013366.1	GCA_003736345	100	10931	77690	10931	100	0	0	54761	65691	10931	1	0	20186
NZ_CP027339.1	GCA_003736365	99,945	16378	92644	16378	100	1	8	28573	44942	16378	1	0	30188
NC_013366.1	GCA_003736465	100	12994	77690	12994	100	0	0	52698	65691	12994	1	0	23996
NC_013366.1	GCA_003736505	100	12994	77690	12994	100	0	0	52698	65691	12994	1	0	23996
NC_017907.1	GCA_003737535	100	30692	92728	30975	99	0	0	1	30692	284	30975	0	56678
NC_013010.1	GCA_003737825	99,997	29093	94601	29387	99	1	0	1	29093	295	29387	0	53720
NZ_CP027354.1	GCA_003738305	99,861	12964	57720	13172	98	9	9	15856	28811	12963	1	0	23832
NZ_CP015816.1	GCA_003738445	100	16598	95910	16598	100	0	0	61792	78389	1	16598	0	30651
NZ_CP046526.1	GCA_003738485	99,964	19599	98304	19620	100	1	1	19692	39284	19620	22	0	36149
NC_013010.1	GCA_003739485	100	29093	94601	29441	99	0	0	1	29093	349	29441	0	53725
NC_017907.1	GCA_003739505	100	30691	92728	31047	99	0	0	1	30691	357	31047	0	56676
NZ_CP027385.1	GCA_003739585	100	8623	118259	8623	100	0	0	66921	75543	1	8623	0	15924
NC_013010.1	GCA_003740485	100	29093	94601	29417	99	0	0	1	29093	325	29417	0	53725
NZ_CP024480.1	GCA_003740545	100	36729	77062	36729	100	0	0	20709	57437	1	36729	0	67826
NZ_CP034795.1	GCA_003740565	100	31488	94987	31488	100	0	0	55713	87200	31488	1	0	58148
NC_013010.1	GCA_003740605	100	29093	94601	29716	98	0	0	1	29093	29093	1	0	53725
NZ_CP024480.1	GCA_003740725	100	18178	77062	18178	100	0	0	20709	38886	1	18178	0	33569
NC_017907.1	GCA_003740765	99,997	30692	92728	31223	98	1	0	1	30692	532	31223	0	56672
NC_013366.1	GCA_003740925	99,993	13701	77690	13701	100	1	0	52698	66398	13701	1	0	25296
NZ_CP027588.1	GCA_003740945	99,989	28150	58109	29181	96	2	1	1	28149	28150	1	0	51965
AP019707.1	GCA_003741045	99,952	20765	91036	20833	100	9	1	47499	68263	70	20833	0	38289
NZ_CP038362.1	GCA_003741065	99,984	58022	95928	58016	100	3	1	34642	92663	58016	1	0	107100
NZ_CP018251.1	GCA_003741085	100	30691	91789	31222	98	0	0	1	30691	532	31222	0	56676
NZ_CP038424.1	GCA_003741105	99,987	15775	96931	16493	96	2	0	1	15775	462	16236	0	29120
NZ_CP028651.1	GCA_003741125	100	33591	94057	33591	100	0	0	53496	87086	33591	1	0	62031
CP027321.1	GCA_003741205	99,984	18902	84276	18900	100	1	2	46706	65607	18900	1	0	34888
NZ_CP038362.1	GCA_003741235	99,988	58021	95928	58014	100	0	1	34643	92663	58014	1	0	107100
NC_017907.1	GCA_003741385	99,997	30692	92728	31209	98	1	0	1	30692	518	31209	0	56672
NZ_CP061760.1	GCA_003741425	99,965	59189	172576	59189	100	20	1	64938	124125	59189	1	0	109200
NZ_CP015816.1	GCA_003742705	99,99	20622	95910	20621	100	1	1	30830	51451	20621	1	0	38069
AP019707.1	GCA_003742745	99,99	19731	91036	19731	100	2	0	48812	68542	1	19731	0	36426
NZ_CP024480.1	GCA_003742885	100	36728	77062	36728	100	0	0	20709	57436	1	36728	0	67824
NZ_CP017445.1	GCA_003742965	99,981	42791	92726	42785	100	2	1	8077	50867	42785	1	0	78971
NC_013010.1	GCA_003746545	99,98	30021	94601	30367	99	4	1	1	30021	349	30367	0	55404
NC_013366.1	GCA_003747865	100	10913	77690	10913	100	0	0	54779	65691	10913	1	0	20153
NZ_CP024480.1	GCA_003752795	100	31647	77062	31647	100	0	0	25790	57436	1	31647	0	58442
NZ_CP038424.1	GCA_003752825	99,987	15775	96931	16345	97	2	0	1	15775	327	16101	0	29120
NZ_CP035546.1	GCA_003753605	99,998	45834	94581	45834	100	0	1	33329	79161	45834	1	0	84633
NZ_CP024480.1	GCA_003753865	100	18699	77062	18699	100	0	0	38739	57437	18699	1	0	34531
NZ_CP027385.1	GCA_003753925	99,977	8623	118259	8623	100	2	0	66921	75543	1	8623	0	15913
NZ_CP027339.1	GCA_003753985	99,95	15895	92644	15895	100	0	8	29057	44943	15895	1	0	29301
NZ_CP028595.1	GCA_003754205	99,994	31211	95624	31211	100	2	0	22108	53318	1	31211	0	57625
NC_011350.1	GCA_003754245	100	20749	94644	20749	100	0	0	42549	63297	1	20749	0	38317
NC_013010.1	GCA_003754625	100	29093	94601	29405	99	0	0	1	29093	313	29405	0	53725
NZ_CP027598.1	GCA_003754645	99,983	29167	74505	29167	100	3	2	4985	34149	1	29167	0	53832
NZ_CP027588.1	GCA_003754845	99,991	10689	58109	10689	100	1	0	44352	55040	1	10689	0	19734
NZ_CP038413.1	GCA_003754925	99,945	45733	121214	45721	100	4	2	13302	59025	1	45721	0	84295
NZ_CP028111.1	GCA_003755365	99,967	12120	83211	12360	98	4	0	17374	29493	1	12120	0	22360
NZ_CP035546.1	GCA_003755565	99,998	45834	94581	45834	100	0	1	33329	79161	45834	1	0	84633
NZ_CP027339.1	GCA_003755705	99,951	16377	92644	16377	100	0	8	28575	44943	16377	1	0	30191
NC_013010.1	GCA_003755825	100	29093	94601	29721	98	0	0	1	29093	29093	1	0	53725
NC_013010.1	GCA_003755885	100	29093	94601	29375	99	0	0	1	29093	283	29375	0	53725
NC_011350.1	GCA_003756025	100	19840	94644	19840	100	0	0	43458	63297	1	19840	0	36638
NC_013010.1	GCA_003756385	100	29092	94601	29712	98	0	0	1	29092	29092	1	0	53723
NZ_CP034795.1	GCA_003756505	100	31489	94987	31489	100	0	0	55713	87201	31489	1	0	58150
NZ_CP027389.1	GCA_003756525	100	20093	68062	20093	100	0	0	26698	46790	20093	1	0	37105
NZ_CP027339.1	GCA_003756685	99,951	16376	92644	16376	100	0	8	28576	44943	16376	1	0	30190
NZ_CP027339.1	GCA_003757005	99,951	16379	92644	16379	100	0	8	28573	44943	16379	1	0	30195
NZ_CP038382.1	GCA_003757205	99,967	30336	91912	31353	97	10	0	1	30336	685	31020	0	55965
NZ_CP038382.1	GCA_003757305	99,97	30336	91912	31353	97	9	0	1	30336	685	31020	0	55971
NC_017907.1	GCA_003757525	99,997	30691	92728	31215	98	1	0	1	30691	525	31215	0	56671
NC_013369.1	GCA_003757565	99,995	22205	85167	22205	100	1	0	17256	39460	1	22205	0	41000
CP014753.1	GCA_003757625	100	15235	72996	15810	96	0	0	1	15235	576	15810	0	28134
NZ_CP017445.1	GCA_003757785	99,995	42793	92726	42793	100	1	1	8077	50868	42793	1	0	79011
NC_017907.1	GCA_003757805	100	30691	92728	31123	99	0	0	1	30691	433	31123	0	56676
NC_013010.1	GCA_003757825	99,997	29093	94601	29728	98	1	0	1	29093	29093	1	0	53720
NZ_CP027343.1	GCA_003758425	99,885	11281	131410	11280	100	11	2	53832	65111	11280	1	0	20759
NZ_CP027339.1	GCA_003758605	99,944	15956	92644	15956	100	1	8	28995	44942	15956	1	0	29409
NC_017907.1	GCA_003758665	100	30691	92728	31045	99	0	0	1	30691	355	31045	0	56676
AP019707.1	GCA_003759245	99,995	19450	91036	19450	100	1	0	48813	68262	1	19450	0	35912
AP019704.1	GCA_003759285	99,991	22006	92337	22005	100	1	1	16834	38839	1	22005	0	40625
NZ_CP034805.1	GCA_003759535	99,998	42793	94014	42793	100	1	0	33426	76218	1	42793	0	79019
NZ_CP038314.1	GCA_003759605	100	15687	97630	16143	97	0	0	1	15687	265	15951	0	28969
AP019709.1	GCA_003759805	99,994	17019	86874	17019	100	1	0	46517	63535	17019	1	0	31423
NZ_CP027339.1	GCA_003760045	99,951	16189	92644	16189	100	0	8	28763	44943	16189	1	0	29844
NZ_CP024480.1	GCA_003760195	99,997	34800	77062	34800	100	1	0	20709	55508	1	34800	0	64258

NZ_CP038424.1	GCA_003760205	100	15775	96931	16399	96	0	0	1	15775	398	16172	0	29132
NZ_CP037944.1	GCA_003760405	99,995	19221	88848	19221	100	1	0	50431	69651	19221	1	0	35490
NZ_CP022408.1	GCA_003760785	100	15415	81950	15415	100	0	0	51797	67211	15415	1	0	28467
NZ_CP041624.1	GCA_003760905	99,903	30908	95081	30881	100	3	2	31968	62875	30881	1	0	56885
NC_013369.1	GCA_003760945	100	19452	85167	19452	100	0	0	47906	67357	1	19452	0	35922
AP019707.1	GCA_003760985	99,995	19453	91036	19453	100	0	1	48813	68264	1	19453	0	35916
NC_013366.1	GCA_003761025	100	12993	77690	12993	100	0	0	52699	65691	12993	1	0	23994
NZ_CP027339.1	GCA_003761465	99,938	16018	92644	16018	100	2	8	28934	44943	16018	1	0	29517
NZ_CP027451.1	GCA_003761485	99,986	42014	173649	42014	100	4	2	42391	84402	1	42014	0	77551
NZ_CP038289.1	GCA_003762945	100	30693	96920	31101	99	0	0	1	30693	409	31101	0	56680
CP043016.1	GCA_003763345	99,93	22752	91446	23274	98	3	2	8909	31653	22746	1	0	41914
NC_013010.1	GCA_003763725	100	29093	94601	29441	99	0	0	1	29093	349	29441	0	53725
NZ_CP027339.1	GCA_003763965	99,951	16379	92644	16379	100	0	8	28573	44943	16379	1	0	30195
AP019704.1	GCA_003764045	100	22204	92337	22204	100	0	0	16834	39037	1	22204	0	41004
NZ_CP027385.1	GCA_003764205	99,989	9094	118259	9094	100	1	0	66921	76014	1	9094	0	16789
NZ_CP024480.1	GCA_003764265	100	36728	77062	36728	100	0	0	20709	57436	1	36728	0	67824
AP019707.1	GCA_003764345	99,99	19473	91036	19471	100	0	1	48790	68262	1	19471	0	35948
NZ_CP038320.1	GCA_003764385	99,996	23579	95338	24235	97	1	0	1	23579	515	24093	0	43537
NZ_CP024480.1	GCA_003764505	100	18641	77062	18641	100	0	0	38796	57436	18641	1	0	34424
NZ_CP024480.1	GCA_003765465	100	35824	77062	35824	100	0	0	21614	57437	1	35824	0	66155
NZ_CP027326.1	GCA_003765485	99,986	14768	74671	14768	100	1	1	8809	23575	1	14768	0	27259
NC_013010.1	GCA_003765865	99,997	29093	94601	29722	98	1	0	1	29093	29093	1	0	53720
NZ_CP038424.1	GCA_003765945	99,987	15775	96931	16540	95	2	0	1	15775	516	16290	0	29120
NC_013010.1	GCA_003766005	100	30049	94601	30162	100	0	0	1	30049	114	30162	0	55491
NZ_CP024480.1	GCA_003766025	99,995	18653	77062	18653	100	1	0	38784	57436	18653	1	0	34441
NZ_CP034795.1	GCA_003766465	100	31488	94987	31488	100	0	0	55713	87200	31488	1	0	58148
NZ_CP024480.1	GCA_003766745	99,995	18718	77062	18718	100	1	0	38719	57436	18718	1	0	34561
NZ_CP035546.1	GCA_003766785	99,998	45834	94581	45834	100	0	1	33329	79161	45834	1	0	84633
NZ_AP018800.1	GCA_003767085	100	9652	81004	9652	100	0	0	11212	20863	1	9652	0	17824
CP012495.1	GCA_003767145	100	7624	52297	7624	100	0	0	15996	23619	7624	1	0	14079
NZ_CP015816.1	GCA_003767395	99,997	29234	95910	29234	100	0	1	31239	60471	1	29234	0	53978
NC_013366.1	GCA_003767865	100	12994	77690	12994	100	0	0	52698	65691	12994	1	0	23996
CP051632.1	GCA_003767945	100	15321	81965	15321	100	0	0	20940	36260	15321	1	0	28293
NZ_CP027553.1	GCA_003768445	99,99	9683	67055	9683	100	0	1	11490	21171	1	9683	0	17874
NZ_CP051657.1	GCA_003768565	100	16664	74390	16664	100	0	0	19242	35905	1	16664	0	30773
NZ_CP018251.1	GCA_003768605	100	30691	91789	31223	98	0	0	1	30691	533	31223	0	56676
NZ_CP027339.1	GCA_003768825	99,951	16379	92644	16379	100	0	8	28573	44943	16379	1	0	30195
NZ_CP038341.1	GCA_003768925	100	30693	92839	31510	97	0	0	1	30693	30693	1	0	56680
NZ_CP031352.1	GCA_003769285	100	22218	86711	22218	100	0	0	16665	38882	1	22218	0	41029
NZ_CP027385.1	GCA_003769865	100	8795	118259	8795	100	0	0	66921	75715	1	8795	0	16242
NC_013369.1	GCA_003770085	100	22205	85167	22205	100	0	0	17256	39460	1	22205	0	41005
NZ_CP038371.1	GCA_003770105	99,996	45639	92990	45638	100	1	1	32009	77647	45638	1	0	84267
NZ_CP038320.1	GCA_003770165	99,996	23579	95338	24337	97	1	0	1	23579	522	24100	0	43537
NZ_CP022408.1	GCA_003770445	100	21056	81950	21056	100	0	0	51797	72852	21056	1	0	38884
NC_013369.1	GCA_003770525	99,995	19452	85167	19452	100	1	0	47906	67357	1	19452	0	35916
AP019704.1	GCA_003770545	99,995	22006	92337	22006	100	1	0	16834	38839	1	22006	0	40632
NC_017907.1	GCA_003770605	100	30692	92728	31216	98	0	0	1	30692	525	31216	0	56678
NZ_CP024480.1	GCA_003773905	100	18612	77062	18612	100	0	0	38826	57437	18612	1	0	34370
AP019707.1	GCA_003774025	99,995	19450	91036	19450	100	1	0	48812	68261	1	19450	0	35912
NZ_CP035546.1	GCA_003776525	100	28910	94581	29351	98	0	0	1	28910	442	29351	0	53387
NZ_CP024480.1	GCA_003777385	99,995	36729	77062	36729	100	2	0	20709	57437	1	36729	0	67815
NC_013010.1	GCA_003777565	100	29093	94601	29441	99	0	0	1	29093	349	29441	0	53725
NC_013369.1	GCA_003782205	100	19175	85167	19175	100	0	0	48183	67357	19175	1	0	35410
NZ_CP034793.1	GCA_003784565	99,998	45033	95421	45858	98	1	0	50389	95421	1	45033	0	83155
NC_017907.1	GCA_003787205	100	30691	92728	31222	98	0	0	1	30691	532	31222	0	56676
NZ_CP017250.1	GCA_003787245	99,982	27046	92690	27920	97	3	1	1	27046	877	27920	0	49916
NZ_CP035546.1	GCA_003787565	100	28910	94581	29417	98	0	0	1	28910	508	29417	0	53387
NZ_CP015022.1	GCA_003787645	100	31237	95170	31237	100	0	0	55682	86918	1	31237	0	57684
NC_017907.1	GCA_003787825	100	30691	92728	31222	98	0	0	1	30691	532	31222	0	56676
NZ_CP022408.1	GCA_003864655	100	20937	81950	20937	100	0	0	51669	72605	1	20937	0	38664
NZ_CP022408.1	GCA_003864675	100	20937	81950	20937	100	0	0	51669	72605	20937	1	0	38664
NZ_CP022408.1	GCA_003864695	100	20937	81950	20937	100	0	0	51669	72605	1	20937	0	38664
NZ_CP022408.1	GCA_003864835	100	20937	81950	20937	100	0	0	51669	72605	20937	1	0	38664
NZ_CP027456.1	GCA_003864855	100	15671	79682	15671	100	0	0	48794	64464	15671	1	0	28939
NZ_CP022408.1	GCA_003864875	100	20936	81950	20936	100	0	0	51669	72604	1	20936	0	38662
NZ_CP022408.1	GCA_003864895	100	20937	81950	20937	100	0	0	51669	72605	1	20937	0	38664
NZ_CP027386.1	GCA_003876425	99,994	17677	54452	17677	100	1	0	25939	43615	1	17677	0	32638
CP027674.1	GCA_003876975	99,865	11139	133420	11135	100	5	5	116349	127481	1	11135	0	20478
NZ_CP018244.1	GCA_003878795	100	30691	92522	31408	98	0	0	1	30691	30691	1	0	56676
NZ_CP023674.1	GCA_003878855	99,61	8979	87524	9294	97	23	7	46539	55506	9294	317	0	16377
CP043012.1	GCA_003878895	99,997	31281	103252	31281	100	0	1	6909	38189	31281	2	0	57758
NZ_CP032792.1	GCA_003879015	99,997	31223	90310	31223	100	1	0	19155	50377	1	31223	0	57653
NZ_CP018244.1	GCA_003879075	99,997	30692	92522	31219	98	1	0	1	30692	528	31219	0	56672
NZ_CP038348.1	GCA_003879155	99,997	31223	89216	31223	100	1	0	22047	53269	1	31223	0	57653
NZ_CP018244.1	GCA_003879255	99,997	30707	92522	31237	98	1	0	1	30707	531	31237	0	56700
CP027674.1	GCA_003879515	99,942	26051	133420	26047	100	10	5	104077	130126	1	26047	0	48019
NZ_CP038422.1	GCA_003879815	100	30707	94959	31238	98	0	0	1	30707	532	31238	0	56706
NZ_CP038314.1	GCA_003880175	99,954	19543	97630	20566	95	2	1	28374	47916	1	19536	0	36032
NZ_CP027385.1	GCA_003880615	99,976	8426	118259	8426	100	2	0	67119	75544	1	8426	0	15549
AP019707.1	GCA_003880675	99,99	19269	91036	19269	100	2	0	48675	67943	1	19269	0	35573
NZ_CP040306.1	GCA_003880875	99,99	38487	95621	38516	100	3	1	14810	53295	30	38516	0	71049
NZ_CP018244.1	GCA_003880935	99,997	30691	92522	31206	98	1	0	1	30691	516	31206	0	56671
CP043012.1	GCA_003881135	99,997	31281	103252	31281	100	0	1	6909	38189	1	31280	0	57758
NZ_CP018244.1	GCA_003881175	100	30691	92522	31398	98	0	0	1	30691	30691	1	0	56676
NZ_CP038422.1	GCA_003881435	99,975	31382	94959	31382	100	1	1	32007	63381	31382	1	0	57900
CP027641.1	GCA_003881875	98,879	13652	126957	13647	100	136	11	55705	69344	1	13647	0	24347
NZ_CP040317.1	GCA_003881915	99,988	42784	93331	42784	100	5	0	8066	50849	1	42784	0	78980
CP043012.1	GCA_003881995	99,997	31281	103252	31281	100	0	1	6909	38189	31281	2	0	57758
NC_013010.1	GCA_003882215	100	29092	94601	29716	98	0	0	1	29092	29092	1	0	53723
CP027641.1	GCA_003882255	99,325	12741	126957	12739	100	77	5	55509	68242	1	12739	0	23043
CP027581.1	GCA_003882335	99,965	14169	118822	14169	100	5	0	34156	48324	1	14169	0	26138
CP043012.1	GCA_003882575	99,994	31281	103252	31281	100	1	1	6909	38189	1	31280	0	57753
NZ_CP018238.1	GCA_003882595	99,997	30692	91420	31277	98	0	1	1	30692	587	31277	0	56671

CP027674.1	GCA_003882735	99,892	22153	133420	22129	100	0	1	46905	69057	1	22129	0	40754
NC_011350.1	GCA_003882975	99,89	31774	94644	31746	100	1	2	17836	49603	31746	1	0	58451
NZ_CP027354.1	GCA_003883195	99,852	17538	57720	17524	100	4	9	15859	33388	1	17524	0	32223
NZ_CP027389.1	GCA_003883855	99,964	16700	68062	16700	100	3	1	26713	43409	16700	1	0	30803
NZ_CP038341.1	GCA_003884015	99,993	30693	92839	31491	97	2	0	1	30693	30693	1	0	56669
CP027674.1	GCA_003884075	98,441	10389	133420	10385	100	154	5	104231	114615	1	10385	0	18281
CP027641.1	GCA_003884135	99,319	12485	126957	12483	100	76	5	55509	67986	1	12483	0	22576
CP027641.1	GCA_003884155	98,904	14238	126957	14233	100	140	11	55632	69858	1	14233	0	25414
CP058232.1	GCA_003884455	99,995	41798	94605	41796	100	0	1	46414	88211	41796	1	0	77174
NZ_CP018244.1	GCA_003884535	99,997	30691	92522	31403	98	0	1	1	30691	30690	1	0	56669
CP043023.1	GCA_003884915	99,749	24735	103546	24930	99	10	39	28140	52830	136	24862	0	45286
NZ_CP038289.1	GCA_003885435	99,99	30421	96920	30951	98	3	0	1	30421	531	30951	0	56161
CP027674.1	GCA_003885595	99,931	26051	133420	26047	100	13	5	104077	130126	1	26047	0	48002
CP043012.1	GCA_003887215	99,997	31281	103252	31281	100	0	1	6909	38189	31281	2	0	57758
NZ_CP018238.1	GCA_003887335	99,997	30693	91420	31284	98	0	1	1	30693	30692	1	0	56672
CP043012.1	GCA_003887395	99,997	31282	103252	31282	100	0	1	6909	38189	31282	1	0	57760
CP027674.1	GCA_003887435	99,92	16317	133420	16304	100	0	3	69336	85652	1	16304	0	30047
NZ_CP006263.1	GCA_003887495	99,906	11721	98066	11721	100	6	1	35713	47428	11721	1	0	21579
CP027674.1	GCA_003887535	99,919	25934	133420	25930	100	11	5	104077	130004	1	25930	0	47766
NZ_CP018244.1	GCA_003887615	100	30691	92522	31219	98	0	0	1	30691	529	31219	0	56676
CP043012.1	GCA_003889795	100	31281	103252	31282	100	0	0	6909	38189	31282	2	0	57766
NZ_CP017437.1	GCA_003891235	100	38257	92565	38257	100	0	0	15352	53608	1	38257	0	70648
NZ_CP022408.1	GCA_003891495	100	15548	81950	15548	100	0	0	51345	66892	15548	1	0	28712
NZ_CP032809.1	GCA_003891915	99,99	41480	92771	42561	97	2	2	11357	52835	41479	1	0	76576
CP043012.1	GCA_003892255	100	31281	103252	31283	100	0	0	6909	38189	31283	3	0	57766
NZ_CP040317.1	GCA_003892655	99,991	42947	93331	42947	100	3	1	7906	50851	1	42947	0	79285
CP043012.1	GCA_003893555	99,994	31279	103252	31279	100	1	1	6911	38189	31279	2	0	57749
CP043012.1	GCA_003893615	99,997	31281	103252	31281	100	0	1	6909	38189	1	31280	0	57758
NZ_CP031923.1	GCA_003893935	99,995	19696	95298	19696	100	1	0	64056	83751	1	19696	0	36367
NZ_CP031923.1	GCA_003893975	99,995	20364	95298	20853	98	1	0	64144	84507	1	20364	0	37600
AP019707.1	GCA_003894455	99,949	19450	91036	19450	100	10	0	48812	68261	1	19450	0	35863
NZ_CP027389.1	GCA_003895475	100	20280	68062	20280	100	0	0	26698	46977	20280	1	0	37451
NZ_CP015816.1	GCA_003895485	99,997	29234	95910	29234	100	0	1	31239	60471	1	29234	0	53978
NZ_CP024480.1	GCA_003895785	100	18531	77062	18531	100	0	0	38906	57436	18531	1	0	34221
NC_013728.1	GCA_003896215	99,995	21613	111481	21613	100	1	0	86665	108277	1	21613	0	39907
NZ_CP006263.1	GCA_003896335	99,967	9046	98066	9046	100	3	0	83809	92854	1	9046	0	16689
NZ_CP041624.1	GCA_003896675	99,98	19554	95081	19554	100	4	0	46781	66334	19554	1	0	36088
NC_013369.1	GCA_003896915	100	22007	85167	22007	100	0	0	17256	39262	1	22007	0	40640
NZ_CP040306.1	GCA_003897355	99,987	38503	95621	39186	98	4	1	14810	53311	684	39186	0	71073
AP019707.1	GCA_003898255	99,975	20252	91036	20251	100	4	1	48012	68263	1	20251	0	37369
NZ_CP024480.1	GCA_003898495	100	36726	77062	36726	100	0	0	20709	57434	1	36726	0	67821
NC_013369.1	GCA_003899475	99,99	19452	85167	19452	100	2	0	47906	67357	1	19452	0	35911
NZ_CP027389.1	GCA_003899675	100	20093	68062	20093	100	0	0	26698	46790	20093	1	0	37105
NZ_CP038318.1	GCA_003899855	99,954	34522	92578	35640	97	11	4	15348	49866	34627	108	0	63656
CP027321.1	GCA_003899885	99,984	18922	84276	18920	100	1	2	46706	65627	18920	1	0	34924
NZ_CP027385.1	GCA_003901125	100	8735	118259	8735	100	0	0	66810	75544	1	8735	0	16131
NZ_CP018242.1	GCA_003901155	99,997	30707	92495	31237	98	1	0	1	30707	531	31237	0	56700
NC_013366.1	GCA_003901175	100	12993	77690	12993	100	0	0	52699	65691	12993	1	0	23994
CP027674.1	GCA_003901435	99,811	25929	133420	25923	100	42	7	104077	130004	1	25923	0	47604
CP043012.1	GCA_003902165	99,997	31281	103252	31282	100	0	1	6909	38189	1	31280	0	57758
NZ_CP041624.1	GCA_003902425	99,895	19052	95081	19052	100	0	2	38053	57084	19052	1	0	35054
NZ_CP035546.1	GCA_003902695	100	28910	94581	29441	98	0	0	1	28910	532	29441	0	53387
NZ_CP018244.1	GCA_003903035	100	45176	92522	45176	100	0	0	31990	77165	45176	1	0	83425
NC_019041.1	GCA_003903535	99,99	41665	92077	42457	98	4	0	45748	87412	42300	636	0	76919
NZ_CP038422.1	GCA_003903655	100	30707	94959	31422	98	0	0	1	30707	30707	1	0	56706
NZ_CP038362.1	GCA_003905255	99,998	45845	95928	45845	100	1	0	34642	80486	45845	1	0	84655
CP027641.1	GCA_003905275	99,81	7903	126957	7903	100	2	1	21813	29702	1	7903	0	14499
NC_013369.1	GCA_003905575	100	19452	85167	19452	100	0	0	47906	67357	1	19452	0	35922
CP027641.1	GCA_003906255	99,814	12388	126957	12388	100	22	1	33212	45598	12388	1	0	22748
CP027641.1	GCA_003906355	99,311	12485	126957	12484	100	77	5	55509	67986	1	12483	0	22570
NC_017907.1	GCA_003906675	99,993	30692	92728	31283	98	2	0	1	30692	30692	1	0	56667
CP027641.1	GCA_003906795	98,859	13142	126957	13137	100	136	11	55705	68837	1	13137	0	23425
CP043012.1	GCA_003907275	99,997	31281	103252	31281	100	0	1	6909	38189	1	31280	0	57758
CP043016.1	GCA_003907345	100	31817	91446	31834	100	0	0	8909	40725	31817	1	0	58755
NZ_CP006263.1	GCA_003907375	99,906	11721	98066	11721	100	6	1	35713	47428	11721	1	0	21579
NZ_CP027549.1	GCA_003907435	99,975	20032	94116	20031	100	2	3	15459	35488	1	20031	0	36961
CP027674.1	GCA_003907455	99,931	10208	133420	10207	100	6	1	6845	17052	10207	1	0	18811
CP027674.1	GCA_003907675	99,16	18210	133420	18191	100	117	25	10442	28634	1	18191	0	32747
NZ_CP038318.1	GCA_003907775	99,957	34523	92578	35636	97	9	4	15348	49866	34626	106	0	63664
CP043012.1	GCA_003907855	99,997	31281	103252	31281	100	0	1	6909	38189	1	31280	0	57758
CP027641.1	GCA_003908195	99,874	11090	126957	11096	100	14	0	32480	43569	11090	1	0	20402
NZ_CP038289.1	GCA_003908275	99,993	30693	96920	31499	97	2	0	1	30693	30693	1	0	56669
CP027674.1	GCA_003908475	99,919	25934	133420	25930	100	11	5	104077	130004	1	25930	0	47766
NZ_CP045828.1	GCA_003908535	99,953	8561	100778	8561	100	4	0	57373	65933	8561	1	0	15788
NC_013366.1	GCA_003908595	100	14830	77690	14830	100	0	0	52699	67528	14830	1	0	27386
NZ_CP027547.1	GCA_003910915	99,976	16574	95367	16572	100	0	4	76135	92706	16572	1	0	30581
NZ_CP027339.1	GCA_003912155	99,951	16379	92644	16379	100	0	8	28573	44943	16379	1	0	30195
NZ_CP027339.1	GCA_003912995	99,938	16145	92644	16145	100	2	8	28806	44942	16145	1	0	29752
NZ_CP035546.1	GCA_003913255	100	28910	94581	29435	98	0	0	1	28910	526	29435	0	53387
NZ_CP018244.1	GCA_003914035	100	44923	92522	44923	100	0	0	32009	76931	44923	1	0	82958
NZ_CP027339.1	GCA_003914115	99,937	15934	92644	15934	100	2	8	29018	44943	15934	1	0	29362
AP019709.1	GCA_003914195	99,994	16972	86874	16972	100	1	0	16834	33805	1	16972	0	31336
AP019709.1	GCA_003914415	99,995	22212	86874	22212	100	1	0	16834	39045	1	22212	0	41013
CP043012.1	GCA_003914855	99,997	31281	103252	31281	100	0	1	6909	38189	1	31280	0	57758
NC_013010.1	GCA_003915275	99,997	29093	94601	29441	99	1	0	1	29093	349	29441	0	53720
NZ_CP017445.1	GCA_003915475	99,986	42290	92726	42441	100	5	1	8576	50865	42289	1	0	78060
NZ_CP038308.1	GCA_003915515	99,997	37219	93167	37219	100	1	0	1193	38411	1	37219	0	68726
NZ_CP038354.1	GCA_003915795	99,997	32943	91510	32943	100	1	0	43138	76080	32943	1	0	60829
CP027581.1	GCA_003916435	98,737	8707	118822	8738	100	107	3	80543	89247	10	8715	0	15466
NC_013366.1	GCA_003916535	100	12994	77690	12994	100	0	0	52698	65691	12994	1	0	23996
NC_013369.1	GCA_003916615	100	19452	85167	19452	100	0	0	47906	67357	1	19452	0	35922
NC_013010.1	GCA_003916635	100	29093	94601	29705	98	0	0	1	29093	29093	1	0	53725
NZ_CP034795.1	GCA_003916975	100	31488	94987	31488	100	0	0	55713	87200	31488	1	0	58148

NZ_CP032804.1	GCA_003916985	99,998	42296	91253	42296	100	1	0	9015	51310	42296	1	0	78101
NZ_CP031923.1	GCA_003917035	99,985	20186	95298	20186	100	2	1	4677	24861	1	20186	0	37259
NC_013366.1	GCA_003917045	100	12994	77690	12994	100	0	0	52698	65691	12994	1	0	23996
AP019704.1	GCA_003917095	99,982	22204	92337	22204	100	4	0	16834	39037	1	22204	0	40981
NZ_CP027385.1	GCA_003917295	100	8624	118259	8624	100	0	0	66921	75544	1	8624	0	15926
NZ_CP038401.1	GCA_003917495	99,978	40760	92513	40754	100	3	1	32244	73003	40754	1	0	75215
NZ_CP028595.1	GCA_003918195	99,99	30861	95624	30861	100	3	0	22101	52961	1	30861	0	56973
NZ_CP038343.1	GCA_003918765	99,988	43068	92743	43066	100	3	1	7823	50890	43066	1	0	79503
NZ_CP034795.1	GCA_003918825	100	31488	94987	31488	100	0	0	55713	87200	31488	1	0	58148
NC_013369.1	GCA_003918885	99,995	19452	85167	19452	100	1	0	47906	67357	1	19452	0	35916
NZ_CP031340.1	GCA_003919135	99,984	19256	88744	20245	95	3	0	22868	42123	20245	990	0	35543
NZ_CP038320.1	GCA_003919415	99,996	23579	95338	24202	97	1	0	1	23579	518	24096	0	43537
NZ_CP032804.1	GCA_003919575	99,995	42297	91253	42297	100	1	1	9015	51310	42297	1	0	78095
AP019709.1	GCA_003919895	99,995	22212	86874	22212	100	1	0	16834	39045	1	22212	0	41013
NC_013369.1	GCA_003919995	99,961	20761	85167	20761	100	8	0	46595	67355	1	20761	0	38295
AP019707.1	GCA_003920015	99,949	19450	91036	19450	100	10	0	48812	68261	1	19450	0	35863
NZ_CP024480.1	GCA_003920215	100	36727	77062	36727	100	0	0	20709	57435	1	36727	0	67823
NZ_CP024480.1	GCA_003920235	99,996	26734	77062	26734	100	1	0	24189	50922	1	26734	0	49363
NZ_CP027339.1	GCA_003920375	99,931	15962	92644	15961	100	2	9	28990	44943	15961	1	0	29407
CP027674.1	GCA_003920995	99,96	32664	133420	32660	100	3	5	30547	63204	32660	1	0	60238
NZ_CP032792.1	GCA_003921295	99,995	42290	90310	42289	100	1	1	8090	50379	42289	1	0	78083
NC_013010.1	GCA_003921555	100	29093	94601	29441	99	0	0	1	29093	349	29441	0	53725
NZ_CP027339.1	GCA_003921585	99,937	15934	92644	15934	100	2	8	29018	44943	15934	1	0	29362
NZ_CP038314.1	GCA_003921655	100	15687	97630	16155	97	0	0	1	15687	275	15961	0	28969
AP019707.1	GCA_003922035	99,949	19450	91036	19450	100	10	0	48812	68261	1	19450	0	35863
NZ_CP032794.1	GCA_003922435	100	31786	92653	31786	100	0	0	24219	56004	1	31786	0	58698
NZ_CP027220.1	GCA_003922595	99,995	20187	94104	20186	100	0	1	38622	58808	1	20186	0	37272
NC_013010.1	GCA_003922775	100	29093	94601	29436	99	0	0	1	29093	344	29436	0	53725
NC_013354.1	GCA_003922815	100	20094	75546	20094	100	0	0	53563	73656	20094	1	0	37107
AP019707.1	GCA_003923135	99,949	19451	91036	19451	100	10	0	48813	68263	1	19451	0	35864
NZ_CP032790.1	GCA_003923235	99,986	27877	93284	27876	100	1	3	8079	35953	1	27876	0	51454
NZ_CP028701.1	GCA_003923535	100	42801	92738	42801	100	0	0	8078	50878	42801	1	0	79039
NZ_CP028111.1	GCA_003923635	99,967	12047	83211	12287	98	4	0	17447	29493	1	12047	0	22225
NC_013010.1	GCA_003924115	100	29093	94601	29709	98	0	0	1	29093	29093	1	0	53725
NC_013366.1	GCA_003924275	100	11613	77690	11613	100	0	0	54786	66398	11613	1	0	21446
NZ_CP018244.1	GCA_003924775	99,99	30707	92522	31420	98	3	0	1	30707	30707	1	0	56689
NZ_CP038348.1	GCA_003924875	100	31159	89216	31159	100	0	0	22110	53268	1	31159	0	57540
NC_013354.1	GCA_004156655	100	15235	75546	15811	96	0	0	1	15235	15235	1	0	28134
AP019707.1	GCA_004156855	99,974	19444	91036	19443	100	4	1	48820	68263	1	19443	0	35877
CP043012.1	GCA_004157135	99,997	31281	103252	31281	100	0	1	6909	38189	31281	2	0	57758
NZ_CP018238.1	GCA_004157195	99,929	62317	91420	62899	99	2	5	1	62283	62309	1	0	114800
NZ_CP018244.1	GCA_004157305	100	30691	92522	31405	98	0	0	1	30691	30691	1	0	56676
CP027641.1	GCA_004157635	99,798	12391	126957	12390	100	23	2	33212	45601	12390	1	0	22742
CP027674.1	GCA_004157695	99,914	11668	133420	11664	100	5	5	116298	127964	1	11664	0	21486
CP043012.1	GCA_004158115	100	31281	103252	31282	100	0	0	6909	38189	31282	2	0	57766
NZ_CP031923.1	GCA_004158215	100	19733	95298	19733	100	0	0	64019	83751	1	19733	0	36441
NZ_CP018244.1	GCA_004158235	100	30691	92522	31414	98	0	0	1	30691	30691	1	0	56676
AP019707.1	GCA_004158395	99,995	19453	91036	19453	100	1	0	48812	68264	1	19453	0	35918
NZ_CP038422.1	GCA_004158615	100	30707	94959	31420	98	0	0	1	30707	30707	1	0	56706
NZ_CP040306.1	GCA_004160135	99,992	38488	95621	38515	100	2	1	14810	53296	28	38515	0	71056
NZ_CP032794.1	GCA_004160215	99,987	31787	92653	31787	100	4	0	24219	56005	1	31787	0	58678
CP027641.1	GCA_004160775	99,308	12289	126957	12286	100	75	6	55705	67986	1	12286	0	22214
NC_017907.1	GCA_004160955	99,993	30692	92728	31283	98	2	0	1	30692	30692	1	0	56667
NZ_CP018238.1	GCA_004161095	99,921	61864	91420	62446	99	1	5	1	61824	61856	1	0	113900
CP027674.1	GCA_004161155	99,927	27438	133420	27434	100	10	5	104077	131508	1	27434	0	50549
NZ_CP040317.1	GCA_004161315	99,988	42784	93331	42784	100	5	0	8066	50849	1	42784	0	78980
NZ_CP032790.1	GCA_004161475	99,988	32524	93284	34120	95	2	2	12253	44775	1	32523	0	60037
NZ_CP040306.1	GCA_004161655	99,99	38487	95621	38514	100	3	1	14810	53295	28	38514	0	71049
CP043012.1	GCA_004161835	99,994	31279	103252	31279	100	1	1	6911	38189	31279	2	0	57749
NZ_CP038289.1	GCA_004161955	99,99	30691	96920	31238	98	3	0	1	30691	548	31238	0	56660
CP027378.1	GCA_004162095	99,241	7511	113102	7602	99	43	2	11439	18935	7526	16	0	13542
CP027641.1	GCA_004162125	98,961	9821	126957	9818	100	89	8	55632	65442	1	9818	0	17559
NZ_CP038422.1	GCA_004162315	100	31362	94959	31362	100	0	0	32009	63370	31362	1	0	57915
NZ_CP018244.1	GCA_004163475	99,997	30697	92522	31409	98	1	0	1	30697	30697	1	0	56682
NC_013369.1	GCA_004163655	100	19452	85167	19452	100	0	0	47906	67357	1	19452	0	35922
NZ_CP032796.1	GCA_004163815	99,997	30662	90978	31224	98	1	0	22096	52757	1	30662	0	56617
NZ_CP018244.1	GCA_004163835	100	30853	92522	31563	98	0	0	1	30853	30853	1	0	56975
CP027674.1	GCA_004163855	99,7	20983	133420	20979	100	59	4	71825	92807	20979	1	0	38396
NZ_CP006263.1	GCA_004163875	99,898	11721	98066	11721	100	7	1	35713	47428	11721	1	0	21573
NZ_CP032792.1	GCA_004163955	99,997	31220	90310	31220	100	1	0	19158	50377	1	31220	0	57647
AP019707.1	GCA_004164095	99,954	19473	91036	19473	100	9	0	48812	68284	1	19473	0	35911
NZ_CP027339.1	GCA_004164235	99,937	15978	92644	15978	100	2	8	28975	44944	15978	1	0	29444
NZ_CP031923.1	GCA_004164245	99,995	19573	95298	19573	100	1	0	64179	83751	1	19573	0	36140
CP027674.1	GCA_004164475	99,531	15337	133420	15332	100	67	5	115913	131249	1	15332	0	27918
CP027674.1	GCA_004164535	99,966	32406	133420	32395	100	0	6	31014	63419	32395	1	0	59771
NZ_CP040317.1	GCA_004164575	99,991	42973	93331	42973	100	3	1	7878	50849	1	42973	0	79333
NZ_CP027386.1	GCA_004164935	100	17677	54452	17677	100	0	0	25939	43615	1	17677	0	32644
NZ_CP027545.1	GCA_004164995	99,886	21143	101089	21142	100	20	4	17711	38850	21142	1	0	38908
CP058232.1	GCA_004165155	100	31280	94605	31280	100	0	0	13819	45098	1	31280	0	57764
CP027674.1	GCA_004165255	99,939	37850	133420	37829	100	2	4	31213	69062	37829	1	0	69749
NZ_CP038352.1	GCA_004165375	99,987	30357	92209	31675	96	4	0	1	30357	987	31343	0	56037
NZ_CP018242.1	GCA_004165515	99,997	30693	92495	31225	98	1	0	1	30693	533	31225	0	56674
CP043012.1	GCA_004165535	99,997	31281	103252	31281	100	0	1	6909	38189	1	31280	0	57758
NZ_CP027356.1	GCA_004165655	99,991	21782	71714	21782	100	1	1	44558	66338	1	21782	0	40211
CP027641.1	GCA_004165695	99,855	10359	126957	10359	100	15	0	33212	43570	10359	1	0	19047
NZ_CP018244.1	GCA_004165795	100	30693	92522	31401	98	0	0	1	30693	30693	1	0	56680
CP043012.1	GCA_004165935	99,997	31281	103252	31281	100	0	1	6909	38189	1	31280	0	57758
CP027641.1	GCA_004166095	99,817	10358	126957	10358	100	19	0	33212	43569	10358	1	0	19023
NZ_CP040317.1	GCA_004173615	99,993	42833	93331	42833	100	2	1	8020	50851	1	42833	0	79080
CP027674.1	GCA_004173635	99,822	14064	133420	14064	100	7	1	104077	118122	1	14064	0	25817
CP027674.1	GCA_004173655	98,968	14433	133420	14415	100	114	24	14219	28634	1	14415	0	25795
NZ_CP018244.1	GCA_004173675	100	30707	92522	31409	98	0	0	1	30707	30707	1	0	56706
NZ_CP064169.1	GCA_004174175	99,995	20956	95164	20956	100	1	0	40330	61285	1	20956	0	38693

CP027674.1	GCA_004174735	99,974	38049	133420	38039	100	0	5	31014	69062	38039	1	0	70199
NZ_CP018244.1	GCA_004174755	99,997	31338	92522	31338	100	1	0	31990	63327	31338	1	0	57865
NZ_CP027385.1	GCA_004174775	100	8573	118259	8573	100	0	0	66971	75543	1	8573	0	15832
NZ_CP028651.1	GCA_004174815	99,99	30668	94057	30668	100	2	1	19470	50136	30668	1	0	56615
NZ_CP028612.1	GCA_004175385	99,991	42300	92725	42592	99	4	0	8576	50875	42300	1	0	78092
CP043012.1	GCA_004175415	99,997	31281	103252	31282	100	1	0	6909	38189	31282	2	0	57760
NZ_CP040310.1	GCA_004175435	99,981	42785	93175	42782	100	4	2	8065	50848	1	42782	0	78962
NZ_CP031923.1	GCA_004175535	99,995	19739	95298	19739	100	1	0	64013	83751	1	19739	0	36446
CP027674.1	GCA_004176215	99,927	26148	133420	26144	100	9	5	104077	130218	1	26144	0	48172
NZ_CP018253.1	GCA_004176455	100	30999	95229	30999	100	0	0	24764	55762	1	30999	0	57245
NZ_CP018238.1	GCA_004176675	99,997	30691	91420	31277	98	0	1	1	30691	30690	1	0	56669
NZ_CP018242.1	GCA_004176985	99,993	30693	92495	31396	98	2	0	1	30693	30693	1	0	56669
CP027641.1	GCA_004177165	99,295	12485	126957	12483	100	79	5	55509	67986	1	12483	0	22559
NZ_CP024480.1	GCA_004180795	99,965	36721	77062	36719	100	10	2	20718	57437	1	36719	0	67736
CP027641.1	GCA_004181095	99,832	10106	126957	10105	100	3	2	21813	31905	10105	1	0	18556
CP027674.1	GCA_004181115	99,89	13662	133420	13658	100	5	5	116349	130004	1	13658	0	25137
NZ_CP018244.1	GCA_004181485	99,997	30707	92522	31424	98	1	0	1	30707	30707	1	0	56700
CP043012.1	GCA_004181505	99,997	31281	103252	31281	100	0	1	6909	38189	1	31280	0	57758
CP043012.1	GCA_004181705	99,997	31282	103252	31282	100	0	1	6909	38189	31282	1	0	57760
CP043012.1	GCA_004181785	99,994	31280	103252	31280	100	0	2	6911	38189	31280	2	0	57751
NZ_CP018244.1	GCA_004181805	100	30707	92522	31414	98	0	0	1	30707	30707	1	0	56706
CP043012.1	GCA_004182055	100	31281	103252	31282	100	0	0	6909	38189	31282	2	0	57766
NZ_CP018244.1	GCA_004182095	99,997	30692	92522	31407	98	1	0	1	30692	30692	1	0	56672
NZ_CP018244.1	GCA_004182795	100	30691	92522	31397	98	0	0	1	30691	30691	1	0	56676
CP027674.1	GCA_004183055	99,165	18210	133420	18191	100	116	25	10442	28634	1	18191	0	32753
NZ_CP018244.1	GCA_004183075	100	30707	92522	31416	98	0	0	1	30707	30707	1	0	56706
NZ_CP018242.1	GCA_004183115	99,997	30707	92495	31420	98	1	0	1	30707	30707	1	0	56700
NZ_CP040315.1	GCA_004183215	99,927	31305	95631	31287	100	4	2	22019	53322	1	31287	0	57664
CP027674.1	GCA_004183355	99,165	18210	133420	18191	100	116	25	10442	28634	1	18191	0	32753
NZ_CP032804.1	GCA_004183375	99,998	42294	91253	42294	100	1	0	9015	51308	42294	1	0	78097
NZ_CP027339.1	GCA_004183705	99,887	15948	92644	15948	100	10	8	29002	44941	15948	1	0	29344
NZ_CP018238.1	GCA_004184375	99,978	40502	91420	41084	99	0	4	1	40501	40494	1	0	74735
NZ_CP032792.1	GCA_004188555	99,997	31221	90310	31221	100	1	0	19157	50377	1	31221	0	57649
CP027674.1	GCA_004190035	98,92	23607	133420	23596	100	218	22	66151	89731	1	23596	0	42147
NZ_AP018800.1	GCA_004215555	99,939	13058	81004	13058	100	2	1	32518	45569	13058	1	0	24064
NZ_CP018244.1	GCA_004215755	99,997	31393	92522	31393	100	1	0	31985	63377	31393	1	0	57967
CP027674.1	GCA_004215775	99,925	25269	133420	25265	100	9	5	104077	129339	1	25265	0	46549
CP043016.1	GCA_004228425	99,96	22511	91446	22504	100	2	1	9176	31686	22504	1	0	41513
NZ_CP038314.1	GCA_004228795	100	15687	97630	16088	98	0	0	1	15687	190	15876	0	28969
NC_013728.1	GCA_004228865	99,991	23124	111481	23123	100	0	2	85155	108277	1	23123	0	42690
NC_017907.1	GCA_004228915	100	30692	92728	31223	98	0	0	1	30692	532	31223	0	56678
NZ_CP028124.1	GCA_004228985	99,957	11609	83012	11609	100	5	0	49504	61112	11609	1	0	21411
AP019704.1	GCA_004230385	99,959	22006	92337	21999	100	2	1	16834	38839	1	21999	0	40581
NZ_CP024480.1	GCA_004230445	99,994	34081	77062	34080	100	1	1	20709	54789	1	34080	0	62923
NC_013369.1	GCA_004230485	99,995	19452	85167	19452	100	1	0	47906	67357	1	19452	0	35916
NZ_CP064169.1	GCA_004230505	100	22381	95164	22879	98	0	0	52060	74440	499	22879	0	41330
NZ_CP040315.1	GCA_004230525	99,993	29959	95631	29959	100	2	0	15875	45833	1	29959	0	55313
NZ_CP031340.1	GCA_004230545	99,995	19246	88744	20235	95	1	0	22878	42123	20235	990	0	35536
NC_013369.1	GCA_004230585	99,995	20006	85167	20006	100	1	0	47352	67357	1	20006	0	36939
NZ_CP044146.1	GCA_004230605	99,996	25970	92722	25970	100	1	0	55737	81706	1	25970	0	47953
NZ_CP027318.1	GCA_004230665	100	18647	81954	18647	100	0	0	52148	70794	18647	1	0	34435
AP019709.1	GCA_004230685	99,995	22212	86874	22212	100	1	0	16834	39045	1	22212	0	41013
NC_013369.1	GCA_004230705	99,995	22205	85167	22205	100	1	0	17256	39460	1	22205	0	41000
NZ_CP028113.1	GCA_004230765	99,979	19451	94220	19451	100	4	0	13527	32977	19451	1	0	35898
NZ_CP037944.1	GCA_004230825	99,99	19451	88848	19451	100	2	0	50202	69652	19451	1	0	35909
NZ_CP038308.1	GCA_004230845	99,985	27110	93167	28420	95	2	1	1	27108	1311	28420	0	50039
CP041748.1	GCA_004230925	99,898	61808	91398	64797	95	30	21	29617	91398	1	61801	0	113800
NZ_CP044144.1	GCA_004230985	99,964	24826	92690	24826	100	0	3	10609	35425	1	24826	0	45786
NZ_CP024480.1	GCA_004231025	100	17034	77062	17034	100	0	0	21642	38675	1	17034	0	31456
NC_017907.1	GCA_004231045	100	30691	92728	31208	98	0	0	1	30691	518	31208	0	56676
NC_013369.1	GCA_004231085	100	19452	85167	19452	100	0	0	47906	67357	1	19452	0	35922
NC_013010.1	GCA_004231125	100	29239	94601	29521	99	0	0	1	29239	283	29521	0	53995
NZ_CP030765.1	GCA_004231145	99,985	27178	93169	28471	95	2	2	1202	28378	1295	28471	0	50165
NZ_CP027339.1	GCA_004231245	99,951	16293	92644	16903	96	0	8	28934	45218	16903	611	0	30036
NZ_CP024480.1	GCA_004231255	99,997	36851	77062	36851	100	1	0	20709	57559	1	36851	0	68046
NZ_CP027354.1	GCA_004231305	99,931	13100	57720	13100	100	2	7	15859	28951	13100	1	0	24134
NZ_CP032804.1	GCA_004231395	99,994	31236	91253	31260	100	2	0	20073	51308	31236	1	0	57671
NZ_CP038375.1	GCA_004231425	99,966	26776	91076	27361	98	7	2	64302	91076	27361	587	0	49395
NZ_CP013028.1	GCA_004231465	100	16186	74656	16186	100	0	0	34210	50395	16186	1	0	29890
NZ_CP024480.1	GCA_004231505	99,997	34800	77062	34800	100	1	0	20709	55508	1	34800	0	64258
NC_013366.1	GCA_004231535	100	12994	77690	12994	100	0	0	52698	65691	12994	1	0	23996
NZ_CP024480.1	GCA_004231665	100	36729	77062	36729	100	0	0	20709	57437	1	36729	0	67826
NC_013728.1	GCA_004231685	99,996	22287	111481	22287	100	1	0	85991	108277	1	22287	0	41151
NZ_CP015816.1	GCA_004231745	99,991	21681	95910	21681	100	2	0	31239	52919	21681	1	0	40027
NC_017907.1	GCA_004231785	100	30691	92728	31215	98	0	0	1	30691	525	31215	0	56676
NC_013010.1	GCA_004231825	100	29093	94601	29717	98	0	0	1	29093	29093	1	0	53725
NZ_CP027389.1	GCA_004231845	100	19191	68062	19191	100	0	0	26698	45888	19191	1	0	35440
NZ_CP027391.1	GCA_004231865	99,99	19605	98724	19604	100	1	1	57253	76857	1	19604	0	36191
NZ_CP024480.1	GCA_004231885	99,997	36851	77062	36851	100	1	0	20709	57559	1	36851	0	68046
NC_013010.1	GCA_004231905	100	29093	94601	29715	98	0	0	1	29093	29093	1	0	53725
AP019709.1	GCA_004231925	100	22212	86874	22212	100	0	0	16834	39045	1	22212	0	41018
NC_013010.1	GCA_004232005	100	29093	94601	29435	99	0	0	1	29093	343	29435	0	53725
NZ_CP027339.1	GCA_004232065	99,945	16379	92644	16379	100	1	8	28573	44943	16379	1	0	30190
NC_013369.1	GCA_004232125	100	19452	85167	19452	100	0	0	47906	67357	1	19452	0	35922
NZ_CP038320.1	GCA_004232145	99,988	25352	95338	25351	100	2	1	54563	79914	25351	1	0	46798
NC_013010.1	GCA_004232185	99,997	29093	94601	29716	98	1	0	1	29093	29093	1	0	53720
NC_013010.1	GCA_004232205	100	29953	94601	30581	98	0	0	1	29953	29953	1	0	55313
CP027641.1	GCA_004232225	99,261	21934	126957	21913	100	126	28	30189	52107	21913	1	0	39574
NZ_CP027220.1	GCA_004232365	99,986	22204	94104	22204	100	3	0	64890	87093	1	22204	0	40987
NC_019041.1	GCA_004232465	99,998	42164	92077	42798	99	1	0	45249	87412	42798	635	0	77857
CP027674.1	GCA_004232485	99,508	14430	133420	14425	100	66	5	116100	130529	1	14425	0	26249
NZ_CP027598.1	GCA_004232505	99,979	29167	74505	29167	100	4	2	4985	34149	1	29167	0	53827
NZ_CP027220.1	GCA_004232545	99,986	22204	94104	22204	100	3	0	64890	87093	1	22204	0	40987

AP019704.1	GCA_004232565	99,991	22006	92337	22006	100	2	0	16834	38839	1	22006	0	40627
NZ_CP023674.1	GCA_004232585	99,621	8187	87524	8400	97	21	5	47330	55506	8400	214	0	14938
NC_013010.1	GCA_004232625	100	29893	94601	30240	99	0	0	1	29893	348	30240	0	55203
NZ_CP034800.1	GCA_004232645	99,974	45848	94606	45836	100	0	1	3207	49054	45836	1	0	84588
NC_013366.1	GCA_004232685	100	13082	77690	13082	100	0	0	30451	43532	1	13082	0	24158
NC_013366.1	GCA_004232705	100	10881	77690	10881	100	0	0	54811	65691	10881	1	0	20094
NZ_CP027385.1	GCA_004232715	100	8756	118259	8756	100	0	0	66789	75544	1	8756	0	16170
AP019704.1	GCA_004232775	99,995	22204	92337	22204	100	1	0	16834	39037	1	22204	0	40998
NZ_CP027446.1	GCA_004232825	100	16029	74269	16029	100	0	0	6600	22628	1	16029	0	29601
NZ_CP024480.1	GCA_004232905	100	36729	77062	36729	100	0	0	20709	57437	1	36729	0	67826
AP019709.1	GCA_004232985	99,995	22014	86874	22014	100	1	0	16834	38847	1	22014	0	40647
NC_013366.1	GCA_004233365	100	12994	77690	12994	100	0	0	52698	65691	12994	1	0	23996
NZ_CP024480.1	GCA_004233765	100	36728	77062	36728	100	0	0	20709	57436	1	36728	0	67824
NZ_CP018626.1	GCA_004233825	99,954	32516	107692	32513	100	0	3	18517	51020	32513	1	0	59948
NZ_CP028606.1	GCA_004233955	100	32395	95648	32395	100	0	0	54650	87044	32395	1	0	59823
AP019704.1	GCA_004234025	100	22204	92337	22204	100	0	0	16834	39037	1	22204	0	41004
NZ_CP027386.1	GCA_004234045	100	17677	54452	17677	100	0	0	25939	43615	1	17677	0	32644
AP019707.1	GCA_004234065	99,99	19473	91036	19471	100	0	1	48790	68262	1	19471	0	35948
NZ_CP027436.1	GCA_004234085	100	18649	83611	18649	100	0	0	10744	29392	18649	1	0	34439
CP051632.1	GCA_004234225	100	15321	81965	15321	100	0	0	20940	36260	15321	1	0	28293
NZ_CP027385.1	GCA_004234265	100	8624	118259	8624	100	0	0	66921	75544	1	8624	0	15926
NC_013366.1	GCA_004234285	100	13852	77690	13852	100	0	0	52698	66549	13852	1	0	25580
NZ_CP038341.1	GCA_004234305	99,997	30693	92839	31498	97	1	0	1	30693	30693	1	0	56674
NC_013369.1	GCA_004234325	99,995	22205	85167	22205	100	1	0	17256	39460	1	22205	0	41000
NC_013010.1	GCA_004234385	99,997	29093	94601	29382	99	1	0	1	29093	290	29382	0	53720
NZ_CP038382.1	GCA_004234415	99,97	30336	91912	31353	97	9	0	1	30336	685	31020	0	55971
NC_013010.1	GCA_004234445	99,997	29091	94601	29433	99	1	0	1	29091	343	29433	0	53716
NZ_CP017437.1	GCA_004234535	100	38255	92565	38255	100	0	0	15352	53606	1	38255	0	70644
NZ_CP024480.1	GCA_004252705	99,997	36787	77062	36787	100	1	0	20679	57465	1	36787	0	67928
AP019707.1	GCA_004252885	99,995	19733	91036	19733	100	1	0	48531	68263	1	19733	0	36435
CP027674.1	GCA_004253505	99,906	26664	133420	26660	100	21	4	104077	130740	1	26660	0	49097
NZ_CP038422.1	GCA_004253805	99,975	31388	94959	31388	100	1	1	31990	63370	31388	1	0	57912
AP019707.1	GCA_004254515	99,954	19473	91036	19473	100	9	0	48812	68284	1	19473	0	35911
NZ_CP038308.1	GCA_004254845	99,986	35642	93167	35799	100	4	1	2272	37912	1	35642	0	65789
NZ_CP038422.1	GCA_004254885	99,975	31388	94959	31388	100	1	1	31990	63370	31388	1	0	57912
NZ_CP027354.1	GCA_004254895	99,948	15297	57720	15297	100	2	6	17893	33183	1	15297	0	28199
NZ_CP028619.1	GCA_004255145	99,994	36285	94487	36285	100	0	1	8076	44358	36285	1	0	66993
NZ_CP032792.1	GCA_004255305	99,997	31224	90310	31224	100	1	0	19156	50379	1	31224	0	57655
NZ_CP006263.1	GCA_004255345	99,906	11721	98066	11721	100	6	1	35713	47428	11721	1	0	21579
NZ_CP032792.1	GCA_004255505	99,997	31225	90310	31225	100	1	0	19155	50379	1	31225	0	57657
NZ_CP018242.1	GCA_004256205	99,997	30691	92495	31397	98	1	0	1	30691	30691	1	0	56671
NZ_CP017445.1	GCA_004256265	99,988	42294	92726	42446	100	5	0	8576	50869	42294	1	0	78075
NZ_CP040310.1	GCA_004256705	99,979	42948	93175	42945	100	5	2	7905	50851	1	42945	0	79257
CP027641.1	GCA_004256725	99,757	20555	126957	20554	100	35	3	23030	43570	20554	1	0	37667
NZ_CP018238.1	GCA_004256945	99,937	71923	91420	72504	99	2	6	1	71889	71914	1	0	132500
AP019707.1	GCA_004257445	99,953	19295	91036	19295	100	9	0	48991	68285	19295	1	0	35582
AP019709.1	GCA_004257585	99,994	17112	86874	17112	100	1	0	16834	33945	1	17112	0	31595
NZ_CP017445.1	GCA_004257845	99,986	42289	92726	42389	100	5	1	8576	50864	42288	1	0	78059
NZ_CP018244.1	GCA_004257945	100	30707	92522	31416	98	0	0	1	30707	30707	1	0	56706
NZ_CP018238.1	GCA_004257985	99,951	41081	91420	41083	100	3	5	30692	71765	41071	1	0	75735
NZ_CP018244.1	GCA_004258045	100	30691	92522	31406	98	0	0	1	30691	30691	1	0	56676
CP058232.1	GCA_004258065	99,994	31558	94605	31558	100	2	0	13543	45100	1	31558	0	58266
CP027641.1	GCA_004258745	98,712	11644	126957	11639	100	133	11	55632	67263	1	11639	0	20655
NZ_CP028653.1	GCA_004258765	99,986	42304	92739	42553	99	4	2	8576	50879	42302	1	0	78086
NZ_CP040317.1	GCA_004259825	99,993	42833	93331	42833	100	2	1	8020	50851	1	42833	0	79080
NZ_CP018242.1	GCA_004259865	99,997	31647	92495	32360	98	1	0	1	31647	31647	1	0	58436
NZ_CP018244.1	GCA_004259945	100	30707	92522	31235	98	0	0	1	30707	529	31235	0	56706
NZ_CP027339.1	GCA_004260635	99,881	16016	92644	16016	100	11	8	28934	44941	16016	1	0	29464
AP019707.1	GCA_004260985	99,954	19473	91036	19473	100	9	0	48812	68284	1	19473	0	35911
CP043012.1	GCA_004261185	100	31281	103252	31283	100	0	0	6909	38189	31283	3	0	57766
CP043012.1	GCA_004261245	99,99	31281	103252	31281	100	2	1	6909	38189	1	31280	0	57747
NZ_CP027354.1	GCA_004261385	99,948	15514	57720	15514	100	1	7	15857	31363	1	15514	0	28598
CP027674.1	GCA_004261405	99,928	26437	133420	26433	100	9	5	104077	130507	1	26433	0	48706
NZ_CP032792.1	GCA_004262065	99,997	31220	90310	31220	100	1	0	19158	50377	1	31220	0	57647
NZ_CP038422.1	GCA_004262605	99,979	37261	94959	37261	100	1	1	31990	69243	37261	1	0	68757
NZ_CP040317.1	GCA_004262885	99,991	42961	93331	42961	100	3	1	7906	50865	1	42961	0	79311
NZ_CP028653.1	GCA_004263105	99,986	42307	92739	42457	100	4	2	8576	50882	42305	1	0	78092
CP043012.1	GCA_004263345	99,997	31282	103252	31284	100	0	1	6909	38189	31284	3	0	57760
NZ_CP032809.1	GCA_004263405	99,995	41479	92771	42308	98	2	0	11357	52835	41479	1	0	76587
NZ_CP040317.1	GCA_004264305	99,989	35988	93331	35988	100	3	1	8068	44054	1	35988	0	66434
NC_013354.1	GCA_004264415	100	15236	75546	15812	96	0	0	1	15236	15236	1	0	28136
CP027674.1	GCA_004264465	99,895	23888	133420	23883	100	20	5	104077	127964	1	23883	0	43969
NZ_CP027589.1	GCA_004264505	99,992	11810	75065	11810	100	1	0	13738	25547	1	11810	0	21804
NZ_CP028124.1	GCA_004264605	99,985	13042	83012	13361	98	2	0	47207	60248	13042	1	0	24074
NZ_CP027339.1	GCA_004264665	99,887	15969	92644	15969	100	10	8	29004	44964	15969	1	0	29383
NZ_CP012498.1	GCA_004264685	99,831	56962	213847	56952	100	41	13	87996	144912	56952	1	0	104600
NZ_CP018244.1	GCA_004264705	100	30707	92522	31413	98	0	0	1	30707	30707	1	0	56706
CP043012.1	GCA_004265505	99,994	31281	103252	31281	100	1	1	6909	38189	1	31280	0	57753
NZ_CP015022.1	GCA_004265605	99,994	31239	95170	31239	100	2	0	55680	86918	1	31239	0	57677
NZ_CP018242.1	GCA_004265965	99,997	30691	92495	31223	98	1	0	1	30691	533	31223	0	56671
NZ_CP018244.1	GCA_004265985	100	30707	92522	31421	98	0	0	1	30707	30707	1	0	56706
NZ_CP018244.1	GCA_004266535	100	30738	92522	31620	97	0	0	1	30738	700	31437	0	56763
NZ_CP027356.1	GCA_004267805	99,994	17935	71714	17935	100	0	1	44558	62491	1	17935	0	33113
NZ_CP038368.1	GCA_004268265	99,997	30877	93665	31596	98	0	1	1	30876	720	31596	0	57012
NZ_CP032809.1	GCA_004268445	99,993	41466	92771	42325	98	2	1	11357	52821	41466	1	0	76555
CP043012.1	GCA_004269365	100	31281	103252	31282	100	0	0	6909	38189	31282	2	0	57766
NZ_CP018238.1	GCA_004269685	99,994	48676	91420	49261	99	1	2	1	48676	48674	1	0	89870
NZ_CP038382.1	GCA_004269705	99,98	30336	91912	31258	97	6	0	1	30336	30668	333	0	55987
NZ_CP018244.1	GCA_004269745	100	30691	92522	31404	98	0	0	1	30691	30691	1	0	56676
NZ_CP018244.1	GCA_004270045	99,997	30707	92522	31418	98	1	0	1	30707	30707	1	0	56700
NZ_CP018238.1	GCA_004270275	99,986	71772	91420	72360	99	0	4	1	71765	71769	1	0	132500
NZ_CP027339.1	GCA_004270345	99,887	16000	92644	16000	100	10	8	28974	44965	16000	1	0	29440
NZ_CP040317.1	GCA_004270545	99,991	42879	93331	42879	100	3	1	7974	50851	1	42879	0	79159

NZ_CP032804.1	GCA_004270715	99,995	41761	91253	41761	100	2	0	9548	51308	41761	1	0	77107
NZ_CP032792.1	GCA_004270725	100	31227	90310	31227	100	0	0	19153	50379	1	31227	0	57666
NZ_CP018244.1	GCA_004271775	100	30707	92522	31238	98	0	0	1	30707	532	31238	0	56706
CP027641.1	GCA_004272375	99,313	12370	126957	12368	100	76	5	55624	67986	1	12368	0	22364
NZ_CP038314.1	GCA_004272705	99,995	21256	97630	21256	100	1	0	18730	39985	1	21256	0	39247
NZ_CP038323.1	GCA_004273465	99,993	27033	92701	27116	100	2	0	1	27033	84	27116	0	49910
NZ_CP027339.1	GCA_004273505	99,939	16369	92644	16369	100	2	8	28958	45318	16369	1	0	30166
NZ_CP018238.1	GCA_004273525	99,986	71896	91420	72485	99	0	4	1	71889	71893	1	0	132700
AP019704.1	GCA_004273565	99,991	22010	92337	22009	100	1	1	16834	38843	1	22009	0	40632
CP043012.1	GCA_004273585	99,99	31281	103252	31282	100	2	1	6909	38189	1	31280	0	57747
NZ_CP040306.1	GCA_004273625	99,992	38503	95621	38530	100	2	1	14810	53311	28	38530	0	71084
NZ_CP018244.1	GCA_004273705	100	30707	92522	31239	98	0	0	1	30707	533	31239	0	56706
NZ_CP032794.1	GCA_004273745	100	19831	92653	19831	100	0	0	57319	77149	1	19831	0	36622
NZ_CP032809.1	GCA_004274045	99,993	41465	92771	42450	98	2	1	11357	52821	41464	1	0	76553
CP043012.1	GCA_004274765	99,997	31281	103252	31281	100	0	1	6909	38189	1	31280	0	57758
NZ_CP040317.1	GCA_004275585	99,988	42947	93331	42947	100	4	1	7906	50851	1	42947	0	79279
NZ_CP013028.1	GCA_004275675	99,771	8305	74656	8630	96	19	0	23334	31638	26	8330	0	15232
NZ_CP031923.1	GCA_004276095	99,995	19865	95298	19865	100	1	0	63887	83751	1	19865	0	36679
NZ_CP028653.1	GCA_004276275	99,986	42303	92739	42455	100	4	2	8576	50878	42301	1	0	78084
NZ_CP018238.1	GCA_004276295	99,986	71953	91420	72541	99	0	4	1	71946	71950	1	0	132800
NZ_CP031923.1	GCA_004276335	99,995	19775	95298	19775	100	1	0	63977	83751	1	19775	0	36513
NZ_CP012499.1	GCA_004278555	99,998	53104	223952	53104	100	1	0	18541	71644	1	53104	0	98060
NZ_CP018244.1	GCA_004280725	100	32743	92522	32743	100	0	0	44189	76931	32743	1	0	60465
NZ_CP040317.1	GCA_004280875	99,993	42879	93331	42879	100	2	1	8020	50897	1	42879	0	79165
NZ_CP038422.1	GCA_004281135	99,997	30691	94959	31223	98	1	0	1	30691	533	31223	0	56671
CP027581.1	GCA_004281195	99,875	40943	118822	40931	100	19	4	71313	112235	40931	1	0	75296
CP027674.1	GCA_004289035	99,81	11600	133420	11600	100	21	1	104077	115675	1	11600	0	21298
NC_013010.1	GCA_004766575	99,99	29094	94601	30434	96	3	0	1	29094	533	29626	0	53710
NZ_CP037944.1	GCA_004766705	99,995	20581	88848	20581	100	1	0	49199	69779	20581	1	0	38001
NZ_CP038382.1	GCA_004766745	99,974	30336	91912	31623	96	8	0	1	30336	30811	476	0	55976
NZ_CP027391.1	GCA_004766755	99,995	22042	98724	22042	100	1	0	17188	39229	22042	1	0	40699
NZ_CP038382.1	GCA_004766775	99,974	30336	91912	31623	96	8	0	1	30336	30811	476	0	55976
NC_013366.1	GCA_004766795	99,968	15747	77690	15747	100	5	0	35764	51510	15747	1	0	29052
NC_013010.1	GCA_004766845	99,99	29094	94601	30434	96	3	0	1	29094	533	29626	0	53710
NZ_CP024055.1	GCA_004766955	99,493	6507	88839	6752	96	21	4	16244	22739	6605	100	0	11823
NZ_CP023164.1	GCA_004767025	99,97	10004	122641	10004	100	2	1	10093	20095	1	10004	0	18456
NZ_CP041624.1	GCA_004767115	99,975	24363	95081	24363	100	6	0	47313	71675	1	24363	0	44957
CP027378.1	GCA_004767135	99,931	10103	113102	10103	100	2	5	96415	106512	10103	1	0	18613
NZ_CP038382.1	GCA_004769025	99,974	30336	91912	31623	96	8	0	1	30336	813	31148	0	55976
AP019704.1	GCA_004796595	99,929	19710	92337	19710	100	14	0	48667	68376	19710	1	0	36321
AP019707.1	GCA_004796605	99,926	20325	91036	21053	97	15	0	49420	69744	20325	1	0	37451
AP019707.1	GCA_004796635	99,981	20593	91036	20593	100	4	0	48617	69209	1	20593	0	38007
AP019704.1	GCA_004796905	99,99	19710	92337	19710	100	2	0	48667	68376	19710	1	0	36387
AP019704.1	GCA_004796915	100	19832	92337	19832	100	0	0	48545	68376	19832	1	0	36623
AP019704.1	GCA_004797065	99,966	20461	92337	20461	100	7	0	48667	69127	20461	1	0	37746
AP019704.1	GCA_004797265	99,971	20461	92337	20461	100	6	0	48667	69127	20461	1	0	37752
AP019704.1	GCA_004797745	99,99	19709	92337	19709	100	2	0	48668	68376	19709	1	0	36385
AP019704.1	GCA_004797875	99,985	20461	92337	20461	100	3	0	48667	69127	20461	1	0	37768
NZ_CP027370.1	GCA_005037875	99,967	23916	176149	23916	100	4	4	38843	62754	23916	1	0	44117
NZ_CP028382.1	GCA_005037905	99,934	36394	160675	36378	100	7	2	53688	90080	36378	1	0	67058
NZ_CP024055.1	GCA_005037915	99,508	6503	88839	6651	98	20	4	16244	22736	100	6600	0	11821
NZ_CP027339.1	GCA_005037985	99,907	16095	92644	16095	100	7	8	28937	45023	1	16095	0	29632
NZ_CP012501.1	GCA_005038015	100	28072	242187	28150	100	0	0	93029	121100	28072	1	0	51840
NZ_CP027441.1	GCA_005038135	99,981	77181	173714	77181	100	15	0	77031	154211	1	77181	0	142400
NZ_CP027441.1	GCA_005038265	99,975	77182	173714	77181	100	17	2	77031	154211	1	77181	0	142400
NZ_CP023164.1	GCA_005038345	99,682	28294	122641	28294	100	3	82	56881	85087	1	28294	0	51670
NZ_CP027588.1	GCA_005038405	99,878	11469	58109	11469	100	14	0	44274	55742	11469	1	0	21102
NZ_CP023674.1	GCA_005038425	99,604	8583	87524	8869	97	23	6	46935	55506	1	8583	0	15651
NZ_CP023674.1	GCA_005038445	99,606	8628	87524	8898	97	23	6	46890	55506	8898	271	0	15734
NZ_CP024055.1	GCA_005038465	99,493	6507	88839	6683	97	21	4	16244	22739	6584	79	0	11823
NZ_CP027588.1	GCA_005038475	99,532	11331	58109	11838	96	35	8	44351	55664	204	11533	0	20615
NZ_CP023674.1	GCA_005038525	99,61	8974	87524	9253	97	23	7	46544	55506	1	8973	0	16367
NZ_CP028121.1	GCA_005038535	99,637	9653	109466	10068	96	30	5	4430	14078	10068	417	0	17627
NZ_CP027343.1	GCA_005038595	99,987	37384	131410	37384	100	1	4	32727	70106	37384	1	0	69004
NZ_CP027441.1	GCA_005038625	99,977	77971	173714	81015	96	17	1	77031	155001	81015	3046	0	143900
NZ_CP031909.1	GCA_005038655	99,997	36917	73224	36917	100	1	0	15407	52323	1	36917	0	68168
NZ_CP031909.1	GCA_005038665	100	16655	73224	16655	100	0	0	15436	32090	16655	1	0	30757
NC_013354.1	GCA_005038675	100	10852	75546	10852	100	0	0	53482	64333	10852	1	0	20040
NZ_CP027441.1	GCA_005038955	99,981	77181	173714	77181	100	15	0	77031	154211	77181	1	0	142400
NZ_CP027370.1	GCA_005039015	99,333	48887	176149	51070	96	300	16	109612	158476	101	48983	0	88448
NZ_CP027446.1	GCA_005039135	99,975	16119	74269	16119	100	4	0	7143	23261	1	16119	0	29745
NZ_CP023674.1	GCA_005039175	99,606	8619	87524	8904	97	23	6	46899	55506	1	8619	0	15717
NZ_CP027588.1	GCA_005039195	99,513	10688	58109	10905	98	34	8	44351	55021	219	10905	0	19433
NZ_CP023674.1	GCA_005039205	99,62	8673	87524	8952	97	22	6	46845	55506	8952	280	0	15823
NZ_CP023674.1	GCA_005039215	99,558	8828	87524	9121	97	28	6	46690	55506	1	8828	0	16076
NZ_CP027391.1	GCA_005039305	99,976	20658	98724	20657	100	4	1	56281	76938	1	20657	0	38119
NZ_CP027370.1	GCA_005039315	99,945	32523	176149	32523	100	12	5	31058	63574	32523	1	0	59954
AP019709.1	GCA_005039325	99,938	17769	86874	17847	100	1	3	16754	34512	17847	79	0	32744
NZ_CP027386.1	GCA_005039985	99,978	17842	54452	17842	100	1	1	25857	43695	17842	1	0	32923
NZ_CP027598.1	GCA_005039995	99,984	18213	74505	18213	100	0	3	55697	73906	18213	1	0	33613
NZ_CP009107.1	GCA_005040005	99,983	29521	161447	29521	100	5	0	76450	105970	29521	1	0	54488
NZ_CP027598.1	GCA_005040045	99,978	18214	74505	18214	100	0	4	55697	73906	18214	1	0	33610
NZ_CP027578.1	GCA_005040145	99,932	22175	87714	22173	100	9	4	42330	64500	22173	1	0	40861
NZ_CP027386.1	GCA_005040155	99,972	17839	54452	17839	100	5	0	25857	43695	17839	1	0	32915
CP027378.1	GCA_005040215	100	11955	113102	11955	100	0	0	7588	19542	11955	1	0	22077
NC_017907.1	GCA_005040245	99,996	47758	92728	47758	100	2	0	31910	79667	1	47758	0	88182
NZ_CP027386.1	GCA_005040255	99,972	17842	54452	17842	100	2	1	25857	43695	1	17842	0	32917
NZ_CP027441.1	GCA_005040305	99,977	77061	173714	77061	100	17	1	77031	154090	1	77061	0	142200
NZ_CP038341.1	GCA_005040325	100	30773	92839	31666	97	0	0	1	30773	30773	1	0	56828
NZ_CP027585.1	GCA_005040335	99,993	29366	160576	29377	100	1	1	40424	69788	1	29366	0	54216
NZ_CP038359.1	GCA_005040345	99,985	47743	92704	47742	100	6	1	31910	79652	1	47742	0	88125
NZ_CP032806.1	GCA_005040355	99,993	42961	92785	42960	100	1	2	7998	50957	42960	1	0	79316
NZ_CP027545.1	GCA_005040415	99,982	27860	101089	27859	100	0	5	11075	38930	27859	1	0	51415

CP027378.1	GCA_005040425	100	11955	113102	11955	100	0	0	7588	19542	1	11955	0	22077
NC_013728.1	GCA_005040525	99,914	20900	111481	20980	100	11	3	86987	107882	1	20897	0	38488
NZ_CP041624.1	GCA_005040545	99,937	47723	95081	47696	100	3	2	31888	79610	47696	1	0	87936
NZ_CP027339.1	GCA_005040555	99,946	16541	92644	16541	100	1	8	28492	45024	1	16541	0	30489
NZ_CP027545.1	GCA_005040615	99,837	23857	101089	23829	100	1	11	62501	86347	1	23829	0	43805
NZ_CP028431.1	GCA_005040625	99,992	12358	76698	12436	99	1	0	24525	36882	79	12436	0	22816
NZ_CP061760.1	GCA_005040645	99,971	59595	172576	59594	100	15	2	65203	124796	1	59594	0	110000
NZ_CP013028.1	GCA_005040725	100	8233	74656	8233	100	0	0	6687	14919	1	8233	0	15204
NZ_CP027545.1	GCA_005040735	99,968	27860	101089	27859	100	4	5	11075	38930	27859	1	0	51393
NC_013354.1	GCA_005040755	99,987	14991	75546	15725	95	2	0	1	14991	15069	79	0	27673
NZ_CP061760.1	GCA_005040835	99,973	59595	172576	59595	100	15	1	65203	124796	1	59595	0	110000
NZ_CP061760.1	GCA_005041035	99,932	60385	172576	63429	95	39	2	64413	124796	3046	63429	0	111300
NZ_CP027343.1	GCA_005041095	99,383	36310	131410	36879	98	208	14	85471	121773	36879	579	0	65797
NC_013354.1	GCA_005041155	100	20254	75546	20254	100	0	0	53483	73736	1	20254	0	37403
NZ_CP028686.1	GCA_005041175	99,995	42956	92724	42955	100	1	1	7997	50952	1	42955	0	79312
NZ_CP028701.1	GCA_005041255	100	42963	92738	42963	100	0	0	7997	50959	1	42963	0	79338
NZ_CP027588.1	GCA_005041265	99,975	28230	58109	29337	96	6	1	1	28229	1108	29337	0	52091
NZ_CP031909.1	GCA_005041275	100	36917	73224	36917	100	0	0	15407	52323	1	36917	0	68173
NZ_CP027588.1	GCA_005041295	99,975	28230	58109	29337	96	6	1	1	28229	28230	1	0	52091
NZ_CP031909.1	GCA_005041345	100	36917	73224	36917	100	0	0	15407	52323	1	36917	0	68173
NZ_CP018251.1	GCA_005041375	100	30772	91789	31349	98	0	0	1	30772	578	31349	0	56826
NZ_CP038283.1	GCA_005041455	99,984	24291	93247	25098	97	4	0	1	24291	669	24959	0	44835
NZ_CP027385.1	GCA_005041565	99,965	8585	118259	8585	100	3	0	67039	75623	8585	1	0	15837
NZ_CP027453.1	GCA_005041585	99,492	13963	159611	14098	99	71	0	90251	104213	14098	136	0	25392
NZ_CP027545.1	GCA_005041595	99,982	27860	101089	27859	100	0	5	11075	38930	27859	1	0	51415
NZ_CP027386.1	GCA_005041665	99,972	17842	54452	17842	100	2	1	25857	43695	17842	1	0	32917
NZ_CP064169.1	GCA_005041675	99,985	19909	95164	19909	100	3	0	26771	46679	19909	1	0	36749
NZ_CP038406.1	GCA_005041695	99,998	42964	92740	42963	100	0	1	7998	50961	42963	1	0	79333
NZ_CP027343.1	GCA_005041705	99,974	31174	131410	31173	100	2	5	38938	70106	1	31173	0	57518
CP027641.1	GCA_005041735	99,301	12442	126957	12520	99	78	5	55552	67986	1	12440	0	22485
NZ_CP027343.1	GCA_005041765	99,99	31172	131410	31172	100	0	3	38938	70106	31172	1	0	57544
NZ_CP061760.1	GCA_005041775	99,95	60385	172576	63429	95	28	2	64413	124796	60384	1	0	111300
NZ_CP061760.1	GCA_005041805	99,95	60385	172576	63429	95	28	2	64413	124796	60384	1	0	111300
NZ_CP061760.1	GCA_005041835	99,932	60385	172576	63429	95	39	2	64413	124796	3046	63429	0	111300
NZ_CP061760.1	GCA_005041865	99,95	60385	172576	63429	95	28	2	64413	124796	3046	63429	0	111300
NZ_CP061760.1	GCA_005041905	99,95	60385	172576	63429	95	28	2	64413	124796	3046	63429	0	111300
NZ_CP061760.1	GCA_005041935	99,95	60385	172576	63429	95	28	2	64413	124796	60384	1	0	111300
NZ_CP061760.1	GCA_005042025	99,95	60385	172576	63429	95	28	2	64413	124796	3046	63429	0	111300
NZ_CP027343.1	GCA_005042035	100	9780	131410	9780	100	0	0	71318	81097	9780	1	0	18061
NZ_CP061760.1	GCA_005042055	99,95	60385	172576	63429	95	28	2	64413	124796	3046	63429	0	111300
NZ_CP027588.1	GCA_005042075	99,986	28230	58109	29341	96	3	1	1	28229	1112	29341	0	52108
NZ_CP027339.1	GCA_005042105	99,925	16076	92644	16076	100	4	8	28957	45024	16076	1	0	29613
NZ_CP027588.1	GCA_005042135	99,986	28230	58109	29341	96	3	1	1	28229	28230	1	0	52108
NZ_CP012499.1	GCA_005042175	99,994	52172	223952	52172	100	2	1	128161	180331	1	52172	0	96326
NZ_CP037944.1	GCA_005042205	99,958	18980	88848	19061	100	7	1	16255	35233	18980	1	0	35004
NZ_CP027343.1	GCA_005042225	99,981	31173	131410	31173	100	2	4	38938	70106	31173	1	0	57529
NZ_CP061760.1	GCA_005042255	99,963	59595	172576	59593	100	19	2	65203	124796	1	59593	0	109900
NZ_CP061760.1	GCA_005042275	99,96	59595	172576	59593	100	21	2	65203	124796	59593	1	0	109900
NZ_CP061760.1	GCA_005042305	99,963	59595	172576	59593	100	19	2	65203	124796	59593	1	0	109900
NZ_CP061760.1	GCA_005042365	99,96	59595	172576	59593	100	21	2	65203	124796	1	59593	0	109900
NZ_CP061760.1	GCA_005042375	99,973	59595	172576	59595	100	15	1	65203	124796	59595	1	0	110000
NZ_CP061760.1	GCA_005042385	99,976	21121	172576	21121	100	5	0	37417	58537	21121	1	0	38976
NZ_CP061760.1	GCA_005042395	99,963	59595	172576	59593	100	19	2	65203	124796	1	59593	0	109900
NZ_CP022408.1	GCA_005042475	100	20841	81950	20841	100	0	0	51717	72557	1	20841	0	38487
NZ_CP022408.1	GCA_005042485	100	20841	81950	20841	100	0	0	51717	72557	1	20841	0	38487
NZ_CP006028.1	GCA_005042505	99,958	16572	87120	16572	100	4	1	50897	67465	1	16572	0	30561
NZ_CP027385.1	GCA_005042555	100	8586	118259	8586	100	0	0	67039	75624	1	8586	0	15856
NZ_CP032792.1	GCA_005042575	99,994	31250	90310	31394	100	1	1	19147	50395	82	31331	0	57695
NZ_CP028382.1	GCA_005042585	99,981	36243	160675	36243	100	7	0	92620	128862	1	36243	0	66890
NZ_CP027545.1	GCA_005042625	99,968	27857	101089	27856	100	4	5	11075	38927	27856	1	0	51387
NZ_CP027343.1	GCA_005042655	100	9780	131410	9780	100	0	0	71318	81097	9780	1	0	18061
NZ_CP061760.1	GCA_005042705	99,958	59595	172576	59593	100	22	2	65203	124796	59593	1	0	109900
NZ_CP061760.1	GCA_005042715	99,963	59595	172576	59593	100	19	2	65203	124796	1	59593	0	109900
CP051632.1	GCA_005042755	100	13148	81965	13148	100	0	0	20860	34007	1	13148	0	24280
NZ_CP027385.1	GCA_005042785	100	8585	118259	8585	100	0	0	67039	75623	1	8585	0	15854
NC_013369.1	GCA_005042795	99,921	20139	85167	20139	100	13	1	19211	39346	20139	1	0	37098
NZ_CP027385.1	GCA_005042825	99,955	8916	118259	8916	100	4	0	66709	75624	8916	1	0	16443
NZ_CP027441.1	GCA_005042875	99,974	77182	173714	77180	100	17	3	77031	154211	77180	1	0	142400
NZ_CP024055.1	GCA_005042885	99,983	11652	88839	11652	100	2	0	55578	67229	11652	1	0	21507
NC_013369.1	GCA_005042895	99,968	22172	85167	22172	100	4	1	17174	39342	1	22172	0	40902
NZ_CP027545.1	GCA_005042935	99,968	27860	101089	27859	100	4	5	11075	38930	1	27859	0	51393
NZ_CP031899.1	GCA_005042955	99,983	59289	160712	59288	100	9	1	69691	128979	1	59288	0	109400
NC_013728.1	GCA_005042965	99,981	21161	111481	21160	100	3	1	87198	108358	21160	1	0	39054
NZ_CP031909.1	GCA_005043025	100	36917	73224	36917	100	0	0	15407	52323	1	36917	0	68173
NZ_CP027583.1	GCA_005043035	99,991	22153	88339	22153	100	2	0	42236	64388	22153	1	0	40898
NZ_CP009105.1	GCA_005043055	100	29833	168318	29911	100	0	0	76455	106287	1	29833	0	55092
NZ_CP027339.1	GCA_005043095	99,938	16076	92644	16076	100	2	8	28957	45024	1	16076	0	29625
NZ_CP027386.1	GCA_005043105	99,963	19061	54452	19059	100	4	3	878	19937	1	19059	0	35157
NZ_CP024480.1	GCA_005043145	99,994	31483	77062	31560	100	2	0	20629	52111	1	31483	0	58128
AP019704.1	GCA_005043175	99,959	19618	92337	19618	100	2	1	48716	68327	19618	1	0	36178
NZ_CP031909.1	GCA_005043235	99,996	23209	73224	23288	100	1	0	15435	38643	1	23209	0	42854
CP027321.1	GCA_005043245	99,974	19061	84276	19059	100	3	2	46626	65686	19059	1	0	35170
NZ_CP027576.1	GCA_005043265	99,993	13405	78427	13405	100	1	0	8068	21472	1	13405	0	24749
NZ_CP027385.1	GCA_005043335	100	8586	118259	8586	100	0	0	67039	75624	8586	1	0	15856
NZ_CP027588.1	GCA_005043565	99,523	11331	58109	11852	96	36	8	44351	55664	11634	305	0	20609
NC_013354.1	GCA_005043575	99,987	15314	75546	15969	96	2	0	1	15314	15314	1	0	28269
NZ_CP027386.1	GCA_005043615	99,978	17842	54452	17842	100	1	1	25857	43695	1	17842	0	32923
CP014753.1	GCA_005043655	100	15314	72996	15970	96	0	0	1	15314	15314	1	0	28280
NC_013366.1	GCA_005043695	100	13401	77690	13401	100	0	0	52621	66021	13401	1	0	24748
NZ_CP031909.1	GCA_005043705	99,997	29485	73224	29485	100	1	0	15438	44922	1	29485	0	54444
NZ_CP022408.1	GCA_005044015	100	15474	81950	15474	100	0	0	51717	67190	1	15474	0	28576
NZ_CP022408.1	GCA_005044025	100	15474	81950	15474	100	0	0	51717	67190	1	15474	0	28576
NC_013366.1	GCA_005044075	99,99	10468	77690	10468	100	1	0	52619	63086	10468	1	0	19326

NC_013366.1	GCA_005044095	99,98	15026	77690	15026	100	3	0	52618	67643	1	15026	0	27732
NZ_CP022408.1	GCA_005044105	100	15474	81950	15474	100	0	0	51717	67190	1	15474	0	28576
NZ_CP027318.1	GCA_005044145	99,91	18807	81954	18806	100	16	1	52068	70874	1	18806	0	34635
NC_013366.1	GCA_005044175	99,98	10010	77690	10010	100	2	0	52618	62627	1	10010	0	18475
NZ_AP018800.1	GCA_005044195	100	13154	81004	13154	100	0	0	10274	23427	1	13154	0	24291
NZ_CP031909.1	GCA_005044225	100	27422	73224	27422	100	0	0	15435	42856	1	27422	0	50639
NZ_CP027545.1	GCA_005044275	99,971	27860	101089	27859	100	3	5	11075	38930	27859	1	0	51398
NZ_CP027386.1	GCA_005044315	99,978	17842	54452	17842	100	1	1	25857	43695	17842	1	0	32923
AP019709.1	GCA_005044325	99,994	17134	86874	17134	100	1	0	16752	33885	17134	1	0	31636
NZ_CP027444.1	GCA_005044375	99,95	22171	91399	22171	100	7	2	25056	47222	1	22171	0	40878
AP019709.1	GCA_005044395	99,994	17134	86874	17134	100	1	0	16752	33885	17134	1	0	31636
NZ_CP051657.1	GCA_005044405	99,988	16716	74390	16716	100	2	0	19269	35984	1	16716	0	30858
NZ_CP022408.1	GCA_005044435	100	15474	81950	15474	100	0	0	51717	67190	1	15474	0	28576
NZ_CP031909.1	GCA_005044445	100	15944	73224	16021	100	0	0	53193	69136	16021	78	0	29444
NZ_CP027545.1	GCA_005044455	99,979	28990	101089	29066	100	1	5	9945	38930	28989	1	0	53496
NZ_CP024055.1	GCA_005044505	100	11649	88839	11649	100	0	0	55578	67226	11649	1	0	21512
NZ_CP009105.1	GCA_005044555	99,972	25060	168318	25058	100	4	3	6675	31733	25058	1	0	46239
NZ_CP027585.1	GCA_005044645	99,995	19761	160576	19761	100	1	0	120947	140707	19761	1	0	36487
CP027674.1	GCA_005044695	99,894	8464	133420	8536	99	3	3	20455	28918	1	8458	0	15575
NZ_CP027385.1	GCA_005044785	99,989	8916	118259	8916	100	1	0	66709	75624	1	8916	0	16460
NZ_CP022408.1	GCA_005044825	100	15474	81950	15474	100	0	0	51717	67190	15474	1	0	28576
AP019704.1	GCA_005044845	99,968	18609	92337	18609	100	3	1	16752	35357	18609	1	0	34328
NZ_CP027583.1	GCA_005044895	99,986	22153	88339	22153	100	3	0	42236	64388	22153	1	0	40893
AP019709.1	GCA_005044935	99,982	17134	86874	17134	100	3	0	16752	33885	17134	1	0	31624
CP027674.1	GCA_005044945	99,899	7894	133420	8048	98	7	1	104898	112790	7969	76	0	14532
AP019709.1	GCA_005044995	99,994	17134	86874	17134	100	1	0	16752	33885	1	17134	0	31636
NZ_CP027386.1	GCA_005045015	99,978	17842	54452	17842	100	1	1	25857	43695	1	17842	0	32923
CP042949.1	GCA_005045035	99,996	26176	118482	26176	100	1	0	59557	85732	1	26176	0	48333
NZ_CP006263.1	GCA_005045125	99,957	11743	98066	11876	99	5	0	35689	47431	11820	78	0	21658
CP027581.1	GCA_005045135	99,94	31824	118822	31878	100	16	3	80487	112309	31822	1	0	58659
NZ_CP027375.1	GCA_005045145	99,995	19874	118863	19874	100	1	0	47409	67282	19874	1	0	36695
NZ_CP013028.1	GCA_005045155	99,994	16351	74656	16351	100	1	0	34125	50475	1	16351	0	30190
NZ_CP061760.1	GCA_005045215	99,971	59595	172576	59594	100	15	2	65203	124796	1	59594	0	110000
NZ_CP009107.1	GCA_005045225	100	32560	161447	32560	100	0	0	76450	109009	32560	1	0	60128
NZ_CP027451.1	GCA_005045235	99,935	57185	173649	57185	100	3	4	43648	100798	1	57185	0	105400
NZ_CP023542.1	GCA_005045315	97,902	17825	161452	17796	100	314	37	18771	36564	17796	1	0	30790
NC_013369.1	GCA_005045325	99,974	18952	85167	19631	97	2	1	17174	36122	1	18952	0	34967
NZ_CP023674.1	GCA_005045365	99,969	12731	87524	12731	100	4	0	57240	69970	1	12731	0	23488
NZ_CP027549.1	GCA_005045385	99,958	11882	94116	11882	100	3	2	57644	69523	11882	1	0	21913
NZ_CP027354.1	GCA_005045445	99,928	13947	57720	14025	99	3	5	19591	33530	14025	79	0	25693
NZ_CP006263.1	GCA_005045475	100	9208	98066	9208	100	0	0	83727	92934	9208	1	0	17005
AP019704.1	GCA_005045505	99,968	22168	92337	22161	100	0	1	16752	38919	1	22161	0	40891
NZ_CP024055.1	GCA_005045575	99,493	6506	88839	6683	97	22	3	16244	22739	6583	79	0	11821
NZ_CP027446.1	GCA_005045595	99,975	16119	74269	16119	100	4	0	7143	23261	16119	1	0	29745
AP019709.1	GCA_005045625	99,994	17132	86874	17132	100	1	0	16754	33885	1	17132	0	31632
NZ_CP027343.1	GCA_005045645	99,987	37154	131410	37154	100	1	4	32957	70106	37154	1	0	68580
NZ_CP027391.1	GCA_005045665	99,978	17842	98724	17842	100	1	1	22048	39886	1	17842	0	32923
NZ_CP061760.1	GCA_005045685	99,548	13488	172576	13489	100	55	6	144109	157592	3	13488	0	24565
CP027674.1	GCA_005045715	99,857	8400	133420	8474	99	7	3	20382	28781	1	8395	0	15440
CP027641.1	GCA_005045805	99,778	20684	126957	20683	100	31	3	28621	49290	1	20683	0	37927
NZ_CP027549.1	GCA_005045815	99,985	20189	94116	20189	100	1	2	15379	35565	20189	1	0	37264
NZ_CP027385.1	GCA_005045955	100	8568	118259	8568	100	0	0	67057	75624	1	8568	0	15823
AP019709.1	GCA_005045975	100	17289	86874	17289	100	0	0	16752	34040	17289	1	0	31927
NZ_AP018805.1	GCA_005045995	99,974	11568	78434	11644	99	2	1	13536	25102	1	11568	0	21344
CP027674.1	GCA_005046005	99,908	26097	133420	26093	100	20	4	103988	130084	1	26093	0	48056
CP027674.1	GCA_005046365	99,368	8224	133420	8221	100	49	3	37241	45464	8221	1	0	14896
CP042949.1	GCA_005046415	99,985	19900	118482	19900	100	3	0	59557	79456	1	19900	0	36732
NZ_CP006263.1	GCA_005046455	99,955	8876	98066	8876	100	4	0	84057	92932	1	8876	0	16369
NZ_CP027318.1	GCA_005046485	99,915	18807	81954	18806	100	15	1	52068	70874	1	18806	0	34640
NZ_CP027385.1	GCA_005046525	99,988	8579	118259	8579	100	1	0	67046	75624	8579	1	0	15837
NC_013354.1	GCA_005046555	100	15315	75546	15969	96	0	0	1	15315	655	15969	0	28282
NC_013354.1	GCA_005046595	100	15315	75546	15971	96	0	0	1	15315	657	15971	0	28282
NC_013354.1	GCA_005046605	99,98	15315	75546	15970	96	3	0	1	15315	15315	1	0	28265
NC_013354.1	GCA_005046615	99,993	15314	75546	15969	96	1	0	1	15314	656	15969	0	28275
NZ_CP031909.1	GCA_005046675	100	13552	73224	13552	100	0	0	15438	28989	13552	1	0	25026
NZ_CP024480.1	GCA_005046685	99,997	31483	77062	31684	99	1	0	20629	52111	31684	202	0	58133
NZ_CP027354.1	GCA_005046735	99,934	15183	57720	15183	100	2	7	15777	30951	15183	1	0	27976
NZ_CP027354.1	GCA_005046745	99,949	15724	57720	15802	100	1	7	17814	33530	15802	79	0	28986
NZ_CP031899.1	GCA_005046765	99,98	39821	160712	39821	100	7	1	69691	109510	1	39821	0	73490
NZ_CP027588.1	GCA_005046855	99,541	11331	58109	11854	96	34	8	44351	55664	219	11548	0	20620
CP042949.1	GCA_005046865	99,987	15044	118482	15044	100	2	0	28336	43379	1	15044	0	27771
NZ_CP027588.1	GCA_005046885	99,541	11331	58109	11854	96	34	8	44351	55664	11636	307	0	20620
NC_013354.1	GCA_005046895	100	15315	75546	15970	96	0	0	1	15315	15315	1	0	28282
NZ_CP027385.1	GCA_005046905	99,902	18425	118259	18503	100	16	2	87886	106308	18425	1	0	33924
NZ_CP027318.1	GCA_005380525	100	18980	81954	19459	98	0	0	51848	70827	19459	480	0	35050
NZ_CP027456.1	GCA_005380645	99,994	15914	79682	15914	100	1	0	48723	64636	15914	1	0	29385
NZ_CP027318.1	GCA_005380705	99,979	19251	81954	20248	95	4	0	51846	71096	19251	1	0	35534
NZ_CP022408.1	GCA_005380745	99,994	15989	81950	15989	100	1	0	51497	67485	1	15989	0	29523
NZ_CP027456.1	GCA_005380765	100	15914	79682	15914	100	0	0	48723	64636	15914	1	0	29388
NZ_CP022408.1	GCA_005380785	99,974	19364	81950	19364	100	5	0	18155	37518	19364	1	0	35741
NZ_CP027456.1	GCA_005380805	100	15914	79682	15914	100	0	0	48723	64636	15914	1	0	29388
NZ_CP022408.1	GCA_005380825	99,958	19238	81950	19248	100	8	0	18165	37402	19248	11	0	35495
NZ_CP027456.1	GCA_005380865	100	15914	79682	15914	100	0	0	48723	64636	15914	1	0	29388
NZ_CP027456.1	GCA_005380905	100	15914	79682	15914	100	0	0	48723	64636	15914	1	0	29388
NZ_CP027456.1	GCA_005380945	100	15914	79682	15914	100	0	0	48723	64636	15914	1	0	29388
NZ_CP022408.1	GCA_005380965	99,875	19992	81950	19978	100	11	1	18155	38146	19978	1	0	36778
NZ_CP027456.1	GCA_005380985	100	15914	79682	15914	100	0	0	48723	64636	15914	1	0	29388
NZ_CP022408.1	GCA_005381005	99,974	19238	81950	19248	100	5	0	18165	37402	19248	11	0	35506
NZ_CP027318.1	GCA_005381125	99,974	19233	81954	19247	100	5	0	51862	71094	19247	15	0	35495
NZ_CP027456.1	GCA_005381145	100	15914	79682	15914	100	0	0	48723	64636	15914	1	0	29388
NZ_CP022408.1	GCA_005381165	99,959	19364	81950	19364	100	8	0	18155	37518	19364	1	0	35730
NZ_CP027318.1	GCA_005381185	99,969	19363	81954	19363	100	6	0	51848	71210	19363	1	0	35733
NZ_CP027436.1	GCA_005381205	99,979	19248	83611	19248	100	4	0	10444	29691	19248	1	0	35528

NZ_CP027456.1	GCA_005381225	100	15914	79682	15914	100	0	0	48723	64636	15914	1	0	29388
NZ_CP027456.1	GCA_005381245	100	15914	79682	15914	100	0	0	48723	64636	15914	1	0	29388
NZ_CP027456.1	GCA_005381265	99,994	15914	79682	15914	100	1	0	48723	64636	15914	1	0	29383
NZ_CP022408.1	GCA_005381305	99,979	19431	81950	19431	100	4	0	18155	37585	19431	1	0	35868
NZ_CP027318.1	GCA_005381345	99,995	19248	81954	19248	100	1	0	51848	71095	19248	1	0	35539
CP057155.1	GCA_005383905	99,943	12315	168860	12512	98	7	0	147811	160125	141	12455	0	22703
NZ_CP051657.1	GCA_005383925	99,994	15675	74390	15675	100	0	1	58652	74325	15675	1	0	28939
NZ_CP061760.1	GCA_005383985	99,974	60446	172576	60446	100	15	1	64653	125097	60446	1	0	111500
NZ_CP009107.1	GCA_005384005	99,934	19585	161447	19582	100	10	1	89485	109069	1	19582	0	36092
NZ_CP027372.1	GCA_005384025	99,974	81762	175427	81762	100	18	2	81990	163748	1	81762	0	150900
NZ_CP027583.1	GCA_005384045	99,984	19152	88339	19283	99	1	2	18357	37507	19283	133	0	35349
NZ_CP042296.1	GCA_005384225	99,988	16642	167256	16641	100	1	1	42807	59448	1	16641	0	30720
AP019709.1	GCA_005390425	99,996	22468	86874	22468	100	1	0	16706	39173	22468	1	0	41486
NZ_CP027343.1	GCA_005390485	99,546	14751	131410	15289	96	54	10	52583	67328	418	15160	0	26856
NZ_CP028382.1	GCA_005390505	100	35775	160675	35775	100	0	0	92483	128257	35775	1	0	66065
NZ_CP027343.1	GCA_005390525	100	10115	131410	10115	100	0	0	71113	81227	10115	1	0	18679
NZ_CP028382.1	GCA_005390545	99,994	35975	160675	35975	100	2	0	92480	128454	35975	1	0	66423
NZ_CP027372.1	GCA_005390585	99,973	77638	175427	77638	100	18	2	81993	159627	1	77638	0	143300
NZ_CP061760.1	GCA_005390605	99,975	64222	172576	64351	100	15	1	60608	124828	64351	130	0	118500
NZ_CP015246.1	GCA_005390885	99,974	19297	118513	19297	100	5	0	59086	78382	19297	1	0	35608
NZ_CP027549.1	GCA_005390905	99,983	17577	94116	17577	100	1	2	18042	35616	17577	1	0	32441
NZ_CP027343.1	GCA_005391065	99,991	42321	131410	42321	100	1	3	38910	81227	1	42321	0	78127
NZ_CP027451.1	GCA_005391085	99,947	79297	173649	79419	100	12	8	42249	121534	79419	142	0	146200
NZ_CP012501.1	GCA_005392005	99,993	28153	242187	28198	100	2	0	93029	121181	28153	1	0	51978
NZ_CP027364.1	GCA_005392045	99,976	16781	74659	16781	100	1	3	14269	31046	1	16781	0	30963
NZ_CP040306.1	GCA_005392065	99,985	38881	95621	39172	99	2	3	14810	53688	294	39172	0	71763
NZ_CP006263.1	GCA_005392105	99,979	9382	98066	9382	100	2	0	83642	93023	9382	1	0	17315
NZ_CP027343.1	GCA_005392205	99,529	14861	131410	15562	95	55	12	52583	67436	15113	261	0	27041
NZ_CP012499.1	GCA_005392225	99,991	22028	223952	22027	100	1	1	83693	105720	22027	1	0	40666
NZ_CP012501.1	GCA_005392265	99,993	28158	242187	28322	99	2	0	93029	121186	28158	1	0	51988
NZ_CP023164.1	GCA_005392325	99,672	7928	122641	7928	100	25	1	12168	20094	7928	1	0	14495
NZ_CP006263.1	GCA_005392545	99,989	9376	98066	9376	100	1	0	83643	93018	1	9376	0	17309
NZ_CP027343.1	GCA_005392745	99,536	14860	131410	15579	95	54	12	52583	67436	15122	272	0	27045
NZ_CP027343.1	GCA_005392805	99,542	14860	131410	15550	96	54	11	52583	67436	15108	257	0	27052
NZ_CP006263.1	GCA_005392825	99,968	9365	98066	9365	100	3	0	83651	93015	1	9365	0	17278
NZ_CP027343.1	GCA_005392955	99,529	14861	131410	15560	96	55	12	52583	67436	449	15301	0	27041
NZ_CP027588.1	GCA_005393035	99,972	28308	58109	29493	96	7	1	1	28307	28308	1	0	52229
NZ_CP027441.1	GCA_005393415	99,657	12812	173714	13046	98	37	5	27632	40439	1	12809	0	23409
NZ_CP012501.1	GCA_005393545	99,946	7390	242187	7503	98	4	0	130206	137595	7503	114	0	13625
NZ_CP037942.1	GCA_005393905	99,991	11718	157534	11718	100	1	0	93422	105139	1	11718	0	21634
NZ_CP006028.1	GCA_005393945	99,994	16520	87120	16657	99	1	0	17445	33964	138	16657	0	30502
AP019709.1	GCA_005393965	100	19736	86874	19736	100	0	0	43945	63680	19736	1	0	36446
NZ_CP037942.1	GCA_005394045	99,959	12053	157534	12053	100	5	0	61331	73383	12053	1	0	22231
NZ_CP027343.1	GCA_005394065	99,991	10718	131410	10718	100	1	0	70520	81237	10718	1	0	19787
NZ_CP027463.1	GCA_005394205	99,917	13330	125059	13329	100	10	1	23129	36458	13329	1	0	24554
NZ_CP027451.1	GCA_005394305	99,988	25053	173649	25053	100	1	2	42247	67297	1	25053	0	46246
CP027581.1	GCA_005394365	99,995	20317	118822	20317	100	1	0	33493	53809	1	20317	0	37513
NZ_CP061760.1	GCA_005394425	99,978	60203	172576	60203	100	12	1	64657	124858	60203	1	0	111100
NZ_CP027451.1	GCA_005394505	99,977	26433	173649	26430	100	2	4	96434	122865	26430	1	0	48776
NZ_CP045828.1	GCA_005394585	99,92	8738	100778	8738	100	7	0	57266	66003	8738	1	0	16098
NZ_CP045828.1	GCA_005394785	99,92	8764	100778	9062	97	7	0	57234	65997	1	8764	0	16146
NZ_CP027441.1	GCA_005394825	99,9	81774	173714	81773	100	80	2	76998	158770	1	81773	0	150600
NZ_CP027576.1	GCA_005394845	99,882	16909	78427	17588	96	18	2	23451	40357	17588	680	0	31113
NZ_CP027451.1	GCA_005394865	99,927	80539	173649	80520	100	5	8	42251	122754	1	80520	0	148400
NZ_CP061760.1	GCA_005394885	99,967	60101	172576	60100	100	18	2	64759	124858	1	60100	0	110900
NZ_CP027370.1	GCA_005394905	99,946	18663	176149	18663	100	6	4	44535	63193	1	18663	0	34406
NZ_CP023164.1	GCA_005394985	99,74	8077	122641	8217	98	20	1	12078	20153	8077	1	0	14798
NZ_CP061760.1	GCA_005395005	99,975	64264	172576	64264	100	15	1	60596	124858	64264	1	0	118600
NZ_CP061760.1	GCA_005395045	99,977	60201	172576	60201	100	13	1	64658	124857	60201	1	0	111100
NZ_CP023164.1	GCA_005395645	99,877	13060	122641	13060	100	15	1	24234	37292	1	13060	0	24027
NZ_CP027441.1	GCA_005395805	99,97	76998	173714	76999	100	21	2	76983	153978	1	76998	0	142100
NZ_CP024291.1	GCA_005395845	99,968	15531	111697	15685	99	4	1	35498	51028	15530	1	0	28651
NZ_CP024480.1	GCA_005396825	99,994	17286	77062	17571	98	1	0	38993	56278	17428	143	0	31916
AP019709.1	GCA_005396985	100	22508	86874	22508	100	0	0	16686	39193	22508	1	0	41565
NZ_CP061760.1	GCA_005397025	99,977	60207	172576	60207	100	13	1	64655	124860	60207	1	0	111100
NZ_CP027343.1	GCA_005397045	99,84	12535	131410	12818	98	18	2	53521	66054	142	12675	0	23036
NZ_CP006028.1	GCA_005397085	99,982	16528	87120	16672	99	3	0	17445	33972	145	16672	0	30505
NZ_CP009107.1	GCA_005397145	99,784	15720	161447	15692	100	6	2	43461	59180	1	15692	0	28816
NZ_CP028382.1	GCA_005397165	99,995	36982	160675	36982	100	2	0	92463	129444	1	36982	0	68282
NZ_CP006263.1	GCA_005397845	99,906	11748	98066	12153	97	6	1	35689	47431	11844	97	0	21629
NZ_CP028684.1	GCA_005398325	99,991	42331	92725	42501	100	3	1	7914	50244	42501	172	0	78147
NZ_CP012501.1	GCA_005398785	99,848	9190	242187	9491	97	11	3	130445	139632	303	9491	0	16890
NZ_CP024055.1	GCA_005400765	99,802	6582	88839	6777	97	11	2	16323	22903	6581	1	0	12081
AP019709.1	GCA_007644155	99,994	17083	86874	17083	100	1	0	16754	33836	17083	1	0	31541
NZ_CP027354.1	GCA_007647045	99,934	15169	57720	15169	100	4	6	17894	33056	1	15169	0	27952
NZ_CP027549.1	GCA_007647955	99,958	11878	94116	11878	100	3	2	57646	69521	1	11878	0	21906
AP019704.1	GCA_007648995	99,986	22169	92337	22169	100	2	1	16754	38921	22169	1	0	40921
NZ_CP037942.1	GCA_007649015	99,983	11855	157534	11855	100	2	0	93485	105339	1	11855	0	21882
CP043020.1	GCA_008122355	99,991	86913	91448	91447	95	3	4	4538	91448	91447	4538	0	160400
CP043020.1	GCA_008122425	99,996	45273	91448	45381	100	1	1	8909	54181	45272	1	0	83591
AP019709.1	GCA_008633505	99,996	23762	86874	23762	100	1	0	16765	40526	1	23762	0	43875
AP019709.1	GCA_008633525	100	22532	86874	22532	100	0	0	16674	39205	1	22532	0	41609
AP019709.1	GCA_008633545	99,981	26907	86874	26906	100	2	3	12427	39331	1	26906	0	49657
AP019709.1	GCA_008633565	100	22352	86874	22352	100	0	0	16665	39016	22352	1	0	41277
NZ_CP027436.1	GCA_008633585	99,924	15888	83611	15888	100	12	0	13783	29670	1	15888	0	29274
NZ_CP031923.1	GCA_008633605	99,99	20527	95298	20527	100	2	0	64138	84664	1	20527	0	37896
AP019709.1	GCA_008633625	99,996	22598	86874	22598	100	1	0	16700	39297	22598	1	0	41726
NZ_CP037944.1	GCA_008633645	99,995	19308	88848	19445	99	1	0	49310	68617	19445	138	0	35650
AP019704.1	GCA_008633665	99,964	22494	92337	22486	100	0	1	16689	39182	1	22486	0	41487
AP019704.1	GCA_008633685	99,962	23703	92337	23695	100	1	1	16696	40398	23695	1	0	43715
AP019709.1	GCA_008633705	99,991	22484	86874	22484	100	2	0	16698	39181	22484	1	0	41510
NZ_CP031923.1	GCA_008633725	99,99	19730	95298	19730	100	2	0	64161	83890	1	19730	0	36424
AP019707.1	GCA_008633845	99,987	22566	91036	22565	100	2	1	16661	39226	22565	1	0	41654

AP019709.1	GCA_008633885	99,991	22546	86874	22546	100	2	0	16667	39212	1	22546	0	41624
AP019709.1	GCA_008633985	99,991	22556	86874	22556	100	2	0	16662	39217	22556	1	0	41643
NZ_CP031923.1	GCA_008634065	99,995	20538	95298	20538	100	1	0	64132	84669	20538	1	0	37922
AP019709.1	GCA_008634095	100	22544	86874	22544	100	0	0	16668	39211	1	22544	0	41631
AP019709.1	GCA_008634125	99,991	22554	86874	22554	100	2	0	16663	39216	1	22554	0	41639
AP019709.1	GCA_008634145	99,995	18978	86874	18978	100	1	0	43949	62926	18978	1	0	35041
AP019709.1	GCA_008634165	100	22494	86874	22494	100	0	0	16693	39186	22494	1	0	41539
AP019709.1	GCA_008634185	100	22512	86874	22512	100	0	0	16684	39195	1	22512	0	41572
AP019709.1	GCA_008634205	100	22490	86874	22490	100	0	0	16695	39184	1	22490	0	41532
AP019709.1	GCA_008634225	100	24825	86874	24825	100	0	0	16695	41519	24825	1	0	45844
AP019709.1	GCA_008634265	99,991	22484	86874	22484	100	2	0	16698	39181	22484	1	0	41510
AP019709.1	GCA_008634305	99,969	22497	86874	22497	100	0	1	16695	39184	1	22497	0	41499
AP019707.1	GCA_008634325	99,992	23734	91036	23734	100	2	0	16697	40430	23734	1	0	43818
AP019709.1	GCA_008634345	100	22328	86874	22328	100	0	0	16677	39004	22328	1	0	41233
AP019709.1	GCA_008634365	99,996	23162	86874	23162	100	1	0	40528	63689	23162	1	0	42767
AP019709.1	GCA_008634445	99,996	24821	86874	24821	100	1	0	16697	41517	24821	1	0	45831
AP019709.1	GCA_008634465	100	22476	86874	22476	100	0	0	16702	39177	1	22476	0	41506
CP027321.1	GCA_008634485	99,995	19373	84276	19373	100	0	1	46482	65853	1	19373	0	35768
AP019709.1	GCA_008634505	100	22466	86874	22466	100	0	0	16707	39172	1	22466	0	41487
AP019709.1	GCA_008634525	100	22452	86874	22452	100	0	0	16714	39165	22452	1	0	41462
AP019709.1	GCA_008634575	100	22466	86874	22466	100	0	0	16707	39172	22466	1	0	41487
AP019709.1	GCA_008634665	100	22364	86874	22364	100	0	0	16683	39046	1	22364	0	41299
AP019709.1	GCA_008635145	100	19689	86874	19689	100	0	0	43902	63590	19689	1	0	36359
AP019709.1	GCA_008635295	100	18998	86874	18998	100	0	0	43939	62936	18998	1	0	35083
AP019709.1	GCA_008635525	99,995	19790	86874	19790	100	1	0	43918	63707	19790	1	0	36540
AP019704.1	GCA_008635545	99,922	20586	92337	20586	100	15	1	48605	69189	1	20586	0	37925
AP019707.1	GCA_008635565	100	19912	91036	19912	100	0	0	48627	68538	1	19912	0	36771
AP019707.1	GCA_008635605	99,99	19834	91036	19833	100	1	1	48621	68454	1	19833	0	36614
AP019709.1	GCA_008635645	100	19836	86874	19836	100	0	0	43895	63730	19836	1	0	36631
AP019707.1	GCA_008635685	99,99	19842	91036	19841	100	1	1	48617	68458	19841	1	0	36629
NZ_CP027339.1	GCA_008635765	99,944	15965	92644	15965	100	1	8	29925	45881	15965	1	0	29425
AP019709.1	GCA_008635785	100	22563	86874	22563	100	0	0	16658	39220	22563	1	0	41667
AP019707.1	GCA_008635835	99,99	20321	91036	20321	100	2	0	48132	68452	1	20321	0	37515
AP019709.1	GCA_008635855	99,995	19826	86874	19826	100	1	0	43900	63725	19826	1	0	36607
AP019709.1	GCA_008635875	100	19832	86874	19832	100	0	0	43897	63728	1	19832	0	36623
AP019709.1	GCA_008635895	100	19826	86874	19826	100	0	0	43900	63725	19826	1	0	36612
NZ_CP027549.1	GCA_008635915	99,978	17862	94116	17902	100	2	2	17819	35678	17862	1	0	32961
AP019709.1	GCA_008635965	99,99	19822	86874	19822	100	2	0	43902	63723	1	19822	0	36594
AP019707.1	GCA_008636025	99,989	18467	91036	18646	99	1	1	50738	69204	18466	1	0	34090
NC_011350.1	GCA_008753435	99,983	41902	94644	41896	100	1	1	17292	59193	41896	1	0	77335
NZ_CP040108.1	GCA_008753445	99,994	30820	93190	31747	97	2	0	1	30820	30820	1	0	56903
NZ_CP017441.1	GCA_008753455	99,998	43057	92729	43057	100	1	0	7949	51005	43057	1	0	79506
NZ_CP035546.1	GCA_008753515	99,998	41869	94581	41869	100	0	1	33190	75057	41869	1	0	77311
NZ_CP038362.1	GCA_008753535	99,998	41869	95928	41869	100	1	0	34514	76382	1	41869	0	77312
NZ_CP008958.1	GCA_008753565	99,998	42291	92076	43053	98	1	0	10977	53267	43053	763	0	78092
NZ_CP038343.1	GCA_008753635	99,998	43072	92743	43071	100	0	1	7948	51019	1	43071	0	79532
NC_011350.1	GCA_008753655	100	22401	94644	22401	100	0	0	41100	63500	22401	1	0	41367
NZ_CP028640.1	GCA_008753715	99,995	43061	93179	43060	100	1	1	7948	51008	1	43060	0	79506
NZ_CP028616.1	GCA_008753725	99,997	31731	94495	32363	98	1	0	54006	85736	633	32363	0	58591
NZ_CP015022.1	GCA_008755365	99,994	33120	95170	33120	100	2	0	8918	42037	1	33120	0	61151
NZ_CP034793.1	GCA_008755385	99,978	40929	95421	41870	98	1	2	54493	95421	1	40921	0	75525
NZ_CP017441.1	GCA_008755395	99,997	39898	92729	39916	100	0	1	7949	47845	39916	19	0	73671
NZ_CP022408.1	GCA_008755455	100	20937	81950	20937	100	0	0	51669	72605	20937	1	0	38664
NC_013366.1	GCA_008755475	100	13956	77690	13956	100	0	0	52571	66526	1	13956	0	25772
NZ_CP027385.1	GCA_008755545	100	8635	118259	8635	100	0	0	66930	75564	1	8635	0	15946
NC_013366.1	GCA_008755645	100	13963	77690	13963	100	0	0	52570	66532	1	13963	0	25785
AP019709.1	GCA_008755655	99,996	22470	86874	22470	100	1	0	16704	39173	1	22470	0	41489
NZ_CP031909.1	GCA_008755735	100	36983	73224	36983	100	0	0	15389	52371	1	36983	0	68295
NZ_CP027385.1	GCA_008755765	100	9183	118259	9183	100	0	0	66490	75672	1	9183	0	16958
NC_013366.1	GCA_008755775	100	13965	77690	13965	100	0	0	52569	66533	13965	1	0	25789
NC_013366.1	GCA_008755835	100	14711	77690	14711	100	0	0	52676	67386	1	14711	0	27167
NC_013366.1	GCA_008755965	100	13035	77690	13035	100	0	0	52678	65712	13035	1	0	24072
NC_013366.1	GCA_008755975	100	13037	77690	13037	100	0	0	52677	65713	13037	1	0	24075
NC_013366.1	GCA_008756055	100	13035	77690	13035	100	0	0	52678	65712	13035	1	0	24072
NZ_CP027456.1	GCA_008756135	99,993	15352	79682	15352	100	1	0	49113	64464	15352	1	0	28345
NC_013366.1	GCA_008756205	100	13035	77690	13035	100	0	0	52678	65712	13035	1	0	24072
NC_013366.1	GCA_008756245	100	13035	77690	13035	100	0	0	52678	65712	1	13035	0	24072
NZ_CP027385.1	GCA_008756255	100	8880	118259	8880	100	0	0	66793	75672	1	8880	0	16399
NC_013366.1	GCA_008756325	100	8198	77690	8216	100	0	0	57515	65712	8198	1	0	15139
NZ_CP022408.1	GCA_008756525	100	20937	81950	20937	100	0	0	51669	72605	1	20937	0	38664
NC_013366.1	GCA_008756635	100	13037	77690	13037	100	0	0	52676	65712	1	13037	0	24075
NZ_CP031909.1	GCA_008756745	100	37013	73224	37013	100	0	0	15359	52371	37013	1	0	68351
NC_013354.1	GCA_008757055	99,987	15362	75546	16067	96	2	0	1	15362	706	16067	0	28358
NZ_CP031909.1	GCA_008757195	99,995	36985	73224	36985	100	2	0	15388	52372	36985	1	0	68288
NC_013354.1	GCA_008757335	99,987	15363	75546	16068	96	2	0	1	15363	706	16068	0	28360
NZ_CP040317.1	GCA_009495455	100	43000	93331	43000	100	0	0	7966	50965	1	43000	0	79407
NZ_CP028606.1	GCA_009495495	100	32310	95648	32310	100	0	0	54552	86861	32310	1	0	59666
CP043544.1	GCA_009647465	99,946	20481	82910	20477	100	6	3	27921	48400	1	20477	0	37755
NZ_CP009105.1	GCA_009821015	99,792	20638	168318	21296	97	23	5	11034	31654	21219	585	0	37857
NZ_CP034385.1	GCA_009896495	99,996	92700	92697	92700	100	1	3	1	92697	1	92700	0	171200
NZ_CP034385.1	GCA_009896505	99,995	92700	92697	92700	100	2	3	1	92697	1	92700	0	171200
